# US exceptionalism? International trends in midlife mortality

**DOI:** 10.1093/ije/dyae024

**Published:** 2024-03-21

**Authors:** Jennifer Beam Dowd, Katarzyna Doniec, Luyin Zhang, Andrea Tilstra

**Affiliations:** Leverhulme Centre for Demographic Science, Nuffield Department of Population Health, University of Oxford, Oxford, UK; Nuffield College, University of Oxford, Oxford, UK; Leverhulme Centre for Demographic Science, Nuffield Department of Population Health, University of Oxford, Oxford, UK; Nuffield College, University of Oxford, Oxford, UK; Office of Population Research, Princeton University, Princeton, USA; Leverhulme Centre for Demographic Science, Nuffield Department of Population Health, University of Oxford, Oxford, UK; Nuffield College, University of Oxford, Oxford, UK

**Keywords:** Mortality trends, midlife, USA, UK, Central and Eastern Europe, high-income countries, working-age mortality

## Abstract

**Background:**

Rising midlife mortality in the USA has raised concerns, particularly the increase in ‘deaths of despair’ (due to drugs, alcohol and suicide). Life expectancy is also stalling in other countries such as the UK, but how trends in midlife mortality are evolving outside the USA is less understood. We provide a synthesis of cause-specific mortality trends in midlife (25–64 years of age) for the USA and the UK as well as other high-income and Central and Eastern European (CEE) countries.

**Methods:**

We document trends in midlife mortality in the USA, UK and a group of 13 high-income countries in Western Europe, Australia, Canada and Japan, as well as seven CEE countries from 1990 to 2019. We use annual mortality data from the World Health Organization Mortality Database to analyse sex- and age-specific (25–44, 45–54 and 55–64 years) age-standardized death rates across 15 major cause-of-death categories.

**Results:**

US midlife mortality rates have worsened since 1990 for several causes of death including drug-related, alcohol-related, suicide, metabolic diseases, nervous system diseases, respiratory diseases and infectious/parasitic diseases. Deaths due to homicide, transport accidents and cardiovascular diseases have declined since 1990 but saw recent increases or stalling of improvements. Midlife mortality also increased in the UK for people aged 45–54 year and in Canada, Poland and Sweden among for those aged 25–44 years.

**Conclusions:**

The USA is increasingly falling behind not only high-income, but also CEE countries, some of which were heavily impacted by the post-socialist mortality crisis of the 1990s. Although levels of midlife mortality in the UK are substantially lower than those in the USA overall, there are signs that UK midlife mortality is worsening relative to that in Western Europe.

Key Messages

- We provide a comprehensive comparison of cause-specific midlife mortality across high-income and Central and Eastern European (CEE) countries.
- All-cause mortality for US males and females aged 25–44 years was the highest of all the countries, surpassing the CEE group by 2019.
- US females aged 25–44 years were the only group whose mortality was higher in 2019 compared with 1990 (a 3.7% increase).
- US midlife mortality relative to high-income peers has worsened significantly since 1990.
- UK mortality has also diverged from peer countries, especially for younger middle-aged groups and females.

## Introduction

High-income countries have experienced unprecedented improvements in life expectancy for >100 years, some of them at a pace of almost 2.5 years per decade.^1^ The seemingly unstoppable gains faltered in the 2010s with life expectancy reversals in the USA and stagnation in the UK,^2–4^ despite continued improvements for most countries. Before the COVID-19 pandemic, life expectancy losses in the USA were attributed to rising mortality in midlife due to slowing improvements in cardiovascular conditions and increases in deaths from ‘despair’-related causes, including drugs, alcohol and suicide.^4–9^ In the UK, stalling life expectancy in the 2010s has been attributed to austerity policies and declining health and social care investments, though these hypotheses are difficult to test empirically.^10^^,^^11^

Cross-country comparisons of overall life expectancy trends are well documented^12^^,^^13^ but less is known about comparative trends in midlife mortality. Although life expectancy changes in higher-income countries are often heavily influenced by mortality at older ages, when most deaths occur, trends in midlife mortality may foreshadow future trends in life expectancy if later-born cohorts continue to see higher mortality risk as they age. The post-socialist mortality crisis of the early 1990s also saw large increases in working-age mortality, including despair-related deaths that have been compared with the current US mortality crisis.^14–16^

We provide an international comparative perspective on midlife mortality to better understand recent life expectancy declines in the USA and stagnation in the UK. We analysed trends in cause-specific midlife mortality in the USA, the UK and a group of 16 high-income (HI) and 7 Central and Eastern European (CEE) countries from 1990 to 2019.

## Data and methods

We used cause-specific mortality and population counts from the World Health Organization (WHO) Mortality Database for 25 countries from 1990 up to the most recent year available. The 18 HI countries included: Austria, Australia, Belgium, Canada, Denmark, France, Finland, Germany, Italy, Japan, Netherlands, Norway, Portugal, Spain, Sweden, Switzerland, the UK and the USA. The seven CEE countries included Bulgaria, the Czech Republic, Hungary, Poland, Romania, Slovakia and Slovenia.

We examined all-cause mortality and 15 mutually exclusive major causes of death [see Supplementary Table S1, available as Supplementary data at *IJE* online, for the International Classification of Diseases, the Ninth and Tenth revisions’ (ICD-9/ICD-10) codes]: (i) infectious and parasitic diseases, excluding HIV/AIDS; (ii) HIV/AIDS; (iii) respiratory diseases; (iv) trachea/bronchus and lung cancers; (v) all other cancers; (vi) nervous system diseases; (vii) metabolic diseases; (viii) cardiovascular disease; (ix) suicide; (x) homicide; (xi) transport accidents; (xii) all other external causes; and (xiii) a residual category capturing all other causes of death. Due to the limitations of the simplified ICD-9 classification [ICD-9 basic tabulation list (BTL)] used by the WHO, we were not able to match ICD-9 BTL and ICD-10 codes for (xiv) drug-related and (xv) alcohol-related causes. We analysed trends for those two causes for 2000–19, when ICD-10 classification was introduced into most countries.

We calculated all-cause and cause-specific mortality rates as deaths per 100 000 individuals for three age groups (25–44, 45–54, 55–64 years) by country, sex and year. Rates were age-standardized within each age category using the 2013 European Standard Population. To smooth short-term fluctuations with small counts, we report 3-year moving averages and use a wider age range at the youngest ages. We also calculated the percentage change in mortality rates from the baseline for each year to show relative changes over time.

All analyses and results are fully reproducible and can be accessed here.

## Results

Because of the large number of age–sex–year–cause-of-death combinations, our figures highlight trends in the USA and UK compared with the mean of HI countries (excluding the USA and UK) and the mean of CEE countries, calculated as the arithmetic mean of individual country age-standardized mortality rates without population weighting. Full results for absolute death rates for each cause of death are available in Supplementary Figures S1–S16 (available as Supplementary data at *IJE* online). Relative mortality changes are available in Supplementary Figures S17–S38 (available as Supplementary data at *IJE* online). Figures for each individual country are available in Supplementary Figures S39–S134 (available as Supplementary data at *IJE* online).

### All-cause mortality

**Figure 1.**
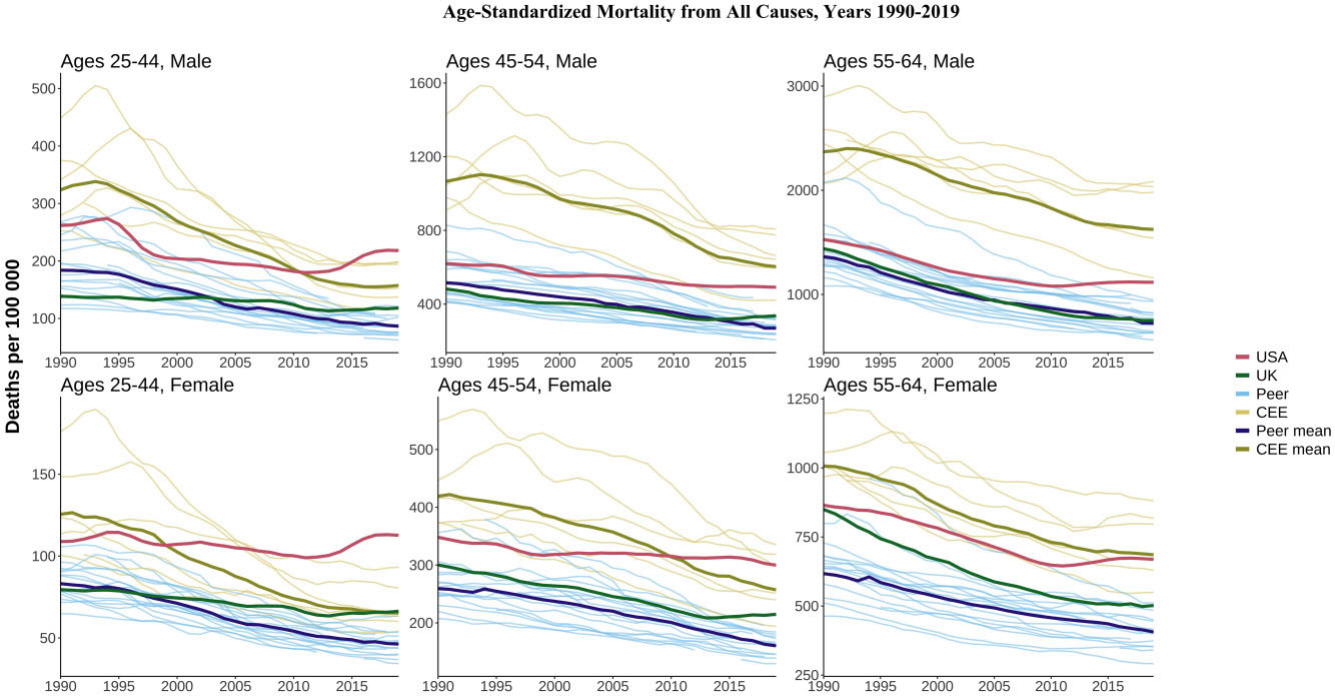
Age-standardized all-cause mortality by age and sex. CEE, Central and Eastern European country. ‘Peer’ indicates a high-income country comparable to the USA

Figure 1 shows trends in all-cause mortality rates for males and females in three age groups (25–44, 45–54, 55–64 years) in the 25 countries. Most countries have experienced substantial declines in midlife mortality since 1990, but at different speeds. CEE countries showed dramatic improvements over this period, recovering from the high mortality of the post-socialist mortality crisis. During the 1990s, the highest overall mortality rates among CEE countries were observed in Hungary, Poland, Romania and Bulgaria, with the lowest in Slovenia. Despite these improvements, the average mortality rate in the CEE group in 2019 remained substantially higher than the average for HI countries (about twice as high for CEE males and 1.5 times higher for CEE females, depending on the age group), excluding the USA and the UK.

US trends diverged substantially from other countries. In contrast to the consistent declines in HI peer and CEE countries, all-cause mortality trends in the USA were flat or increasing in most age and sex groups. The divergence was most noticeable for people aged 25–44 years, where US all-cause mortality rates rose above the CEE mean in the late 1990s for females and in 2011 for males. By 2019, US mortality was above the CEE mean in this age group and higher than in each CEE country. Compared with peer countries, the US all-cause rates in 2019 were 2.5 times higher for both males and females. At ages 45–54 years, all-cause mortality rates in 2019 were 88% higher for US females than the HI peer mean and 85% higher for US males compared with the peer mean. CEE countries consistently had the highest mortality for ages 55–64 years but US females in this age group were roughly equal to the CEE mean by 2019. Overall, US midlife mortality worsened relative to both peer and CEE countries, most noticeably for females and younger middle-aged groups.

**Figure 2.**
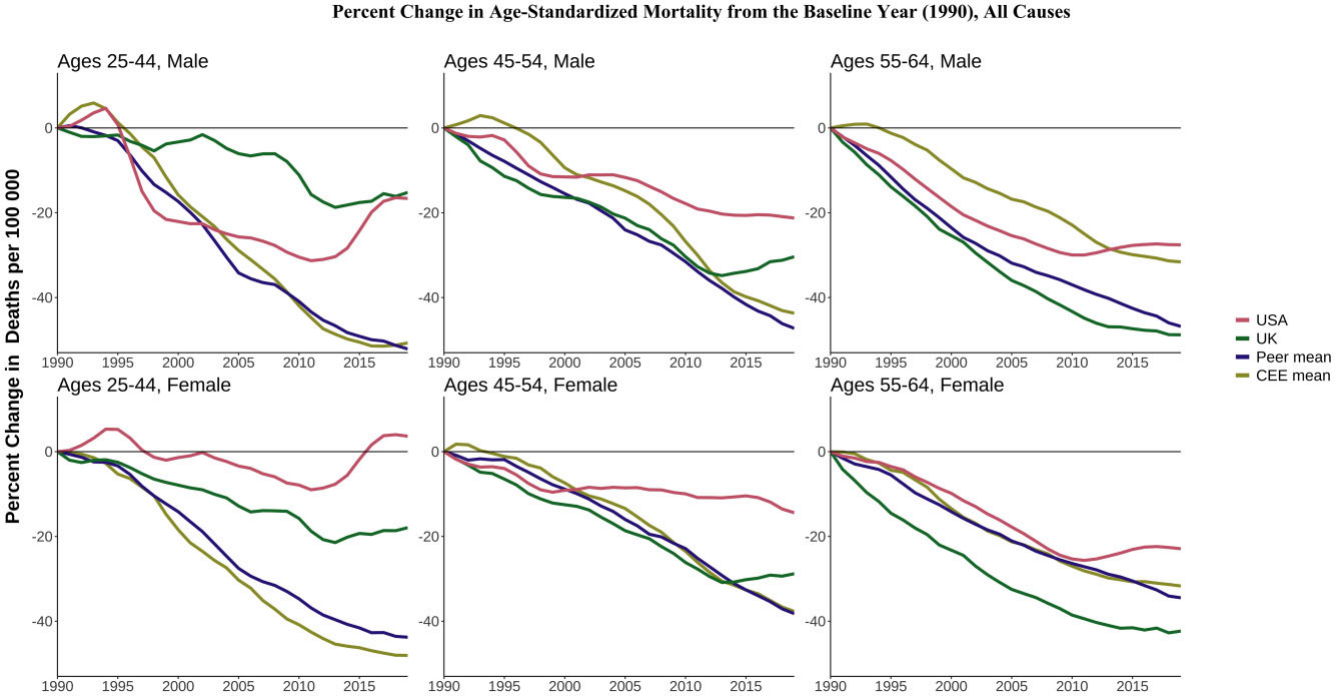
Percentage change in all-cause mortality rates since 1990. CEE, Central and Eastern European country. ‘Peer’ indicates a high-income country comparable to the USA

Figure 2 shows the percentage change in all-cause mortality rates across age, sex and country relative to 1990 (or the first year for which data are available). These relative changes highlight the lack of improvements among US women aged 25–44 years since 1990 and the recent stagnation of improvements compared with other countries in the age groups of 25–44 and 45–54 years. For those aged 55–64 years in the USA, relative mortality improvements tracked other countries until around 2010, when mortality improvements reversed.

The UK fared better than the USA but its performance worsened over time compared with its HI peers, particularly at the youngest ages. Mortality in UK males aged 25–44 years was mostly flat between 1990 and 2003 and then rose above the peer mean. UK male mortality at ages 45–54 years started increasing in 2015 in contrast to continued declines in peer countries. Despite the relative and absolute deterioration, the all-cause mortality rate for UK males aged 45–54 years in 2019 (329 deaths per 100 000) was well below that in the USA (490 per 100 000) and the CEE mean (594 per 100 000). Across the analysis years, UK females in younger midlife fared worse than peer and CEE countries. Death rates among UK people aged 25–44 and 45–54 years of both sexes were mostly stable rather than improving since the early 2010s, consistently with stalling life expectancy.

The USA and UK were not the only HI countries to have experienced rising or stalling midlife mortality. In Canada, all-cause mortality among people aged 25–44 years has risen since 2013 (from 101.3 deaths per 100 000 in 2013 to 118.7 in 2019 for males and 57.4 deaths per 100 000 in 2013 to 62.6 in 2019 for females). Poland and Sweden have seen small increases in midlife mortality in the past few years among males aged 25–44 years.

### Cause-specific mortality

**Figure 3.**
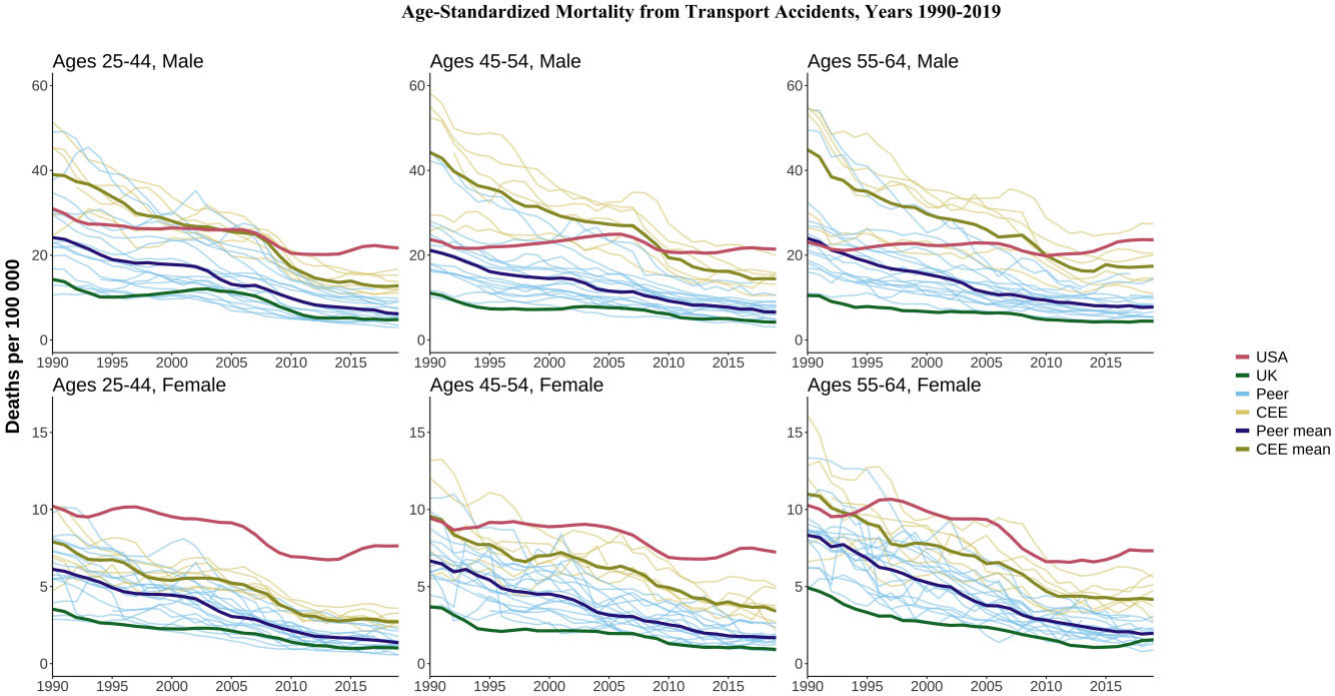
Age-standardized mortality rates from transport accidents. CEE, Central and Eastern European country. ‘Peer’ indicates a high-income country comparable to the USA

The USA stood apart with higher mortality for many causes compared with both HI peers and the CEE countries. Full results are available in Supplementary Figures S1–S16 (available as Supplementary data at *IJE* online). For example, whereas deaths from transport accidents have fallen since 1990 across most countries, the rates for US males aged 25–44 years stagnated in the late 1990s and early 2000s and have increased since 2010. By 2019, levels were ∼3.5 times as high as peer HI countries and 1.7 times as high as CEE countries (Figure 3). These patterns held at older ages and for females, where US mortality from transport accidents was well above the CEE mean for most of the period. In contrast, the UK had among the lowest death rates from transport accidents among all countries.

This negative US exceptionalism also carried over to deaths by homicide (Supplementary Figure S10, available as Supplementary data at *IJE* online). US homicide rates were far above those of all other countries for all age and sex groups. For males aged 25–44 years, the US homicide mortality rate was nearly 15 times higher than both the peer and CEE means in 2019.

### Suicide

**Figure 4.**
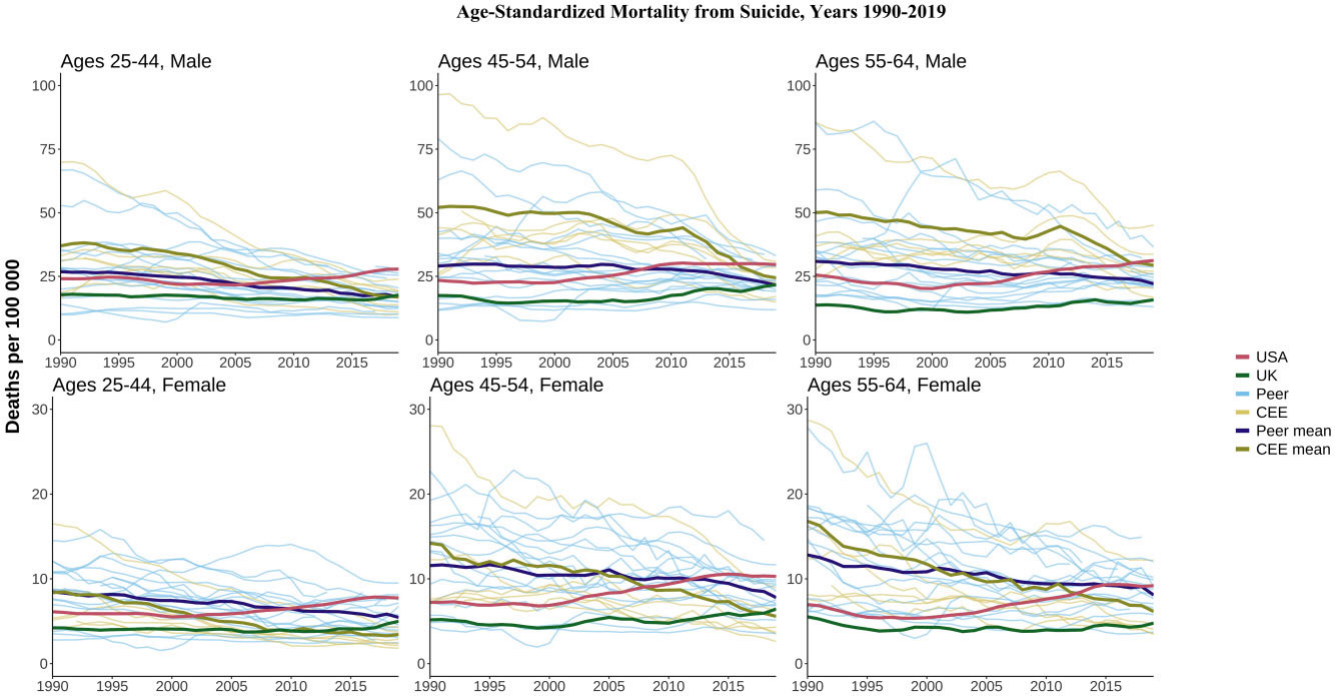
Age-standardized mortality rates from suicide. CEE, Central and Eastern European country. ‘Peer’ indicates a high-income country comparable to the USA

In 1990s, the USA had suicide mortality rates mostly below the HI peer mean (Figure 4). A gradual uptick since the late 1990s/early 2000s and continued declines elsewhere left the USA with rates above both peer and CEE means by 2019. The US divergence from peers was largest for males aged 25–44 years, where 2019 rates were 1.7 times higher than the peer mean and 1.7 times higher than the CEE mean. The UK suicide mortality was generally low compared with those of other countries, but there were some increases at younger ages in the 2010s, bringing it closer to HI peers and CEE means, but still far below that in the USA.

### Alcohol-related deaths

**Figure 5.**
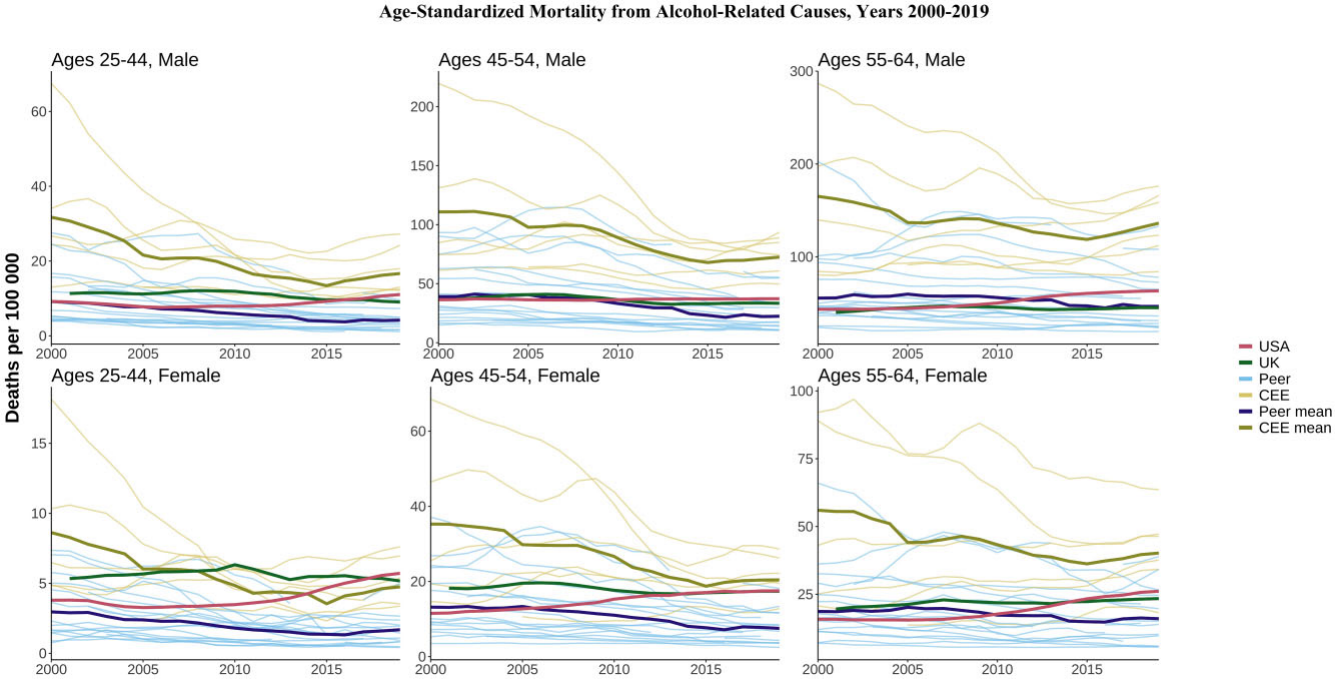
Age-standardized alcohol-related mortality. CEE, Central and Eastern European country. ‘Peer’ indicates a high-income country comparable to the USA

The CEE countries continued to have the highest levels of alcohol-related mortality, especially at older ages, but these have generally decreased over time (Figure 5). The USA began the period below the peer mean for alcohol-related deaths in most age and sex groups but has trended upward since the late 2000s, ending the period above both the UK and the peer mean in most age and sex categories. The UK’s alcohol-related mortality was higher than the peer mean in most age and sex categories but has remained mostly flat since the 2000s.

**Figure 6.**
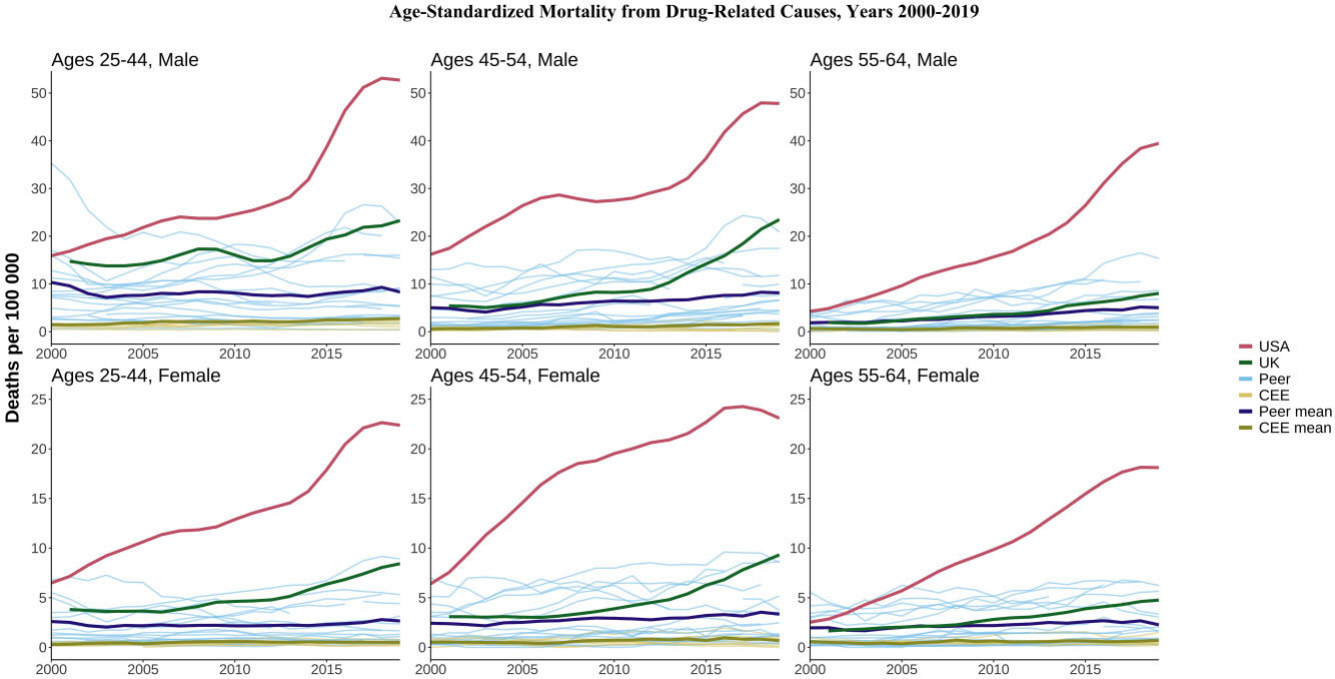
Age-standardized drug-related mortality. CEE, Central and Eastern European country. ‘Peer’ indicates a high-income country comparable to the USA

### Drug-related deaths

US drug-related mortality increased 3-fold in some age and sex groups and ≤10-fold in others between 2000 and 2019, diverging tremendously from other countries. The highest US levels were seen in males aged 45–54 years but the dramatic increases were universal across all age and sex groups. Drug deaths also increased in the UK during the late 2000s/early 2010s and worsened compared with peer means, but absolute levels were still much lower than those in the USA. Trends in drug-related deaths in the HI peer and CEE countries were relatively flat in most of the age and sex groups, confirming the unique nature of high drug-related mortality in the USA.

### Cardiovascular disease

**Figure 7.**
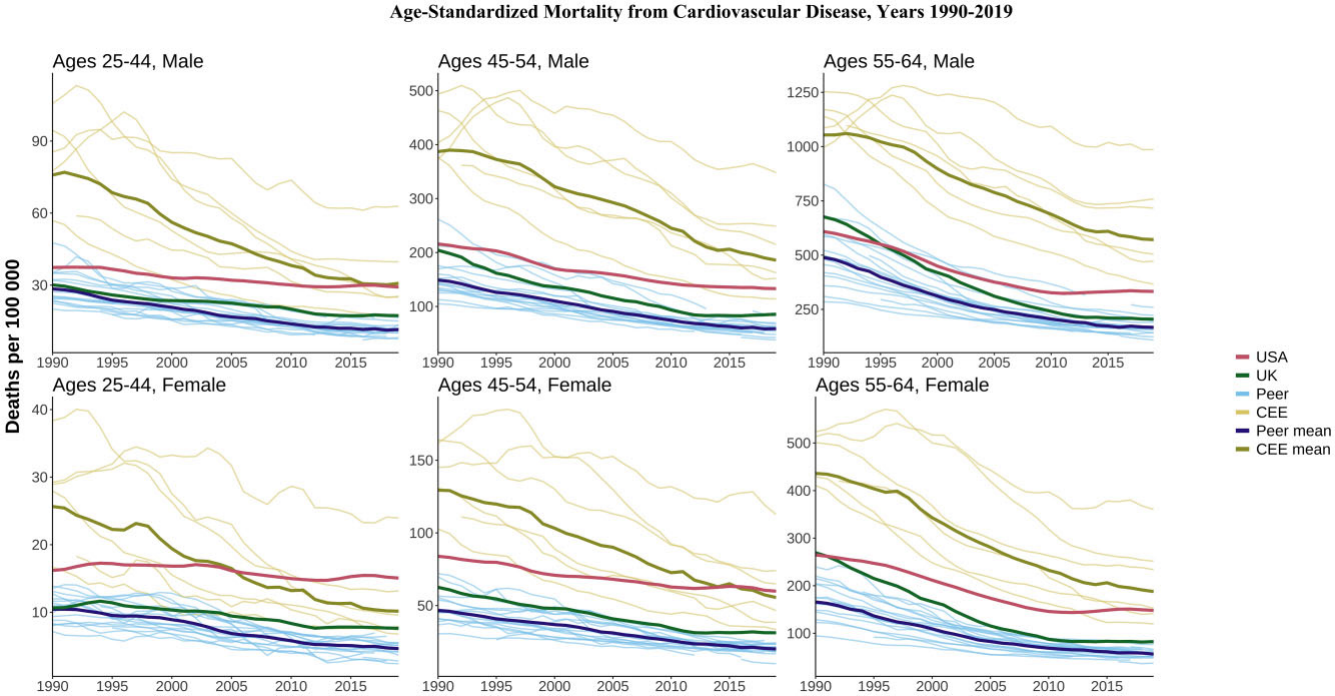
Age-standardized mortality from cardiovascular disease. CEE, Central and Eastern European country. ‘Peer’ indicates a high-income country comparable to the USA

Cardiovascular disease (CVD) mortality was highest in CEE countries in those aged 55–64 years but dramatic declines had brought the CEE closer to overall levels in the USA by 2019 (Figure 7). CVD mortality in the oldest group (55–64 years) in the USA was similar to or better than that in the UK in 1990, but both countries witnessed gradual declines followed by stagnation for both sexes by 2019. By 2019, CVD mortality in the USA in this age group was worse than in all peer countries and some individual CEE countries. The US stagnation in CVD mortality improvements was more evident at younger ages, particularly for females. By 2019, CVD mortality for US women aged 25–44 and 45–54 years was higher than the CEE mean despite much higher CEE levels in 1990. UK CVD mortality at the same ages was above the peer mean by 2019, with initial declines flattening out in recent years. Compared with those of its peers, the slower declines in the USA were particularly clear when looking at relative changes (Supplementary Figure S8, available as Supplementary data at *IJE* online), as is the relative stagnation for those aged 25–44 years in the UK.

### Lung cancer

**Figure 8.**
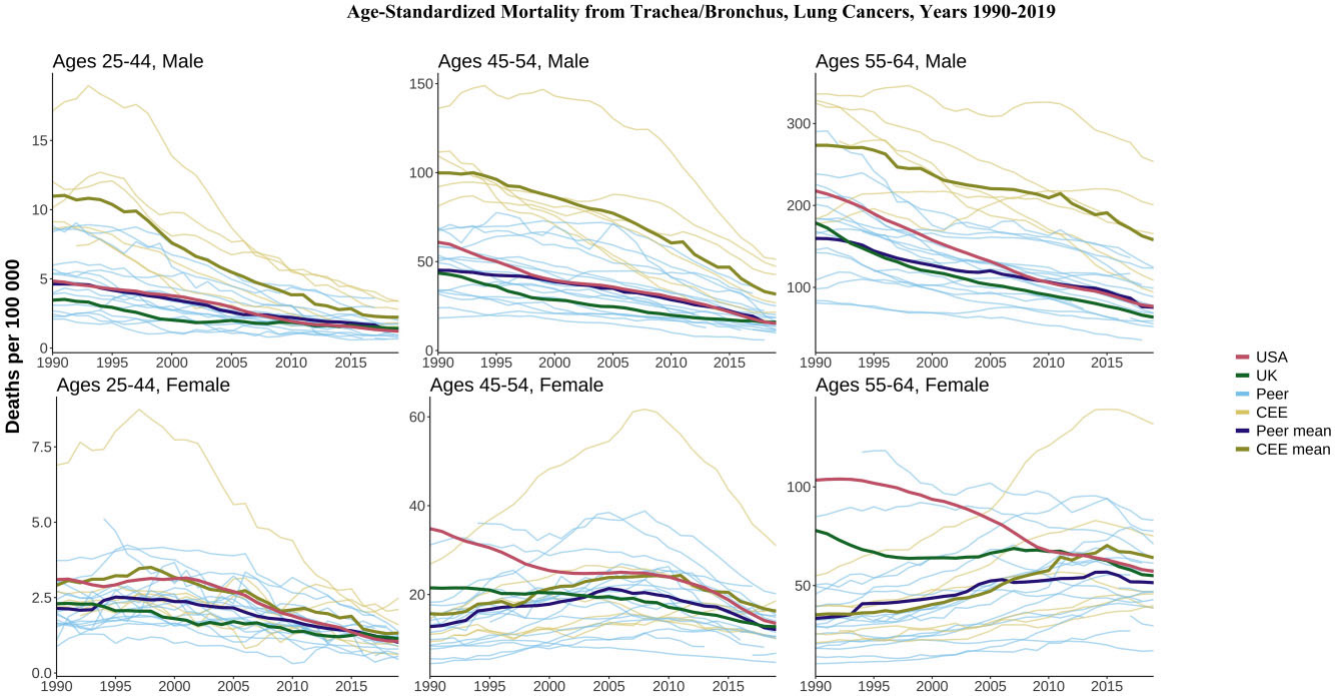
Age-standardized lung cancer mortality. CEE, Central and Eastern European country. ‘Peer’ indicates a high-income country comparable to the USA

Lung cancer mortality consistently declined in most countries for males across all ages, with levels in the CEE still higher in 2019 than in all HI peers (Figure 8). Patterns were more varied for females. For those aged 55–64 years, lung cancer mortality rose in the CEE and HI peer mean over this period, reflecting different smoking initiation and cessation patterns compared with men.^17^ US females aged 55–64 years saw substantial declines from their 1990 levels, which were much higher than the levels observed in peer countries. In contrast, UK females aged 55–64 years had rates that were above those of peers for most of this period.

### Other causes of death

Figures for all other cause-of-death categories are available in the Supplementary material (available as Supplementary data at *IJE* online). Mortality due to metabolic disease such as diabetes was noticeably higher in the USA compared with the UK, other HI peers and the CEE mean (Supplementary Figure S7, available as Supplementary data at *IJE* online). Similarly, deaths from respiratory diseases for females were higher in the USA and the UK than in HI peers or the CEE mean, and these deaths have trended upwards in the last decade in the USA. For males, the CEE countries had the highest levels of respiratory mortality throughout the period, but the levels in the USA and the UK were far above the HI mean (Supplementary Figure S3, available as Supplementary data at *IJE* online). Cancer was one cause of death for which the USA performed relatively well, with rates below those of the UK and the peer mean for most of the period, except for those aged 25–44 years (Supplementary Figure S5, available as Supplementary data at *IJE* online).

## Discussion

Over three decades, most countries have experienced declines in all-cause midlife mortality. The USA was the notable exception to this pattern, with increases in all-cause mortality for some age and sex groups over the smallest part of this period. Strikingly, all-cause mortality rates for females aged 25–44 years in the USA were higher in 2019 than in 1990. UK midlife mortality remained lower than that in the USA but fell behind compared with HI peers.

US midlife mortality rates worsened over the period for several causes of death, including drug-related, alcohol-related, suicide, metabolic diseases, nervous system diseases, respiratory diseases and infectious/parasitic diseases. Despite overall declines in the USA for homicide, transport accidents and cardiovascular disease, we documented recent increases or stalling improvements. This led to a deterioration of the relative standing of the USA, with mortality rates now surpassing the CEE countries for several causes—a pattern that is more pronounced for US females compared with males, and for younger age groups. The bright spot for the USA was cancer deaths, which were more comparable to peer countries.

Our findings are consistent with previous work showing the relative mortality disadvantage of the USA.^9^^,^^12^^,^^18^ Life expectancy is lower in the USA than in other HI countries and its standing has deteriorated substantially since 2010.^8^^,^^19^^,^^20^ The recent comprehensive US 2020 National Academy of Sciences report documented diverging life expectancy compared with peers from 1950 to 2016 and found that mortality in those aged 18–60 years contributed most to this gap.^4^ In particular, the diverging trends between the USA and 16 peer countries in age-standardized mortality among people aged 25–64 years has accelerated over time. We confirmed these findings and highlighted that the US disadvantage is most severe for those aged 25–44 and 45–54 years. Besides HI peers, we found that the USA increasingly lags behind CEE countries. Higher US mortality was especially noticeable for highly preventable external causes of death, including drug-related, homicide, suicide and transport accidents. Although narratives around ‘deaths of despair’ due to economic and social distress have garnered much attention,^5^^,^^21^^,^^22^ specific aspects of the US risk environment, especially concerning guns, vehicles and drugs, merit research and policy attention. In addition to external causes, the USA is falling behind due to stalling improvements in deaths from cardiovascular disease and from increases in deaths from metabolic, respiratory and nervous system causes. The US disadvantage in these causes suggests longer-term processes that contribute to chronic diseases such as high levels of obesity, which are more extreme in the USA compared with peer countries.^23^

There has been less work comparing trends in UK mortality to its peers. The rate of improvement in life expectancy slowed sharply in England and Wales from 2011 to 2016 relative to Organisation for Economic Co-operation and Development peer countries.^13^ Additionally, overall mortality rates in England and Wales in people aged 25–50 years have been appreciably higher than those in peer countries since the 2010s, consistently with our findings. Our results suggest that, although the UK performs relatively well on external causes such as suicide, homicide and transport accidents, this is countered by stalling improvements in cardiovascular disease and cancer, and drug deaths are also increasing. Our analyses did not separate the UK into its constituent countries but previous work has shown much higher midlife mortality in Scotland, with drug mortality in recent years even higher than it is in the USA.^24^ Overall, our results confirm the divergence of UK mortality from peer countries, which, like the USA, is more pronounced in the younger middle-aged groups and in females. Although the US mortality crisis has received substantial attention, a greater understanding of the causes behind the stagnation in the UK and prospects for the future is needed.

The mortality disadvantage of CEE countries has diminished significantly in the last 20 years. In the last decade, the mean death rate among CEE countries for some causes such as metabolic diseases, suicides and transport accidents have dropped below the mean rates of the HI group. The CEE countries, which have historically suffered from high levels of alcohol abuse, are again facing increasing midlife mortality from alcohol-related causes, but without rising drug-related mortality. On the other hand, HI countries experienced the inverse: alcohol-related deaths have been relatively stable over the last three decades, but drug-related mortality is gradually rising in several countries.

### Limitations

The analyses rely on the comparability of cause-specific mortality data across countries and some variations in the coding are likely. Harmonization between ICD-9 and ICD-10 coding schemes introduces an additional risk of bias, as the codes are not always directly translatable between classifications, such as for alcohol- and drug-related mortality. For most other causes of death, the comparability between coding schemes is high.^25^ Next, our data did not allow examination of variation in mortality by subgroups and regions within countries. Previous studies have suggested large mortality differences between socio-economic groups^26^^,^^27^ and regions in the USA and the UK^24^^,^^28^ so analysis at the national level alone may obscure important trends in certain groups.

Our study does not cover the years of the COVID-19 pandemic (2020 onwards), when the life expectancy gap between the USA and other HI countries widened.^29^^,^^30^ The contributions of midlife age groups to declines in life expectancy in 2020 and 2021 were more pronounced in the USA compared with other Western European countries, suggesting an increasing midlife US mortality disadvantage during the COVID pandemic. Deaths due to drug overdose increased significantly in the USA during the pandemic.^31^ Future studies should examine the effects of the disruption of the pandemic on long-term, cause-specific mortality trends when reliable cause-specific data become available.

## Conclusions

Worsening midlife mortality trends in the USA increasingly stand apart not only from HI peers, but also from Eastern Europe, which suffered a mortality crisis in the not-so-distant past. Although levels of midlife mortality in the UK are substantially lower than those in the USA, there are signs of trouble on the horizon relative to the rest of Europe. The more favourable mortality levels in HI peer countries imply significant room for mortality improvement in both the USA and the UK.

## Ethics approval

This research project does not require ethics approval as it uses only national-level vital statistics that are publicly available online.

## Supplementary Material

dyae024_Supplementary_Data

## Data Availability

Data used in the analyses of this manuscript are publicly available at https://www.who.int/data/data-collection-tools/who-mortality-database.
