# Supplementary material for: US exceptionalism? International trends in midlife mortality
Source: Int J Epidemiol. 2024 Mar 21;53(2):dyae024. doi: 10.1093/ije/dyae024 (PMC10954513; doi:10.1093/ije/dyae024)

Supplementary Material

Table of Contents

List of Abbreviations and Definitions…………………………………………………………………………2
Table S1 ICD9-ICD10 harmonization 3
Table S2 Availability of countries-years in mortality data 4
Table S3 Availability of countries-years in population data...5
Table S4 Countries-years with 4-digit ICD10 codes unavailable 7
Figure S1 Age−Standardized Mortality from Infectious and Parasitic Diseases, Years 1990−2019 8
Figure S2 Age−Standardized Mortality from HIV/AIDS, Years 1990−2019 9
Figure S3 Age−Standardized Mortality from Respiratory Diseases, Years 1990−2019 10
Figure S4 Age−Standardized Mortality from Trachea/Bronchus, Lung Cancers, Years 1990−2019 11
Figure S5 Age−Standardized Mortality from All Other Cancers, Years 1990−2019..........................12
Figure S6 Age−Standardized Mortality from Nervous System Diseases, Years 1990−2019...................13
Figure S7 Age−Standardized Mortality from Metabolic Diseases, Years 1990−2019...........................14
Figure S8 Age−Standardized Mortality from Cardiovascular Disease, Years 1990−2019 15
Figure S9 Age−Standardized Mortality from Suicide, Years 1990−2019..16
Figure S10 Age−Standardized Mortality from Homicide, Years 1990−2019 17
Figure S11 Age−Standardized Mortality from Transport Accidents, Years 1990−2019 18
Figure S12 Age−Standardized Mortality from Other External Causes, Years 1990−2019....................19
Figure S13 Age−Standardized Mortality from All Other Causes, Years 1990−2019 20
Figure S14 Age−Standardized Mortality from All Causes, Years 1990−2019.....................................21
Figure S15 Age−Standardized Mortality from Drug-Related Causes, Years 2000−2019.......................22
Figure S16 Age−Standardized Mortality from Alcohol-Related Causes, Years 2000−2019 23
Figure S17 Percent Change in Age−Standardized Mortality from the Baseline Year (1990), Infectious and Parasitic Diseases 24
Figure S18 Percent Change in Age−Standardized Mortality from the Baseline Year (1990), HIV/AIDS............25
Figure S19 Percent Change in Age−Standardized Mortality from the Baseline Year (1990), Respiratory Diseases..26
Figure S20 Percent Change in Age−Standardized Mortality from the Baseline Year (1990), Trachea/Bronchus, Lung Cancers 27
Figure S21 Percent Change in Age−Standardized Mortality from the Baseline Year (1990), All Other Cancers 28
Figure S22 Percent Change in Age−Standardized Mortality from the Baseline Year (1990), Nervous System 29
Figure S23 Percent Change in Age−Standardized Mortality from the Baseline Year (1990), Metabolic Diseases……………………………………………………………………………………30
Figure S24 Percent Change in Age−Standardized Mortality from the Baseline Year (1990), Cardiovascular Disease 31

Figure S25 Percent Change in Age−Standardized Mortality from the Baseline Year (2000), Suicide 32
Figure S26 Percent Change in Age−Standardized Mortality from the Baseline Year (2000), Homicide 33
Figure S27 Percent Change in Age−Standardized Mortality from the Baseline Year (1990), Transport Accidents 34
Figure S28 Percent Change in Age−Standardized Mortality from the Baseline Year (1990), Other External Causes Diseases 35
Figure S29 Percent Change in Age−Standardized Mortality from the Baseline Year (1990), All Other Causes 36
Figure S30 Percent Change in Age−Standardized Mortality from the Baseline Year (1990), All Causes............37
Figure S31 Percent Change in Age−Standardized Mortality from the Baseline Year (2000), Drug-Related Causes 38
Figure S32 Percent Change in Age−Standardized Mortality from the Baseline Year (2000), Alcohol-Related Causes 39
Figure S33 Percent Change in All−Cause Mortality Between 1990 and 2019/Males, Ages 25−44......................40
Figure S34 Percent Change in All−Cause Mortality Between 1990 and 2019/Males, Ages 45−54......................41
Figure S35 Percent Change in All−Cause Mortality Between 1990 and 2019/Males, Ages 55−64......................42
Figure S36 Percent Change in All−Cause Mortality Between 1990 and 2019/Females, Ages 25−44..................43
Figure S37 Percent Change in All−Cause Mortality Between 1990 and 2019/Females, Ages 45−54..................44
Figure S38 Percent Change in All−Cause Mortality Between 1990 and 2019/Females, Ages 55−64..................45
Figures S39-S134 Three-year moving average mortality figures by country, cause, sex and age 46-141

List of Abbreviations and Definitions

Abbreviations

AIDS Acquired Immune Deficiency Syndrome
CEE Central and Eastern European country
HIV Human Immunodeficiency Virus
ICD9 BTL International Classification of Diseases, Ninth Revision, Basic Tabulation List
ICD10 International Classification of Diseases, Tenth Revision
UK United Kingdom
US United States

Definitions

Peer A high-income country comparable to the USA.

Table S1. ICD9 BTL - ICD10 harmonization.

Categories	ICD9 BTLcodes	ICD10 codes	Notes
Infectious and parasitic (excluding HIV/AIDS)	B01 - B07	A00 - A99, B00 - B19, B25 -B99	
HIV/AIDS	B184, B185	B20 - B24	
Respiratory system	B31 - B32	J00 - J98	
All cancers (excluding trachea/bronchus, lung and liver cancers)	B08 - B09, B10, B100, B109, B11 - B17	C00 - D48, excluding C33, C34	
Trachea/ bronchus, lung cancers	B101	C33 -- C34	
Nervous system	B22	G00 - G98	
Endocrine, Nutritional & Metabolic	B180-B183, B189, B19	E00 - E88	
Circulatory system	B25 – B30	I00 - I99	
Suicide	B54	X60–X84, Y87.0	
Drug poisoning	N/A in ICD9 BTL coding scheme	F11-F16, F18-F19, X40–X44, X85, Y10–Y14	Due to ICD9 BTL scheme limitations this category is only kept for country-years where ICD10 scheme is available. For country-years with ICD9 BTL scheme drug-related deaths are set to 'other external causes' category.
Alcohol-induced	N/A in ICD9 BTL coding scheme	K70, K73-74, F10, X45, Y15	Due to ICD9 BTL scheme limitations this category is only kept for country-years where ICD10 scheme is available. For country-years with ICD9 BTL scheme drug-related deaths are set to 'other external causes' category.
Homicide	B55	X86 - X99, Y00 - Y09, Y87.1	
Transport accidents	B47	V01–V99, Y85	
Other external causes	B480-B482, B49, B50, B51, B52, B53, B56	W00–W99, X00–X39, X46–X59, Y16–Y36, Y40–Y84, Y86, Y87.2, Y88, Y89	

ICD9 BTL, International Classification of Diseases, Ninth Revision, Basic Tabulation List; ICD 10, International Classification of Diseases, Tenth Revision; HIV, Human Immunodeficiency Virus; AIDS, Acquired Immune Deficiency Syndrome.

Table S2. Availability of countries-years in mortality data as of 1st June 2023.
Source: https://www.who.int/data/data-collection-tools/who-mortality-database

Country Code	Name	Year	List	Icd	Note
2090	Canada	1979-1999<br>2000-2019	09B<br>104	Icd9<br>Icd10	
2450	United States of America	1979-1998<br>1999-2019	09B<br>104	Icd9<br>Icd10	
3160	Japan	1979-1994<br>1995-2017<br>2018-2019	09B<br>104<br>103	Icd9<br>Icd10<br>Icd10	
4010	Austria	1980-2001<br>2002-2019	09B<br>104	Icd9<br>Icd10	
4020	Belgium	1979-1997<br>1998-1999<br>2000-2018	09B<br>103<br>104	Icd9<br>Icd10<br>Icd10	
4050	Denmark	1994-2019	104	Icd10	
4070	Finland	1987-1994<br>1996-2019	09B<br>103	Icd9<br>Icd10	
4080	France	1979-1999<br>2000-2017	09B<br>104	Icd9<br>Icd10	
4085	Germany	1990-1997<br>1998-2019	09B<br>104	Icd9<br>Icd10	
4180	Italy	1979-2002<br>2003-2019	09B<br>104	Icd9<br>Icd10	
4210	Netherlands	1979-1995<br>1996-1999<br>2000-2019	09B<br>10M<br>104	Icd9<br>Icd10<br>Icd10	
4220	Norway	1986-1995<br>1996-2016	09B<br>104	Icd9<br>Icd10	
4240	Portugal	1980-2001<br>2002-2003<br>2004-2005<br>2007-2019	09B<br>104<br>UE1<br>104	Icd9<br>Icd10<br>Icd10<br>Icd10	Mortality data for 2004-2005 are not disaggregated by age; mortality data are not available for 2006
4280	Spain	1980-1998<br>1999-2019	09B<br>104	Icd9<br>Icd10	
4290	Sweden	1987-1996<br>1997-1998<br>1999-2018	09B<br>10M<br>104	Icd9<br>Icd10<br>Icd10	
4300	Switzerland	1995-2019	104	Icd10	
4308	United Kingdom	1979-1999<br>2001-2019	09B<br>104	Icd9<br>Icd10	Mortality data are not available for 2000
5020	Australia	1979-1997<br>1998-2019	09B<br>104	Icd9<br>Icd10	Mortality data are not

					available for 2005
4030	Bulgaria	1980-2004<br>2005-2019	09B<br>104	Icd9<br>Icd10	
4045	Czech Republic	1986-1993<br>1994-2019	09B<br>104	Icd9<br>Icd10	
4150	Hungary	1979-1995<br>1996-2019	09B<br>104	Icd9<br>Icd10	
4230	Poland	1980-1996<br>1999-2019	09B<br>104	Icd9<br>Icd10	No data for 1997-1998
4270	Romania	1980-1998<br>1999-2019	09B<br>104	Icd9<br>Icd10	
4274	Slovakia	1992-1993<br>1994-2009<br>2010-2019	09B<br>103<br>104	Icd9<br>Icd10<br>Icd10	No data for 1990, 2011, 2015
4276	Slovenia	1985-1996<br>1997-2019	09B<br>103	Icd9<br>Icd10	

Icd9, International Classification of Diseases, Ninth Revision; Icd10, International Classification of Diseases, Tenth Revision.

Table S3. Availability of countries-years (1990-2019) on population data as of 1st June 2023.
Source: https://www.who.int/data/data-collection-tools/who-mortality-database

Country Code	Name	Year	Note	Link
2090	Canada	1990-2005	Data for 2006-2019 are unavailable; extract from Statistics Canada instead	https://www150.statcan.gc.ca/t1/tbl1/en/cv.action?pid=1710000501
2450	United States of America	1990-2007	Data for 2008-2019 are unavailable; extract from US Census Bureau instead	https://www.census.gov/programs-surveys/popest/technical-documentation/research/evaluation-estimates/2020-evaluation-estimates/2010s-national-detail.html
3160	Japan	1990-2019		
4010	Austria	1990-2019		
4020	Belgium	1990-2019		
4050	Denmark	1990-2019		
4070	Finland	1990-2019		
4080	France	1990-2014,2017	Data for 2015-2016 are unavailable; extract from France National Institute of Statistics and Economic Studies instead	https://www.insee.fr/en/statistiques/pyramide/3312960/xls/pyramides-des-ages_bilan-demo_2019.xls

4085	Germany	1990-2019		
4180	Italy	1990-2019		
4210	Netherlands	1990-2019		
4220	Norway	1990-2019		
4240	Portugal	1990-2018		
4280	Spain	1990-2019		
4290	Sweden	1990-2018		
4300	Switzerland	1990-2019		
4308	United Kingdom	1990-2019		
5020	Australia	1990-2019		
4030	Bulgaria	1990-2019		
4045	Czech Republic	1990-2019		
4150	Hungary	1990-2019		
4230	Poland	1990-2019		
4270	Romania	1990-2019		
4274	Slovakia	1990-2019	Year 2015 missing	
4276	Slovenia	1990-2019		

Table S4. Countries-years for which ICD10 4-digit codes are unavailable.

Country	Year
Japan	2018
Bulgaria	2008
Bulgaria	2009
Bulgaria	2010
Bulgaria	2011
Bulgaria	2012
Finland	2008
Finland	2009
Finland	2010
Finland	2011
Finland	2012
Finland	2013
Finland	2014
Finland	2015
Finland	2016
Finland	2017
Finland	2018
Slovakia	2008
Slovakia	2009
Slovenia	2008
Slovenia	2009
Slovenia	2010
Slovenia	2011
Slovenia	2012
Slovenia	2013
Slovenia	2014
Slovenia	2015
Slovenia	2016
Slovenia	2017
Slovenia	2018
Slovenia	2019
Slovenia	2020

ICD 10, International Classification of Diseases, Tenth Revision

Figure S1. Age-Standardized Mortality from Infectious and Parasitic Diseases, Years 1990-2019

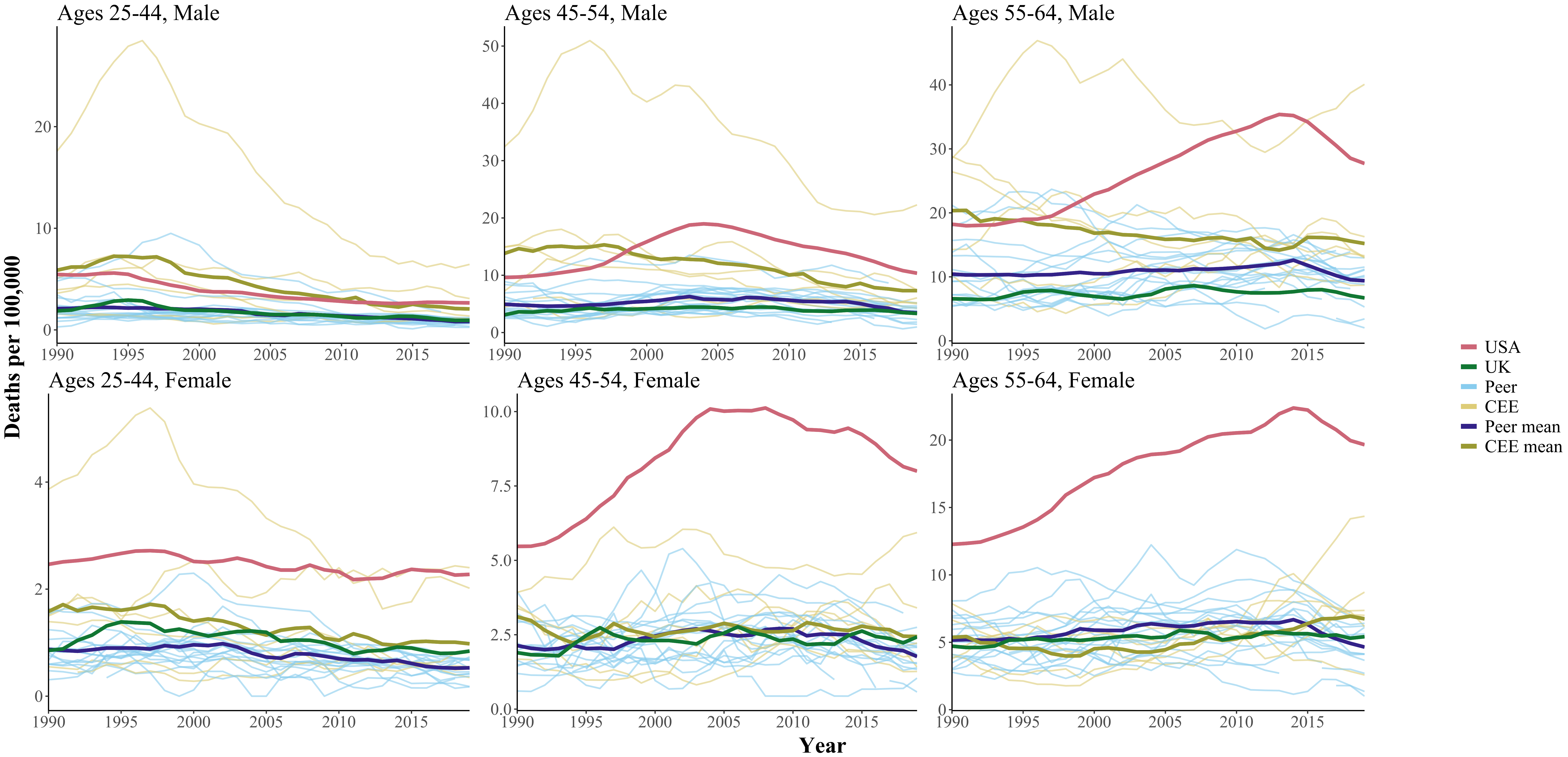

Figure S2. Age-Standardized Mortality from HIV/AIDS, Years 1990-2019

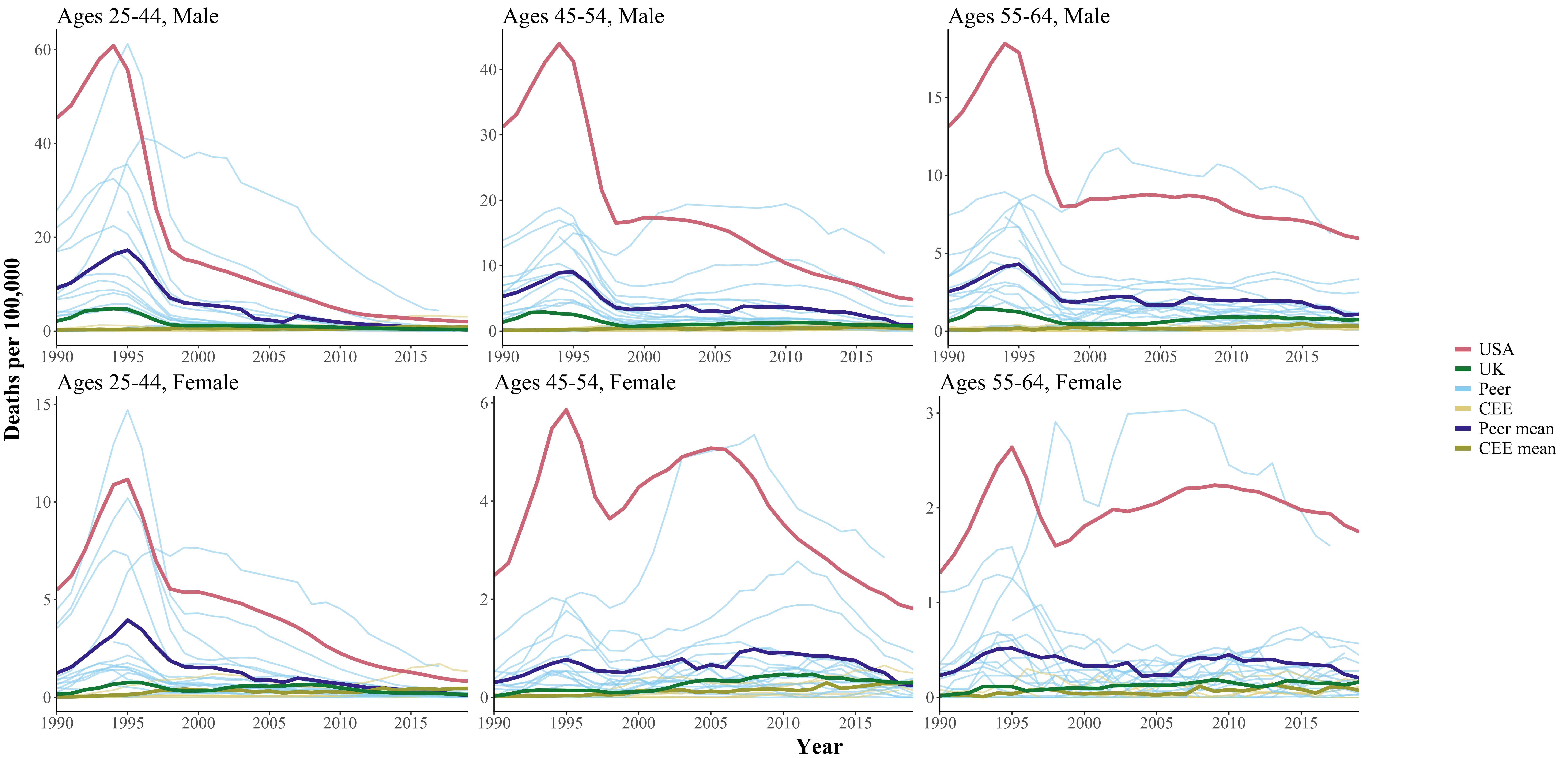

Figure S3. Age-Standardized Mortality from Respiratory Diseases, Years 1990-2019

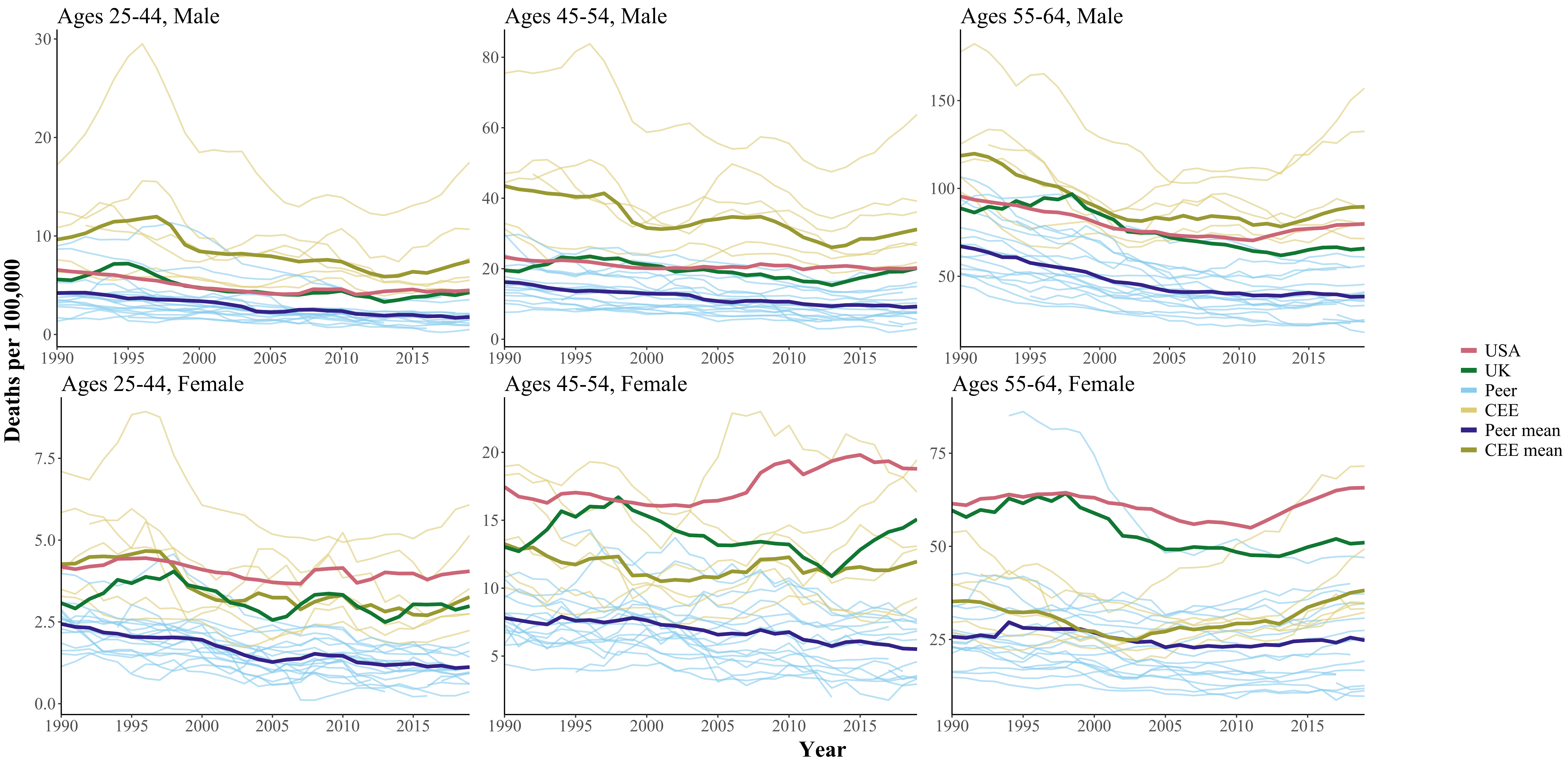

Figure S4. Age-Standardized Mortality from Trachea/Bronchus, Lung Cancers, Years 1990-2019

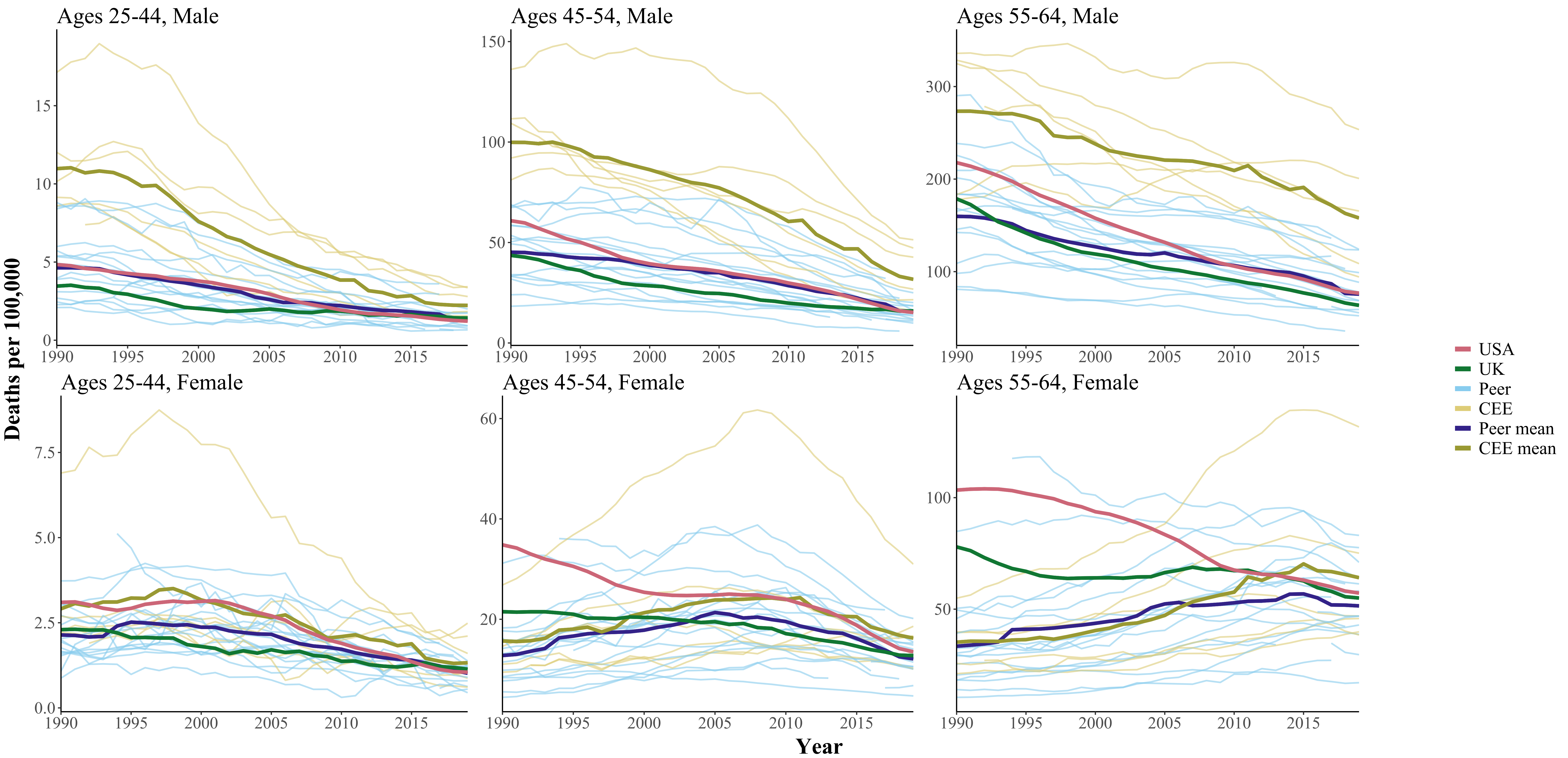

Figure S5. Age-Standardized Mortality from All Other Cancers, Years 1990-2019

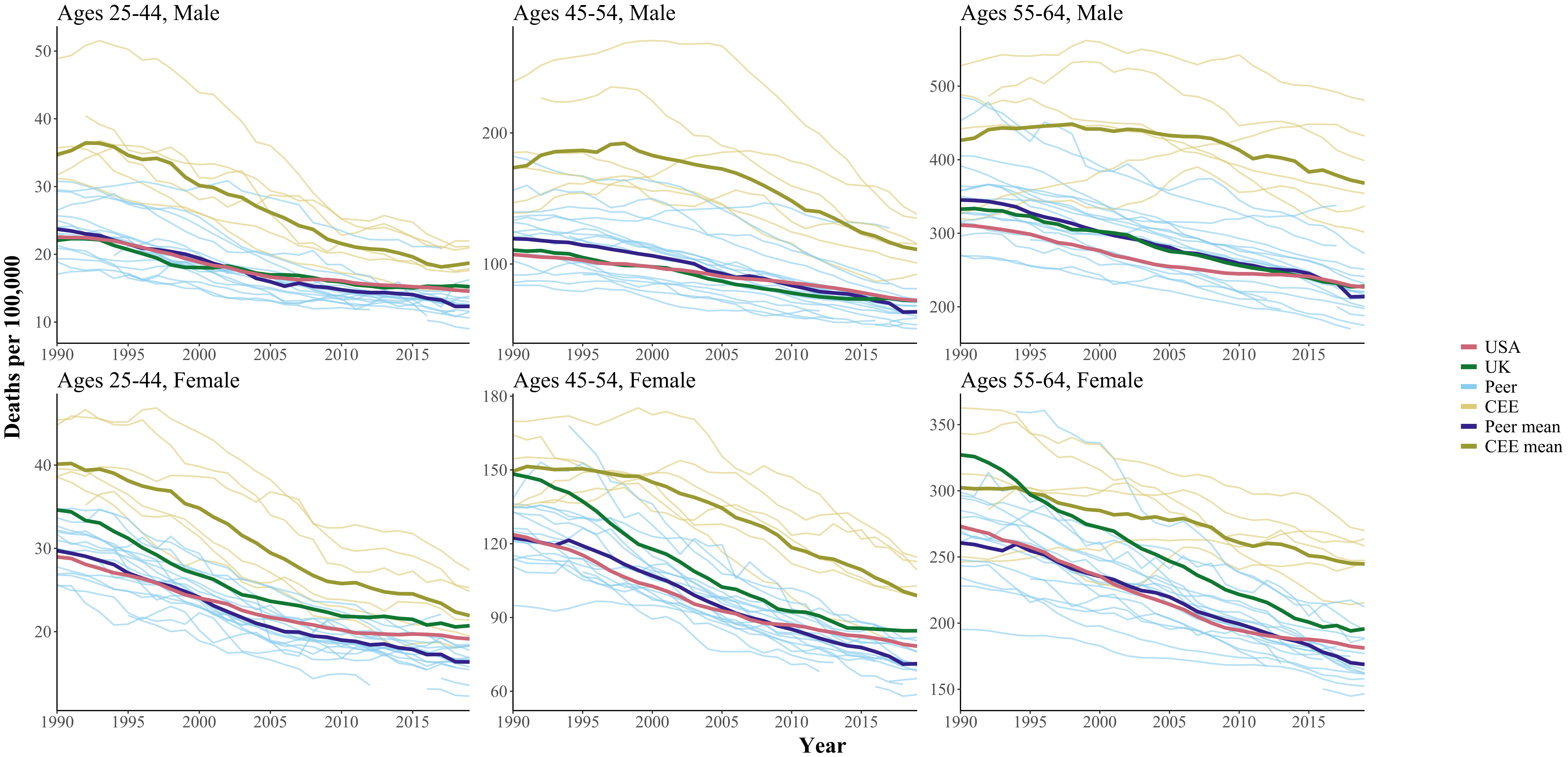

Figure S6. Age-Standardized Mortality from Nervous System Diseases, Years 1990-2019

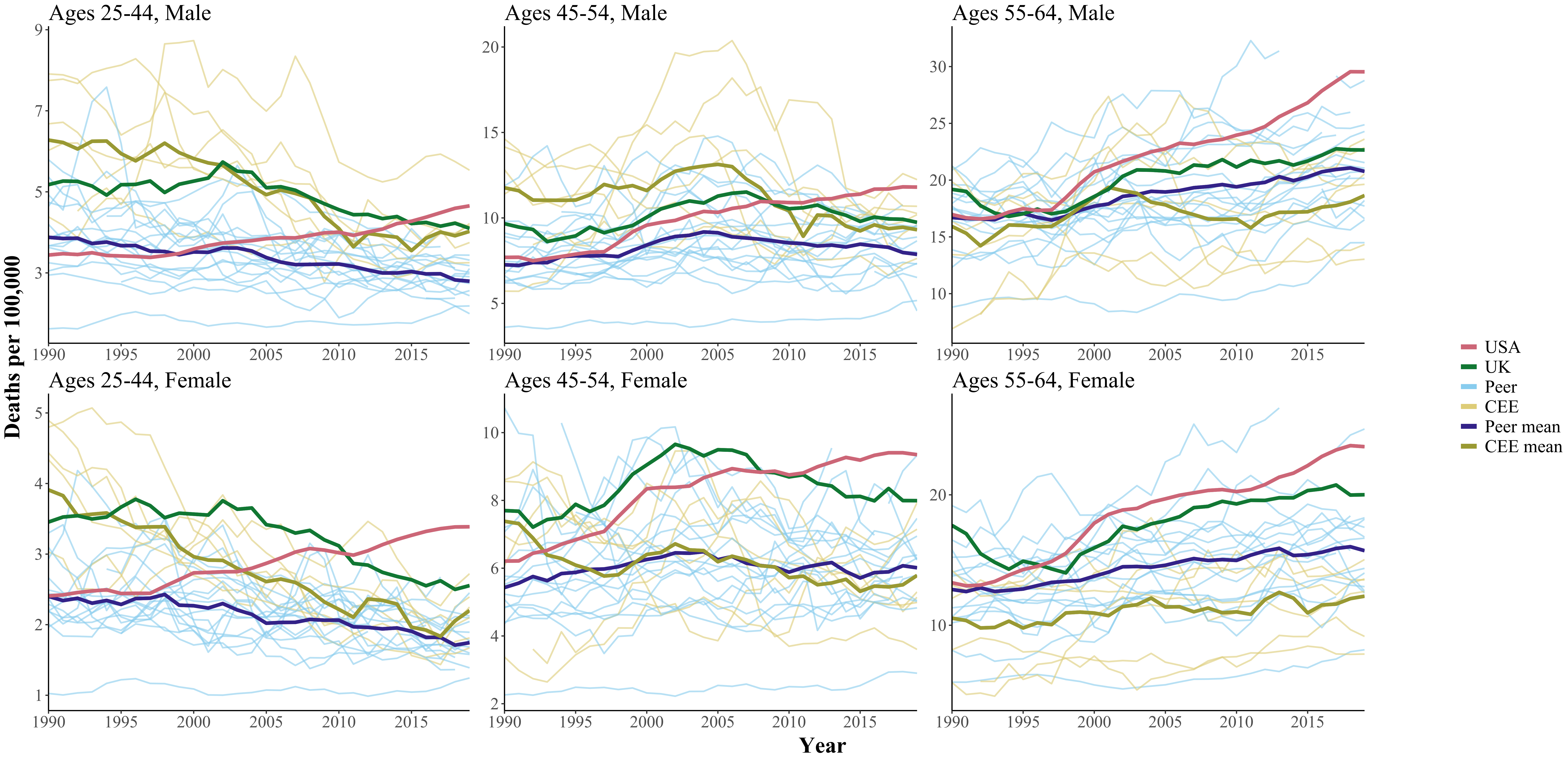

Figure S7. Age-Standardized Mortality from Metabolic Diseases, Years 1990-2019

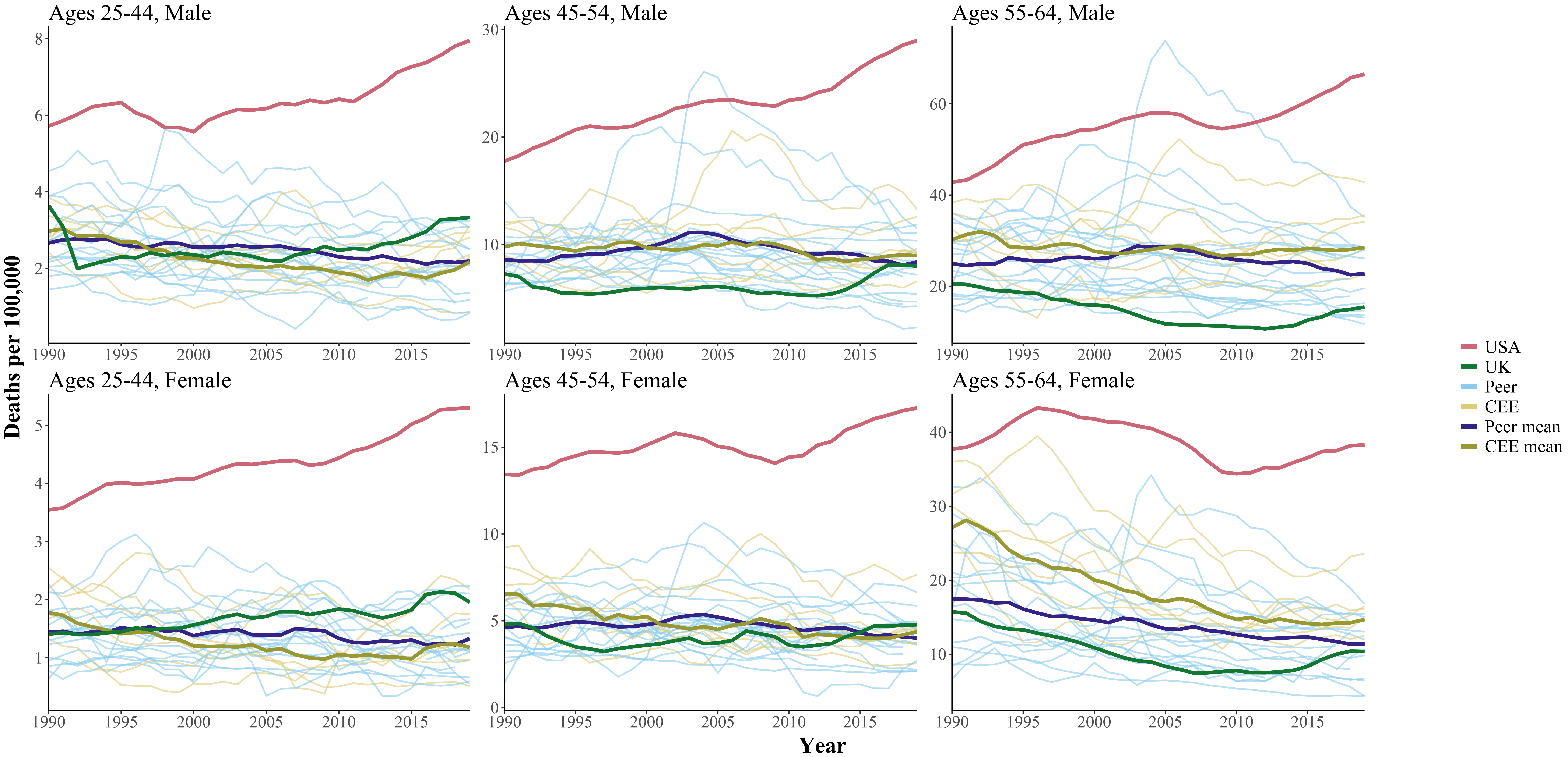

Figure S8. Age-Standardized Mortality from Cardiovascular Disease, Years 1990-2019

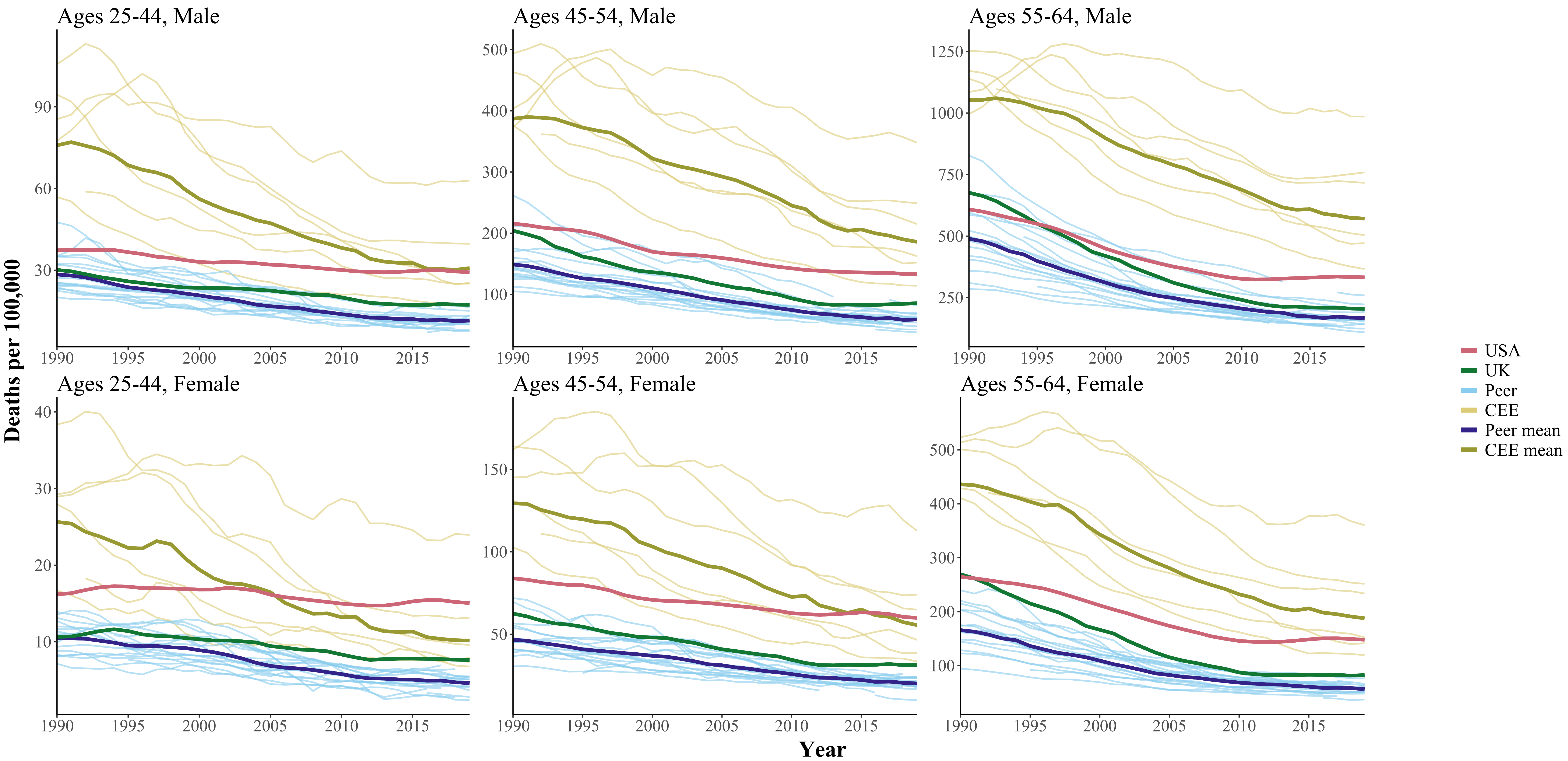

Figure S9. Age-Standardized Mortality from Suicide, Years 1990-2019

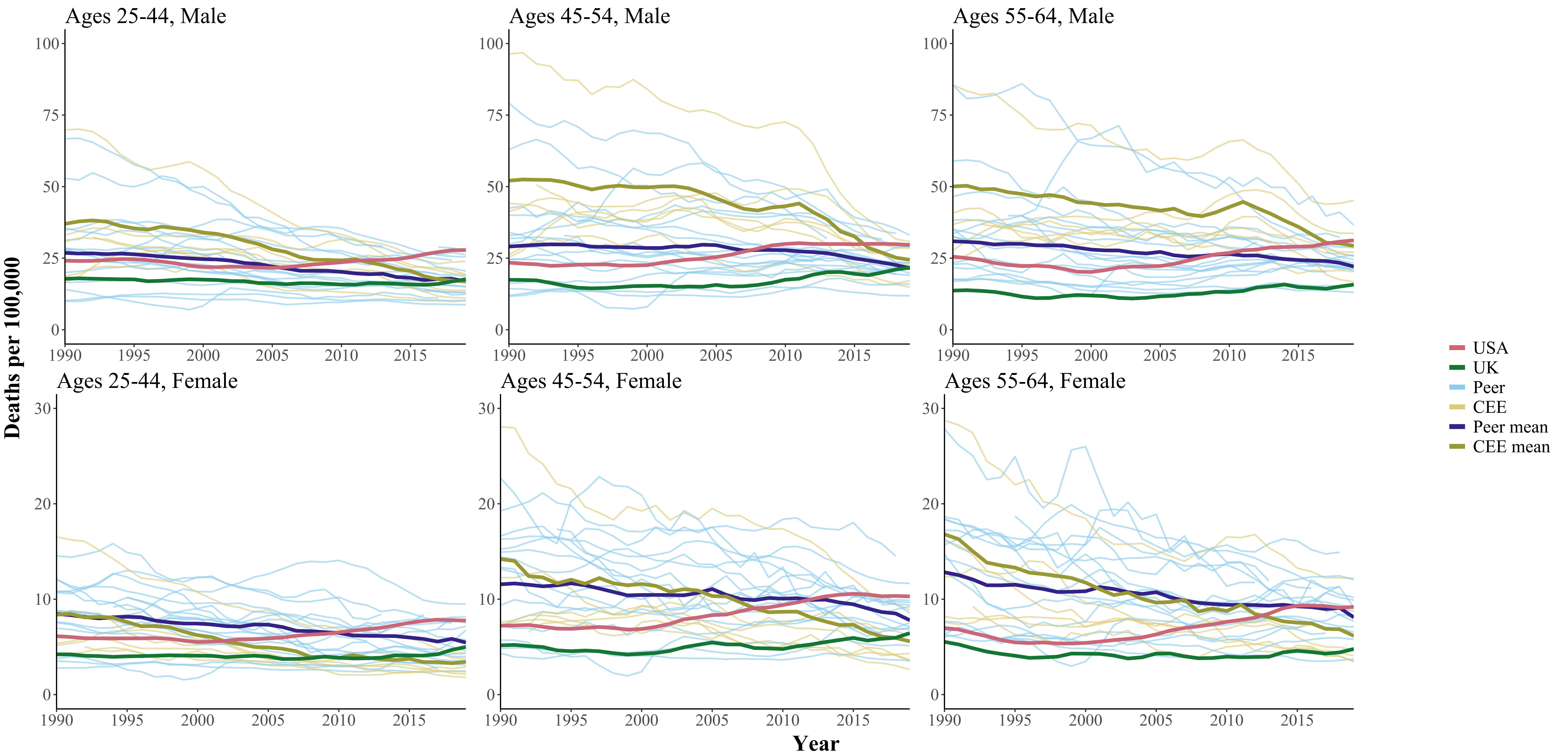

Figure S10. Age-Standardized Mortality from Homicide, Years 1990-2019

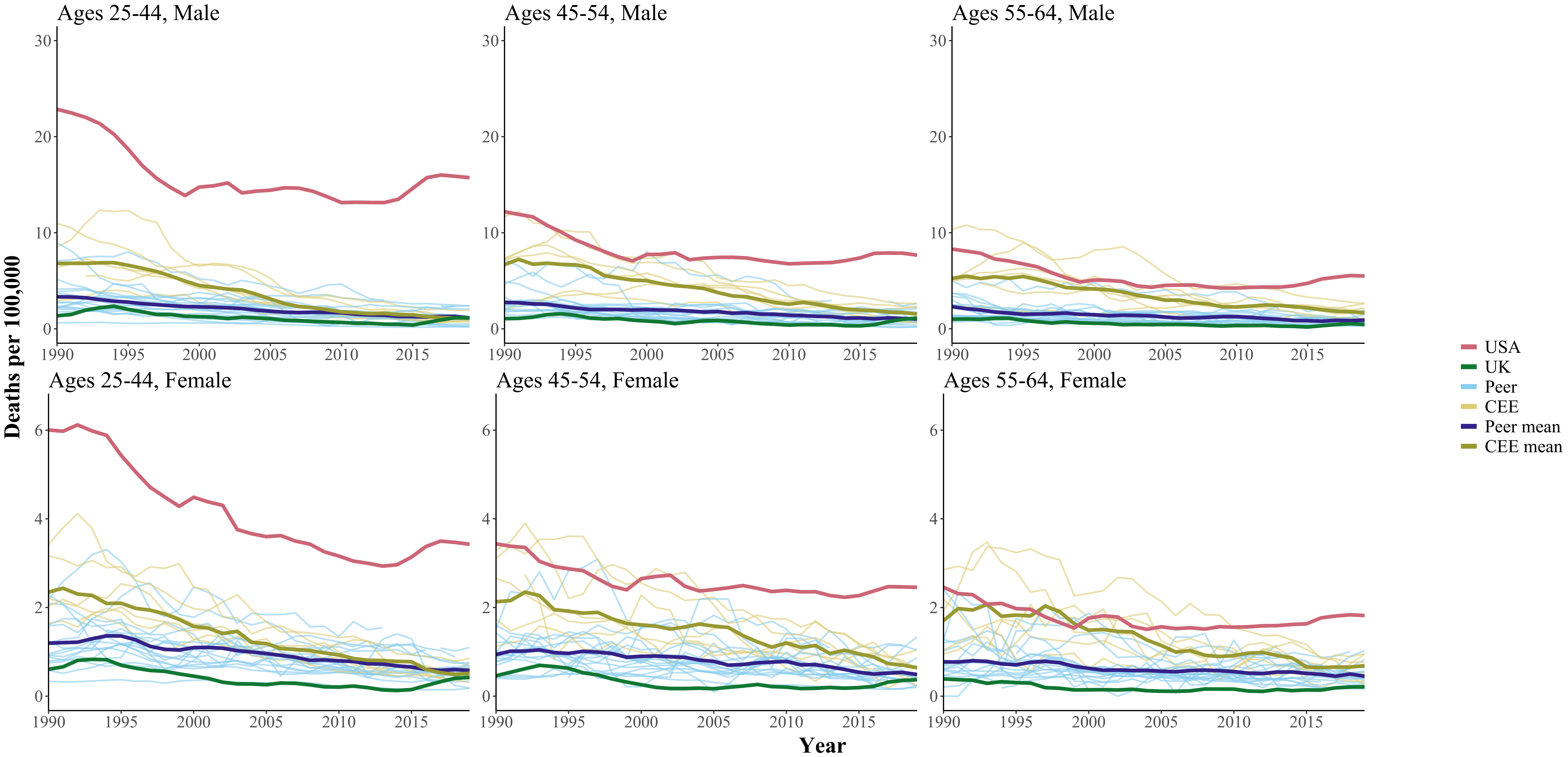

Figure S11. Age-Standardized Mortality from Transport Accidents, Years 1990-2019

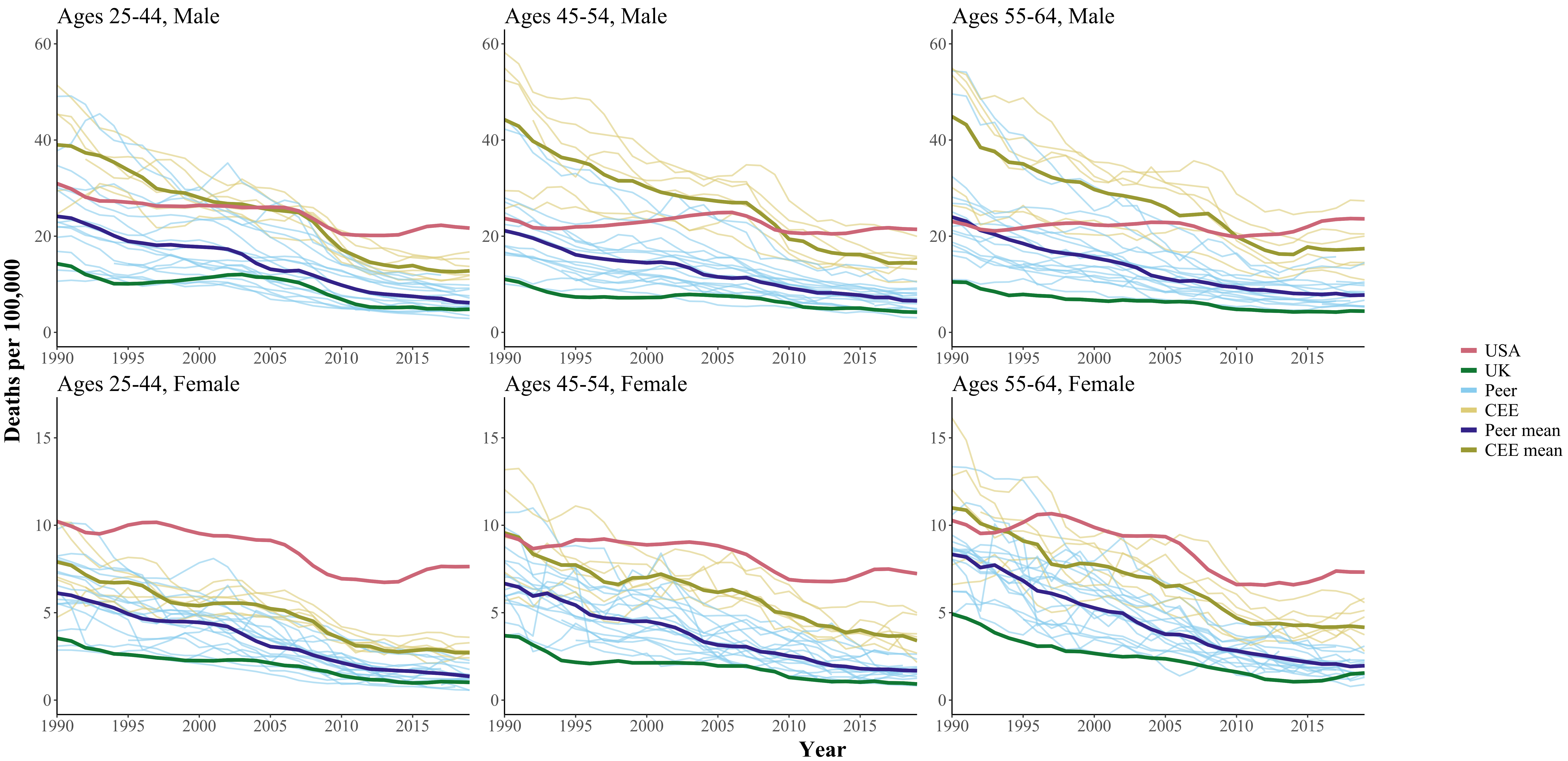

Figure S12. Age-Standardized Mortality from Other External Causes, Years 1990-2019

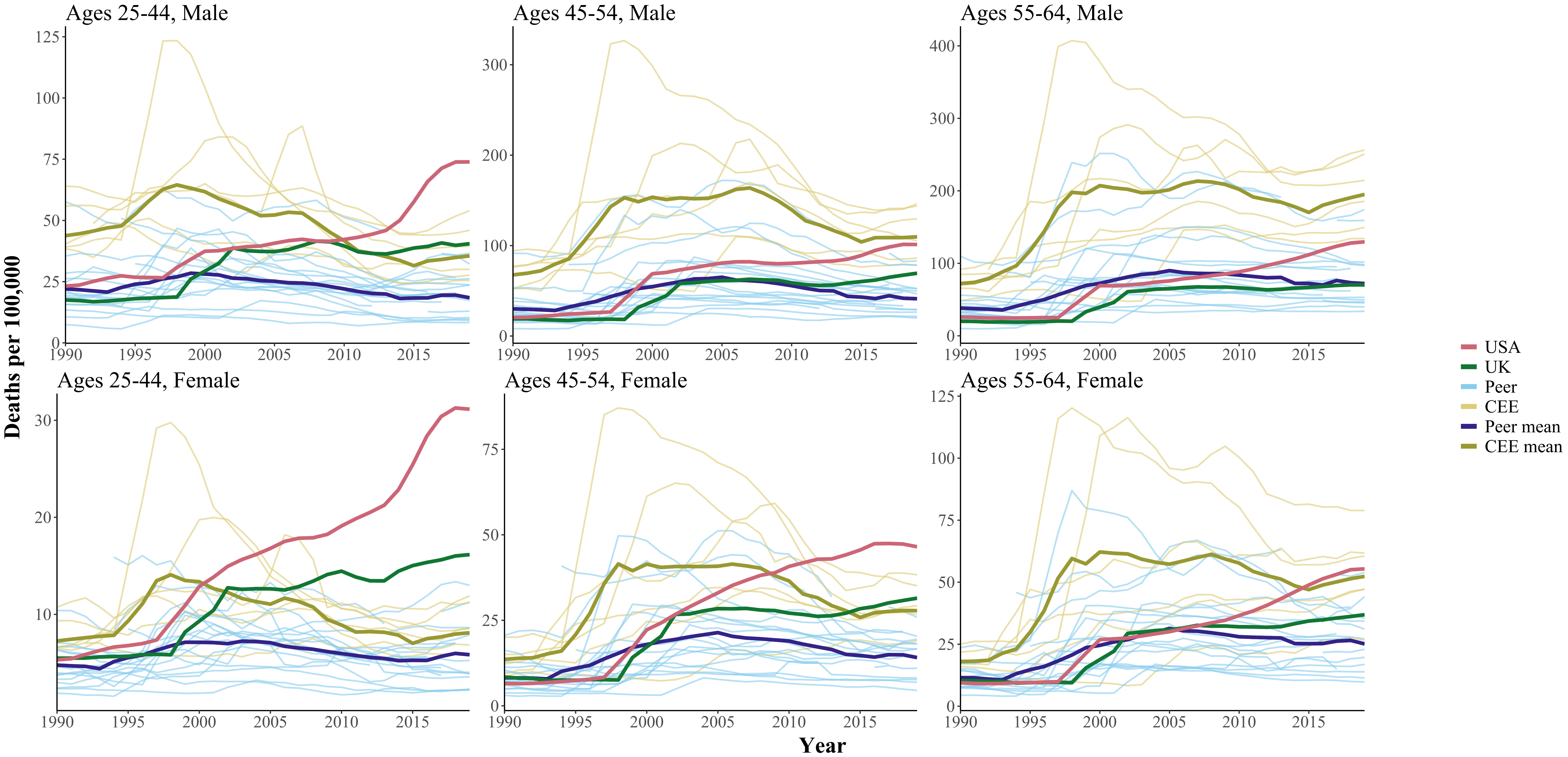

Figure S13. Age-Standardized Mortality from All Other Causes, Years 1990-2019

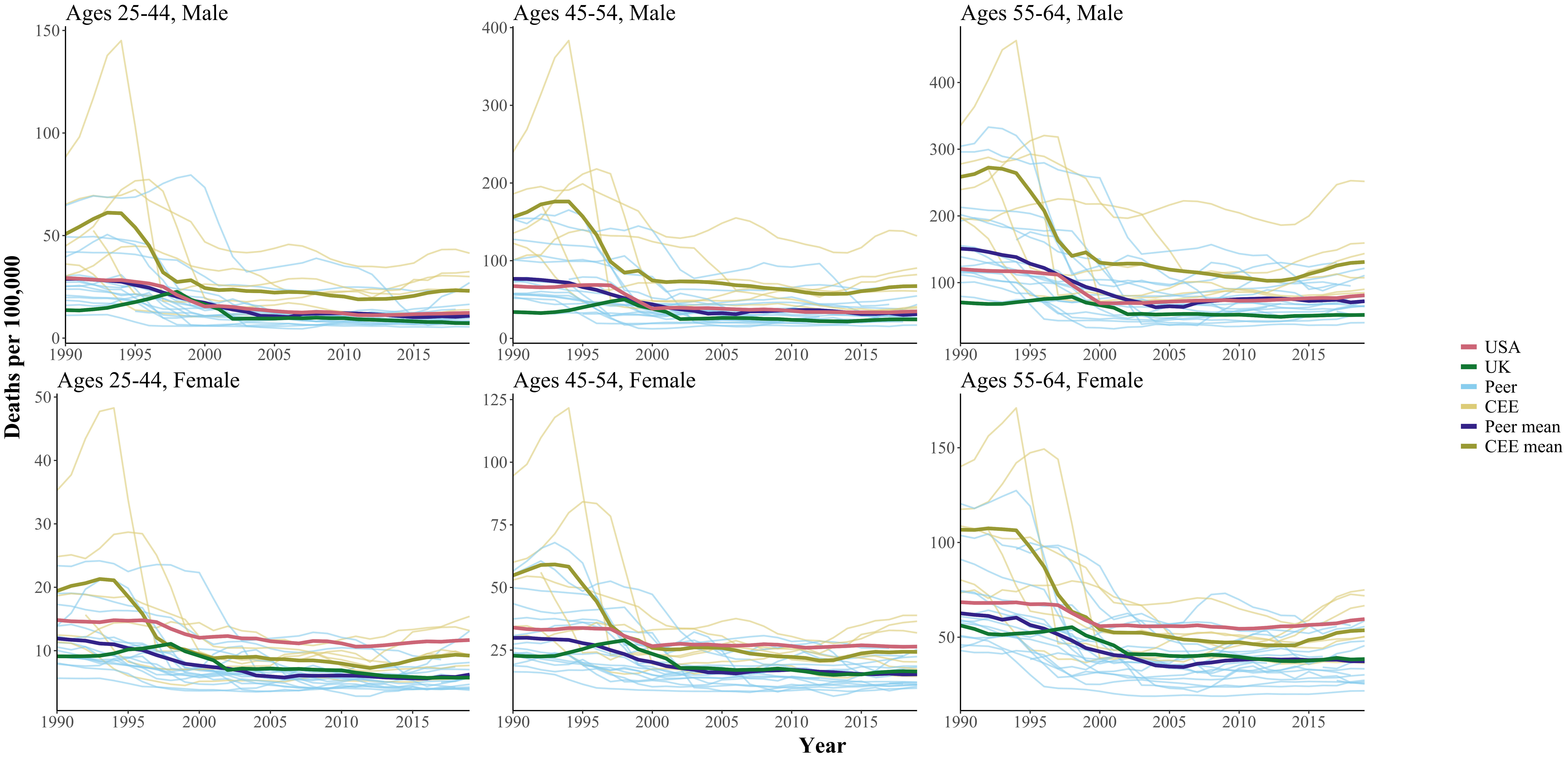

Figure S14. Age-Standardized Mortality from All Causes, Years 1990-2019

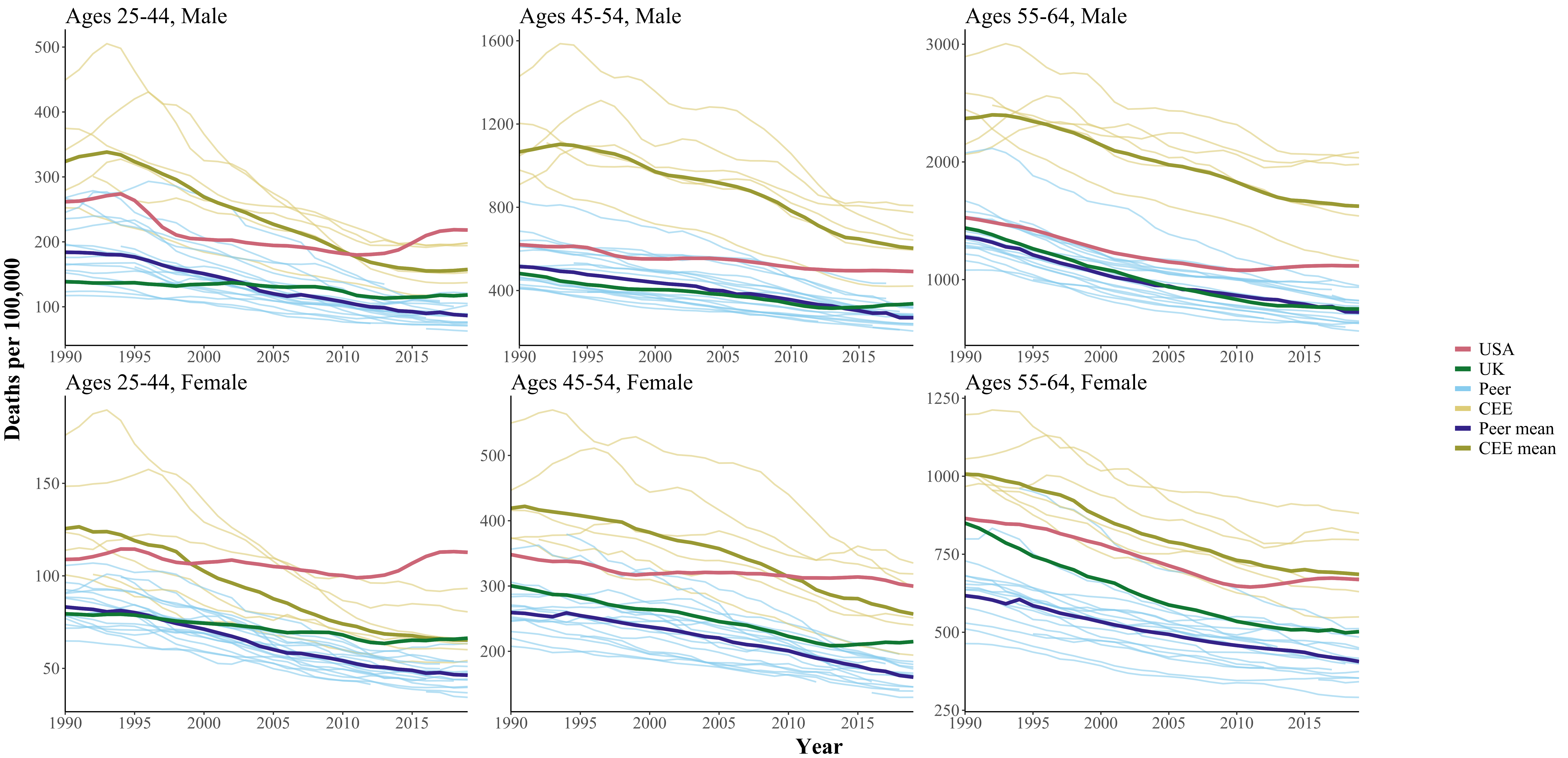

Figure S15. Age-Standardized Mortality from Drug-Related Causes, Years 2000-2019

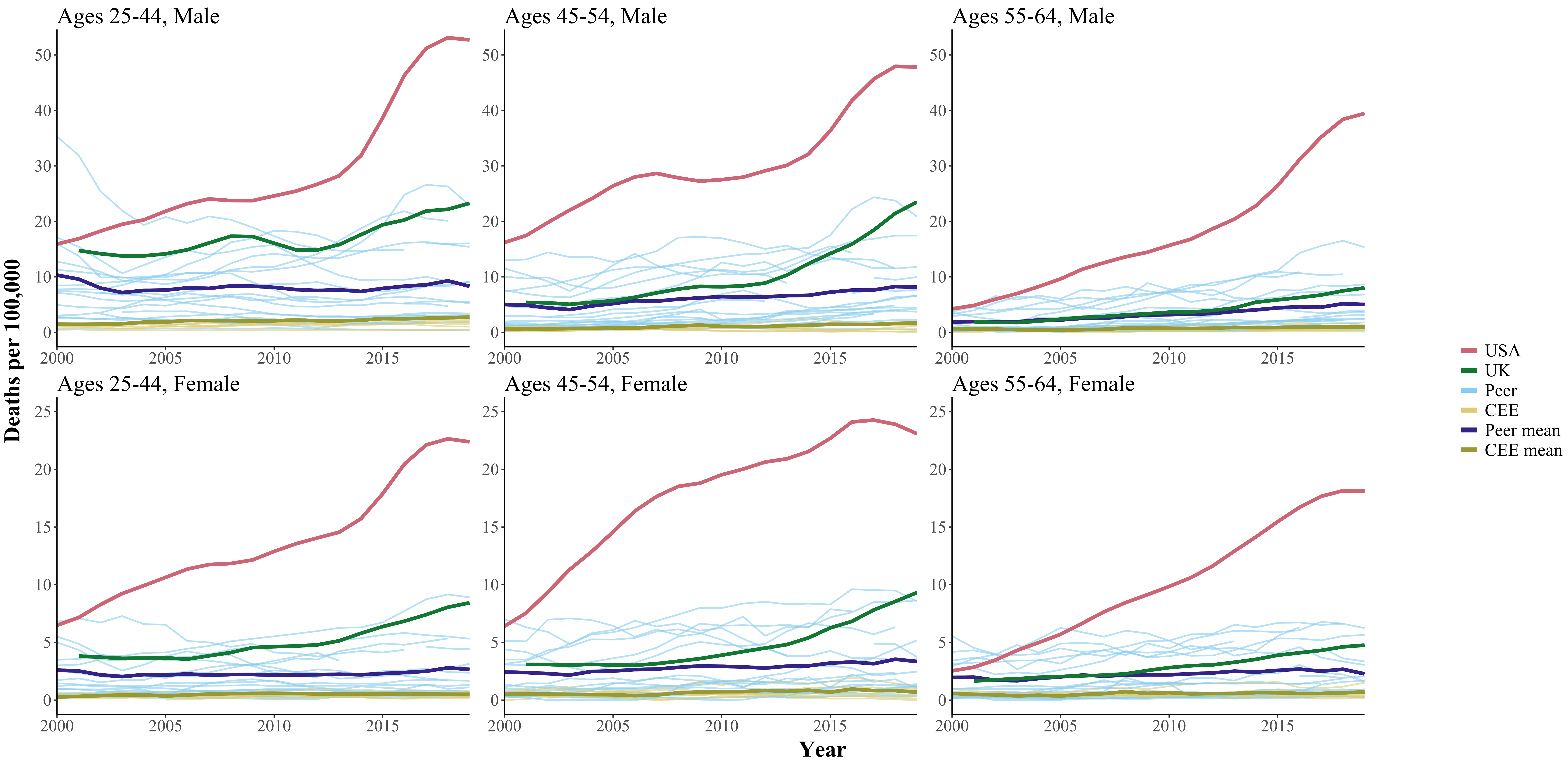

Figure S16. Age-Standardized Mortality from Alcohol-Related Causes, Years 2000-2019

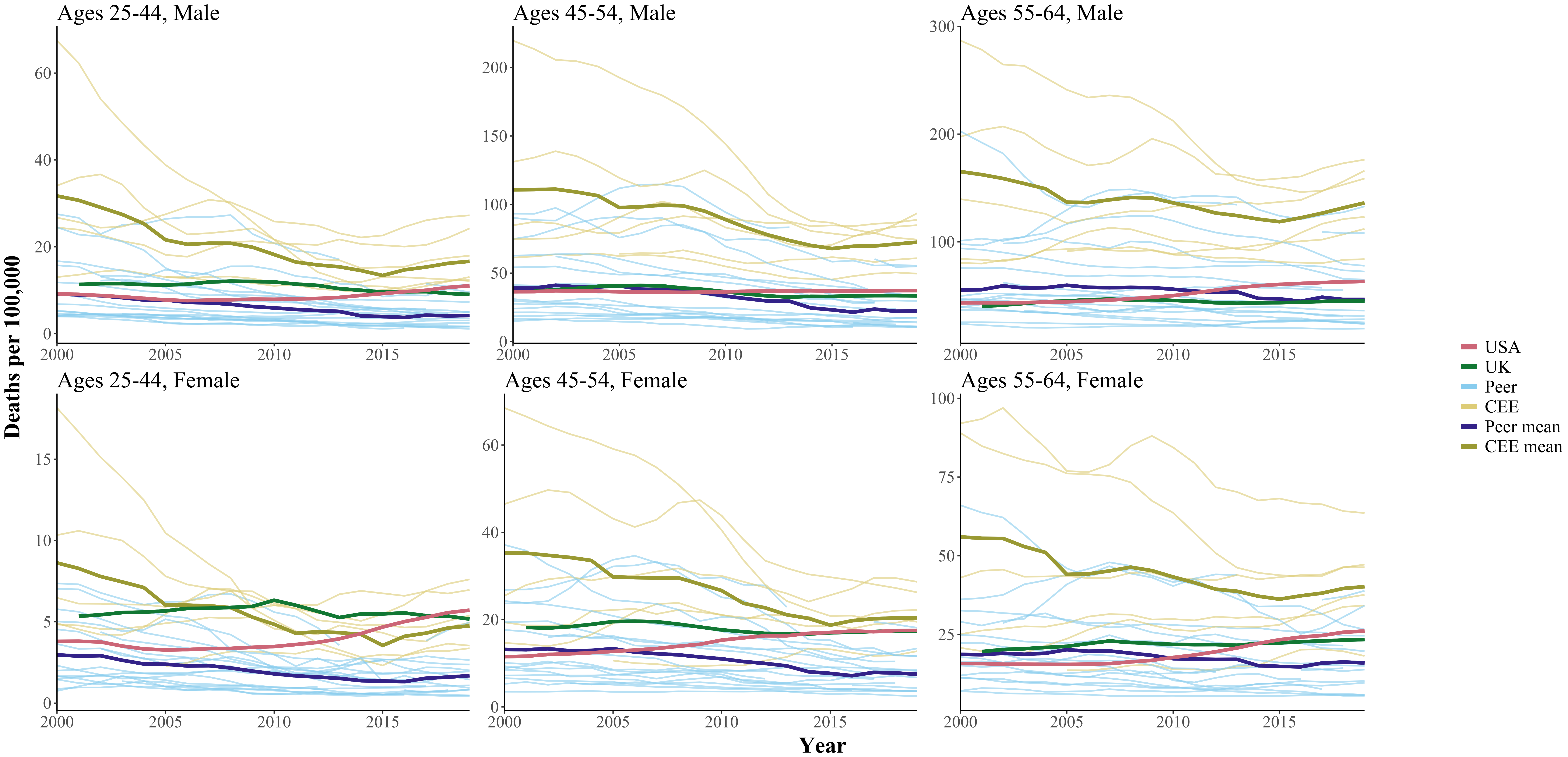

Figure S17. Percent Change in Age-Standardized Mortality from the Baseline Year (1990), Infectious and Parasitic Diseases

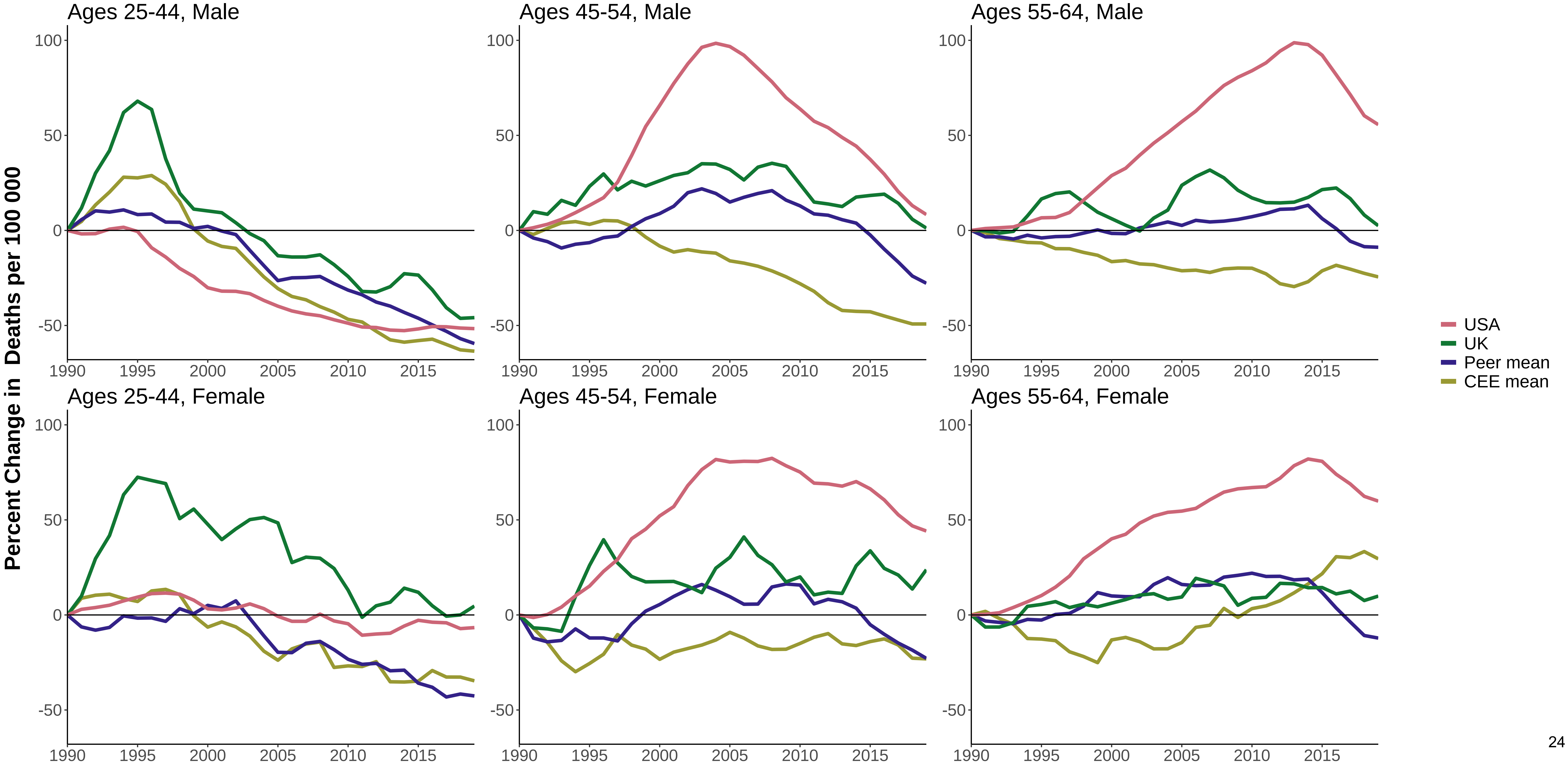

Figure S18. Percent Change in Age-Standardized Mortality from the Baseline Year (1990), HIV/AIDS

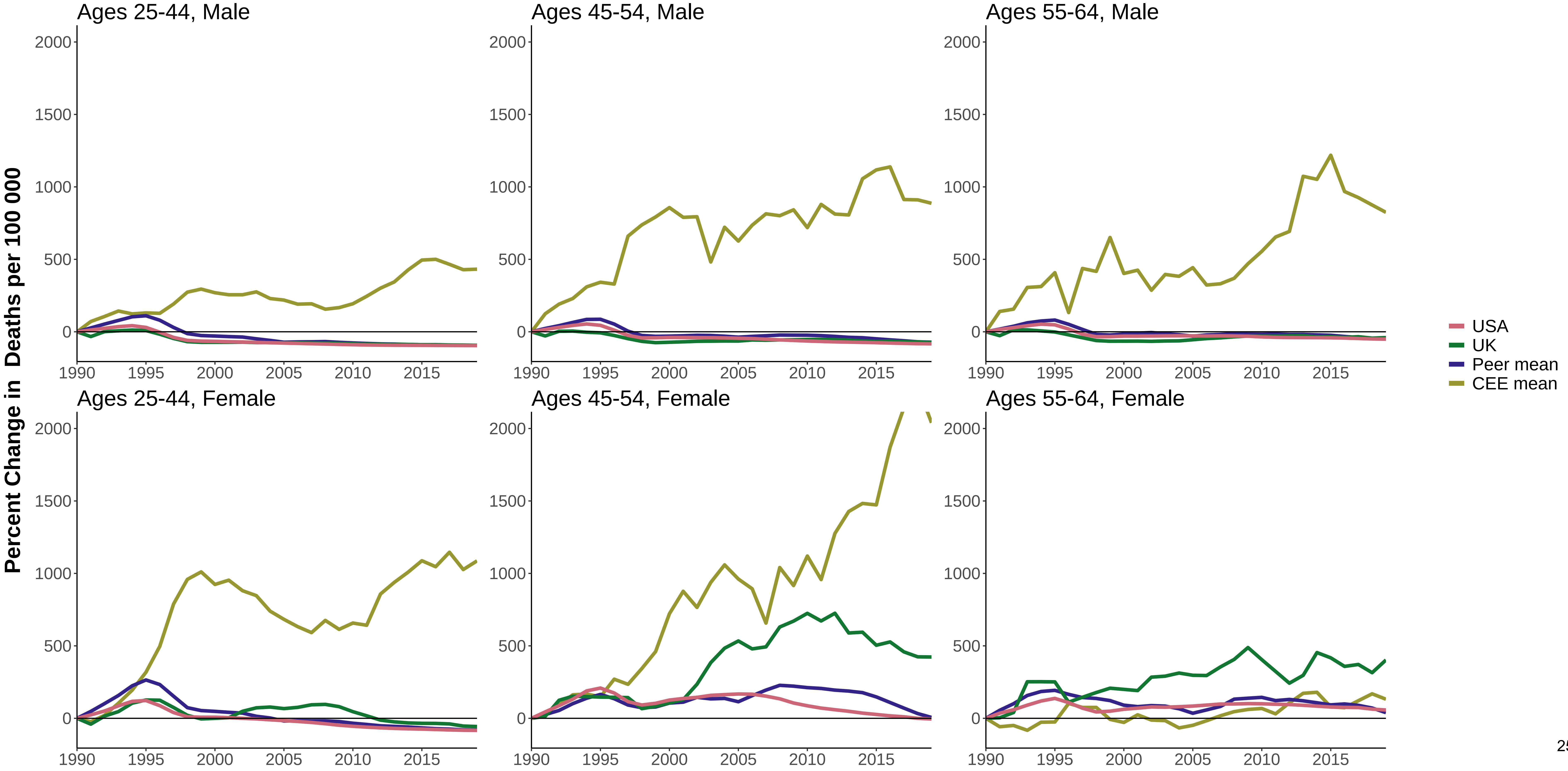

Figure S19. Percent Change in Age-Standardized Mortality from the Baseline Year (1990), Respiratory Diseases

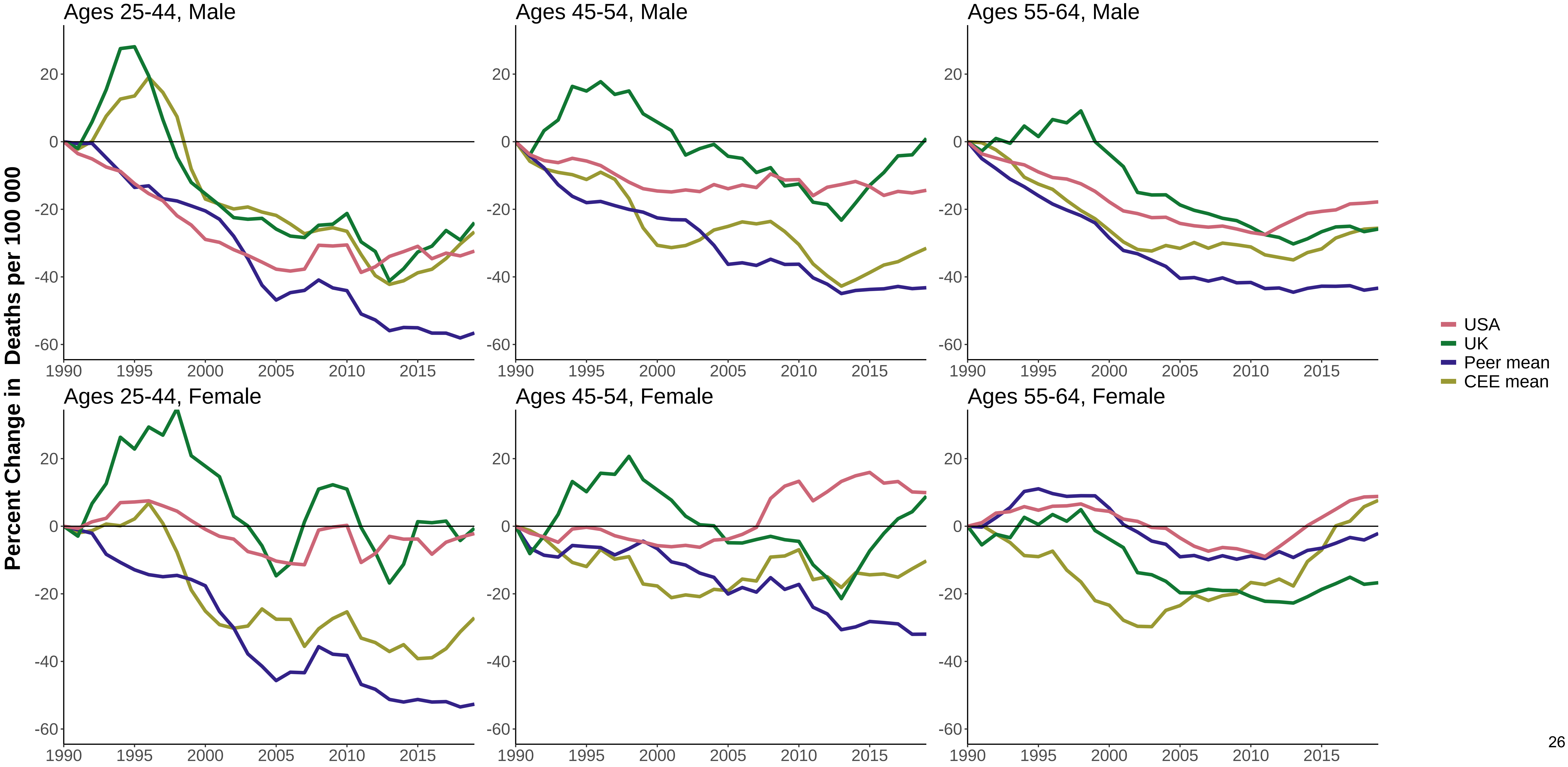

Figure S20. Percent Change in Age-Standardized Mortality from the Baseline Year (1990), Trachea/Bronchus, Lung Cancers

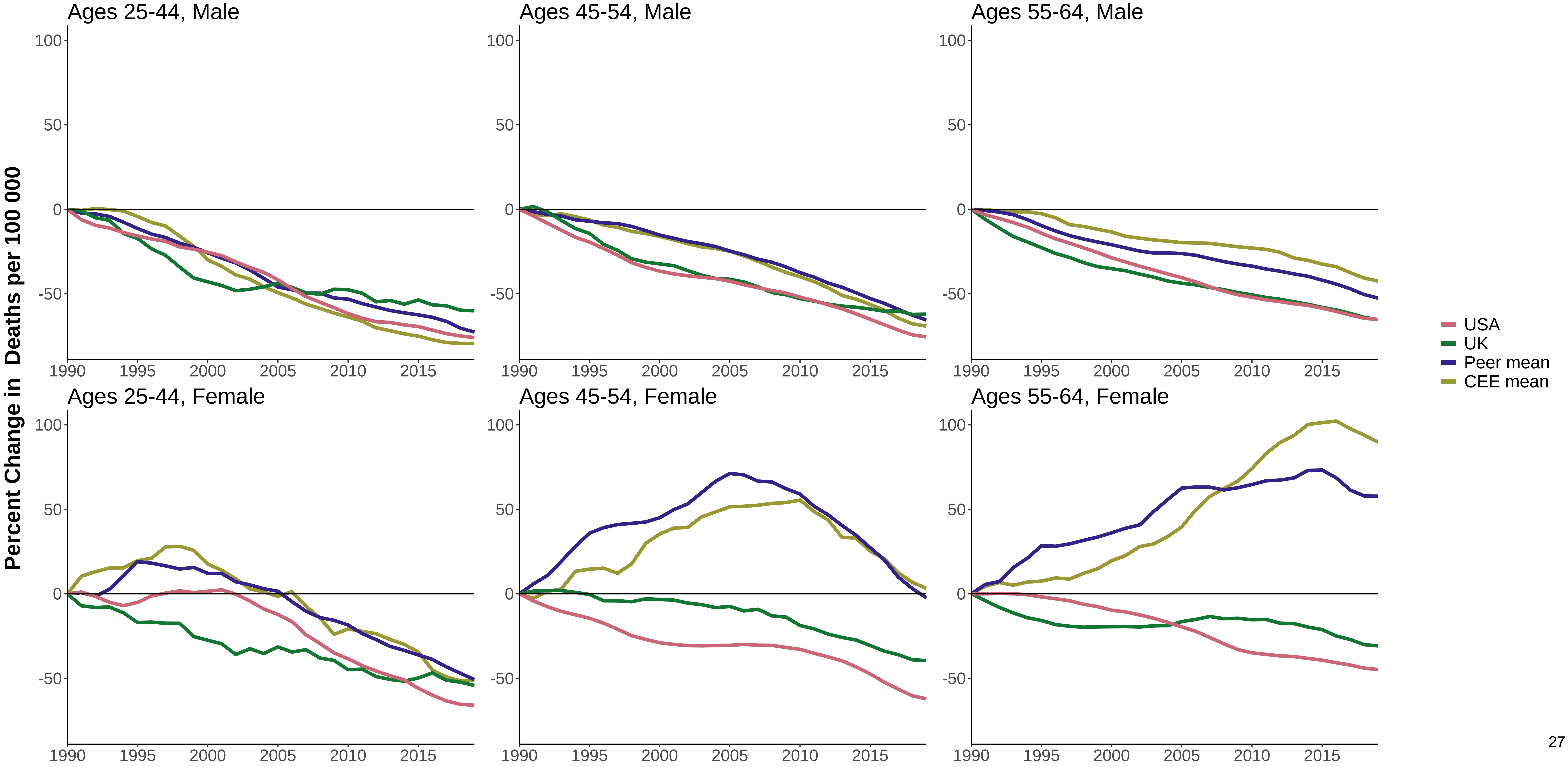

Figure S21. Percent Change in Age-Standardized Mortality from the Baseline Year (1990), All Other Cancers

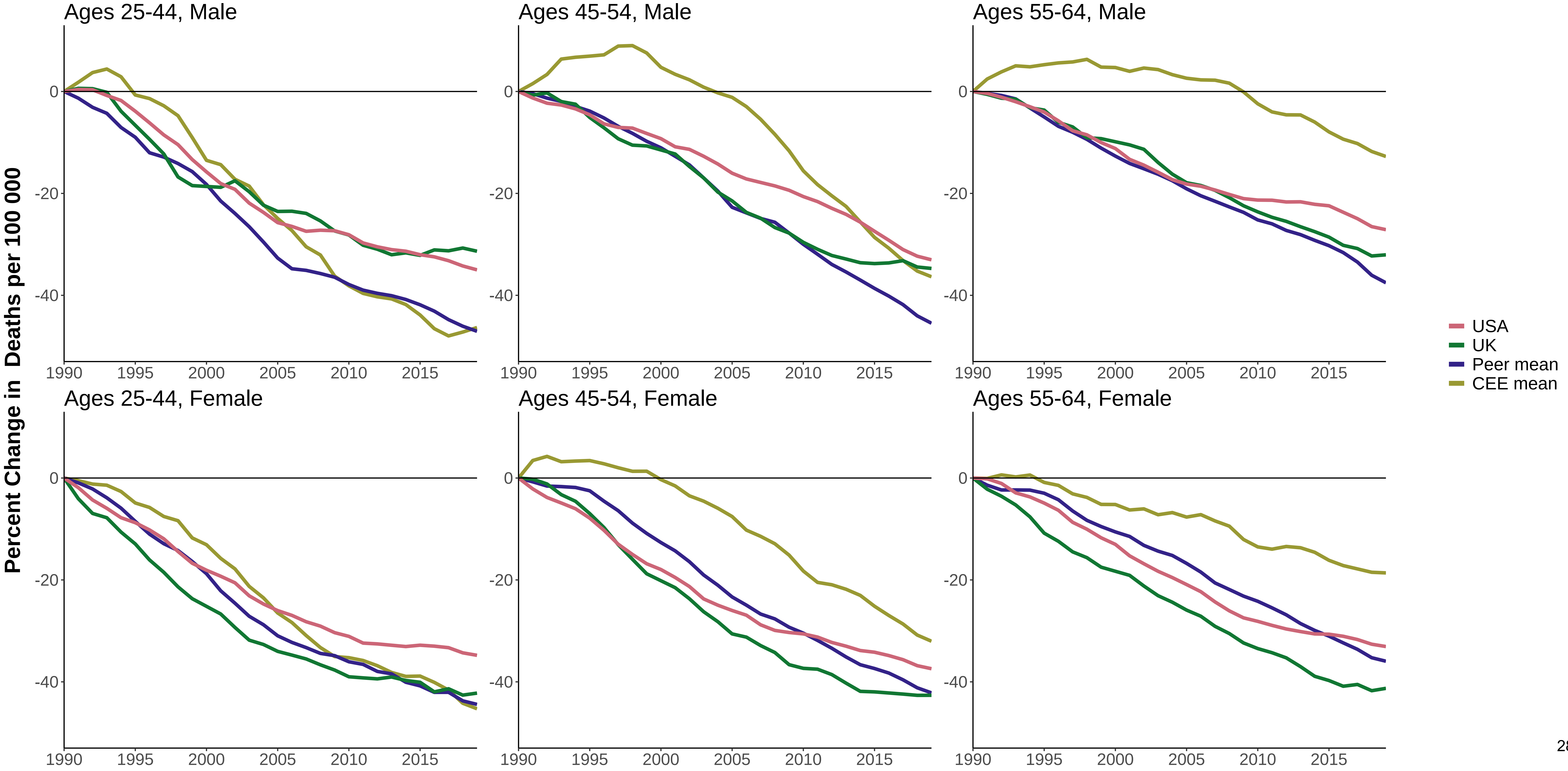

Figure S22. Percent Change in Age-Standardized Mortality from the Baseline Year (1990), Nervous System Diseases

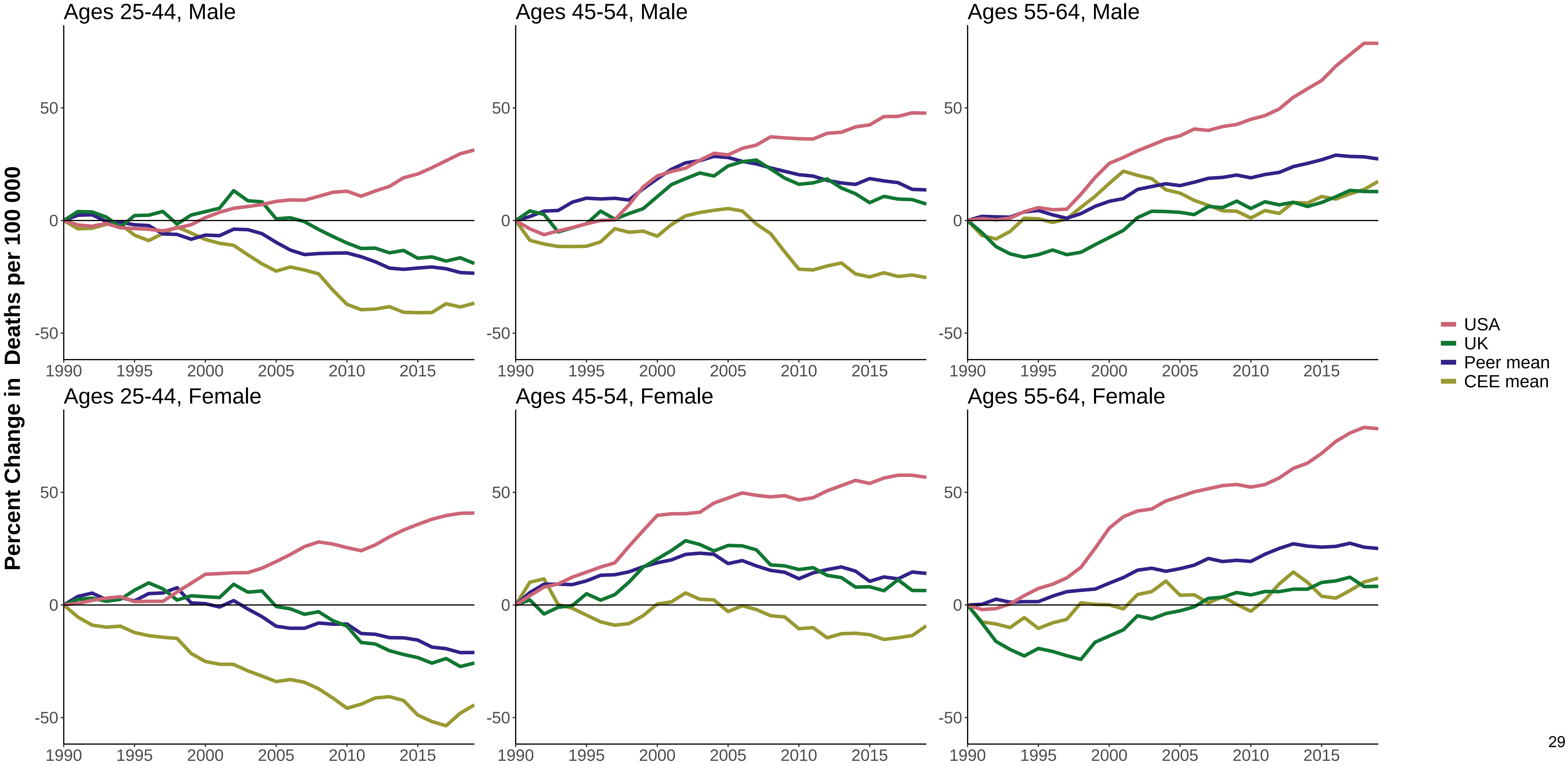

Figure S23. Percent Change in Age-Standardized Mortality from the Baseline Year (1990), Metabolic Diseases

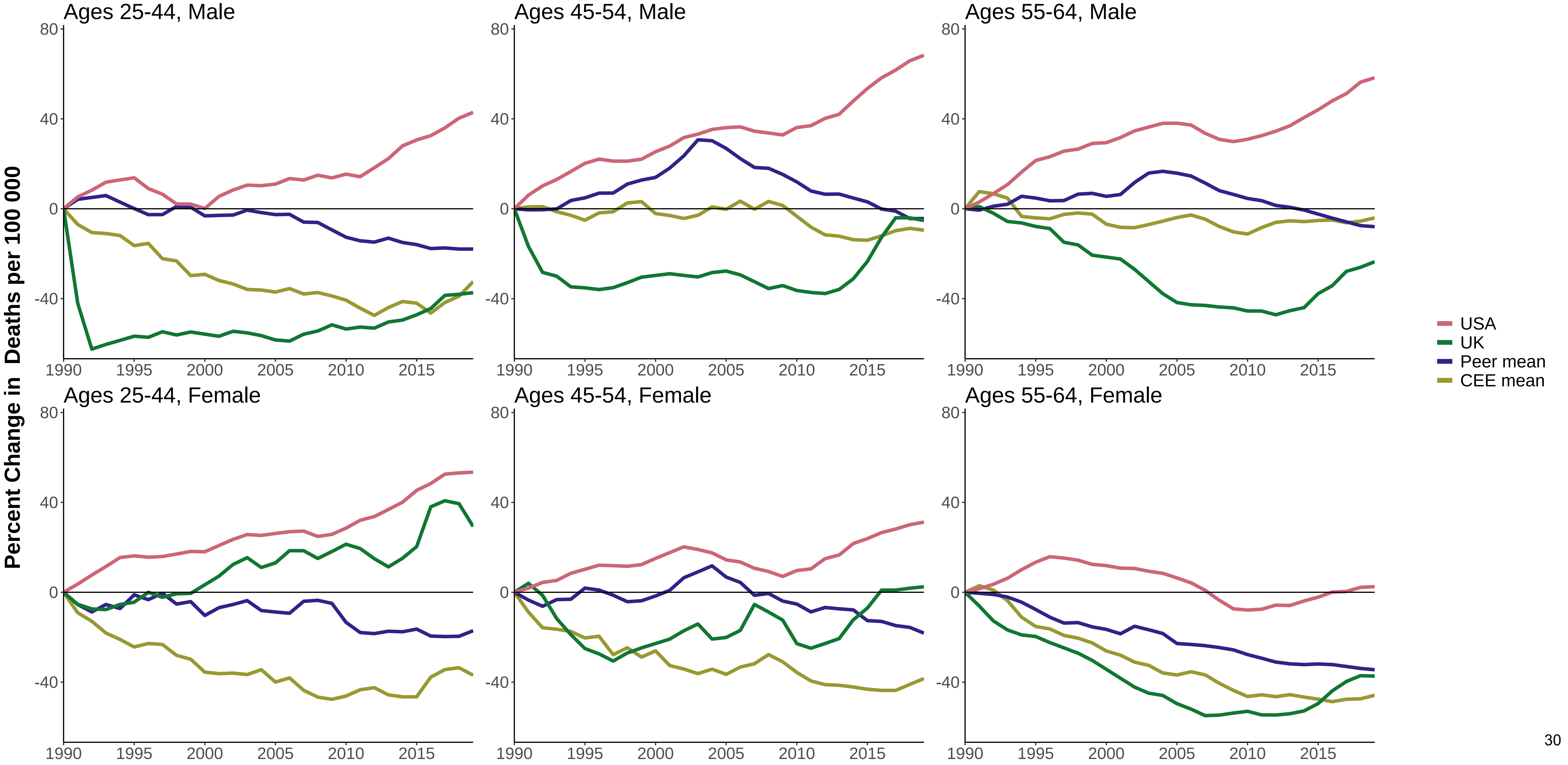

Figure S24. Percent Change in Age-Standardized Mortality from the Baseline Year (1990), Cardiovascular Disease

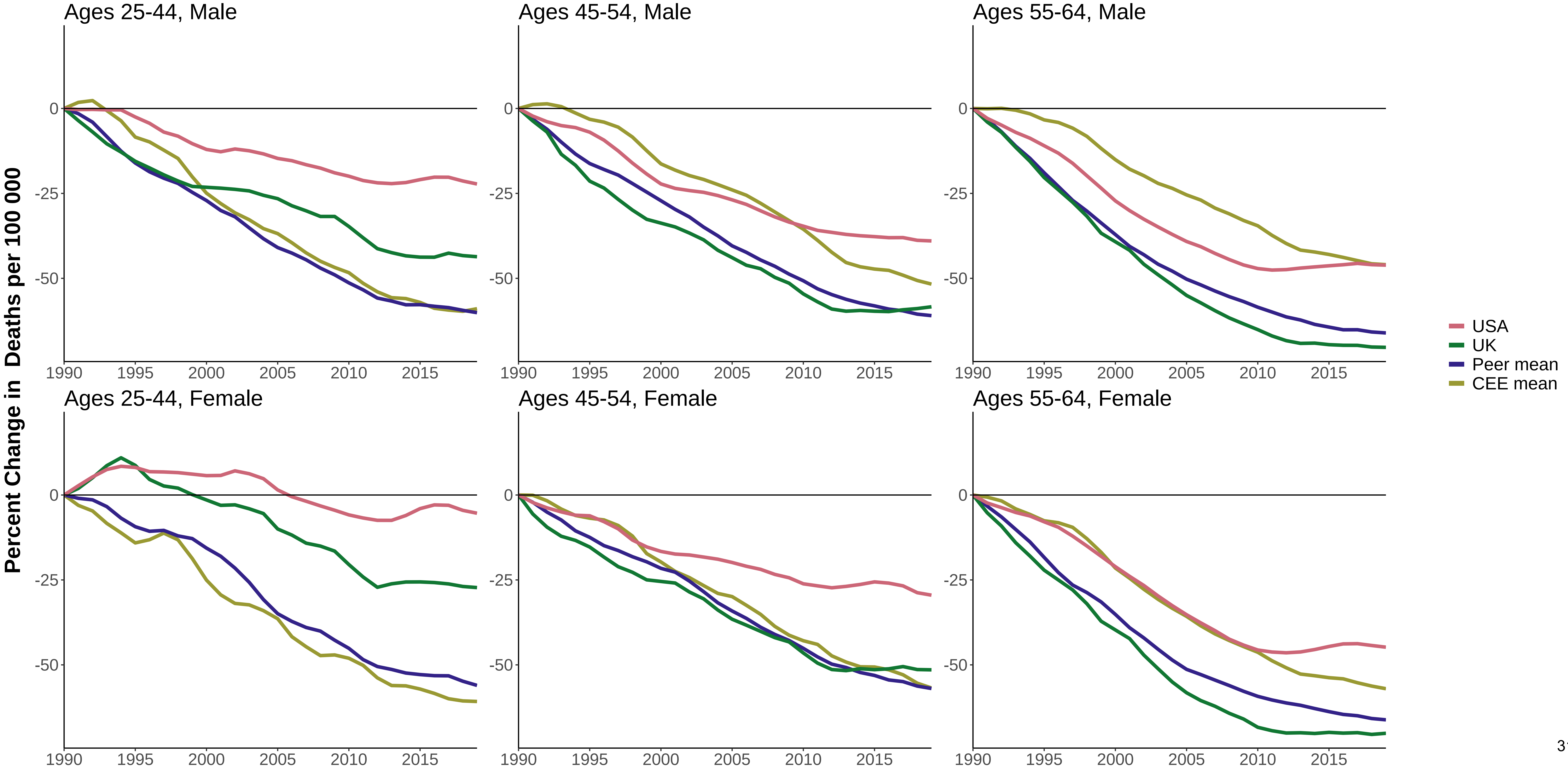

Figure S25. Percent Change in Age-Standardized Mortality from the Baseline Year (1990), Suicide

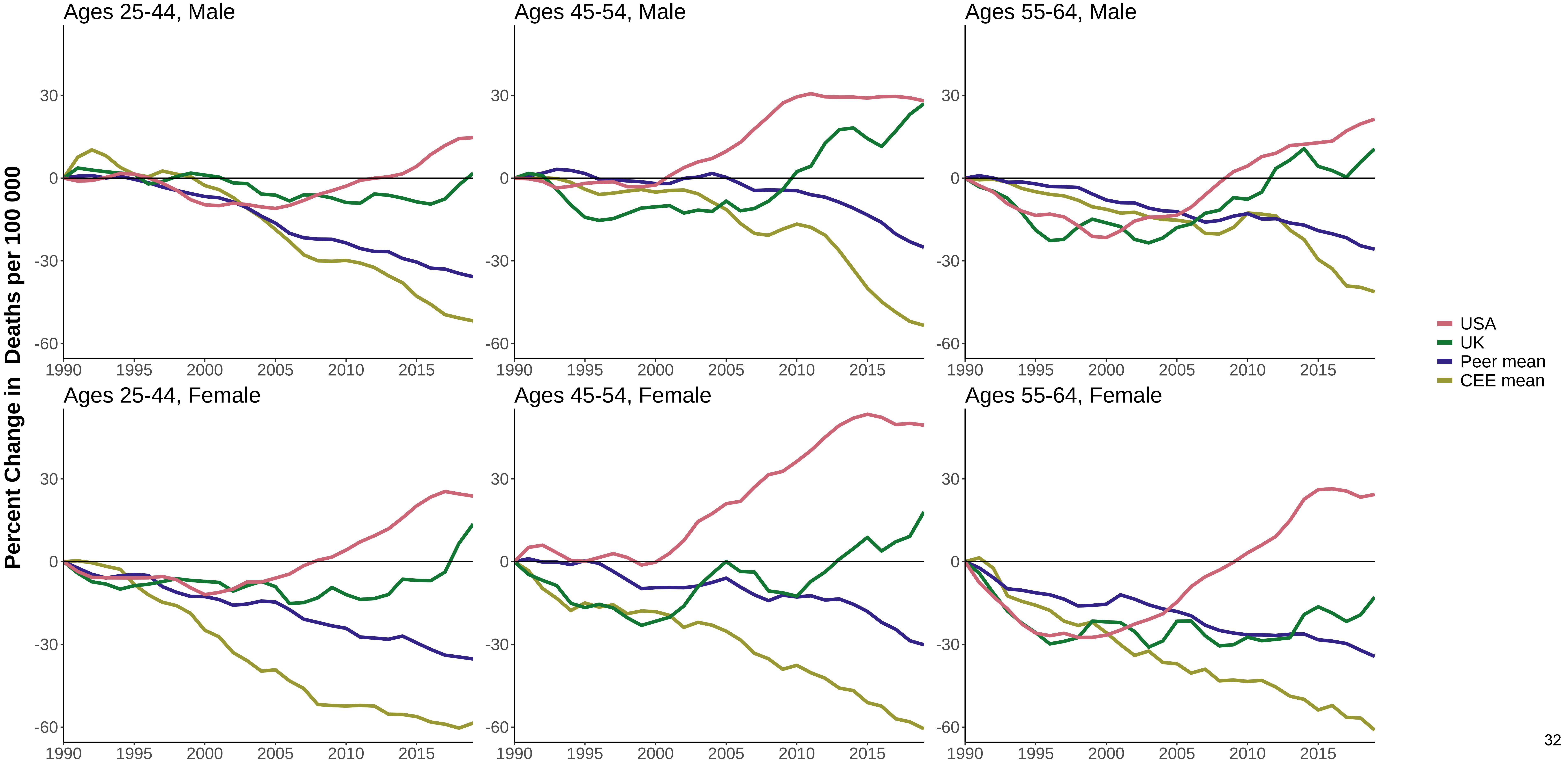

Figure S26. Percent Change in Age-Standardized Mortality from the Baseline Year (1990), Homicide

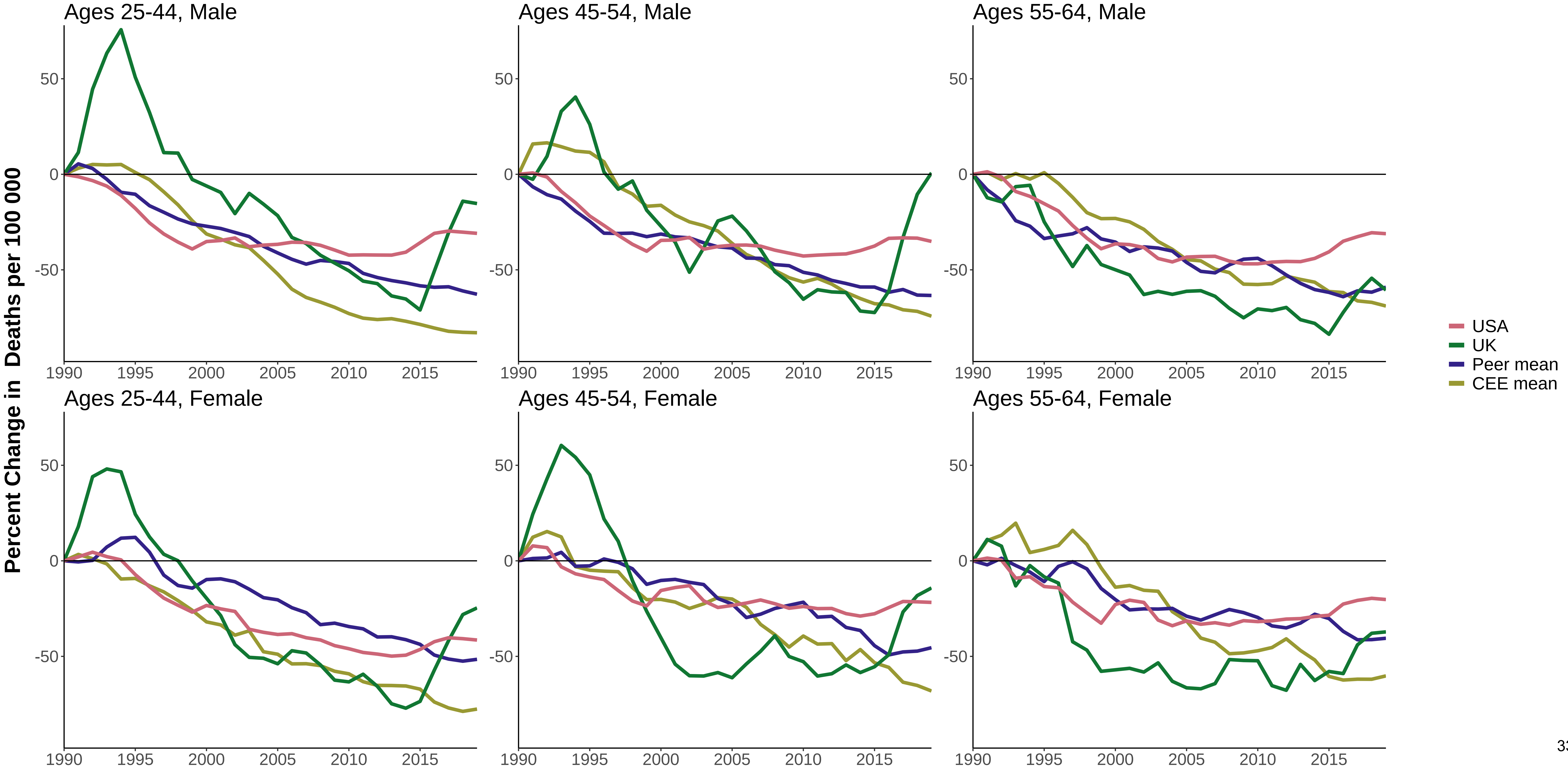

Figure S27. Percent Change in Age-Standardized Mortality from the Baseline Year (1990), Transport Accidents

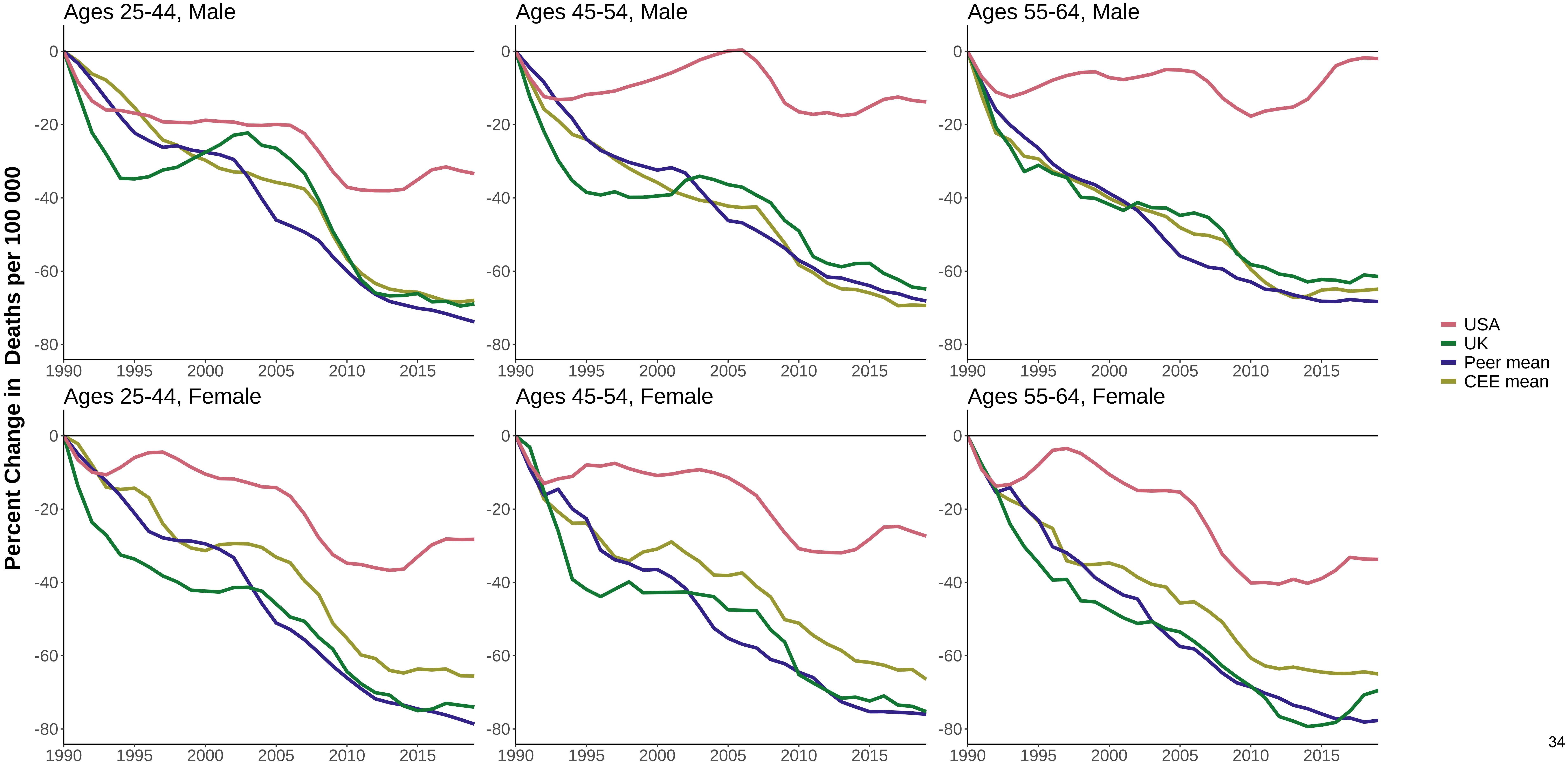

Figure S28. Percent Change in Age-Standardized Mortality from the Baseline Year (1990), Other External Causes

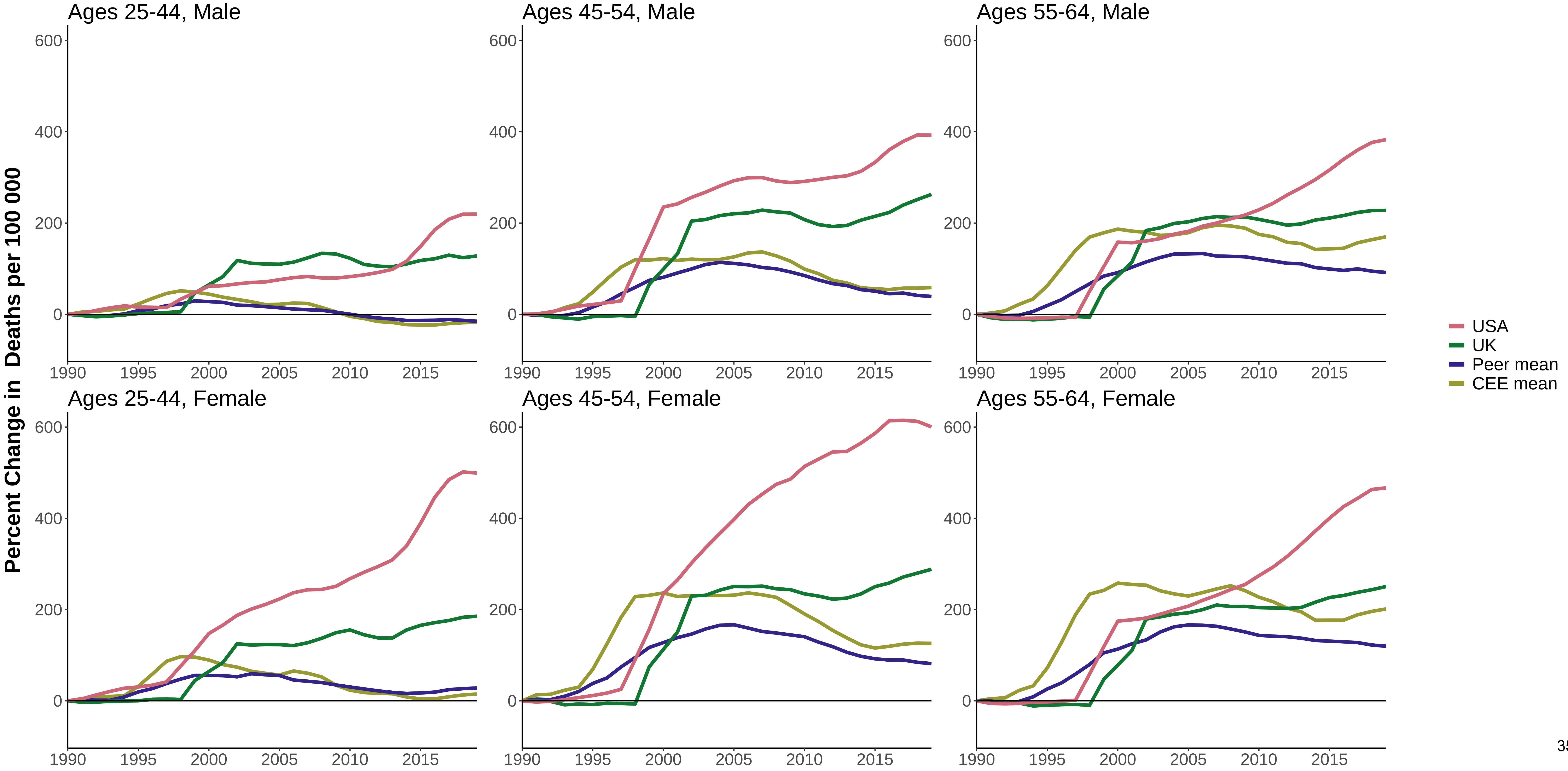

Figure S29. Percent Change in Age-Standardized Mortality from the Baseline Year (1990), All Other Causes

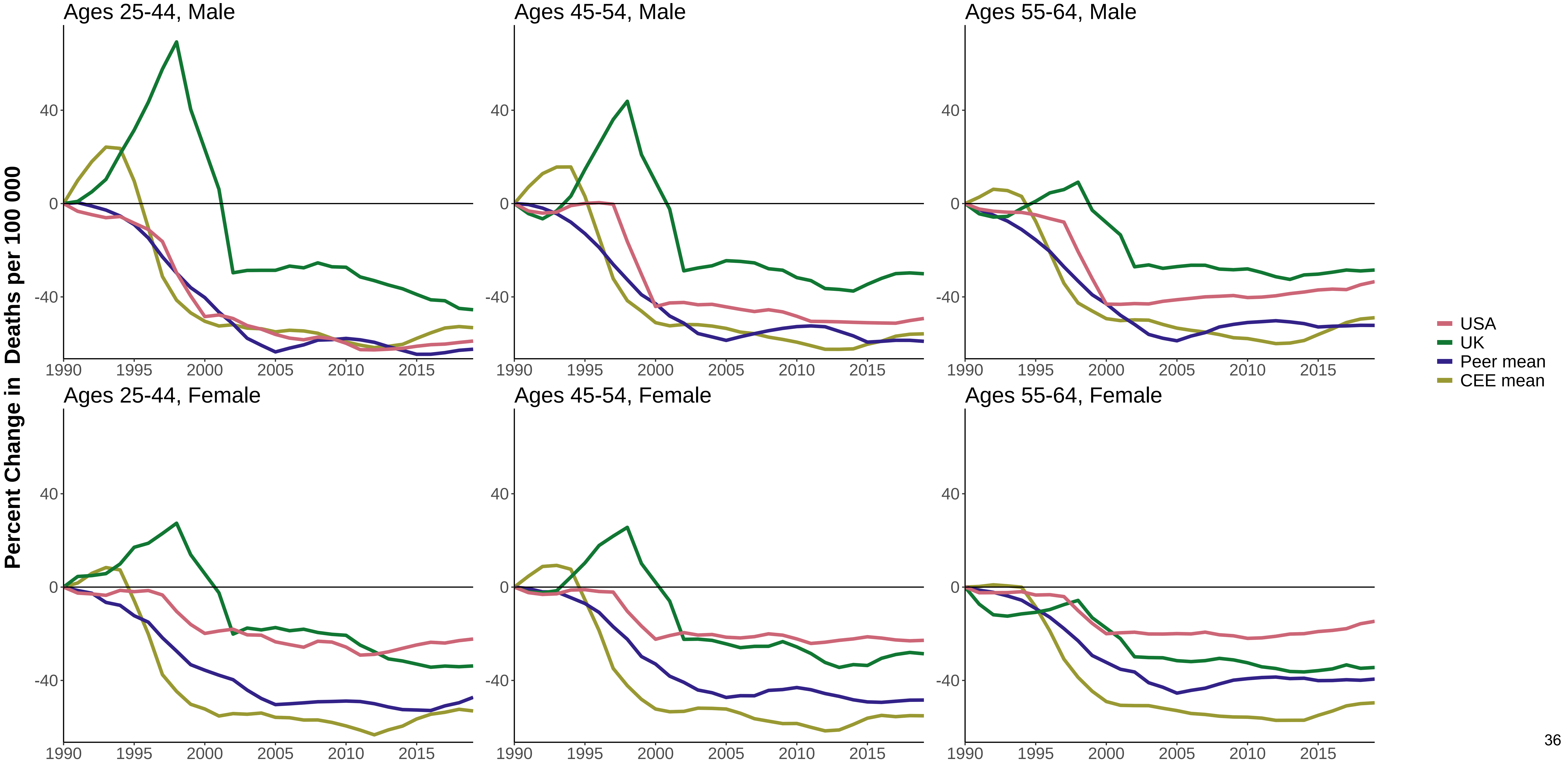

Figure S30. Percent Change in Age-Standardized Mortality from the Baseline Year (1990), All Causes

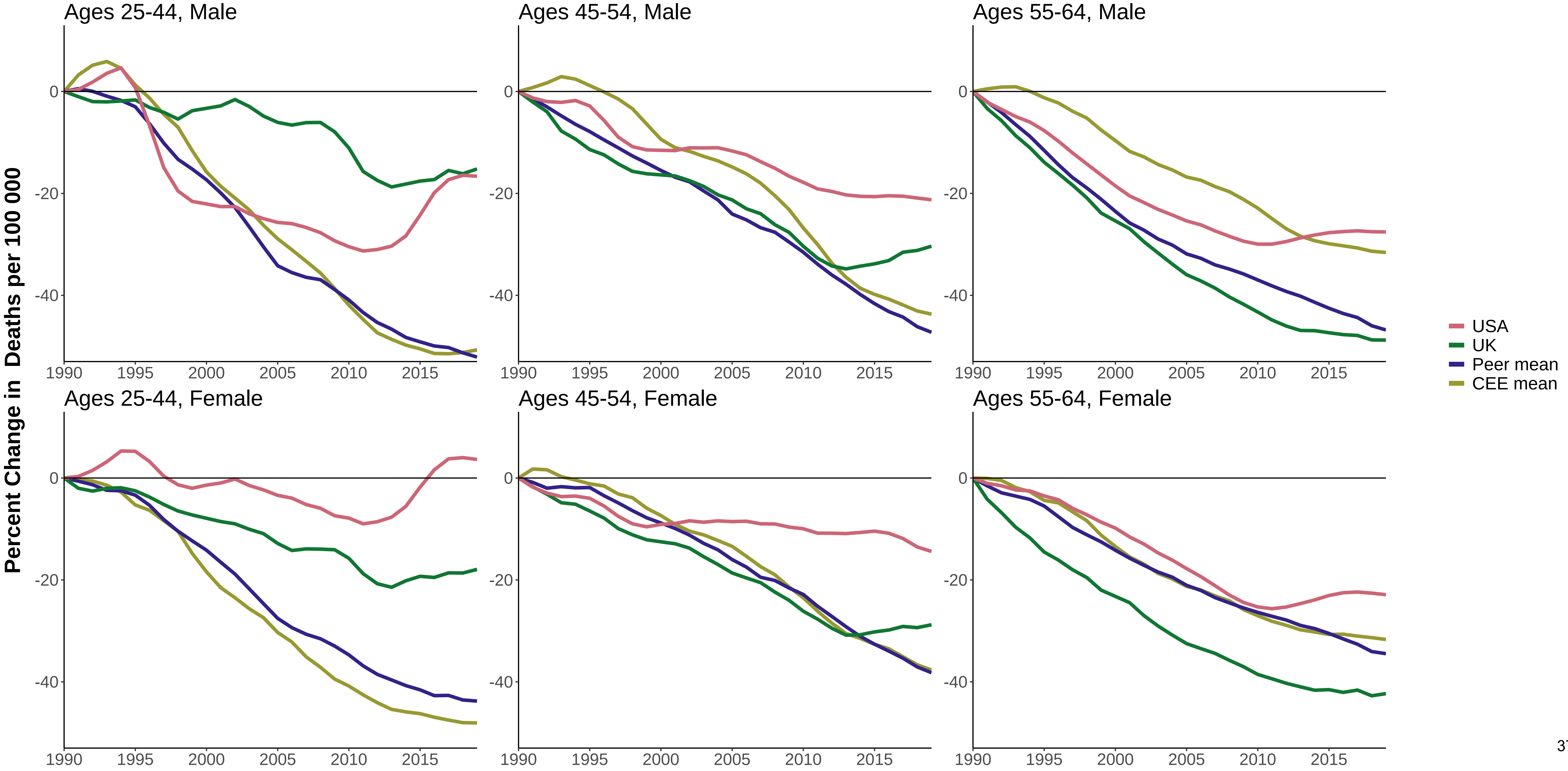

Figure S31. Percent Change in Age-Standardized Mortality from the Baseline Year (2000), Drug-Related Causes

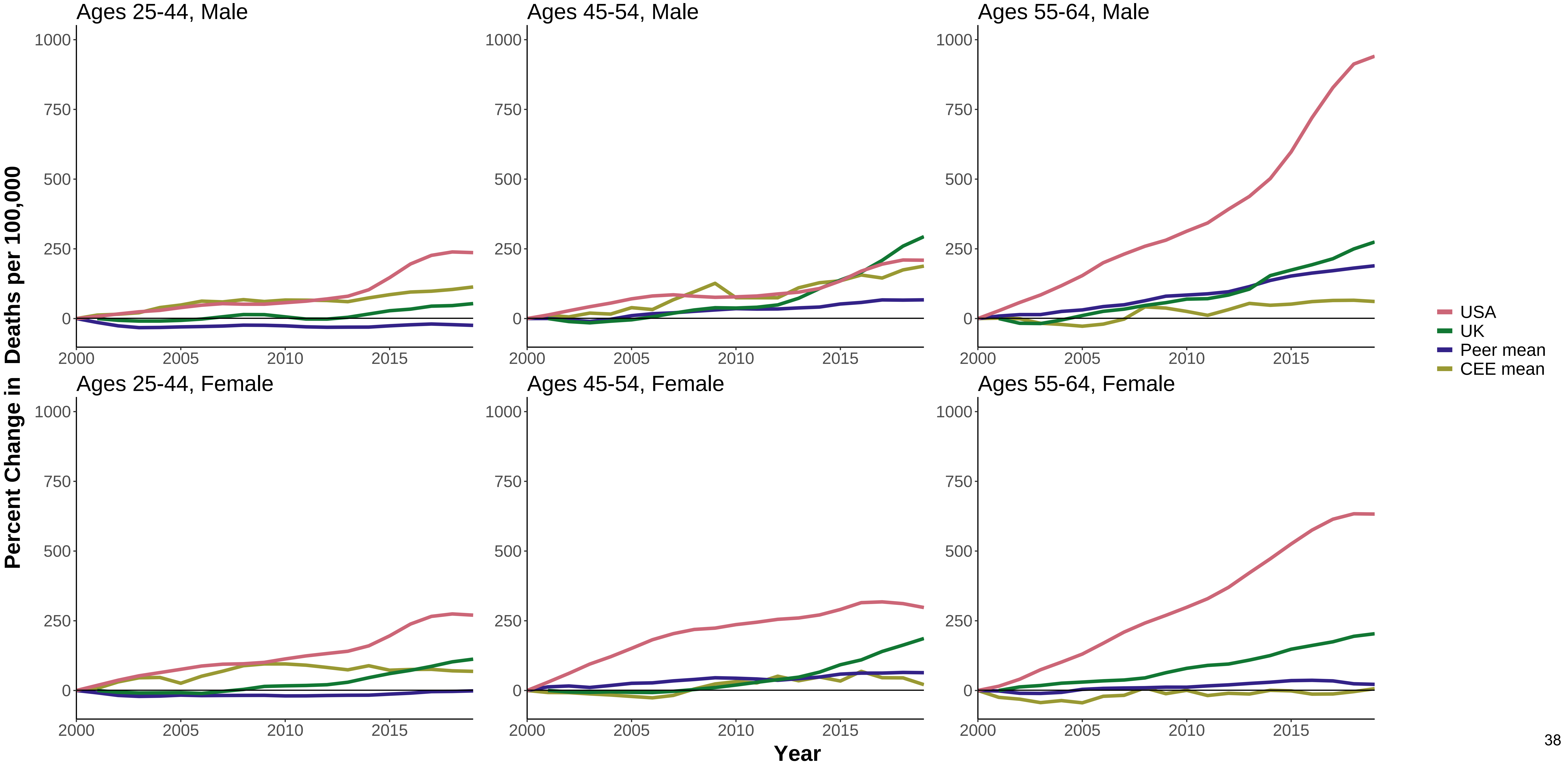

Figure S32. Percent Change in Age-Standardized Mortality from the Baseline Year (2000), Alcohol-Related Causes

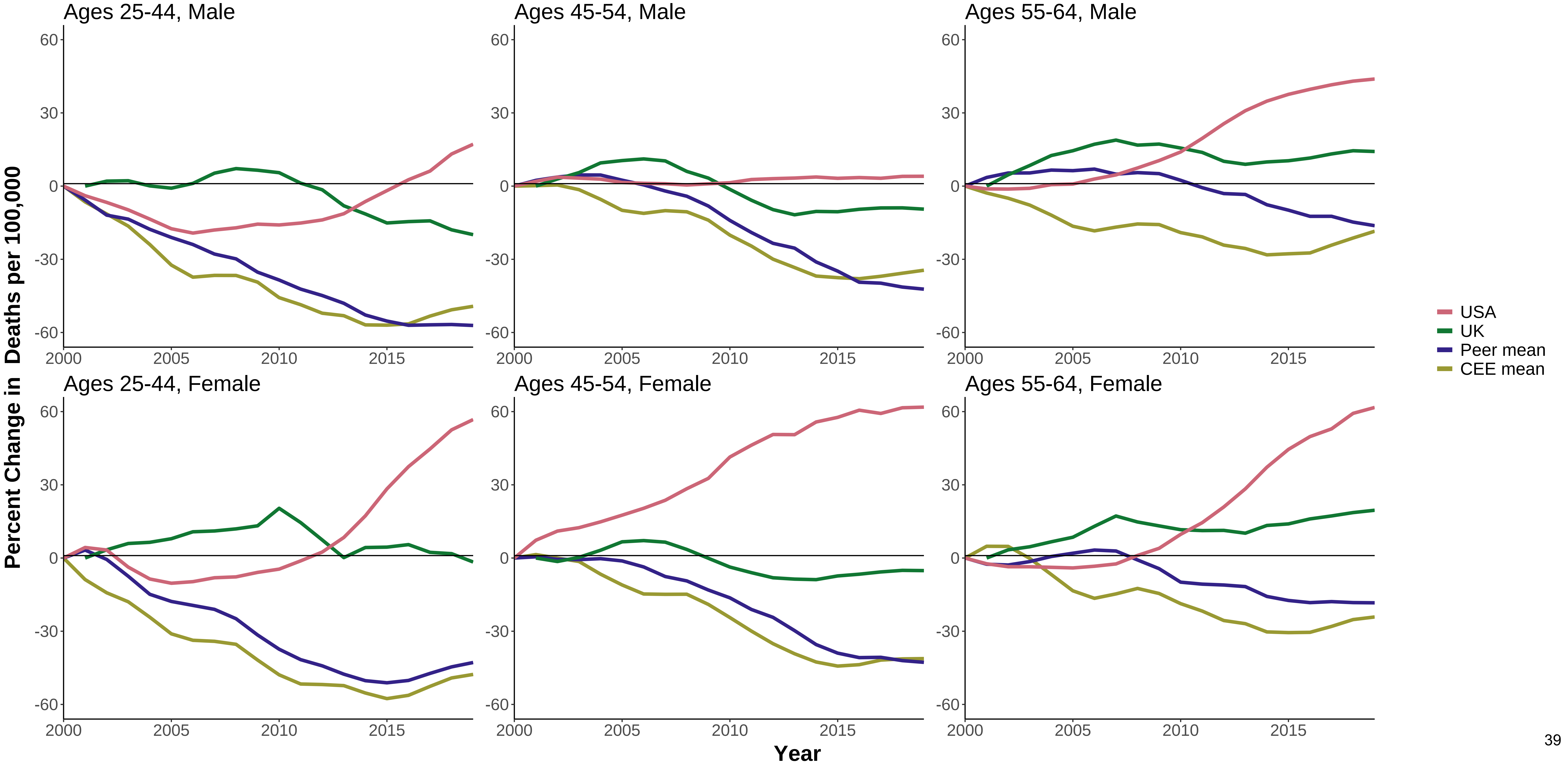

Figure S33. Percent Change in All-Cause Mortality Between 1990 and 2019/Males, Ages 25-44

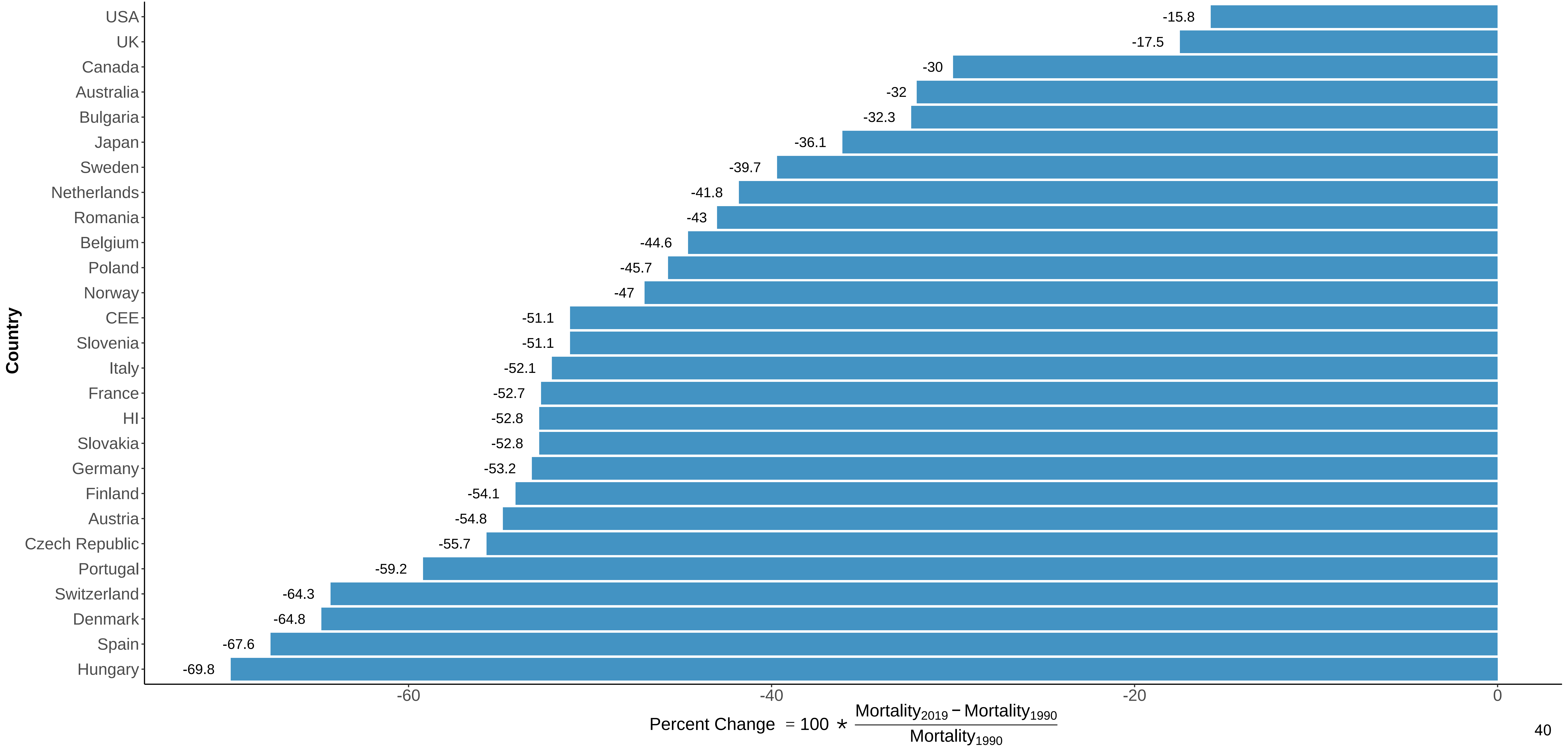

Figure S34. Percent Change in All-Cause Mortality Between 1990 and 2019/Males, Ages 45-54

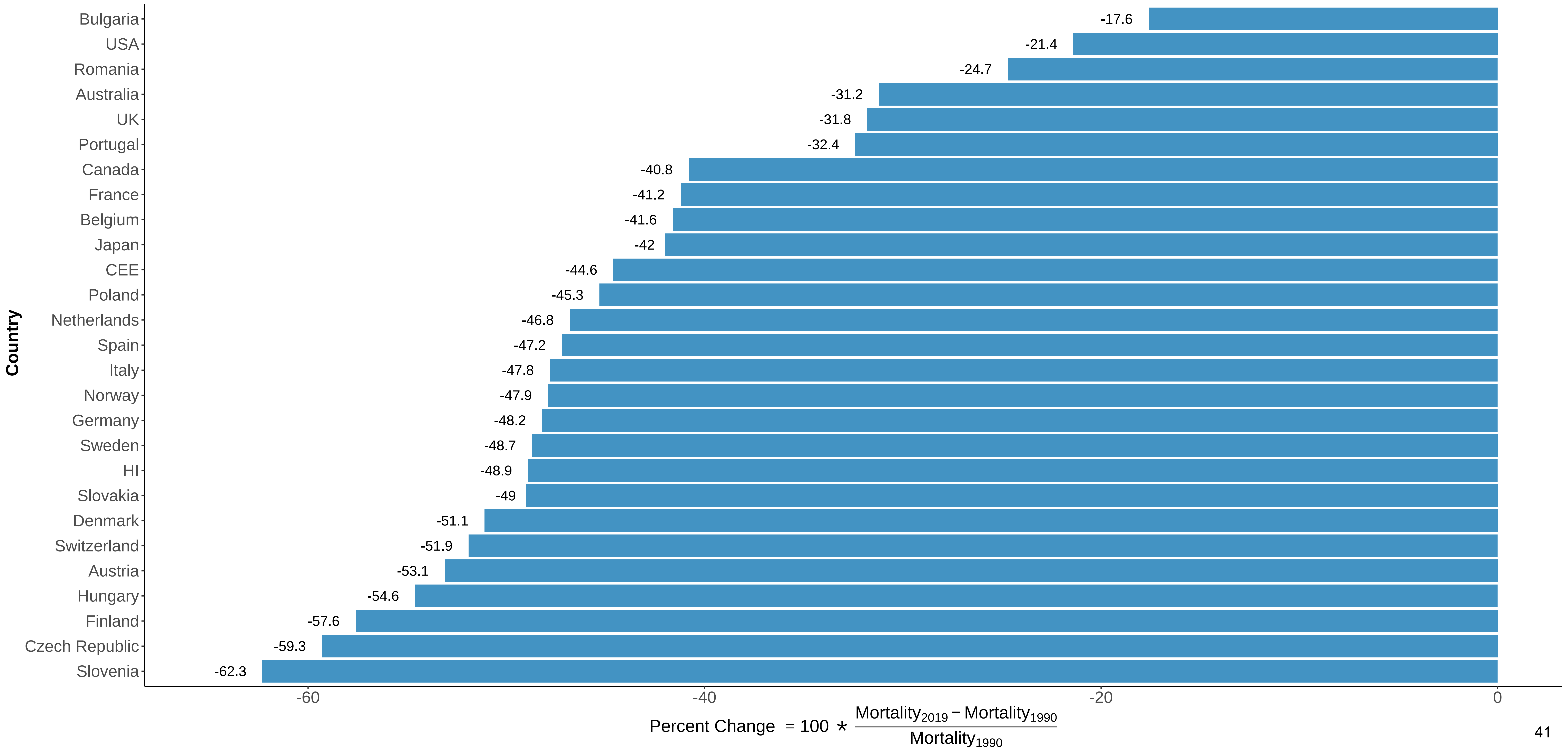

Figure S35. Percent Change in All-Cause Mortality Between 1990 and 2019/Males, Ages 55-64

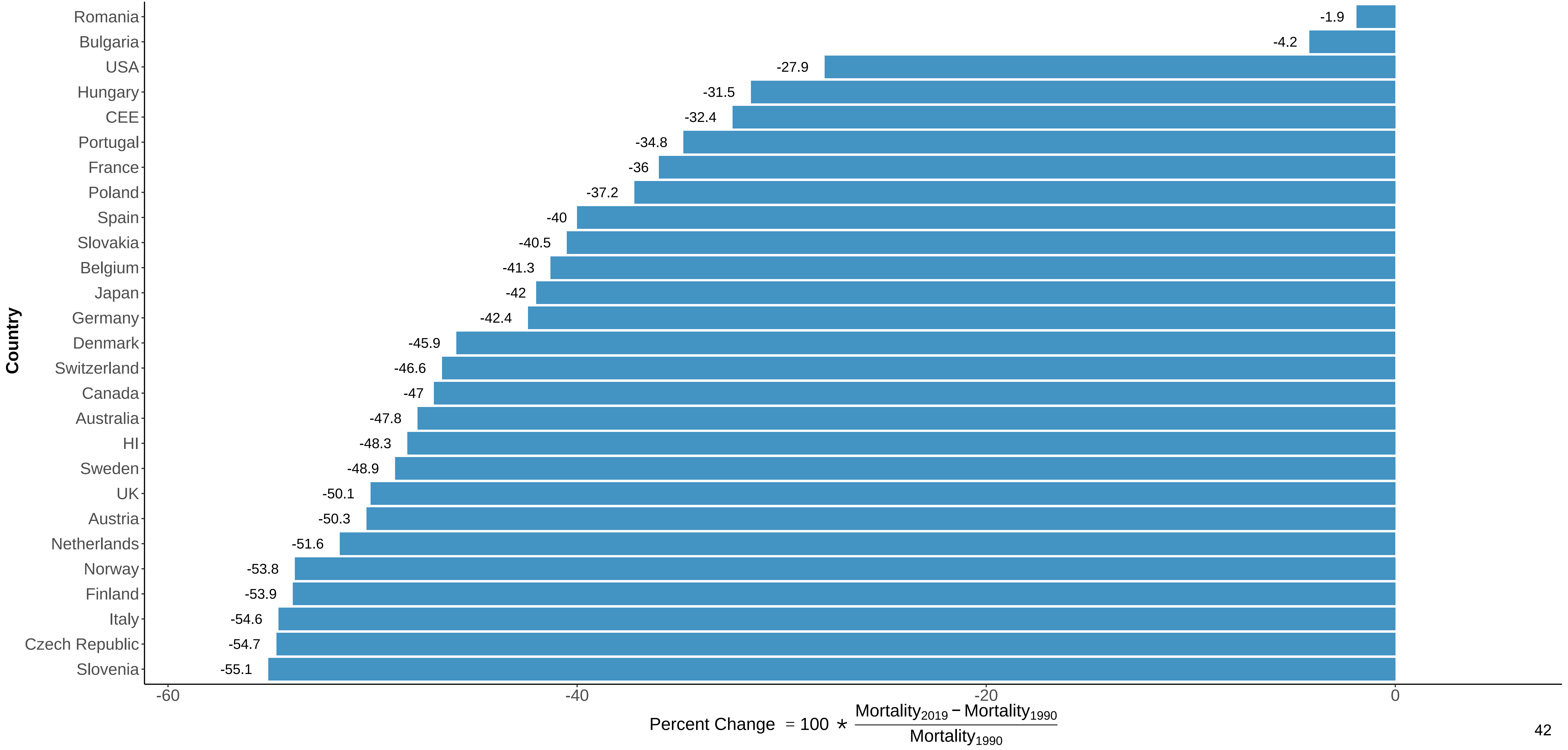

Figure S36. Percent Change in All-Cause Mortality Between 1990 and 2019/Females, Ages 25-44

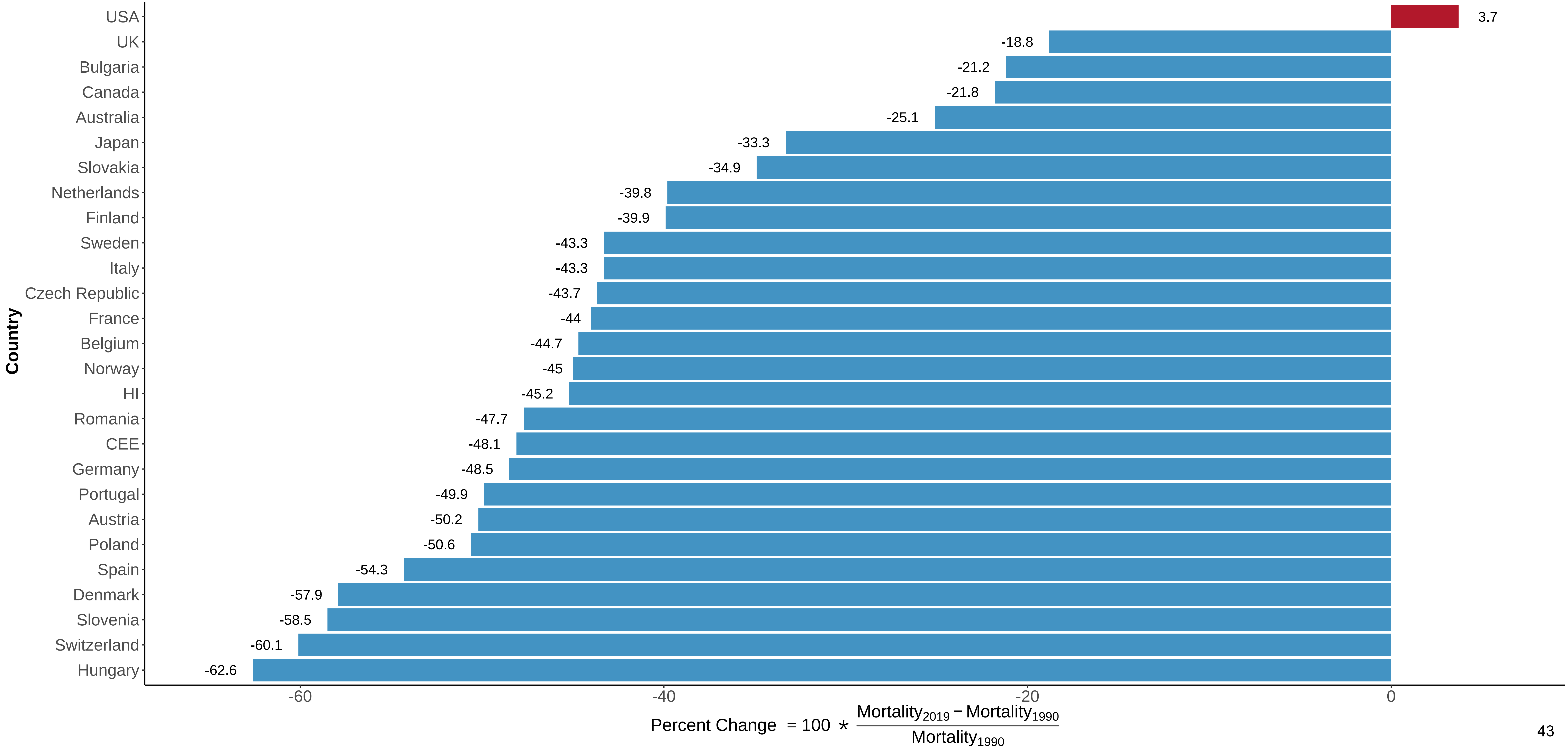

Figure S37. Percent Change in All-Cause Mortality Between 1990 and 2019/Females, Ages 45-54

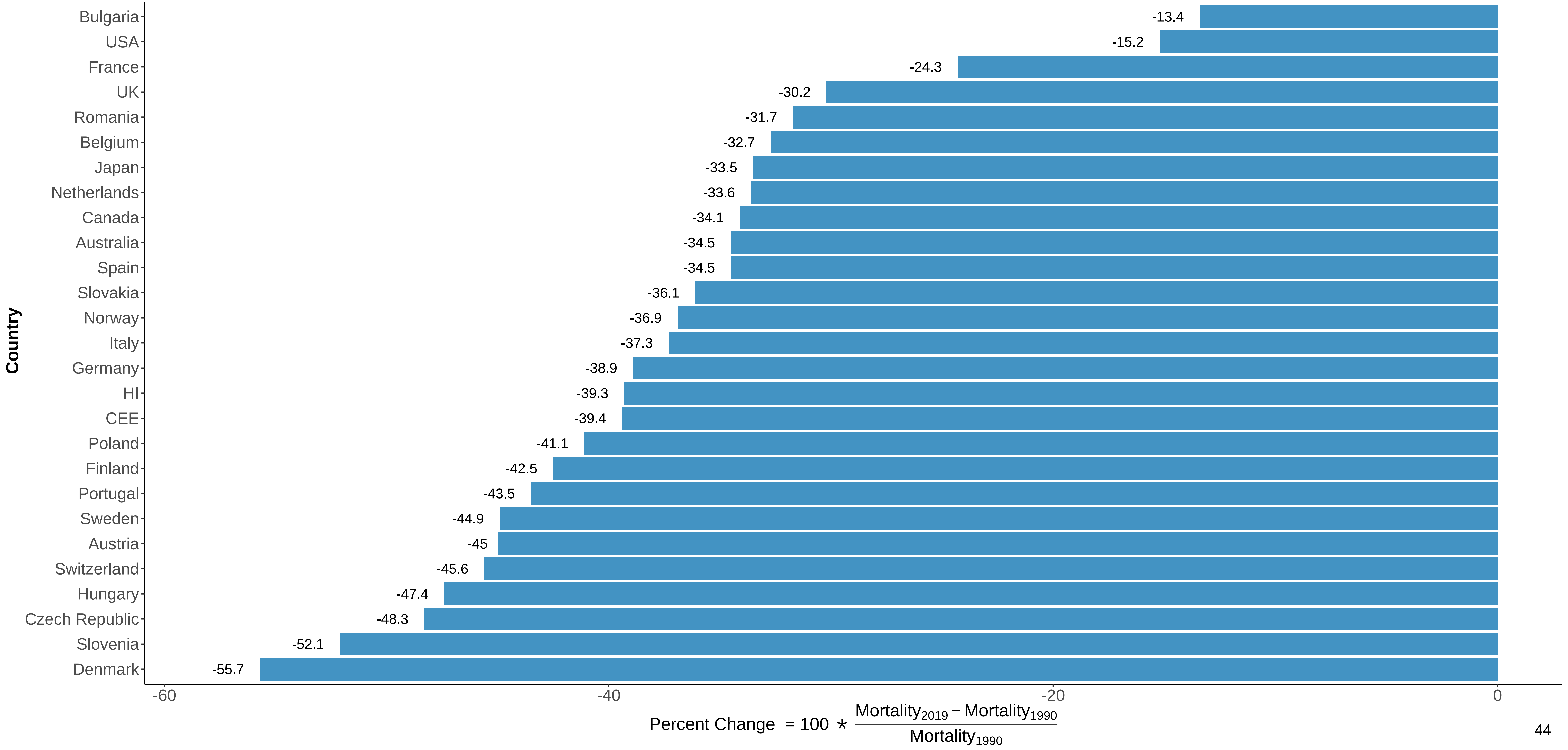

Figure S38. Percent Change in All-Cause Mortality Between 1990 and 2019/Females, Ages 55-64

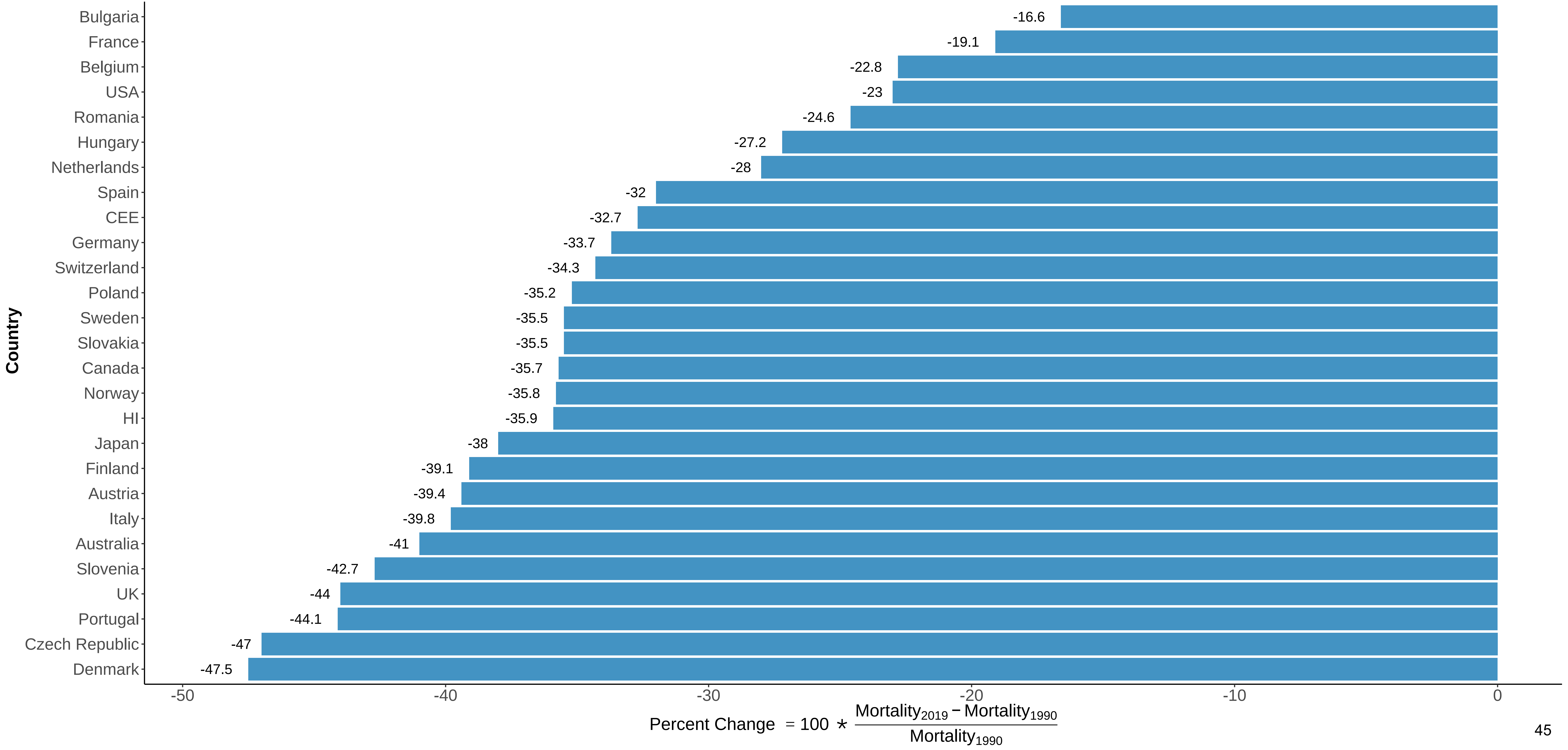

Figure S39. Three-Year Moving Average of Male Mortality from Infectious and Parasitic Diseases at Ages 25-44

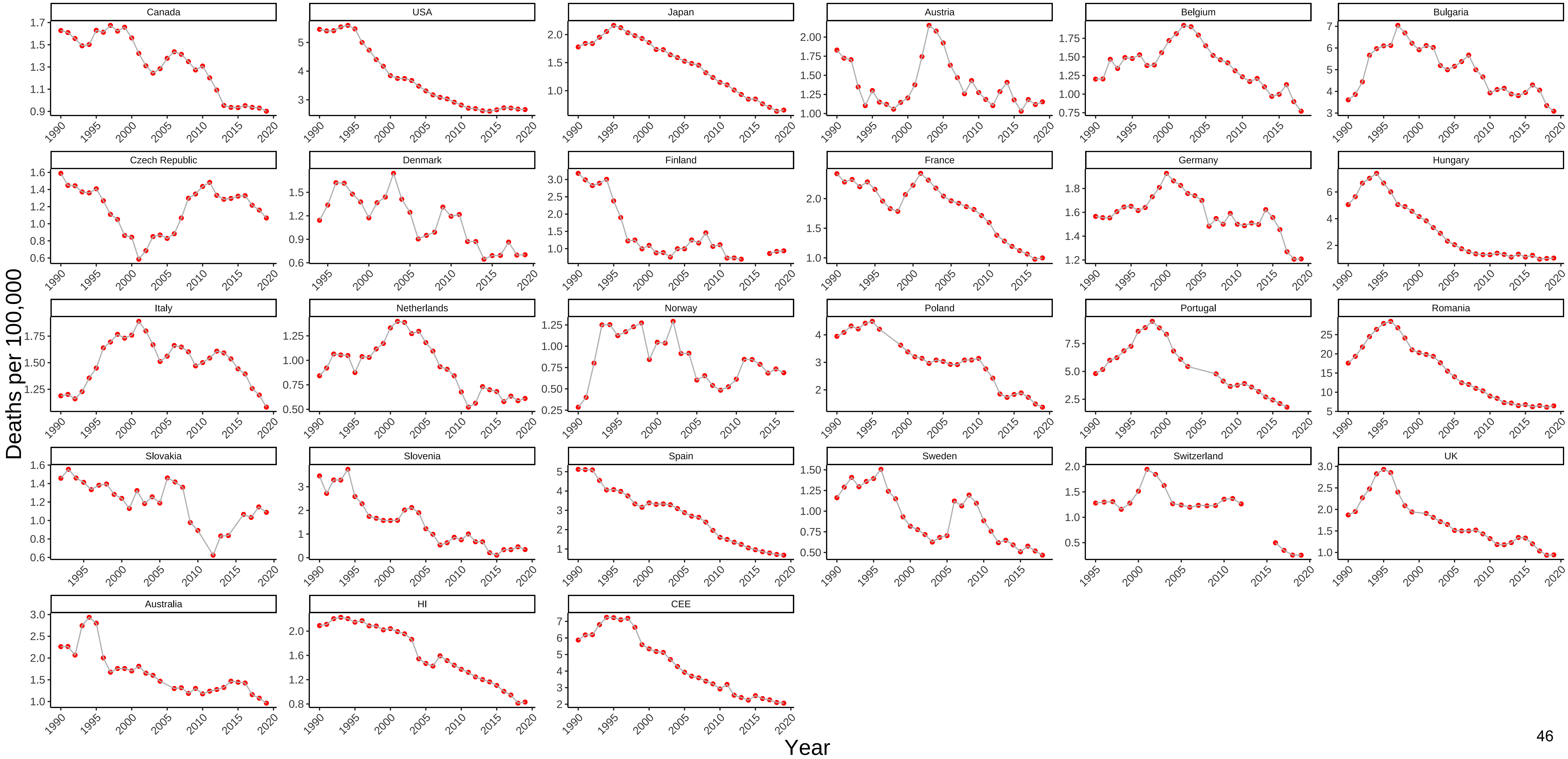

Figure S40. Three-Year Moving Average of Female Mortality from Infectious and Parasitic Diseases at Ages 25-44

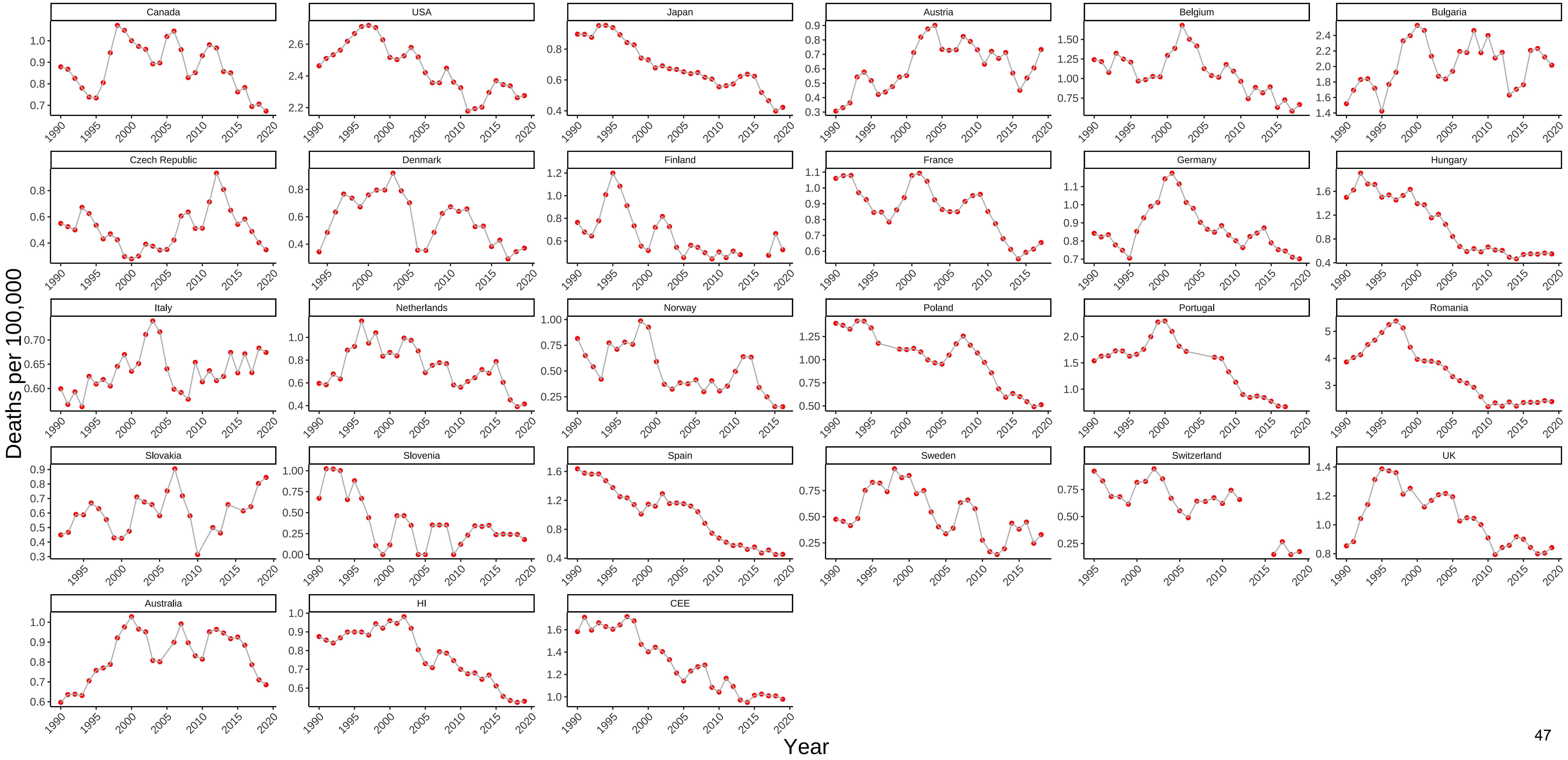

Figure S41. Three-Year Moving Average of Male Mortality from HIV/AIDS at Ages 25-44

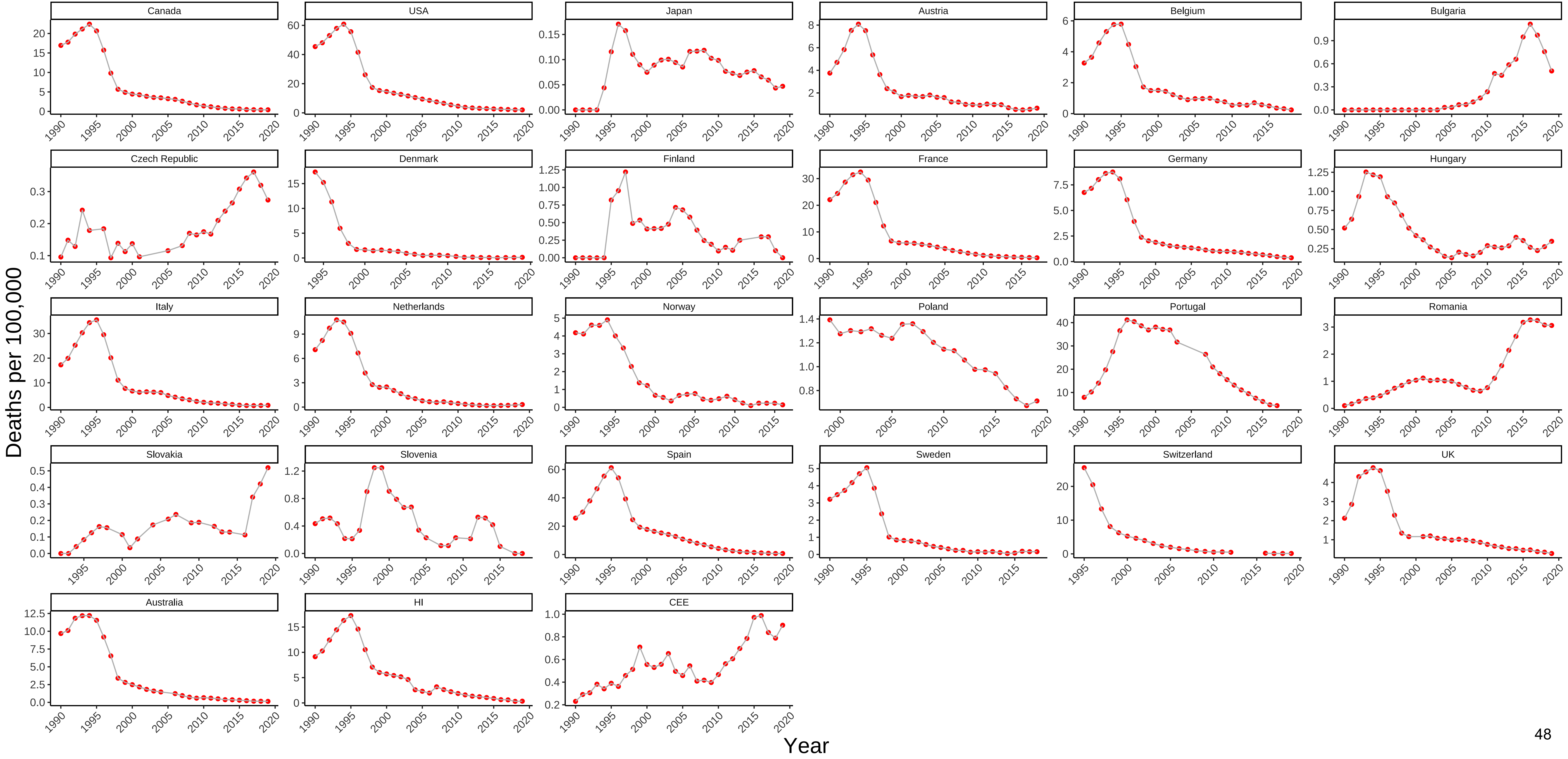

Figure S42. Three-Year Moving Average of Female Mortality from HIV/AIDS at Ages 25-44

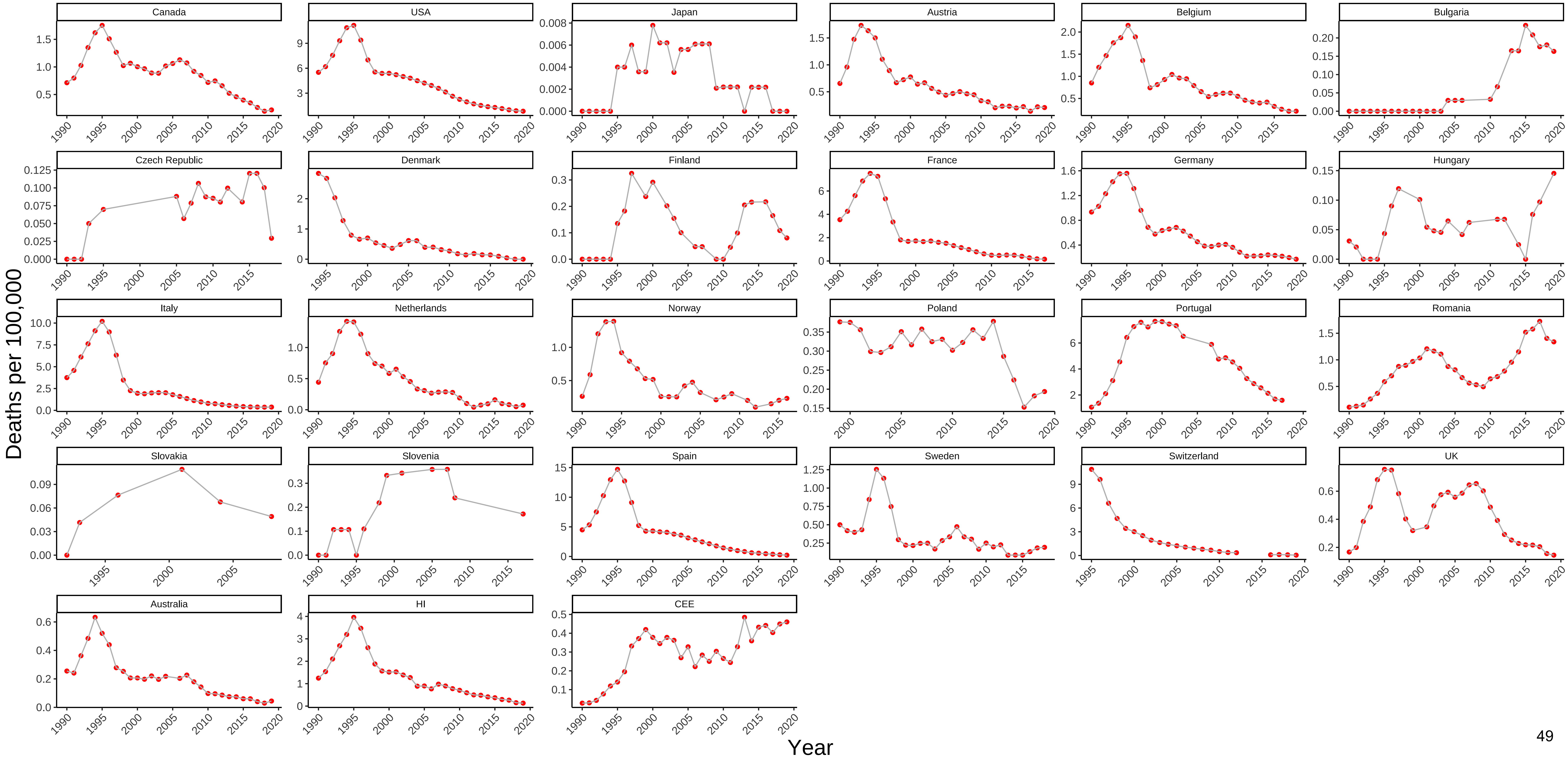

Figure S43. Three-Year Moving Average of Male Mortality from Respiratory Diseases at Ages 25-44

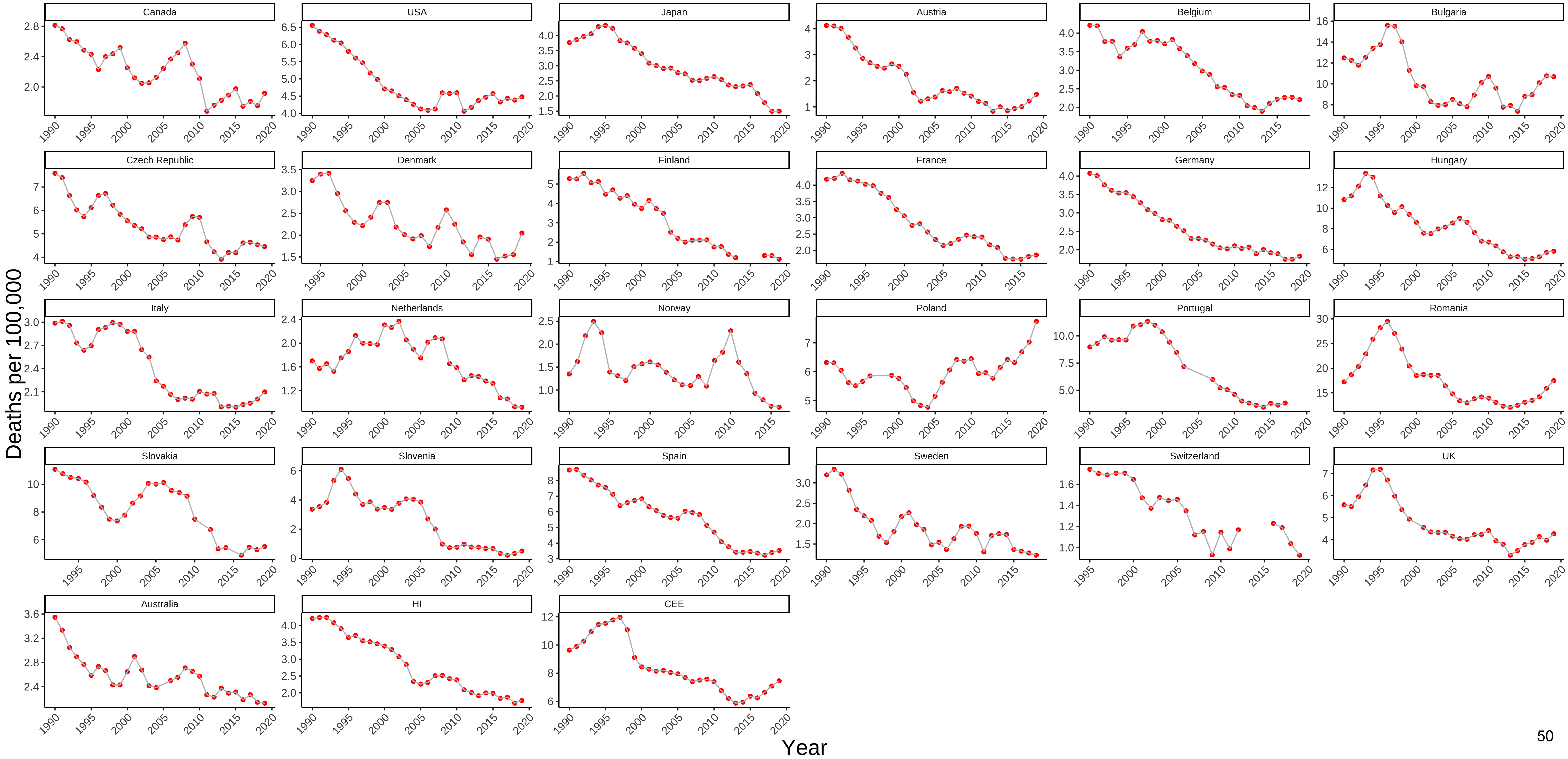

Figure S44. Three-Year Moving Average of Female Mortality from Respiratory Diseases at Ages 25-44

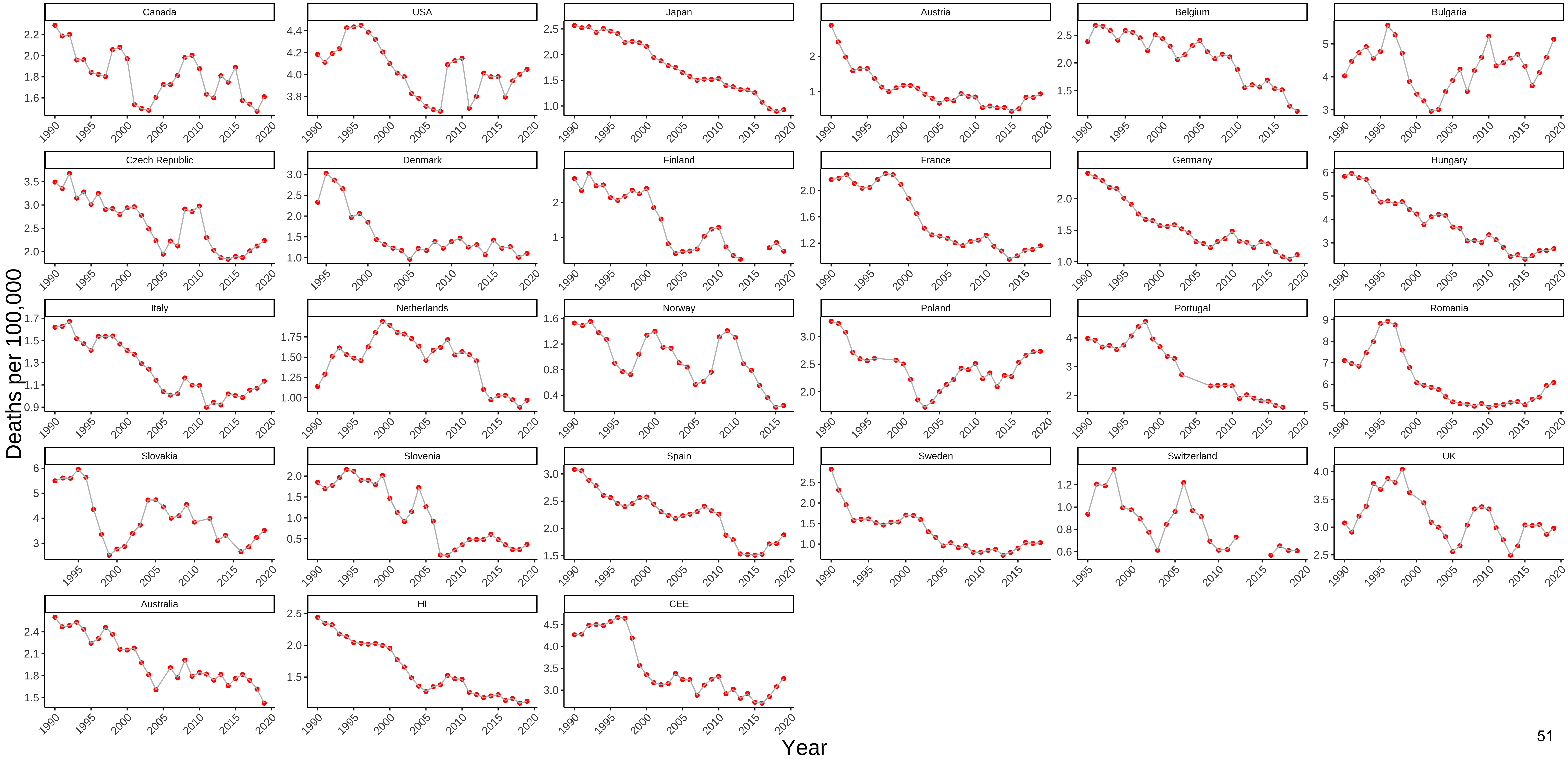

Figure S45. Three-Year Moving Average of Male Mortality from Trachea/Bronchus, Lung Cancers at Ages 25-44

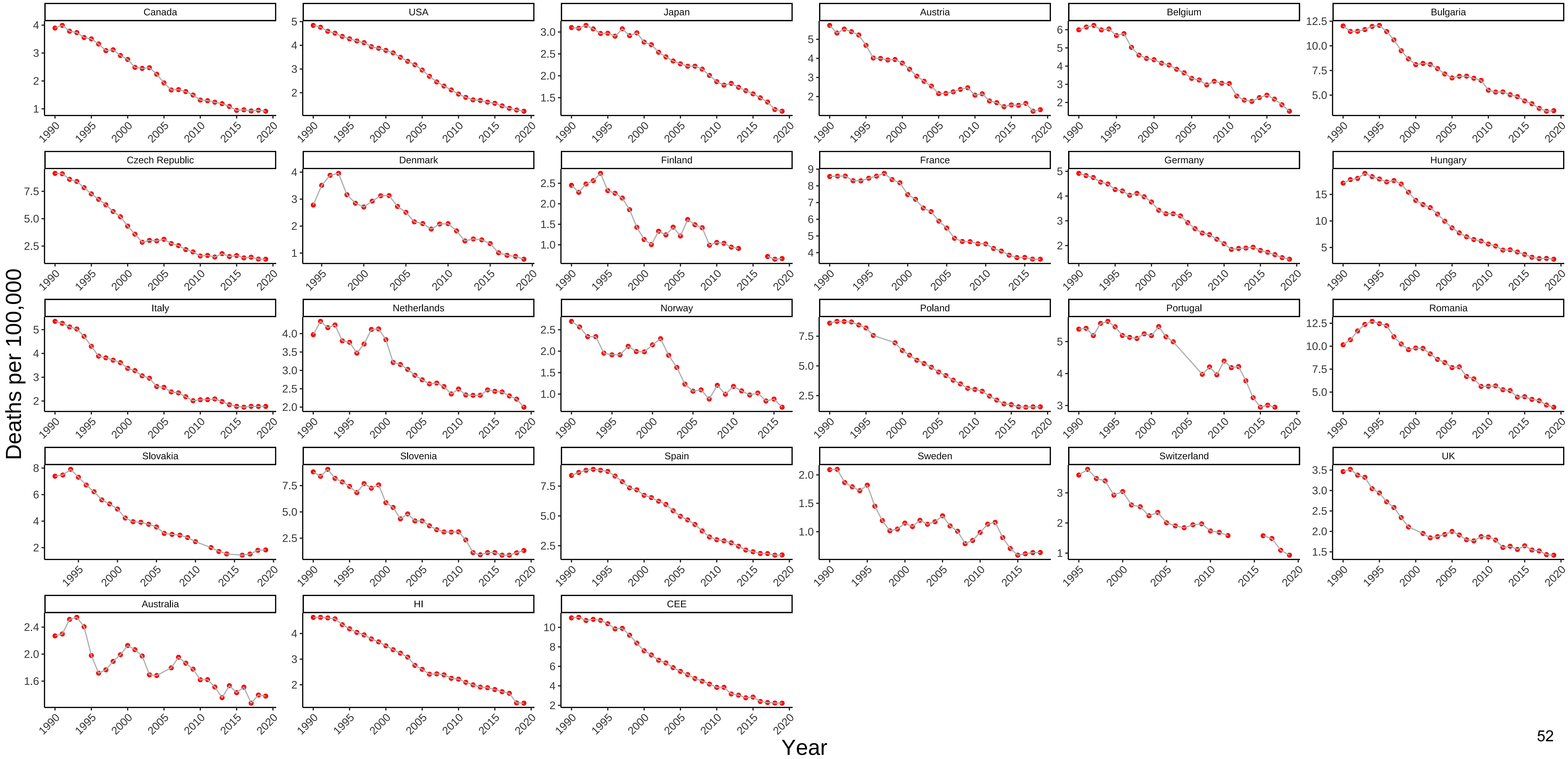

Figure S46. Three-Year Moving Average of Female Mortality from Trachea/Bronchus, Lung Cancers at Ages 25-44

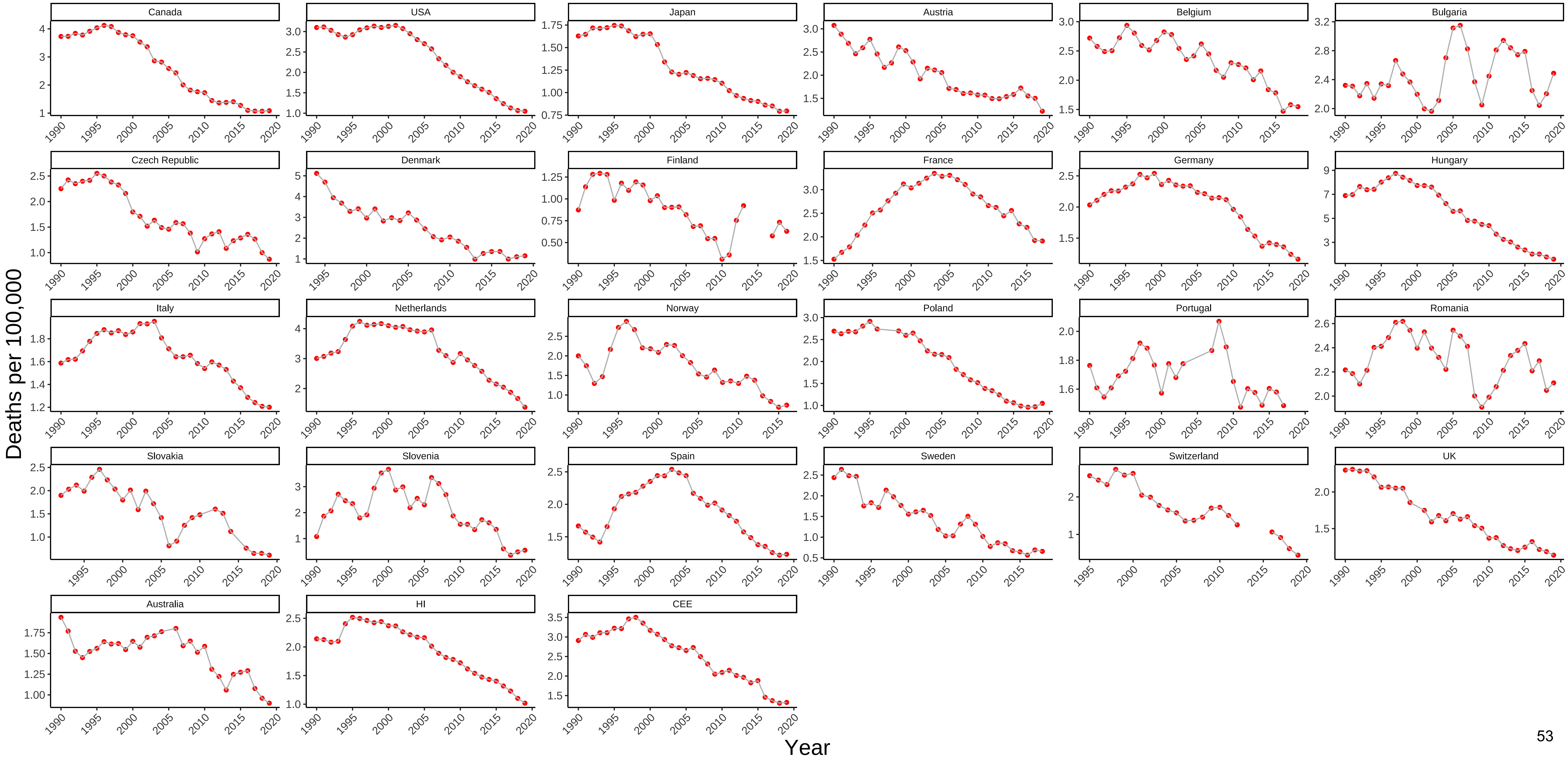

Figure S47. Three-Year Moving Average of Male Mortality from All Other Cancers at Ages 25-44

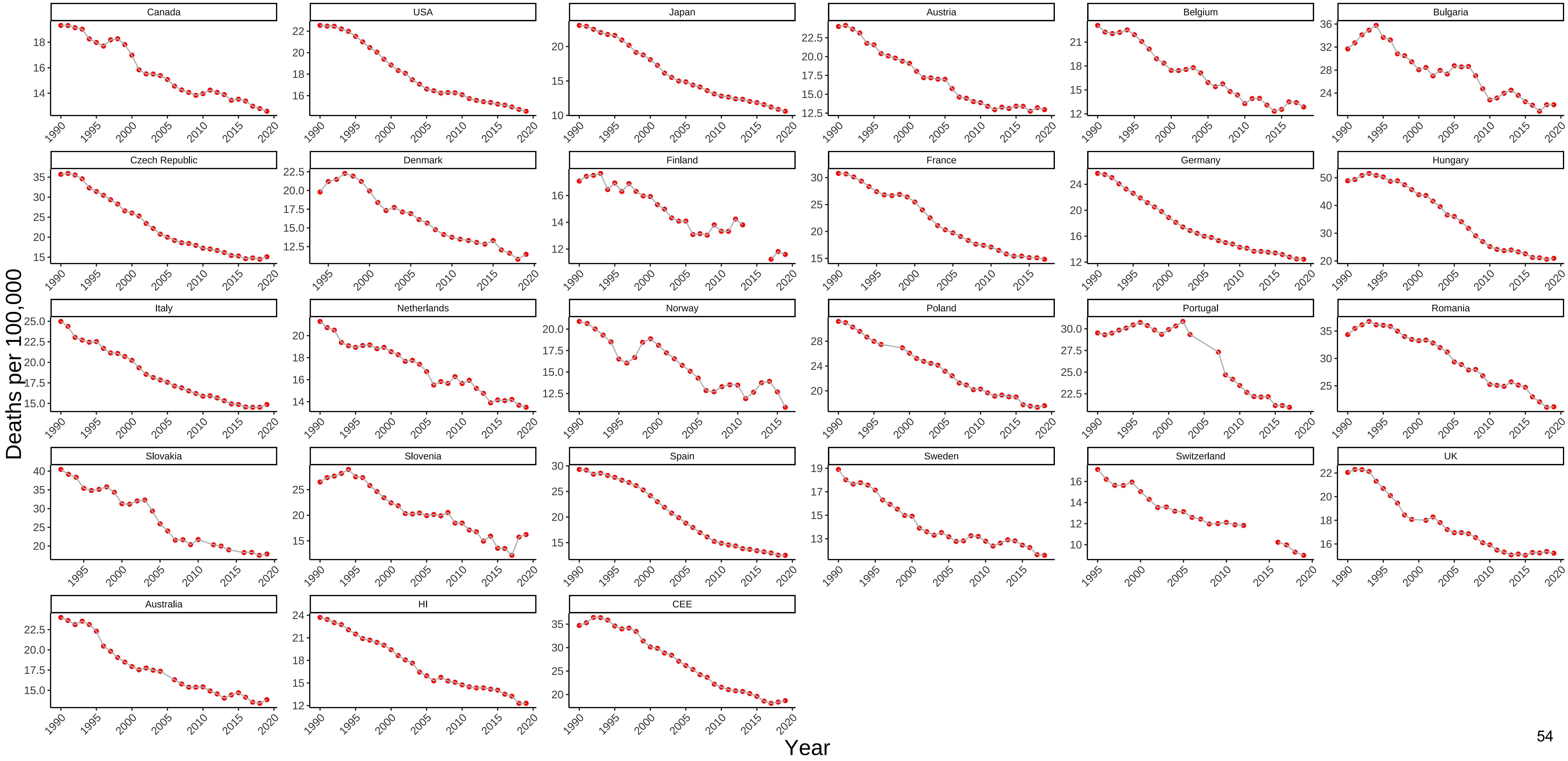

Figure S48. Three-Year Moving Average of Female Mortality from All Other Cancers at Ages 25-44

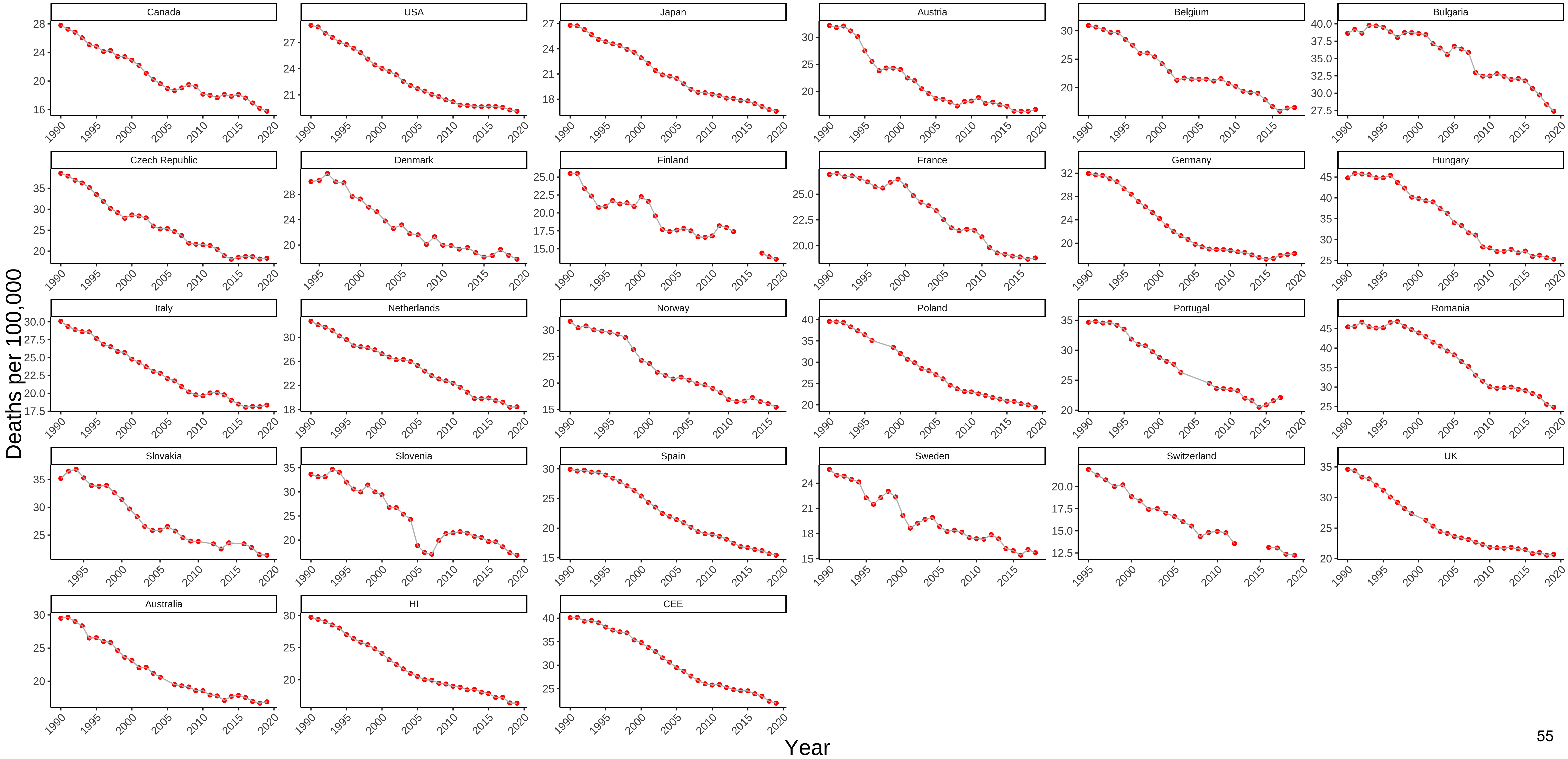

Figure S49. Three-Year Moving Average of Male Mortality from Nervous System Diseases at Ages 25-44

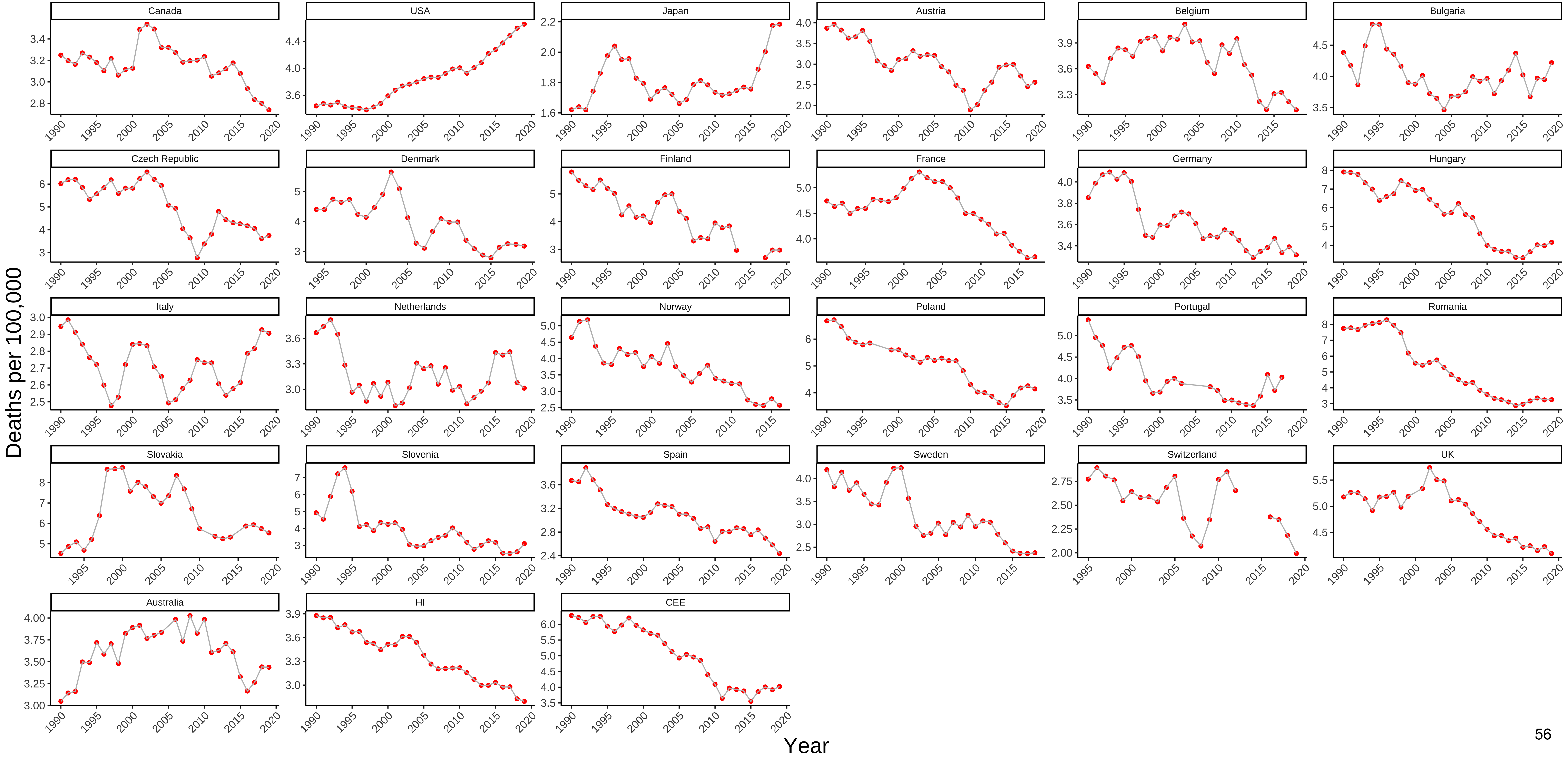

Figure S50. Three-Year Moving Average of Female Mortality from Nervous System Diseases at Ages 25-44

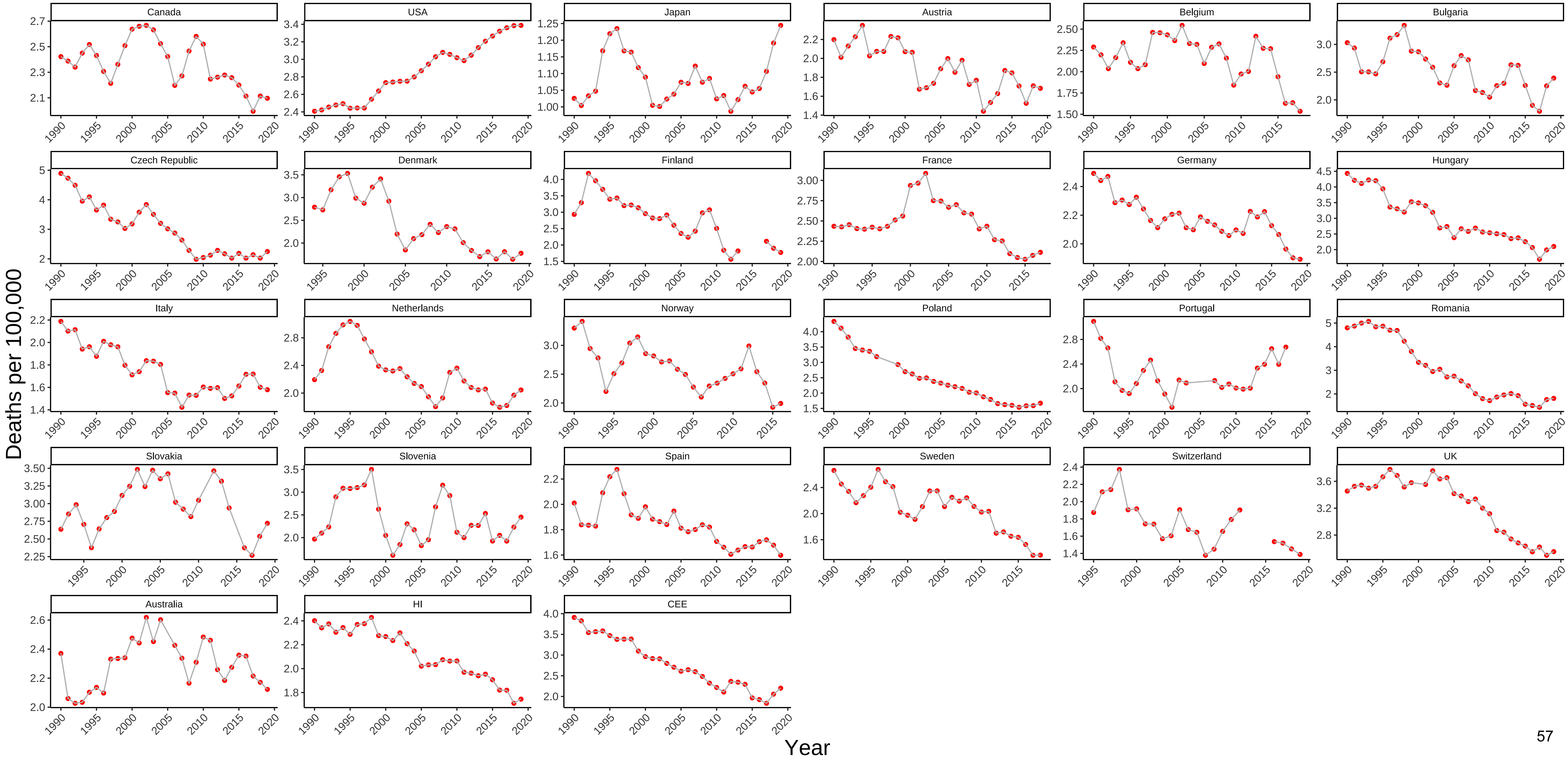

Figure S51. Three-Year Moving Average of Male Mortality from Metabolic Diseases at Ages 25-44

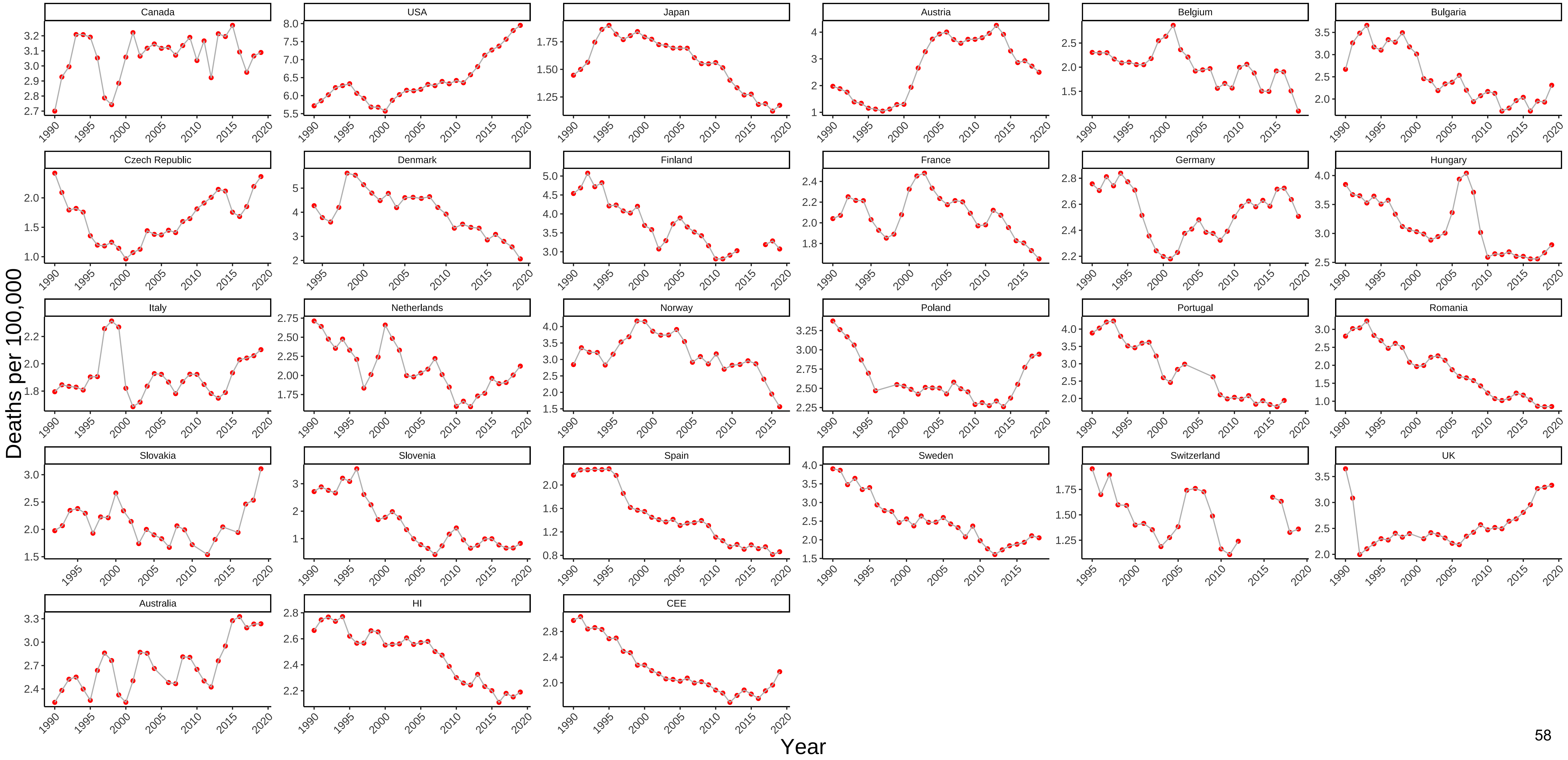

Figure S52. Three-Year Moving Average of Female Mortality from Metabolic Diseases at Ages 25-44

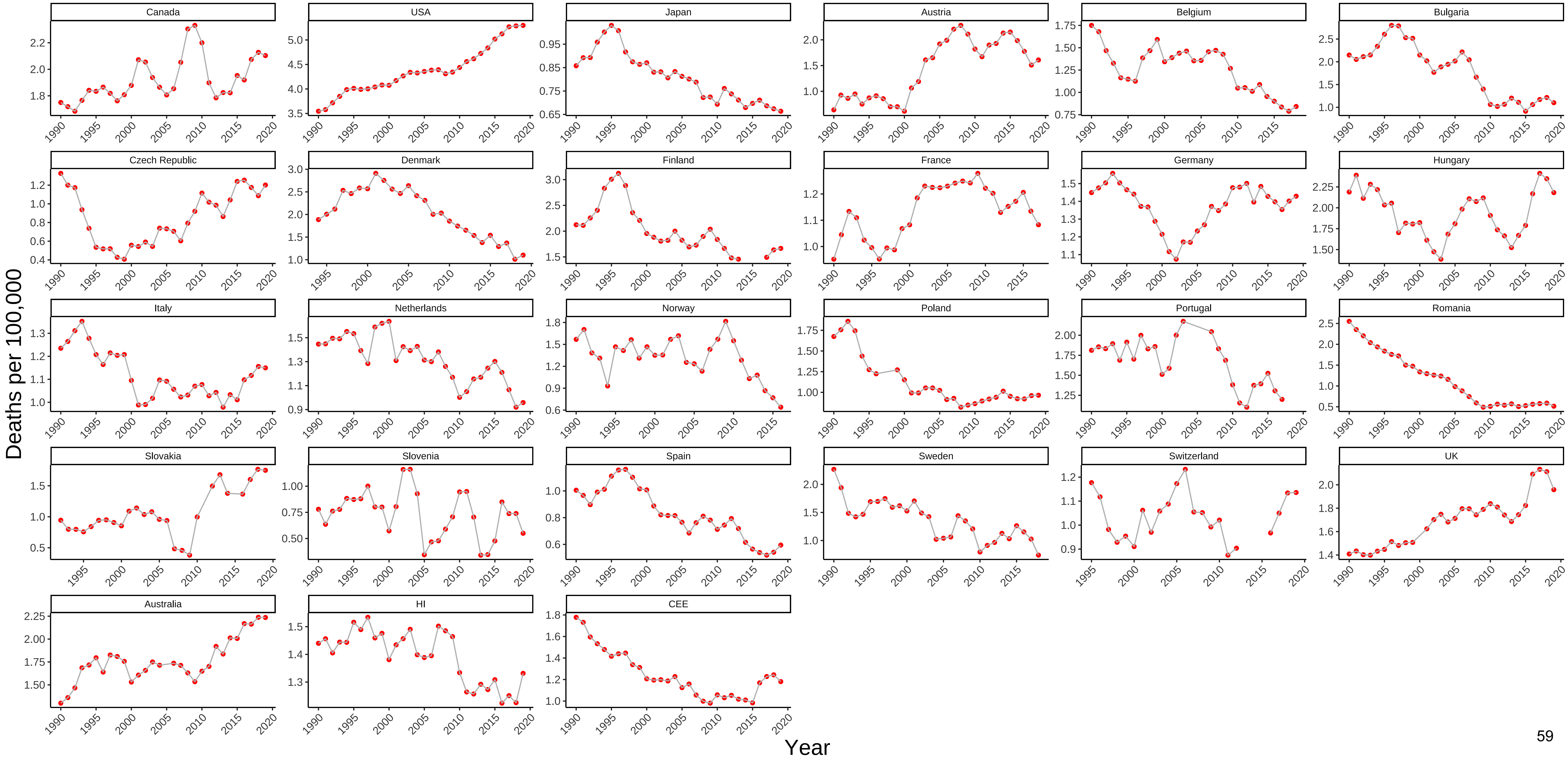

Figure S53. Three-Year Moving Average of Male Mortality from Cardiovascular Disease at Ages 25-44

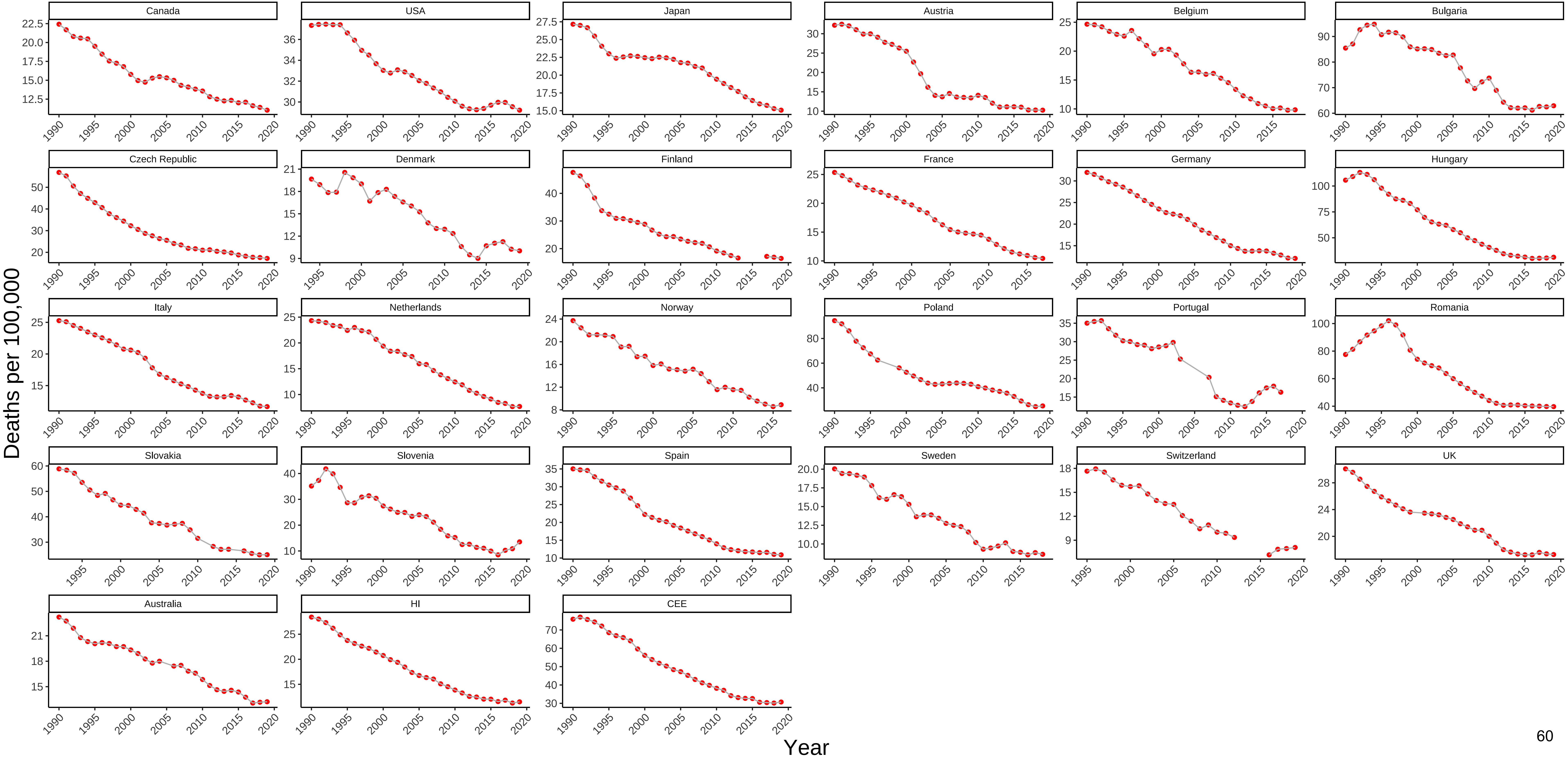

Figure S54. Three-Year Moving Average of Female Mortality from Cardiovascular Disease at Ages 25-44

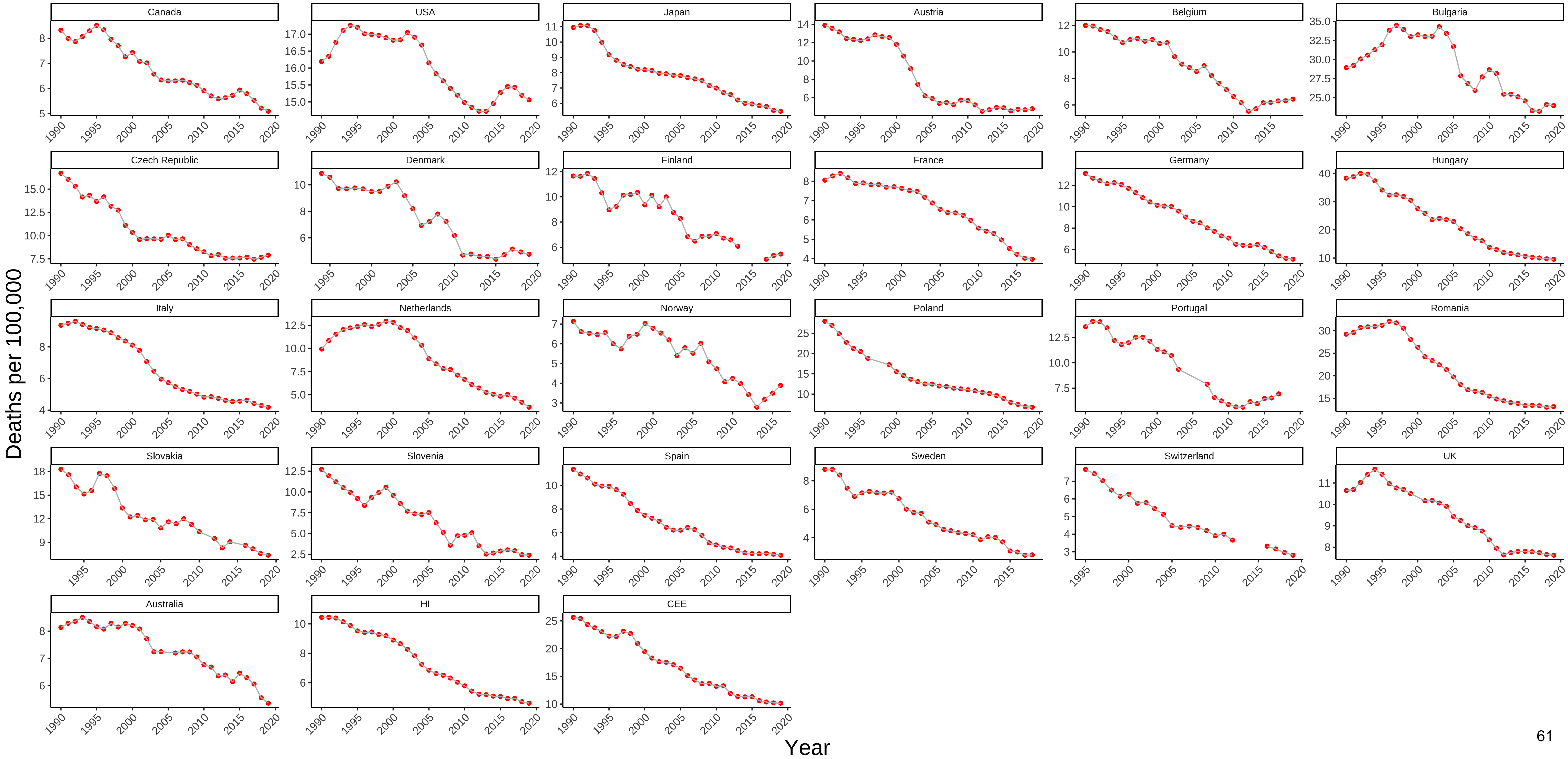

Figure S55. Three-Year Moving Average of Male Mortality from Suicide at Ages 25-44

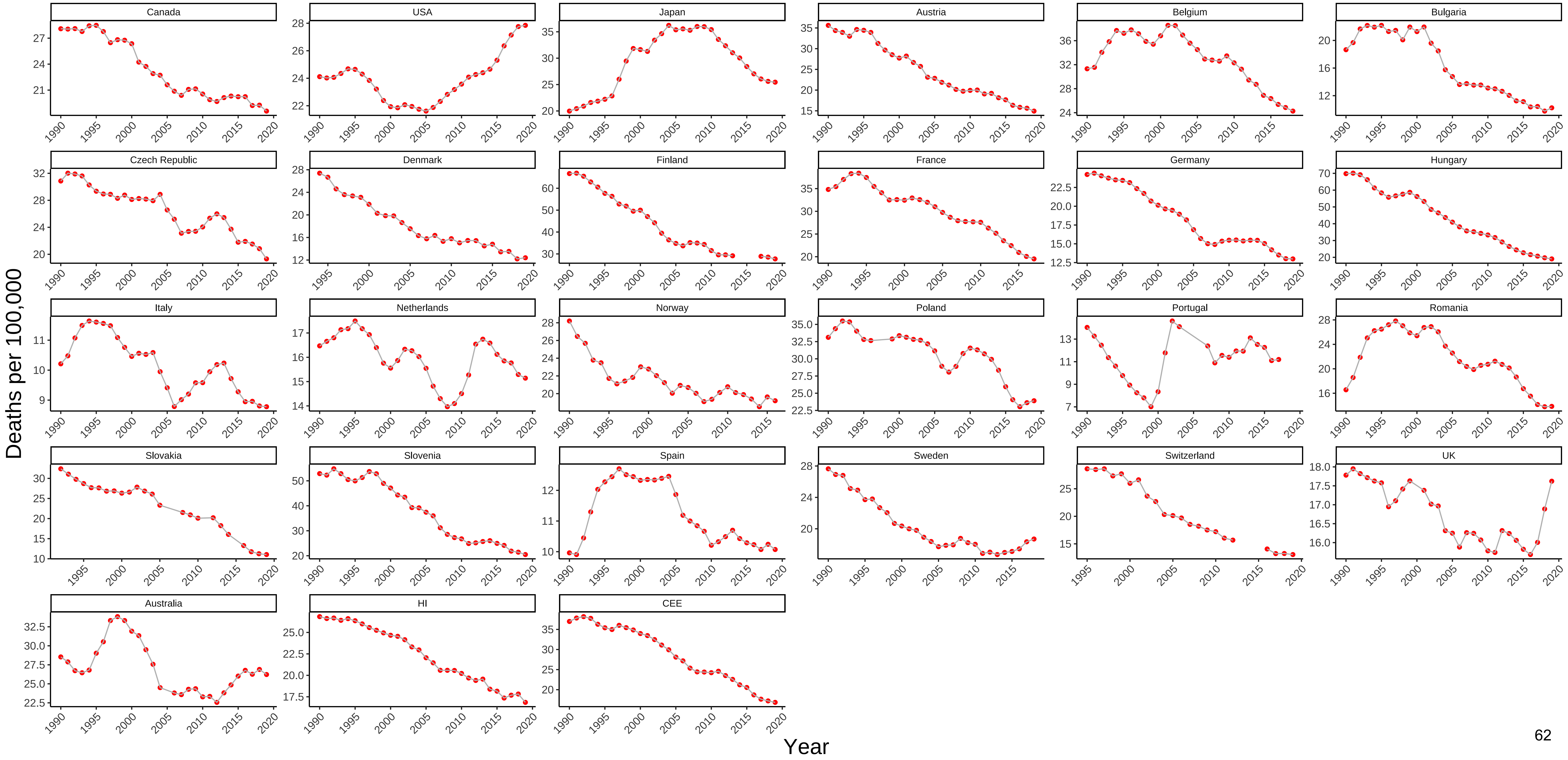

Figure S56. Three-Year Moving Average of Female Mortality from Suicide at Ages 25-44

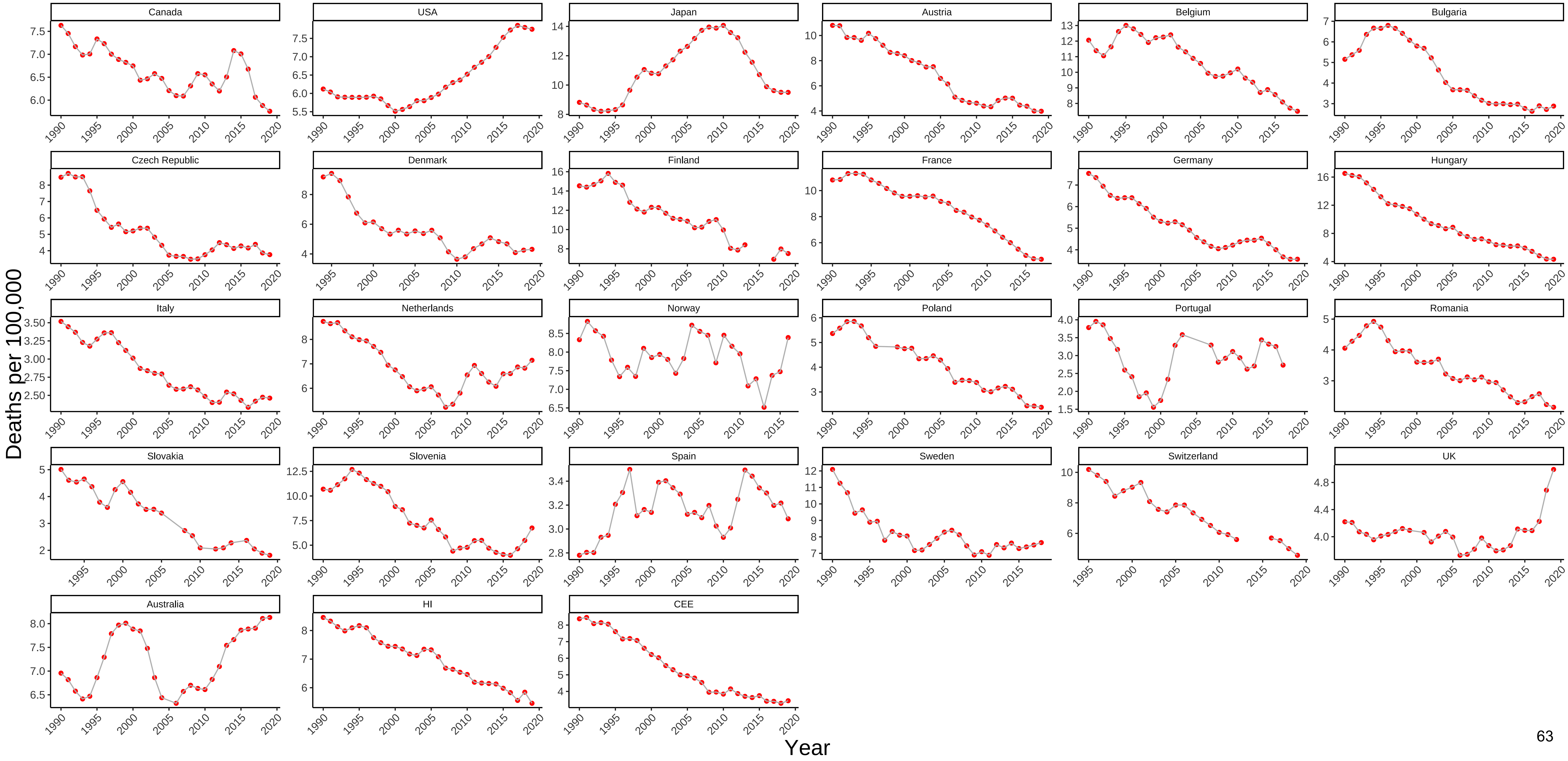

Figure S57. Three-Year Moving Average of Male Mortality from Homicide at Ages 25-44

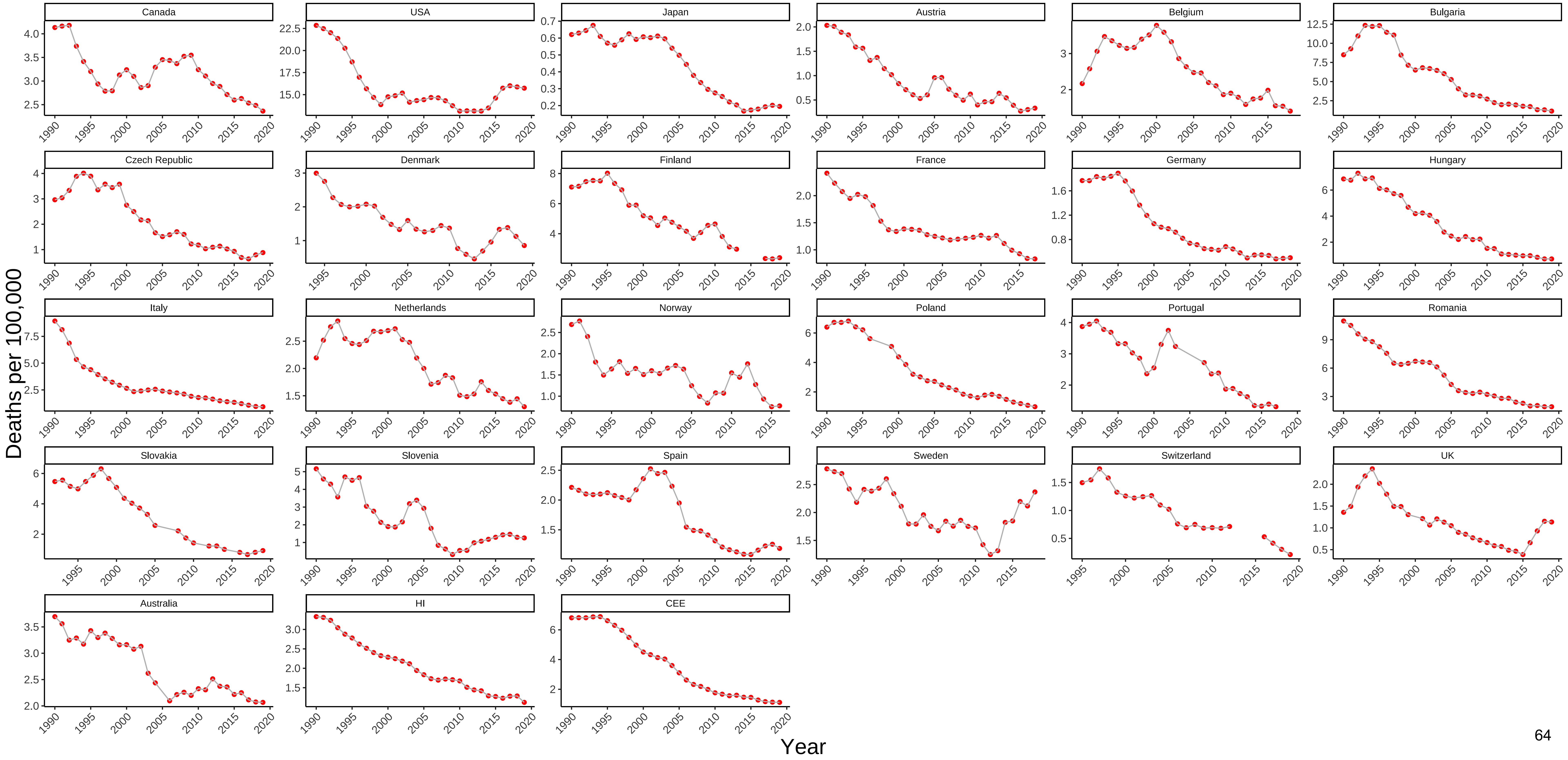

Figure S58. Three-Year Moving Average of Female Mortality from Homicide at Ages 25-44

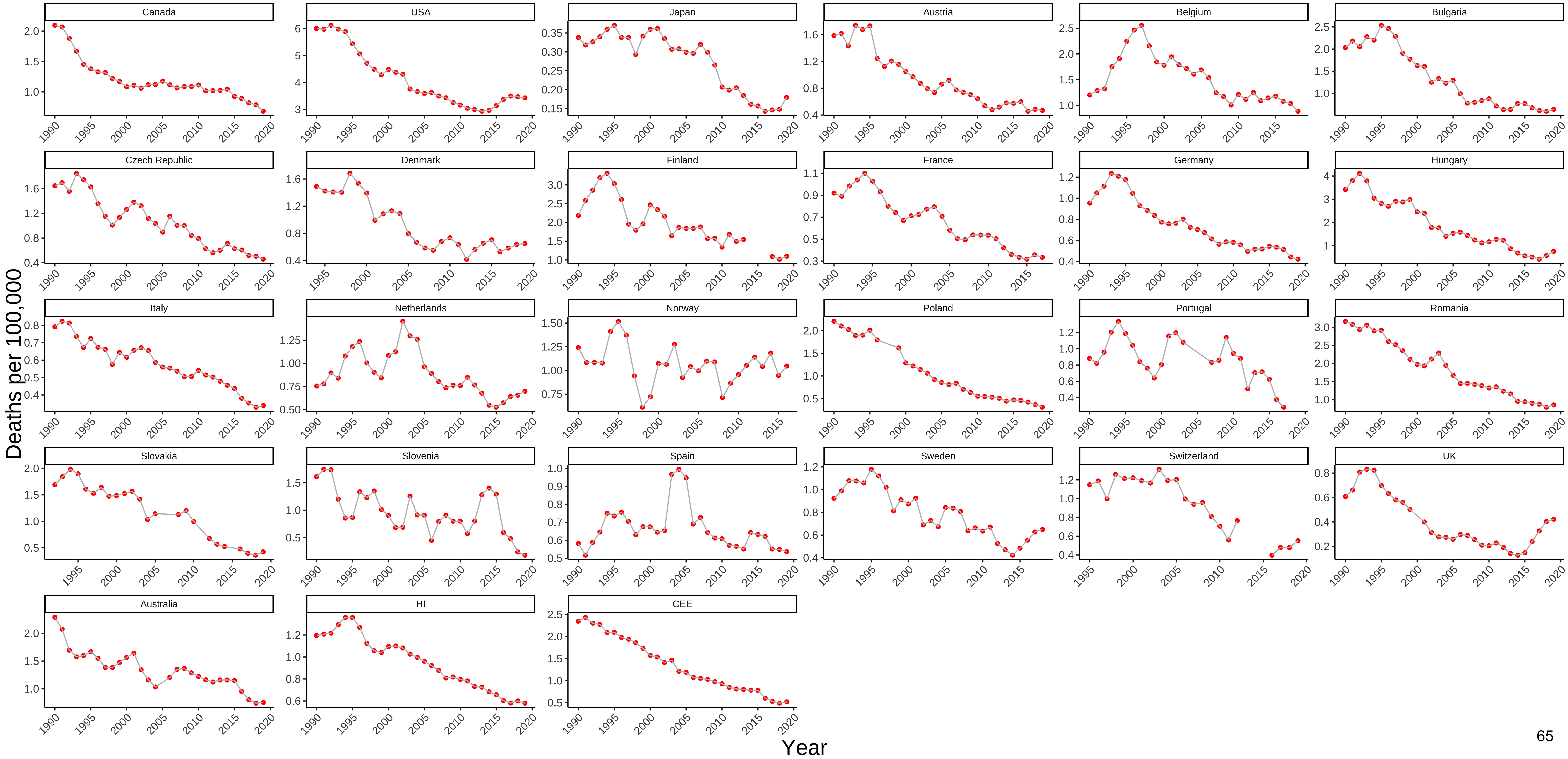

Figure S59. Three-Year Moving Average of Male Mortality from Transport Accidents at Ages 25-44

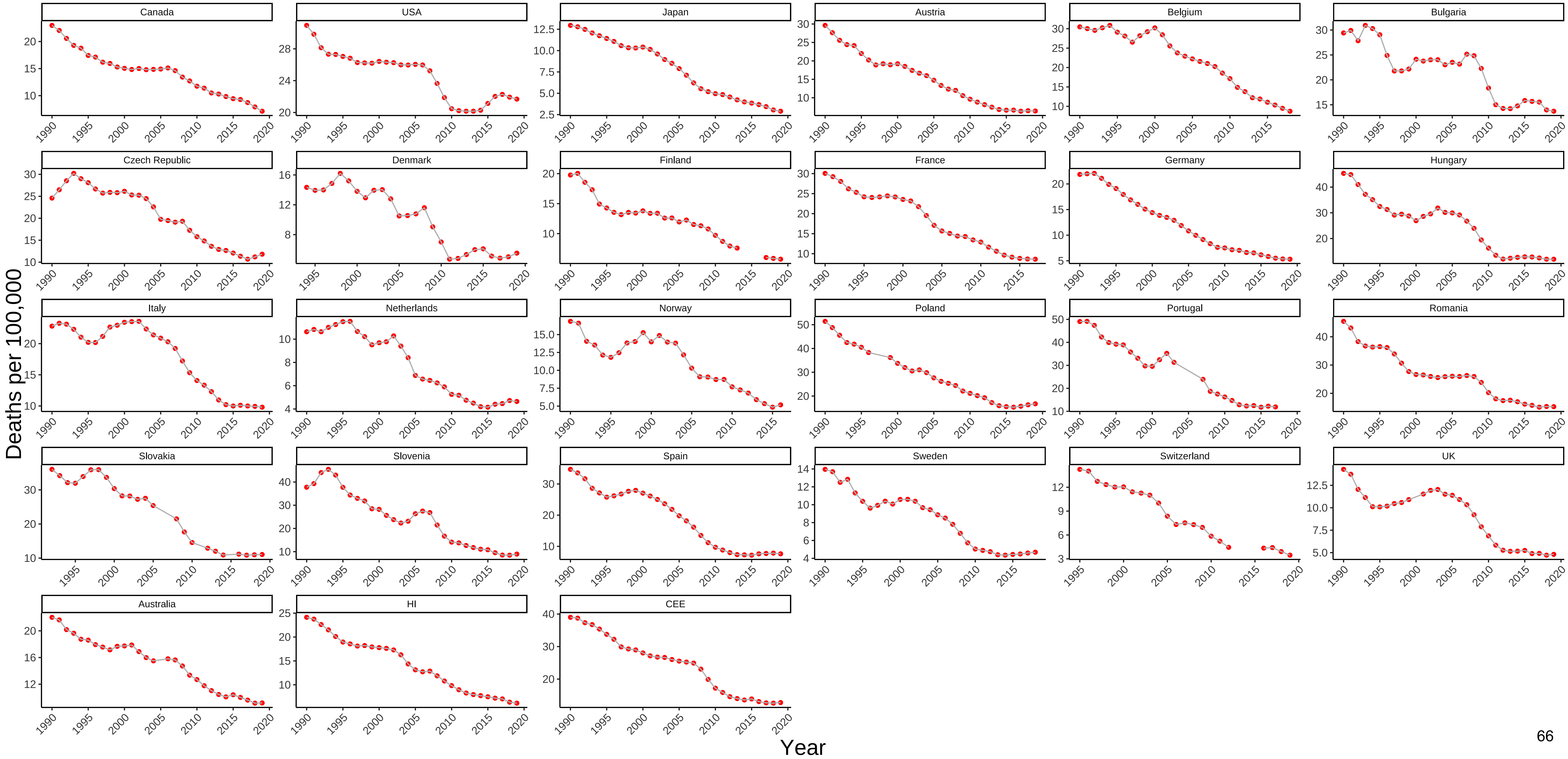

Figure S60. Three-Year Moving Average of Female Mortality from Transport Accidents at Ages 25-44

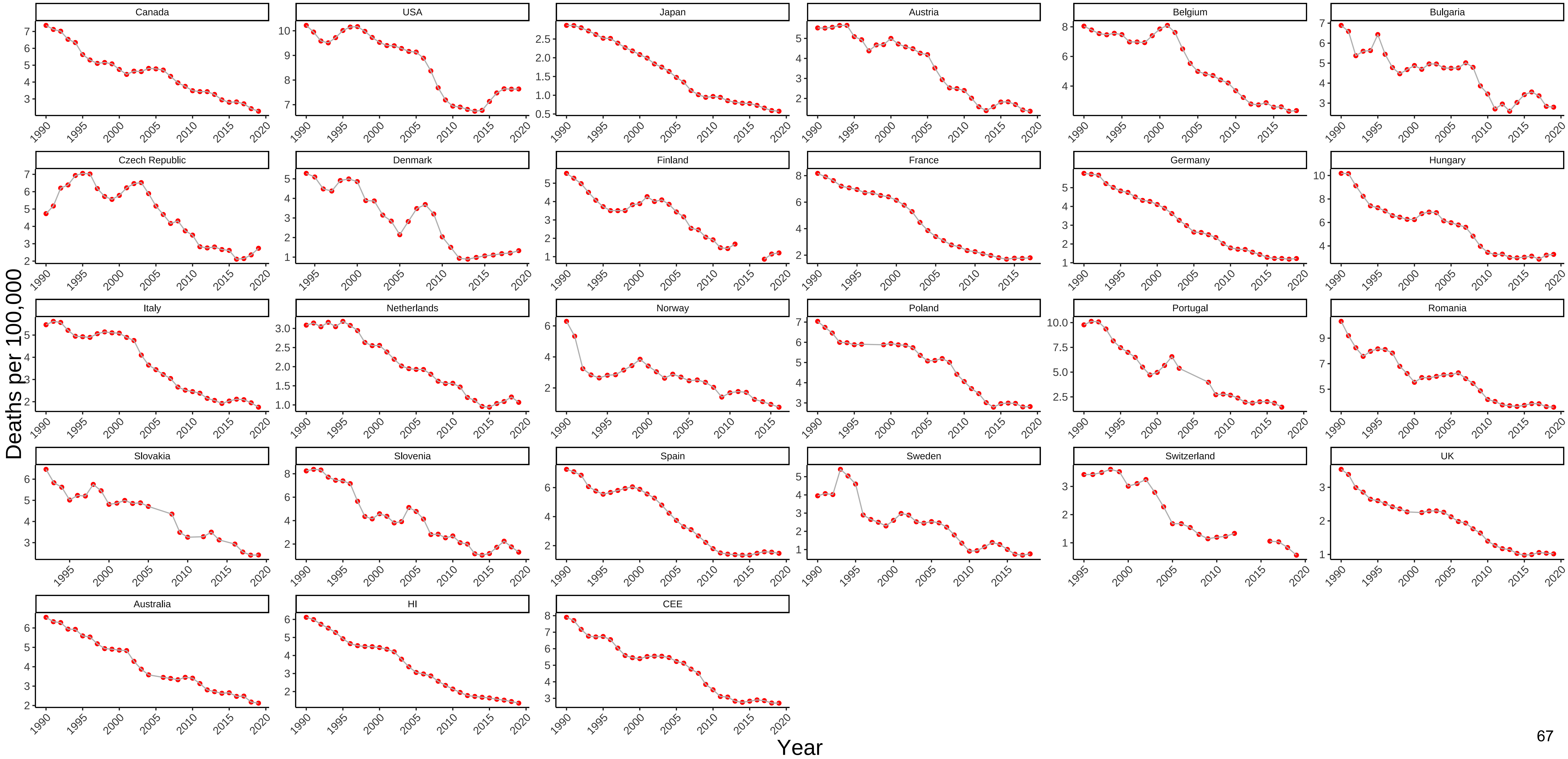

Figure S61. Three-Year Moving Average of Male Mortality from Other External Causes at Ages 25-44

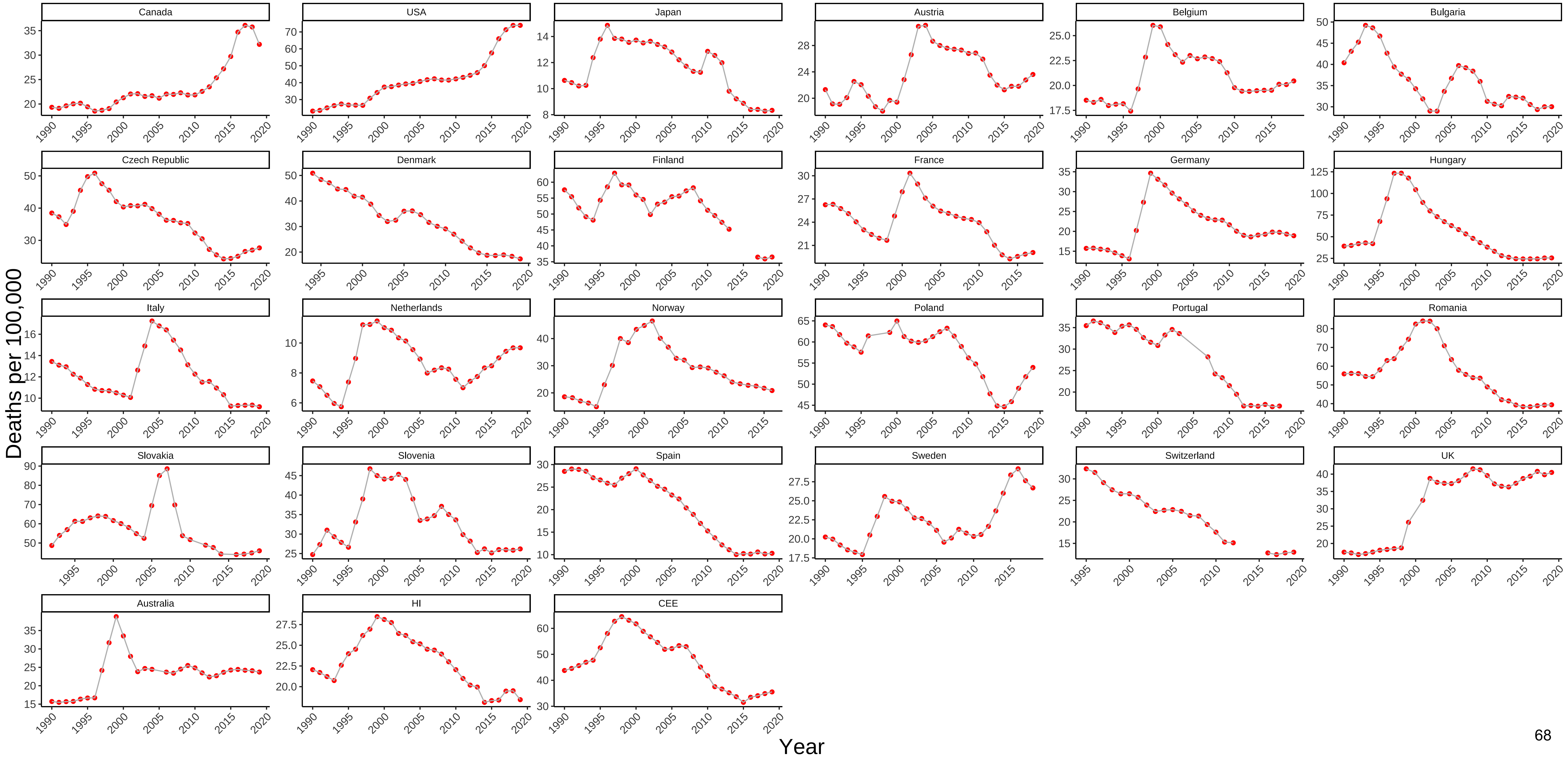

Figure S62. Three-Year Moving Average of Female Mortality from Other External Causes at Ages 25-44

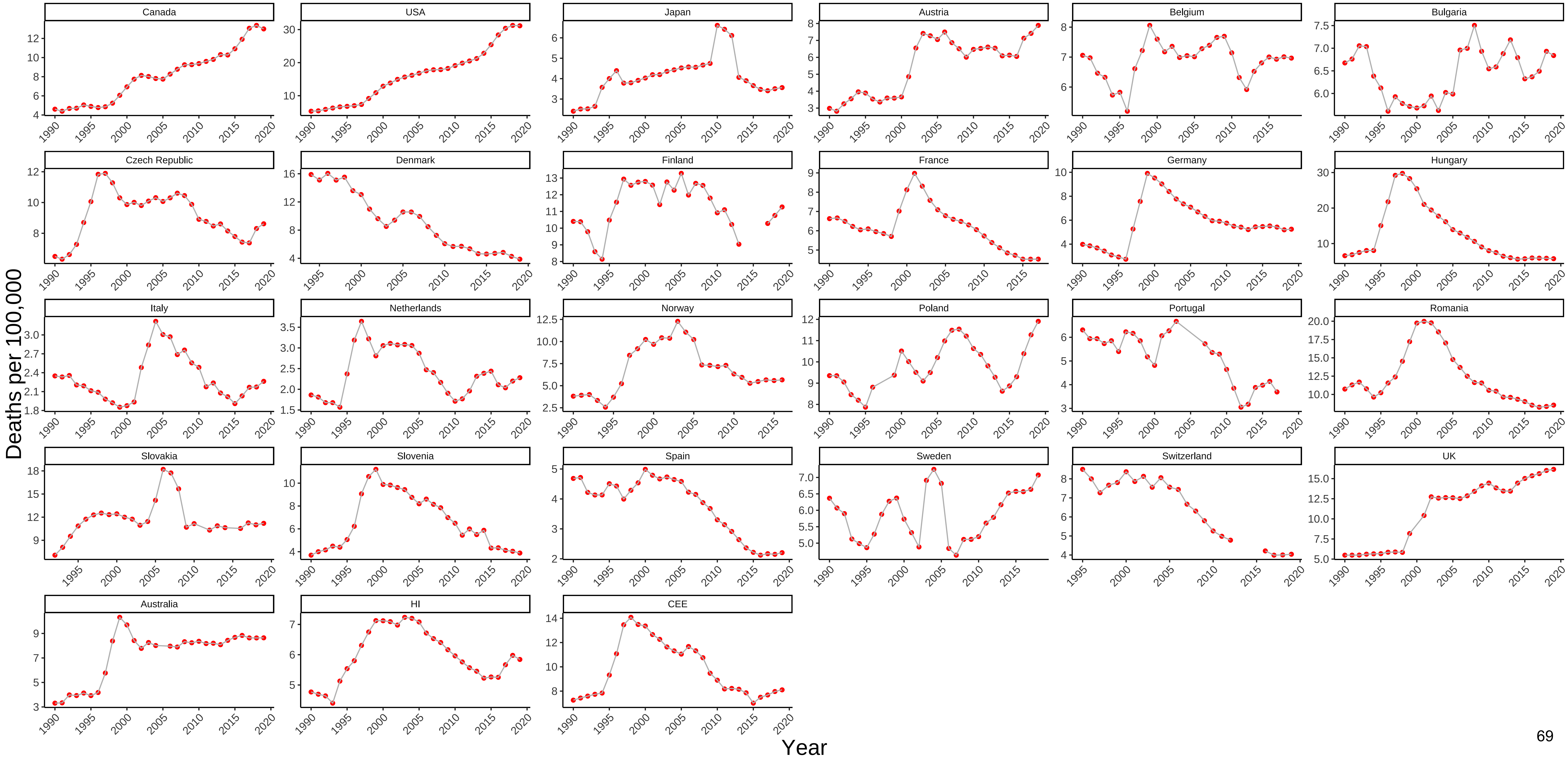

Figure S63. Three-Year Moving Average of Male Mortality from All Other Causes at Ages 25-44

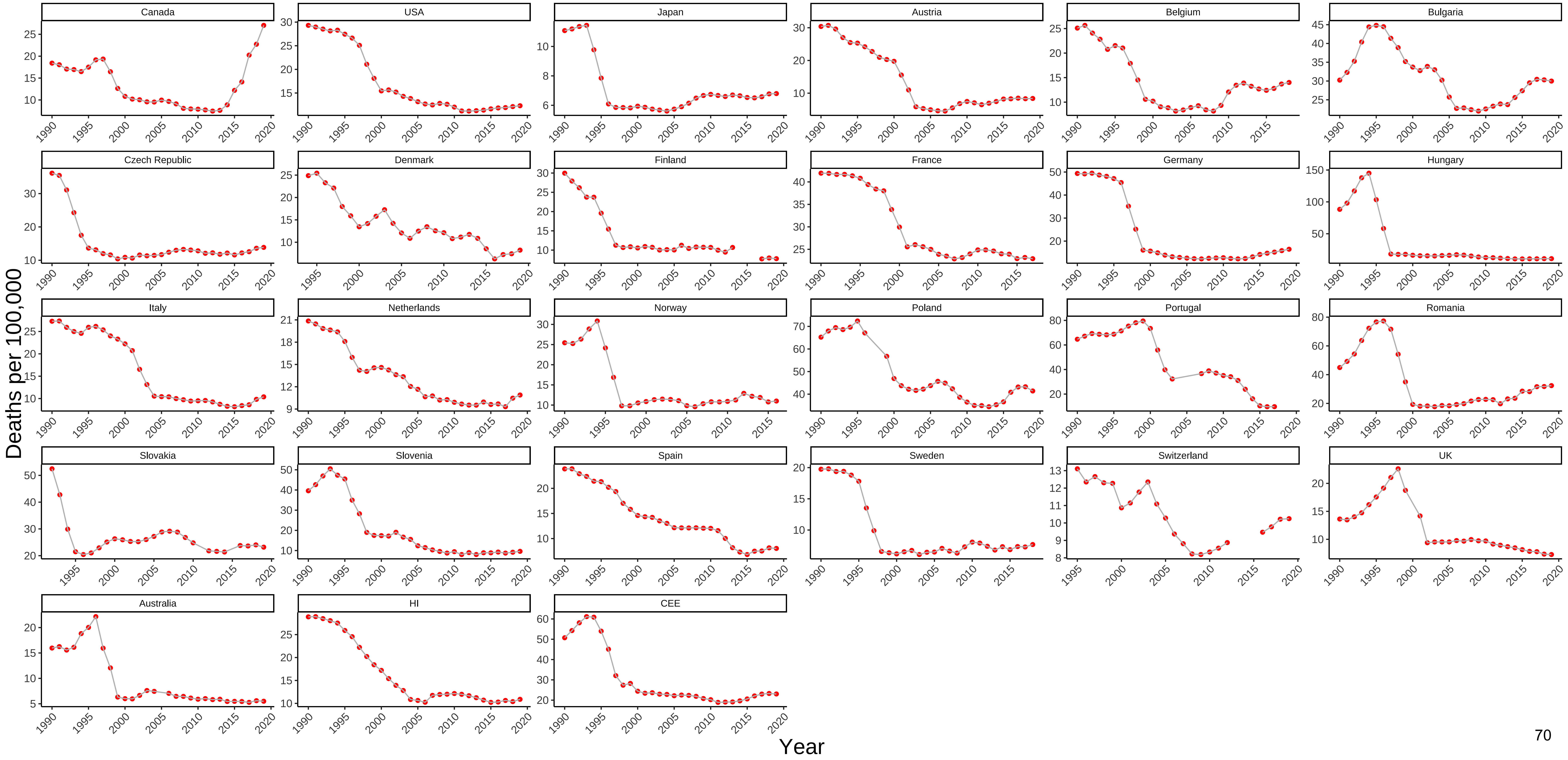

Figure S64. Three-Year Moving Average of Female Mortality from All Other Causes at Ages 25-44

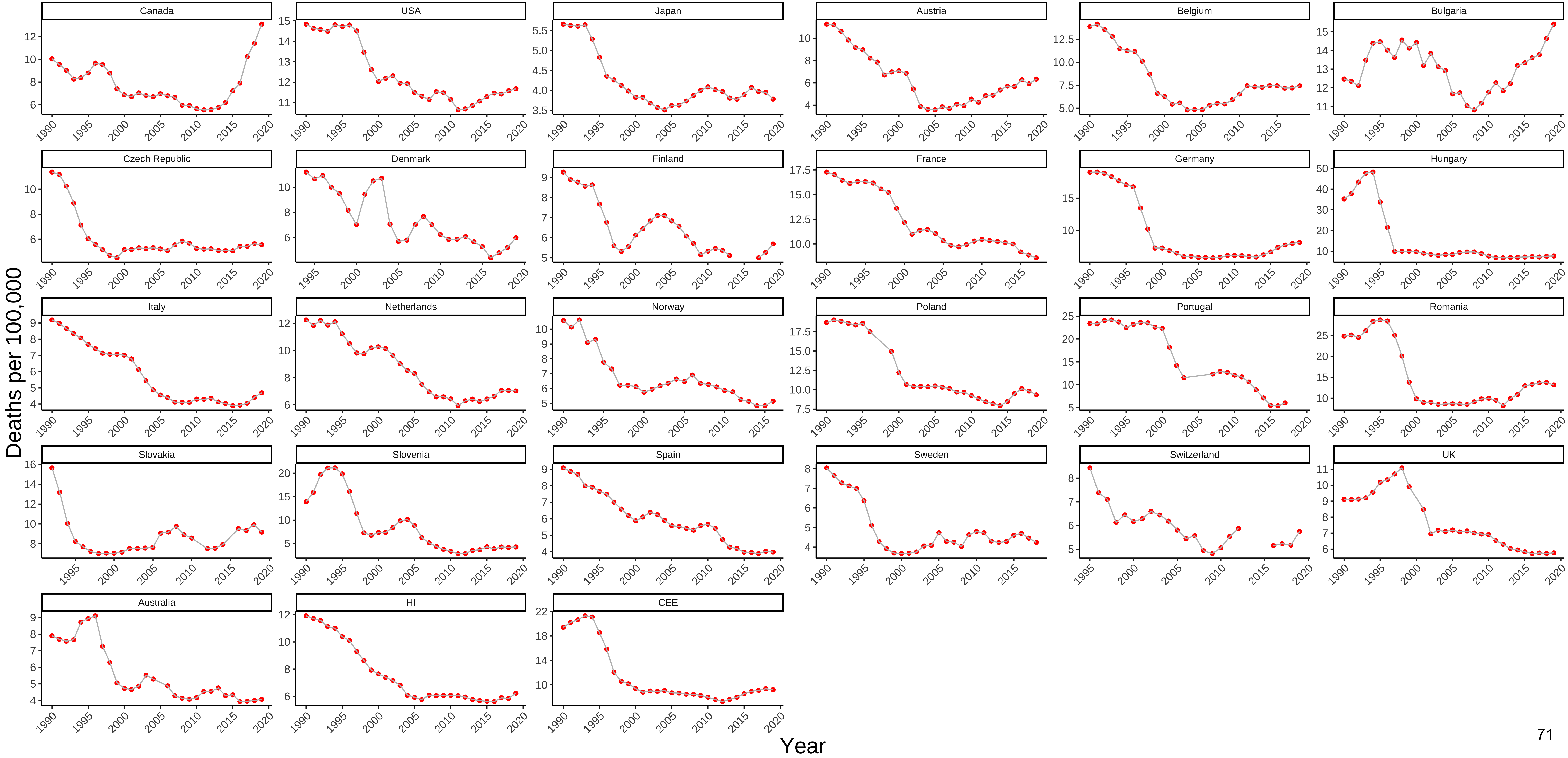

Figure S65. Three-Year Moving Average of Male Mortality from All Causes at Ages 25-44

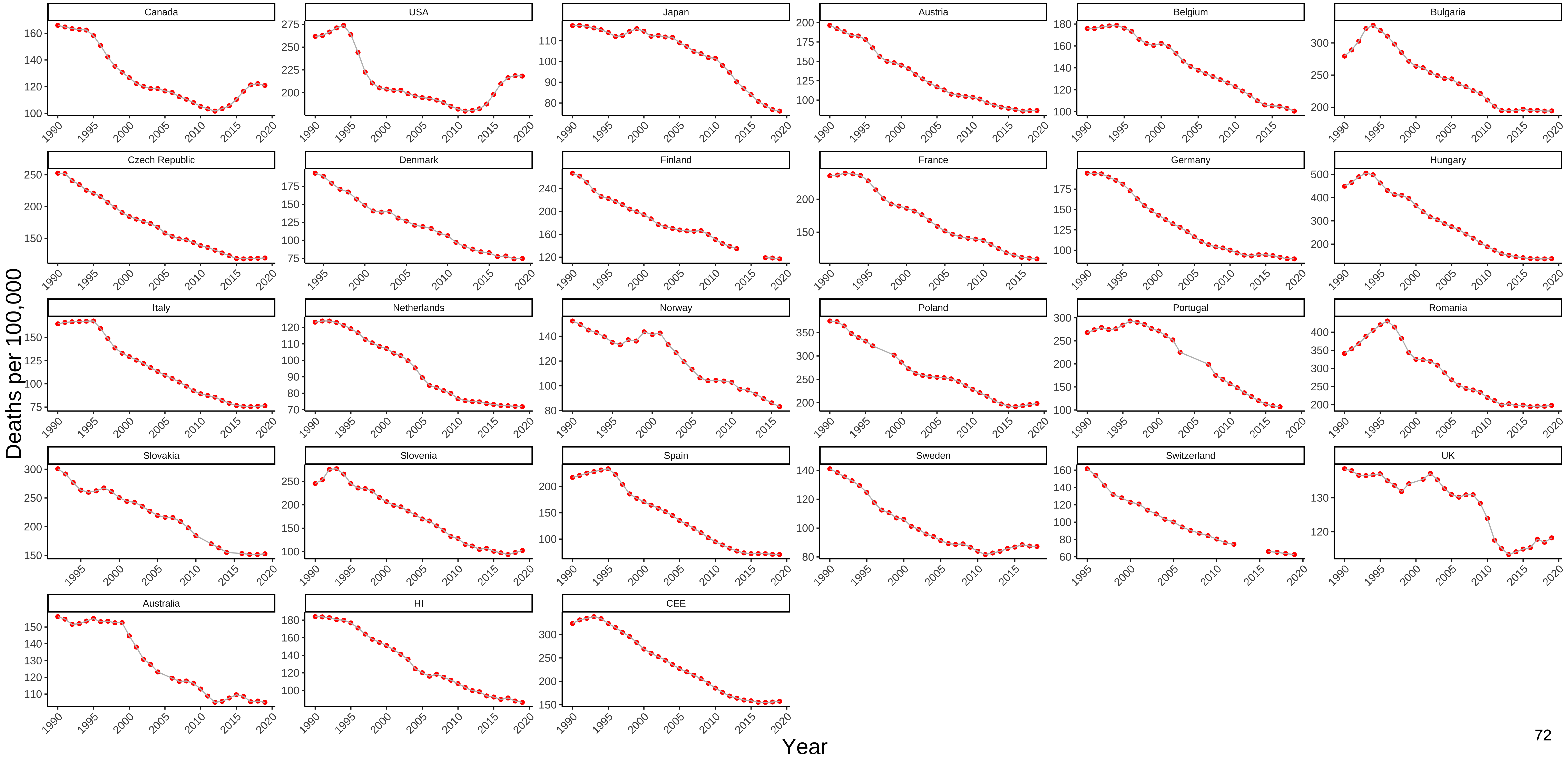

Figure S66. Three-Year Moving Average of Female Mortality from All Causes at Ages 25-44

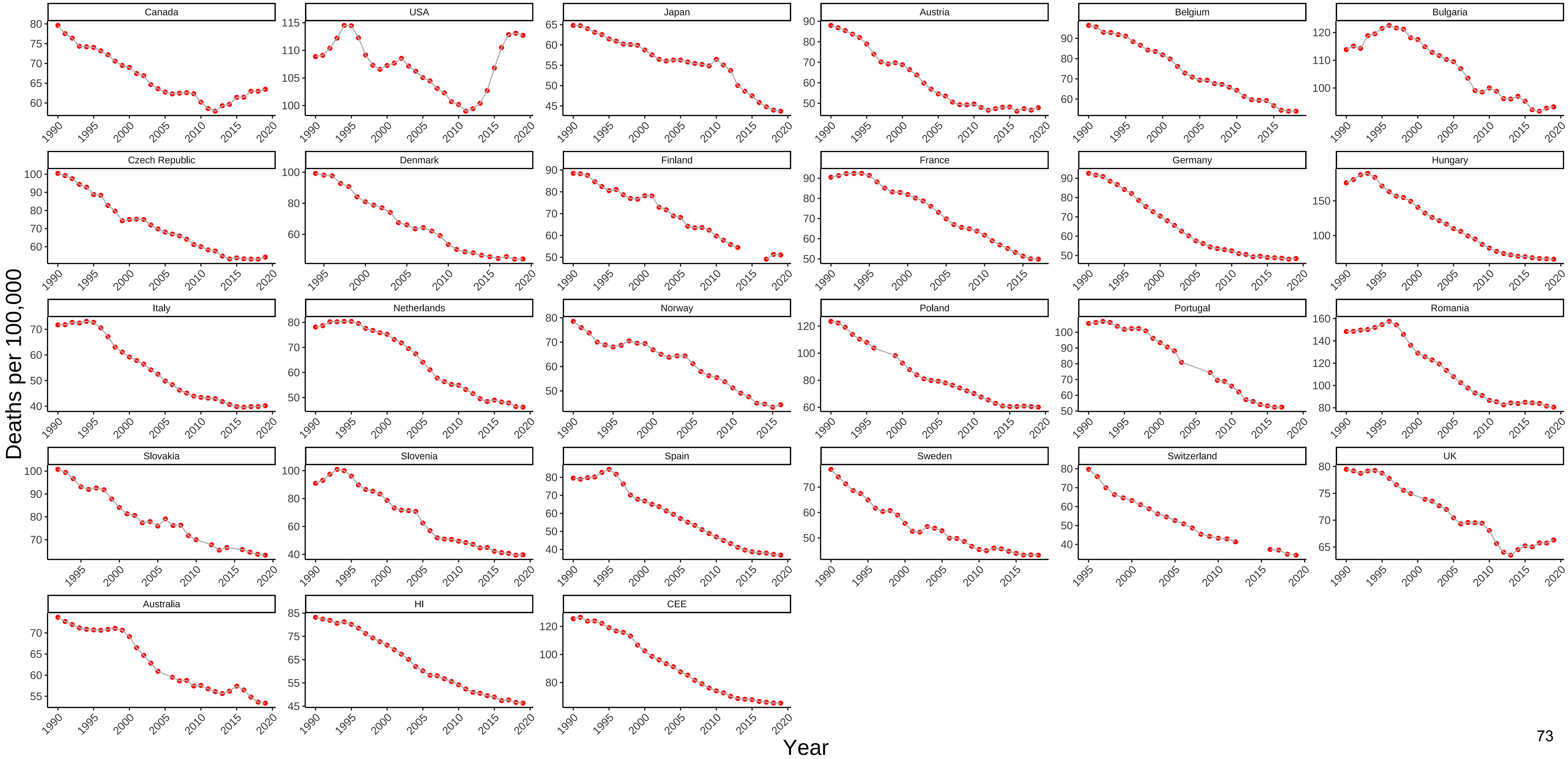

Figure S67. Three-Year Moving Average of Male Mortality from Infectious and Parasitic Diseases at Ages 45-54

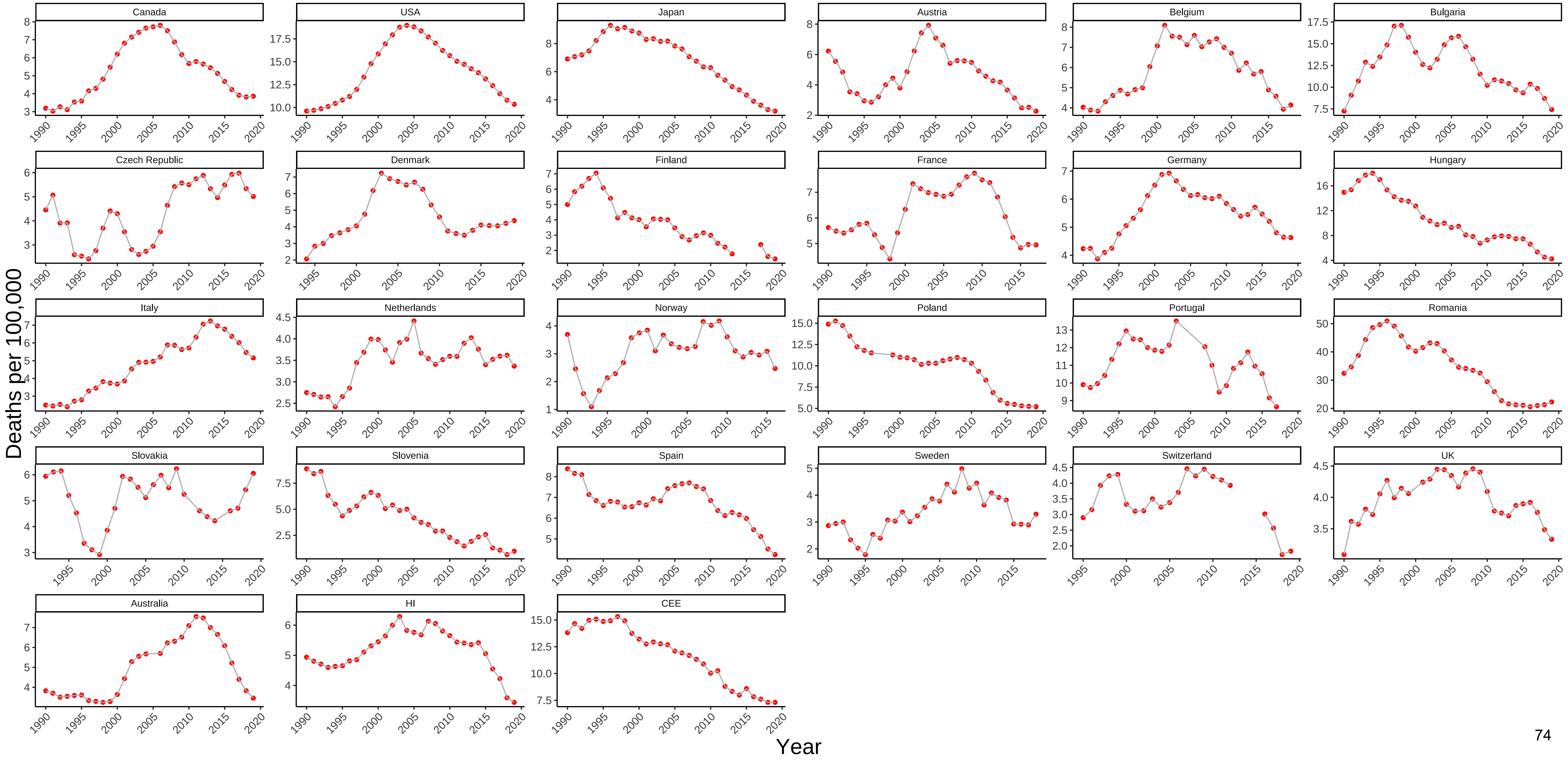

Figure S68. Three-Year Moving Average of Female Mortality from Infectious and Parasitic Diseases at Ages 45-54

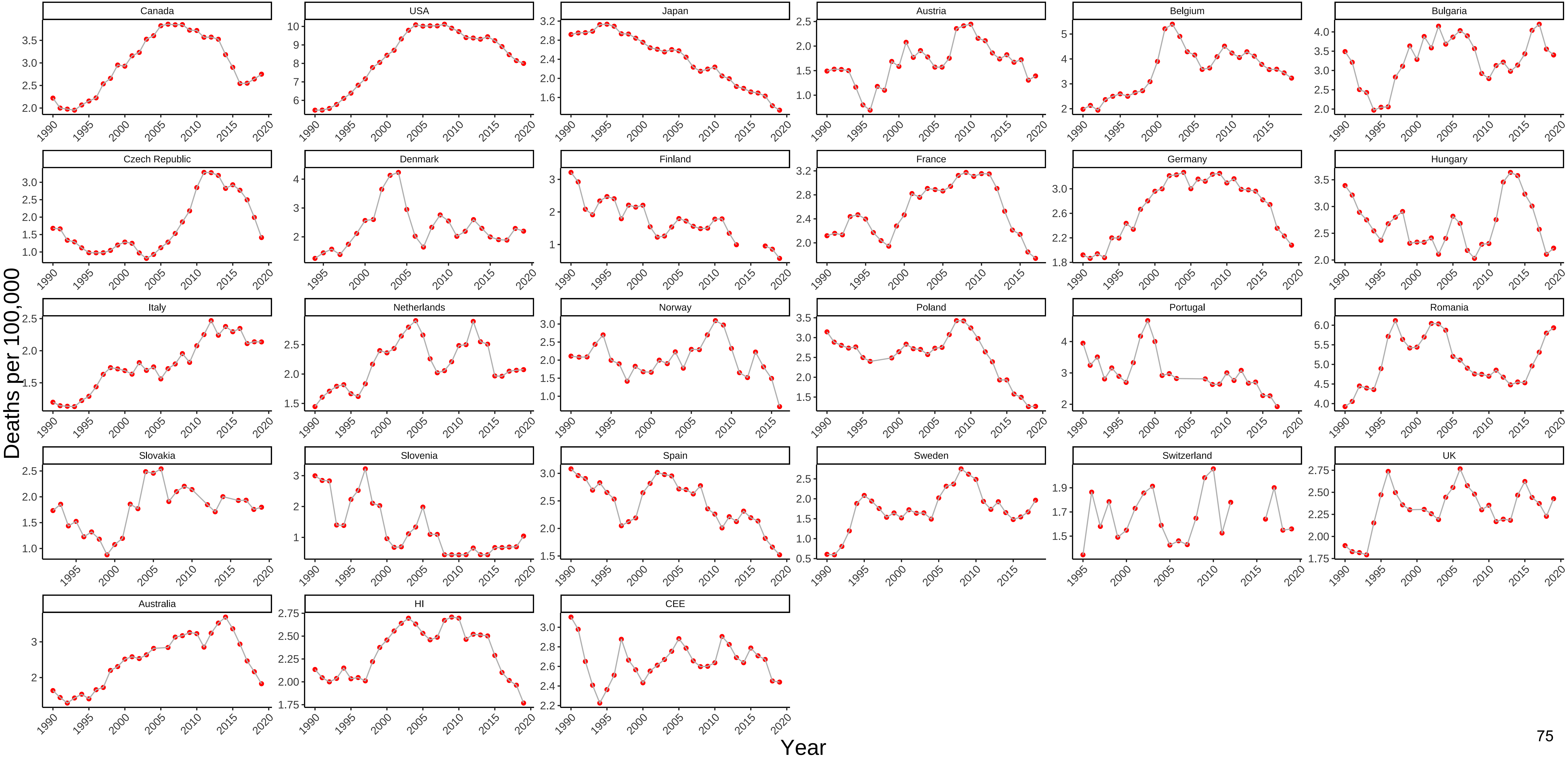

Figure S69. Three-Year Moving Average of Male Mortality from HIV/AIDS at Ages 45-54

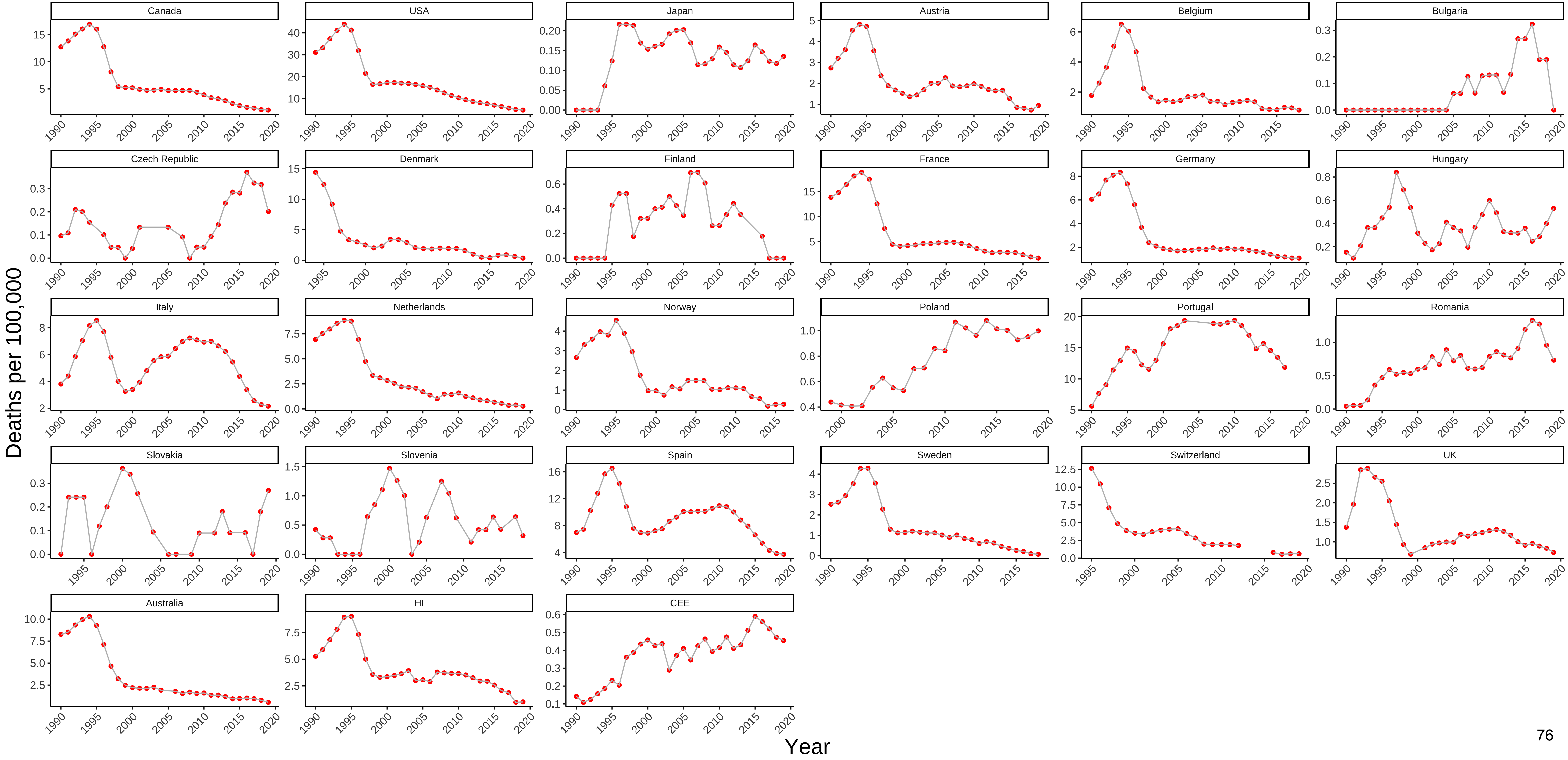

Figure S70. Three-Year Moving Average of Female Mortality from HIV/AIDS at Ages 45-54

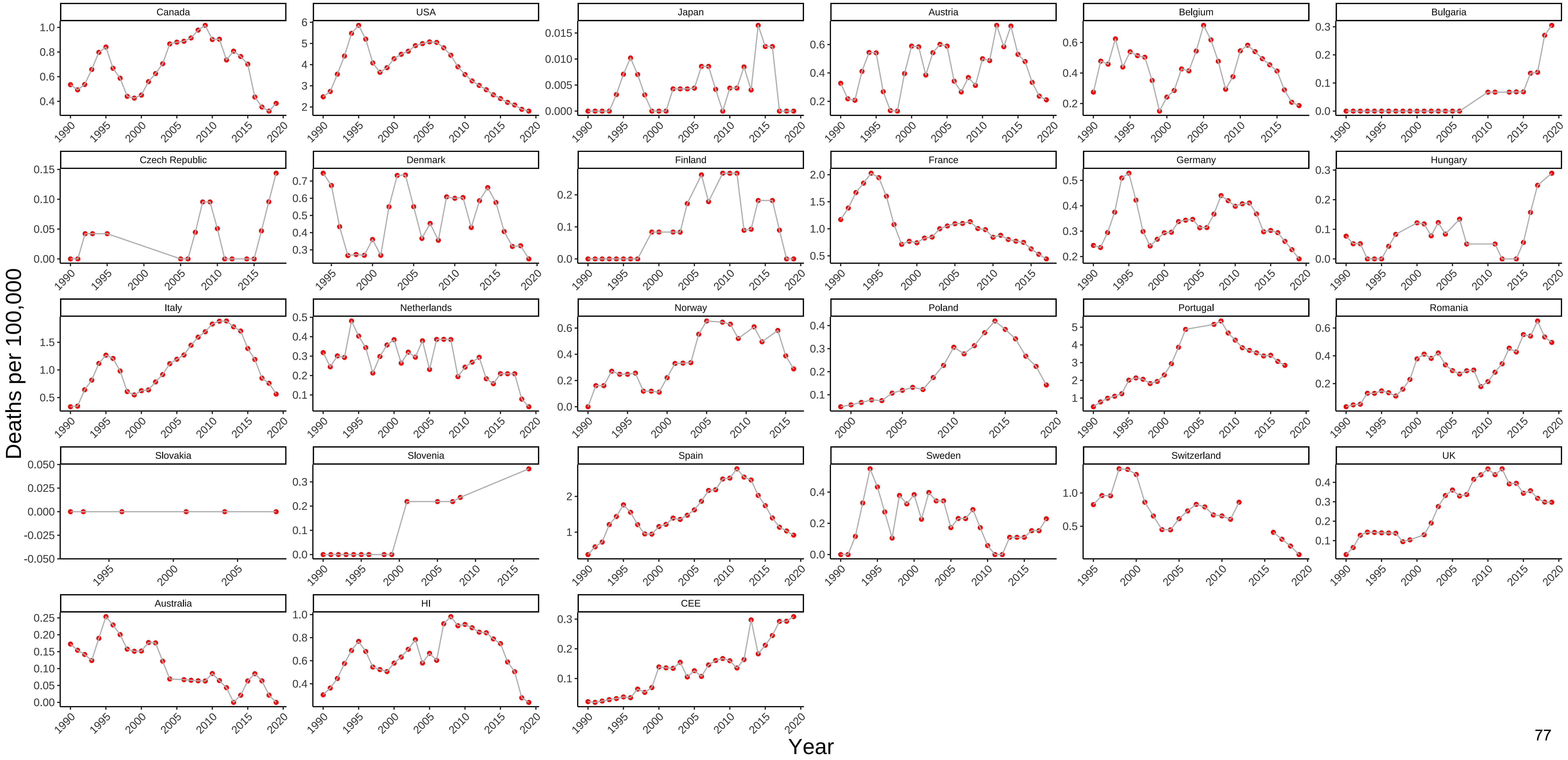

Figure S71. Three-Year Moving Average of Male Mortality from Respiratory Diseases at Ages 45-54

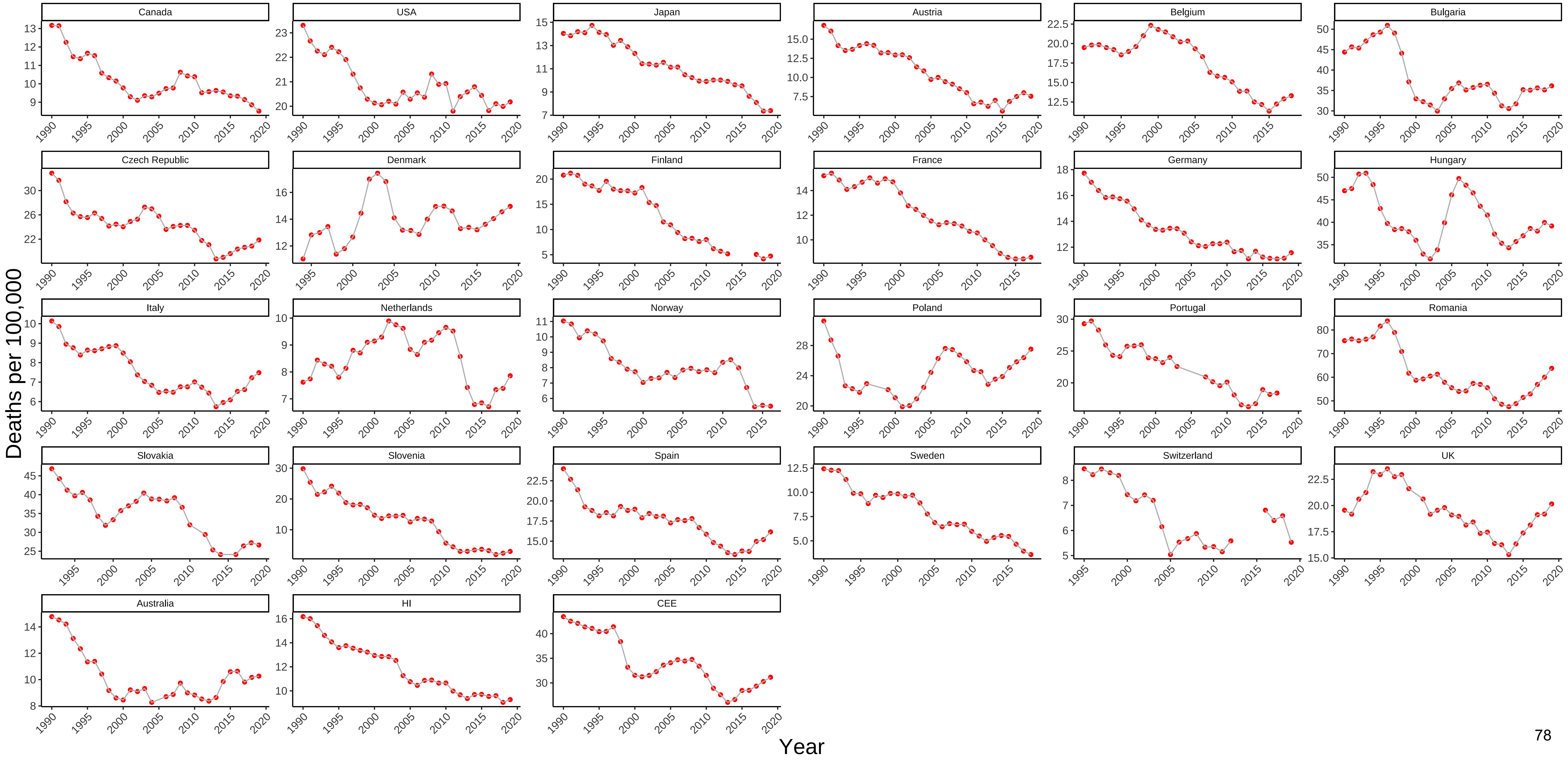

Figure S72. Three-Year Moving Average of Female Mortality from Respiratory Diseases at Ages 45-54

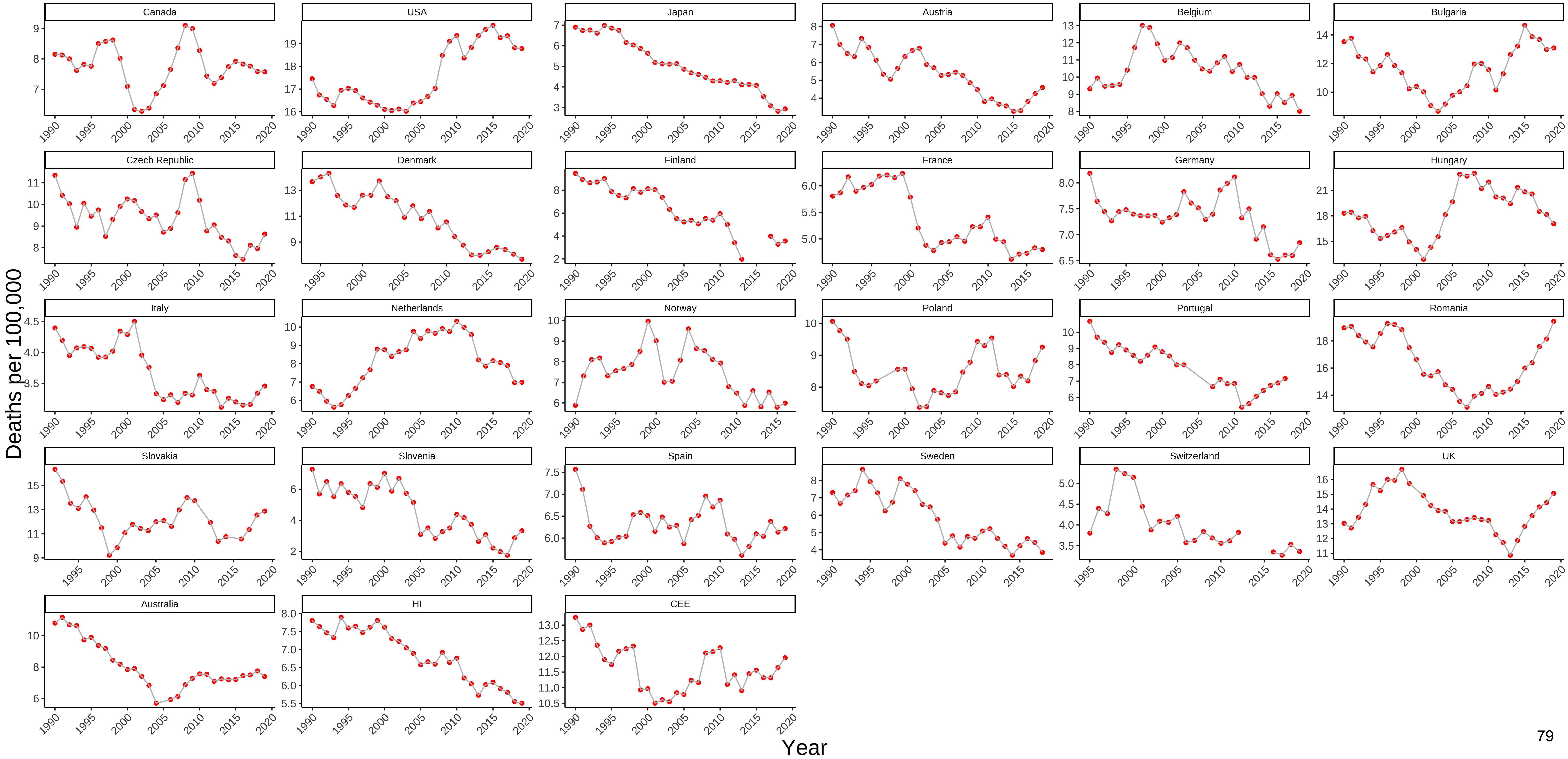

Figure S73. Three-Year Moving Average of Male Mortality from Trachea/Bronchus, Lung Cancers at Ages 45-54

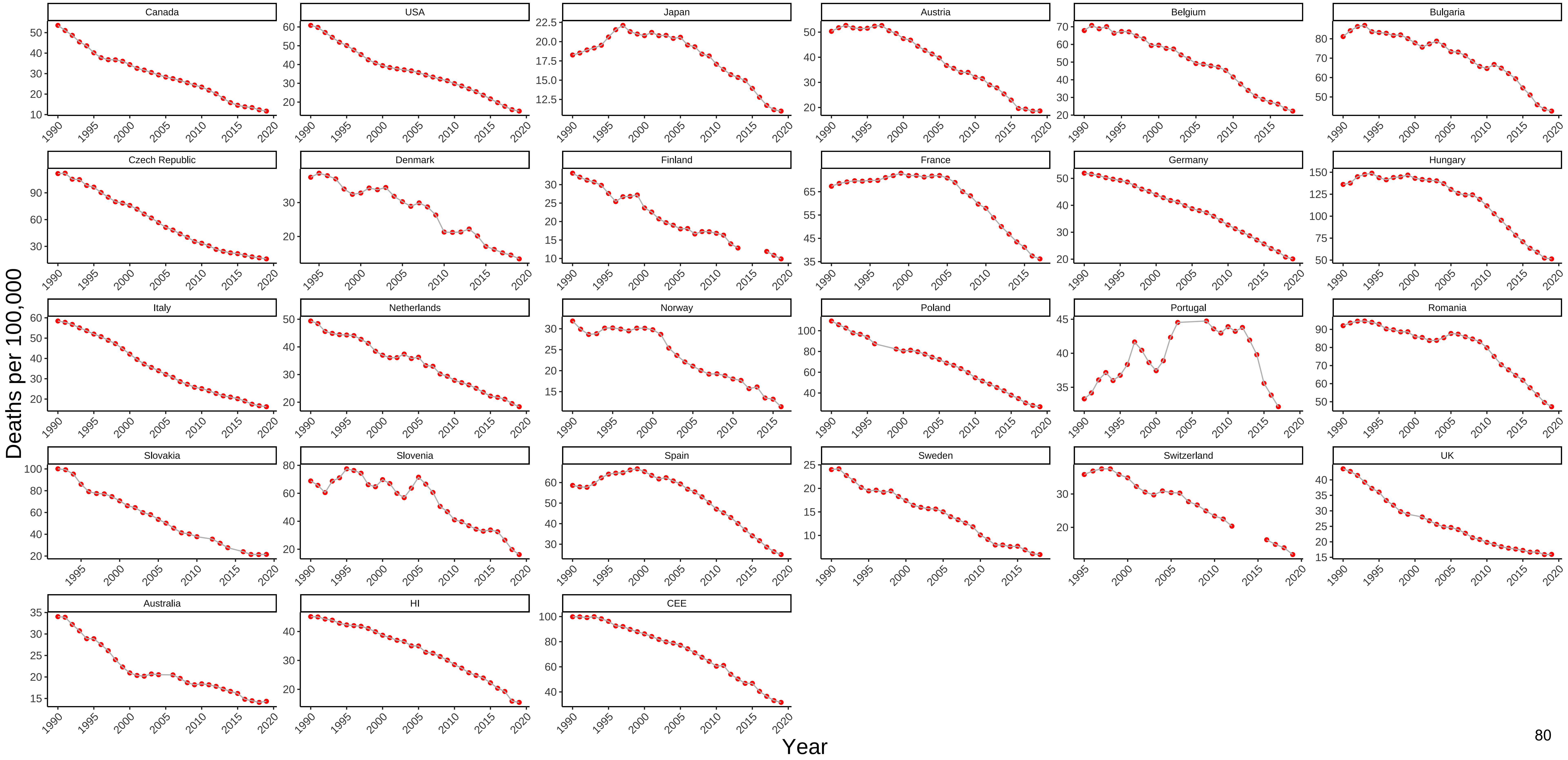

Figure S74. Three-Year Moving Average of Female Mortality from Trachea/Bronchus, Lung Cancers at Ages 45-54

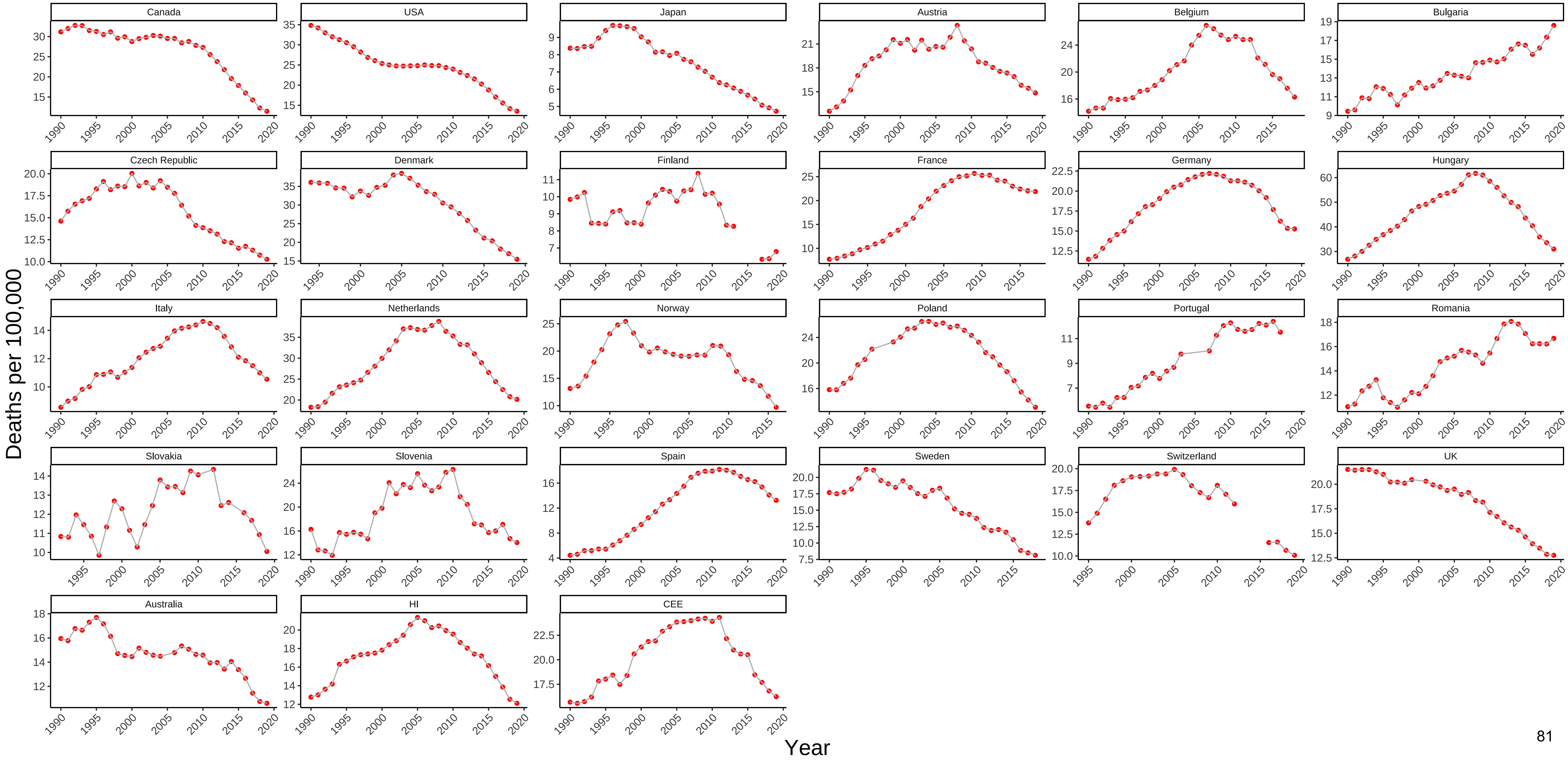

Figure S75. Three-Year Moving Average of Male Mortality from All Other Cancers at Ages 45-54

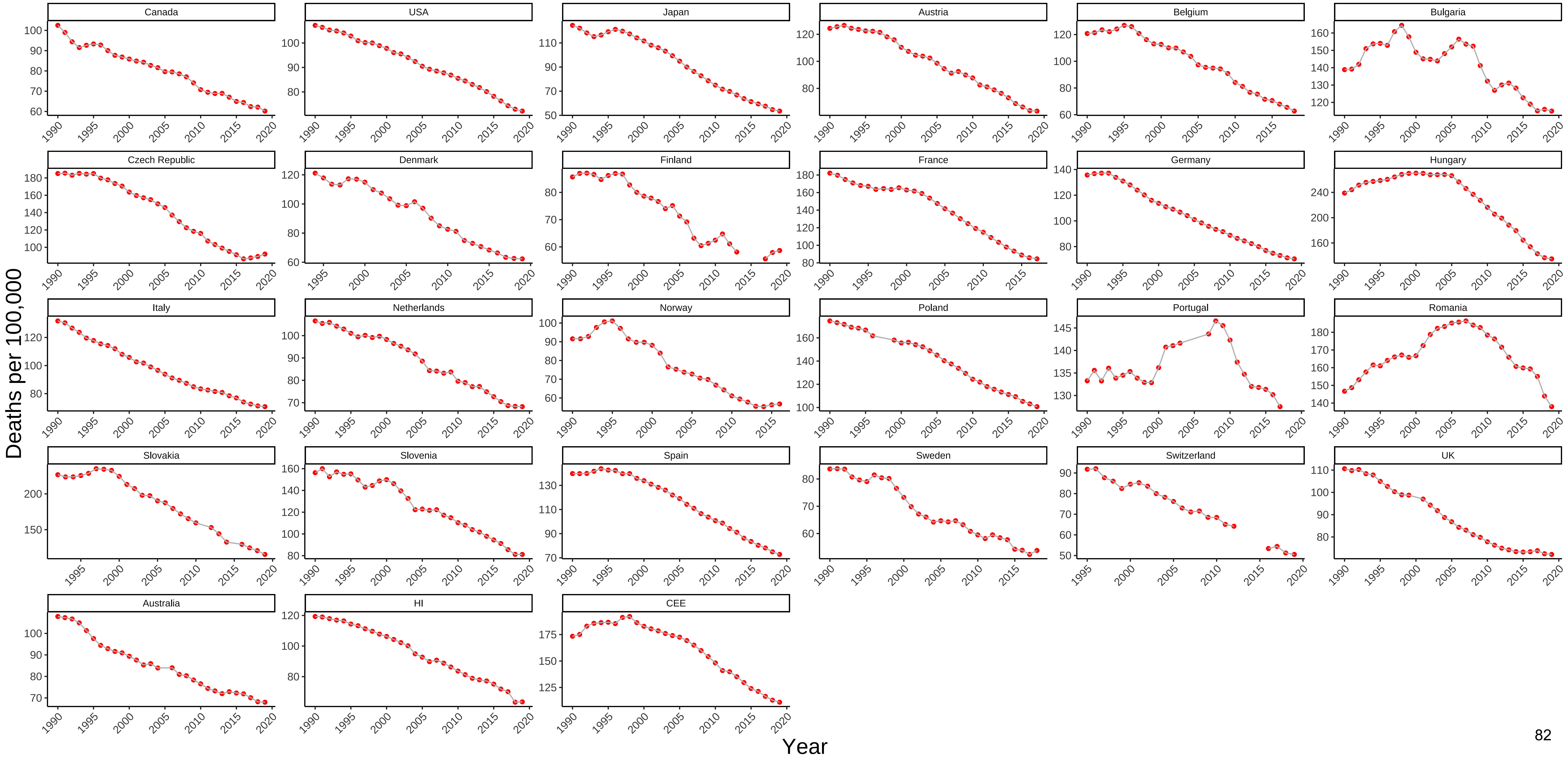

Figure S76. Three-Year Moving Average of Female Mortality from All Other Cancers at Ages 45-54

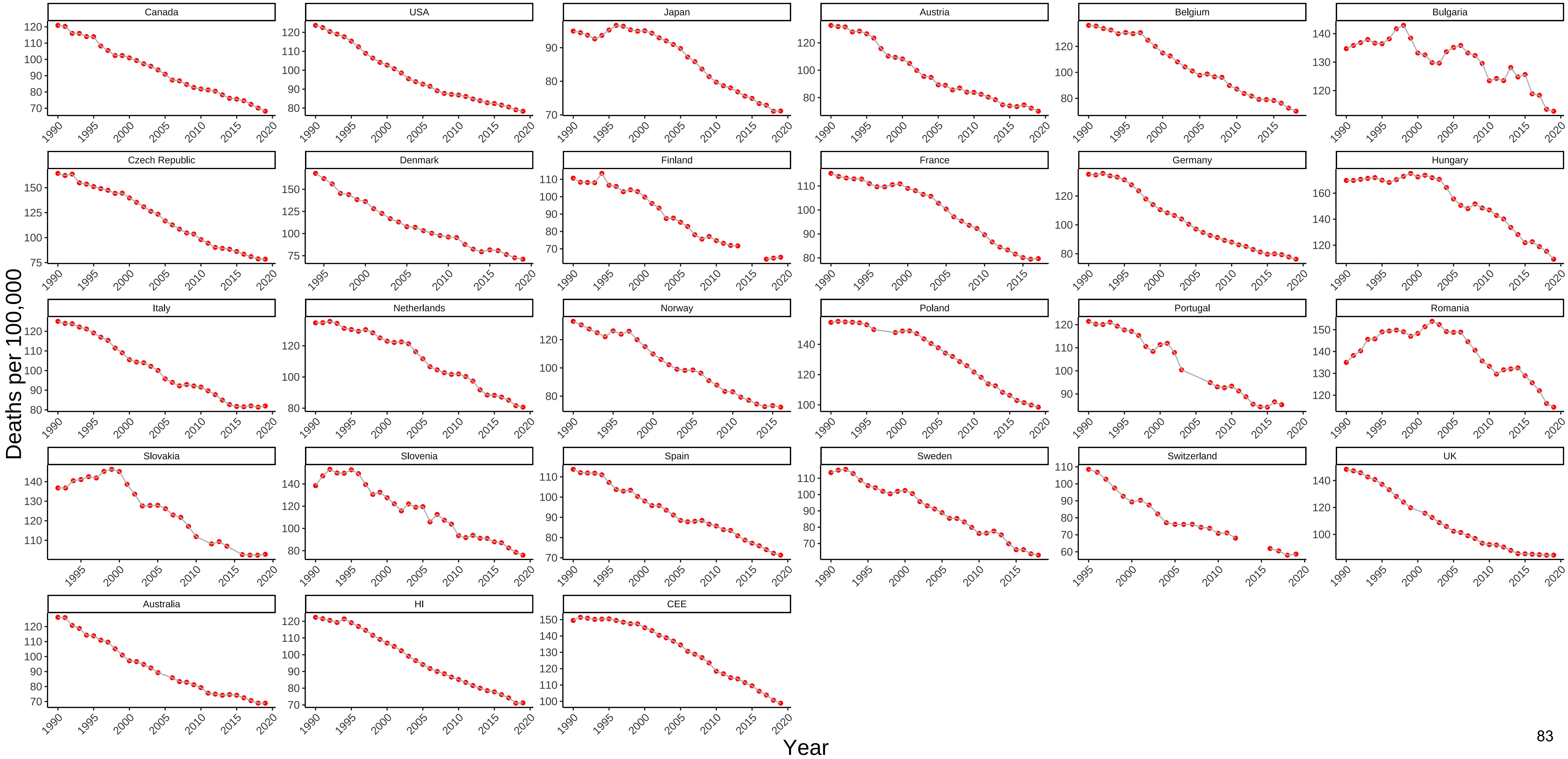

Figure S77. Three-Year Moving Average of Male Mortality from Nervous System Diseases at Ages 45-54

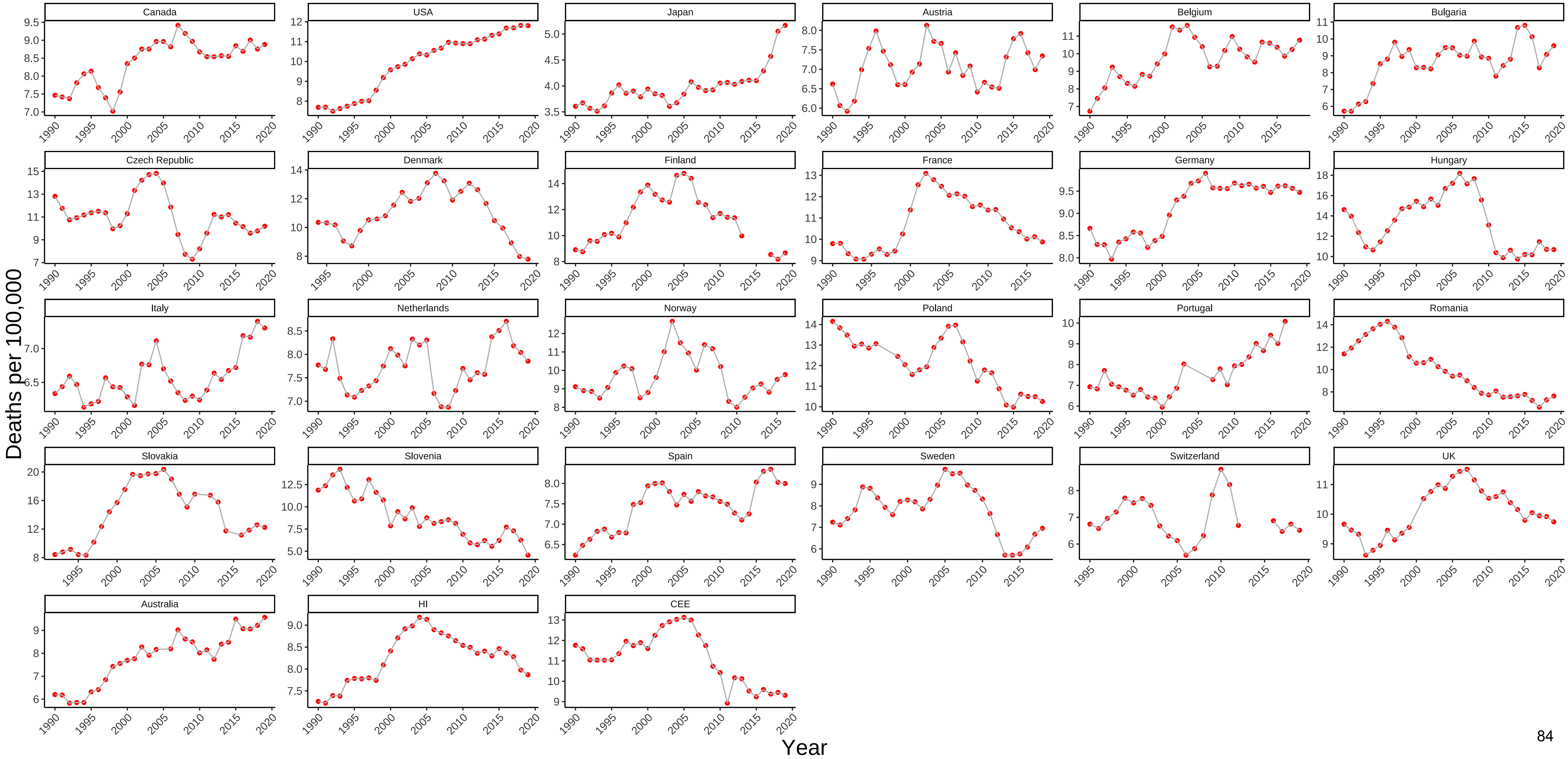

Figure S78. Three-Year Moving Average of Female Mortality from Nervous System Diseases at Ages 45-54

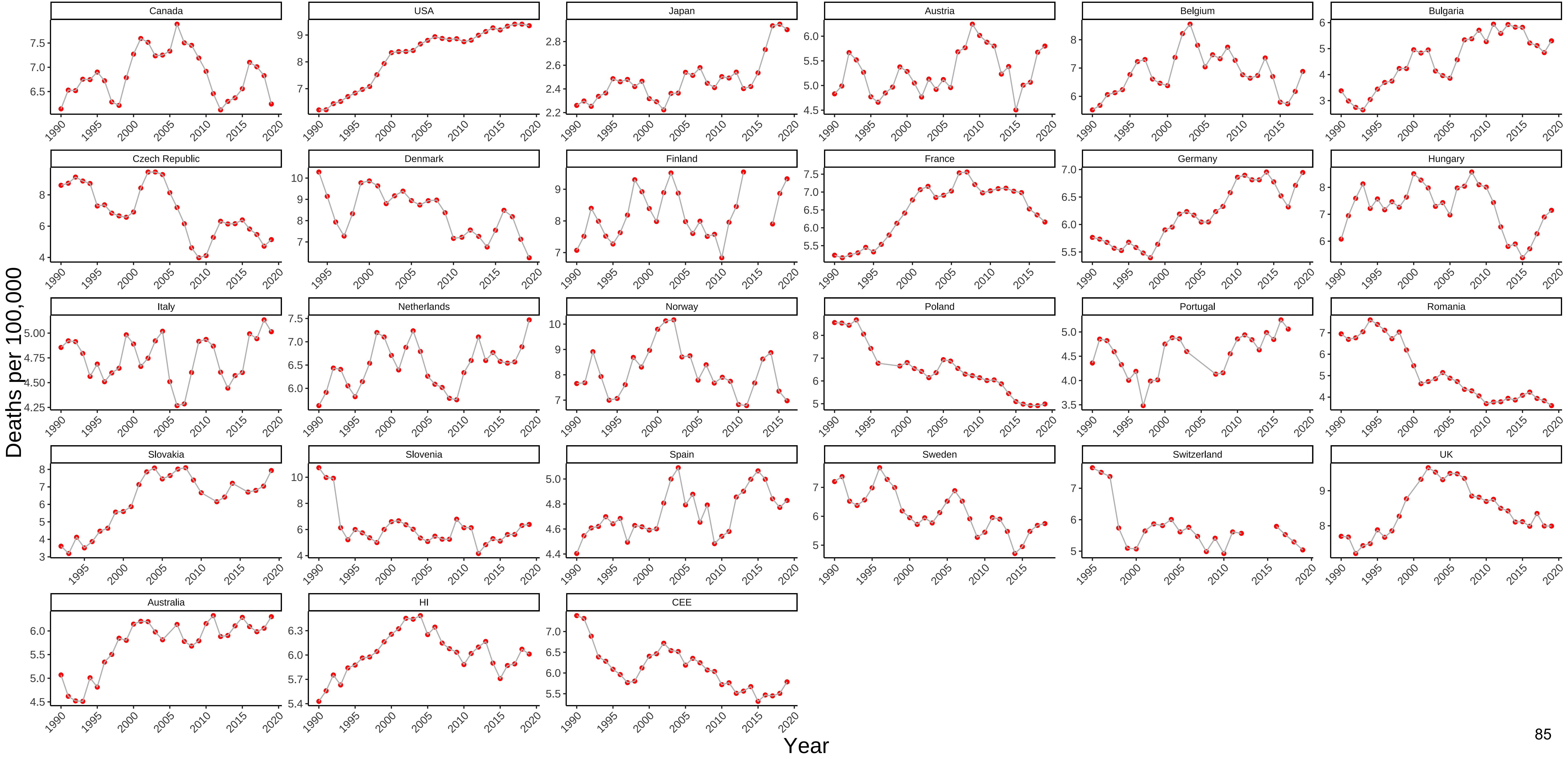

Figure S79. Three-Year Moving Average of Male Mortality from Metabolic Diseases at Ages 45-54

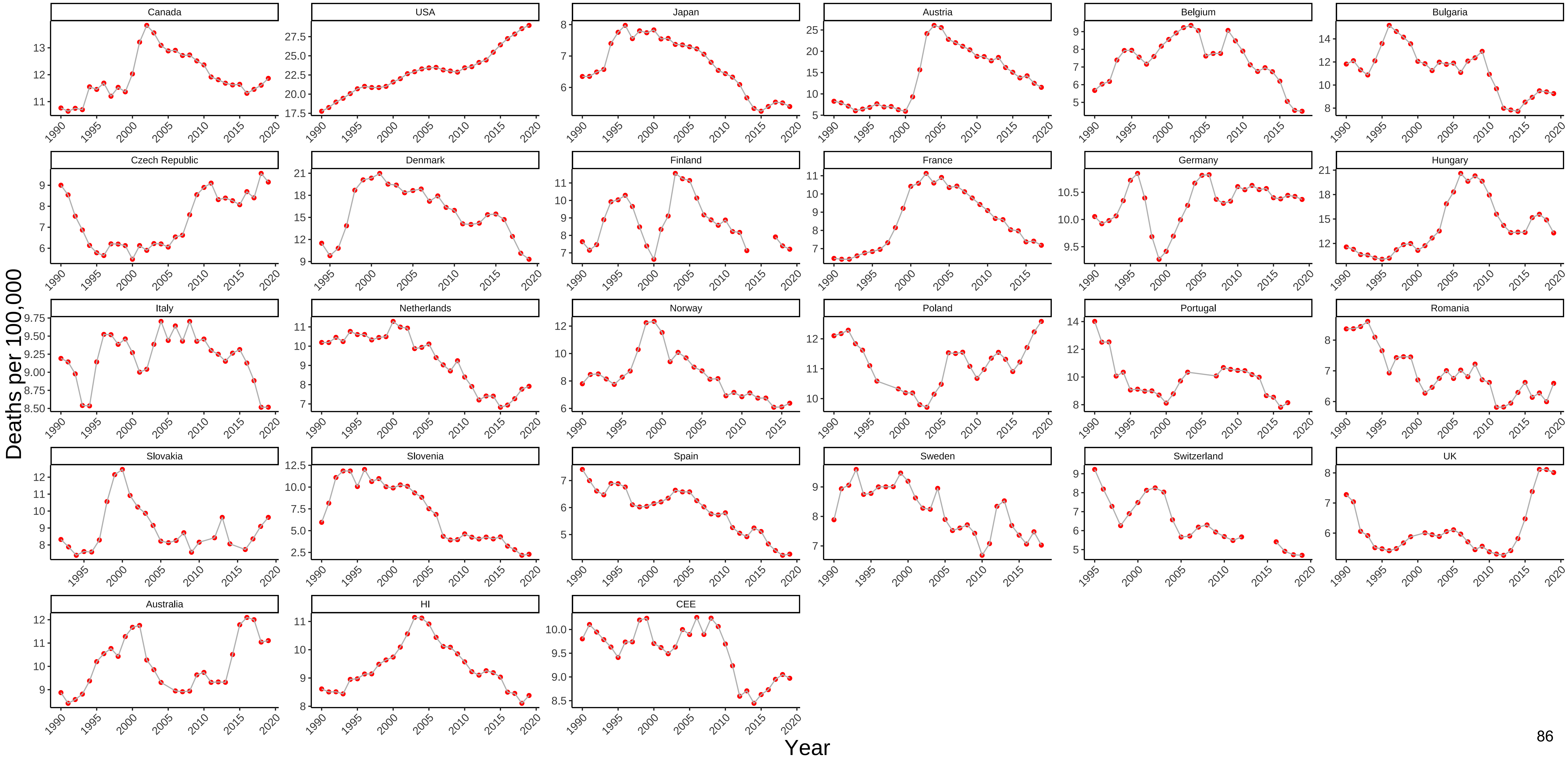

Figure S80. Three-Year Moving Average of Female Mortality from Metabolic Diseases at Ages 45-54

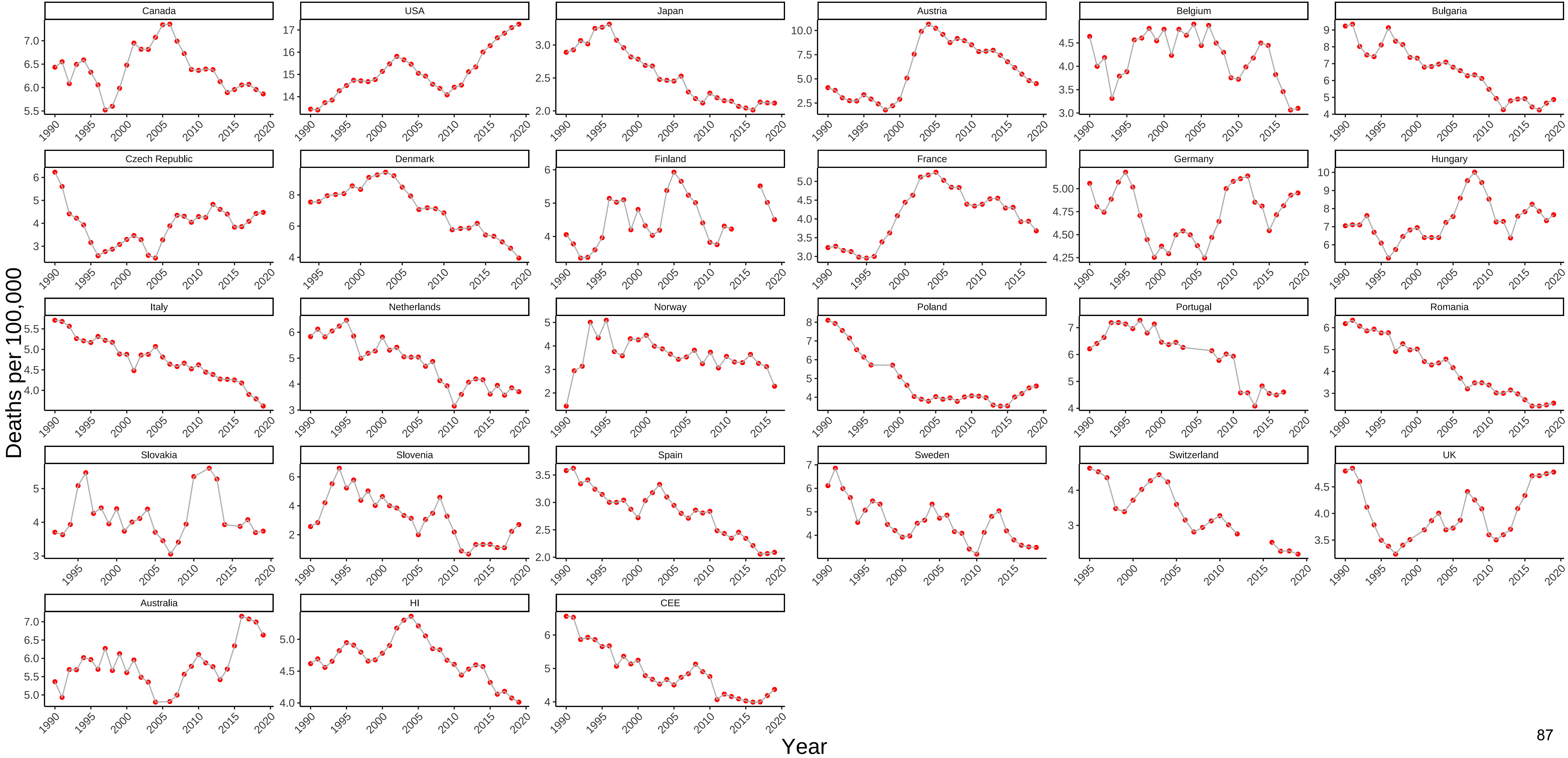

Figure S81. Three-Year Moving Average of Male Mortality from Cardiovascular Disease at Ages 45-54

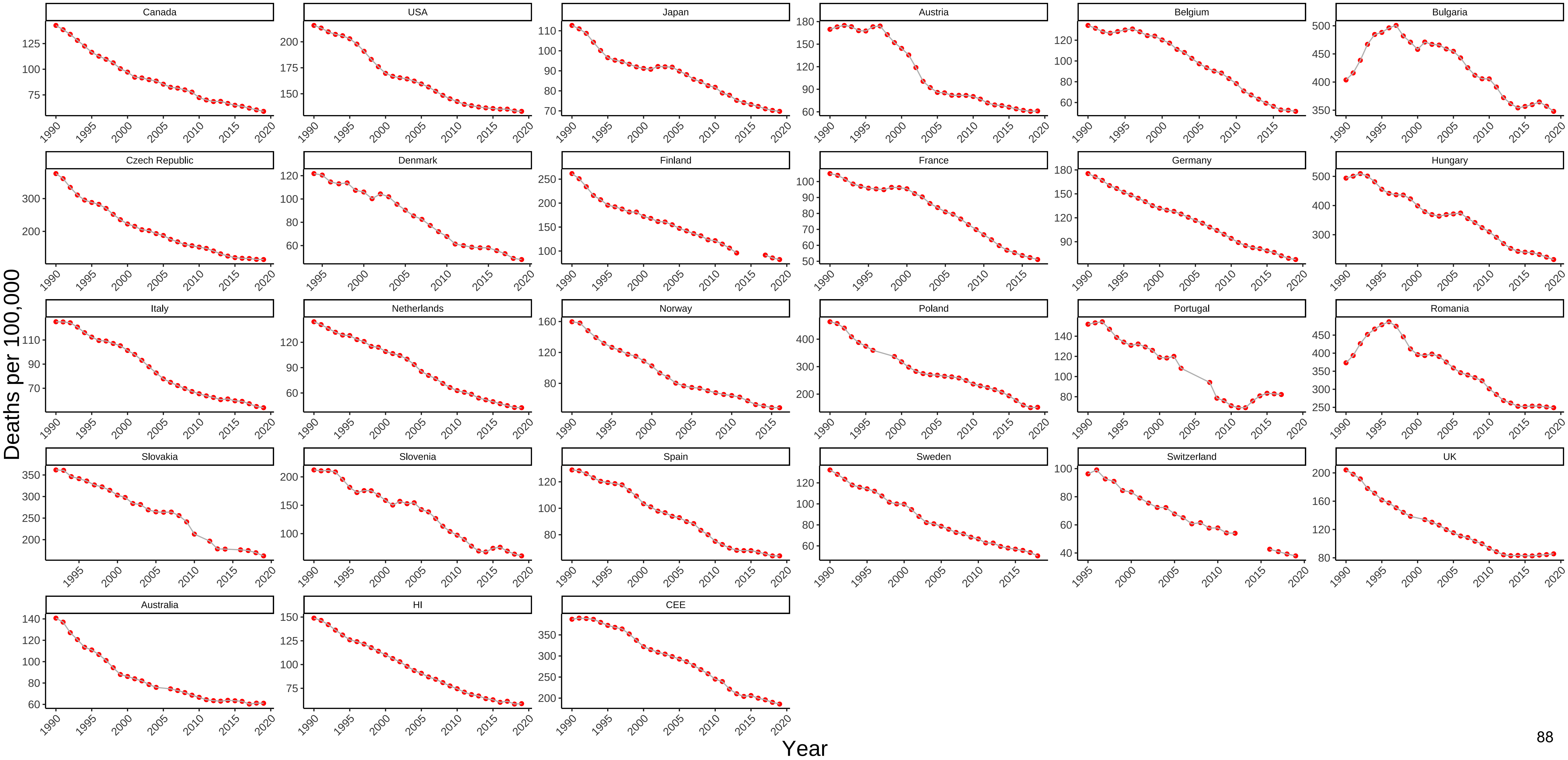

Figure S82. Three-Year Moving Average of Female Mortality from Cardiovascular Disease at Ages 45-54

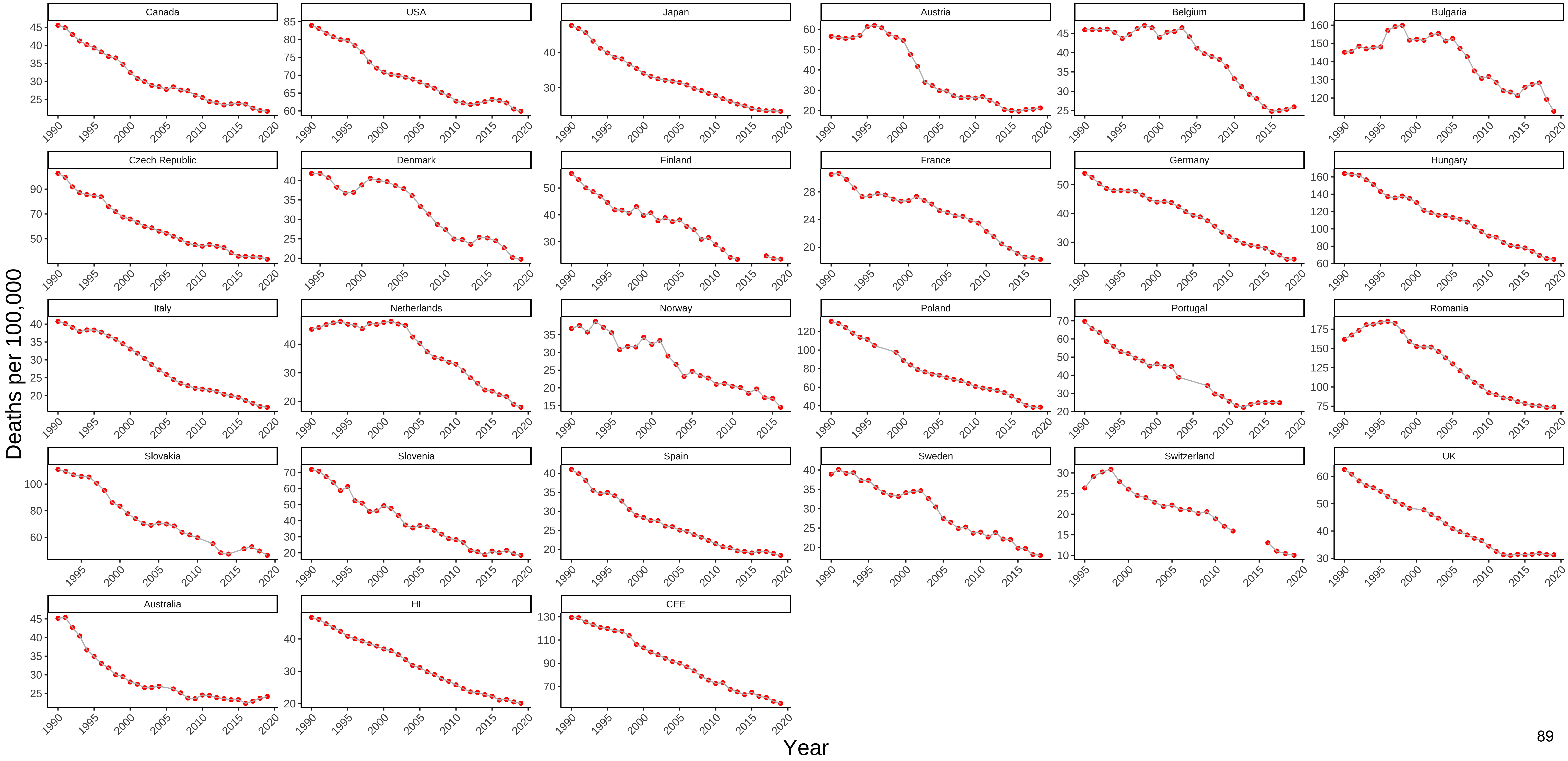

Figure S83. Three-Year Moving Average of Male Mortality from Suicide at Ages 45-54

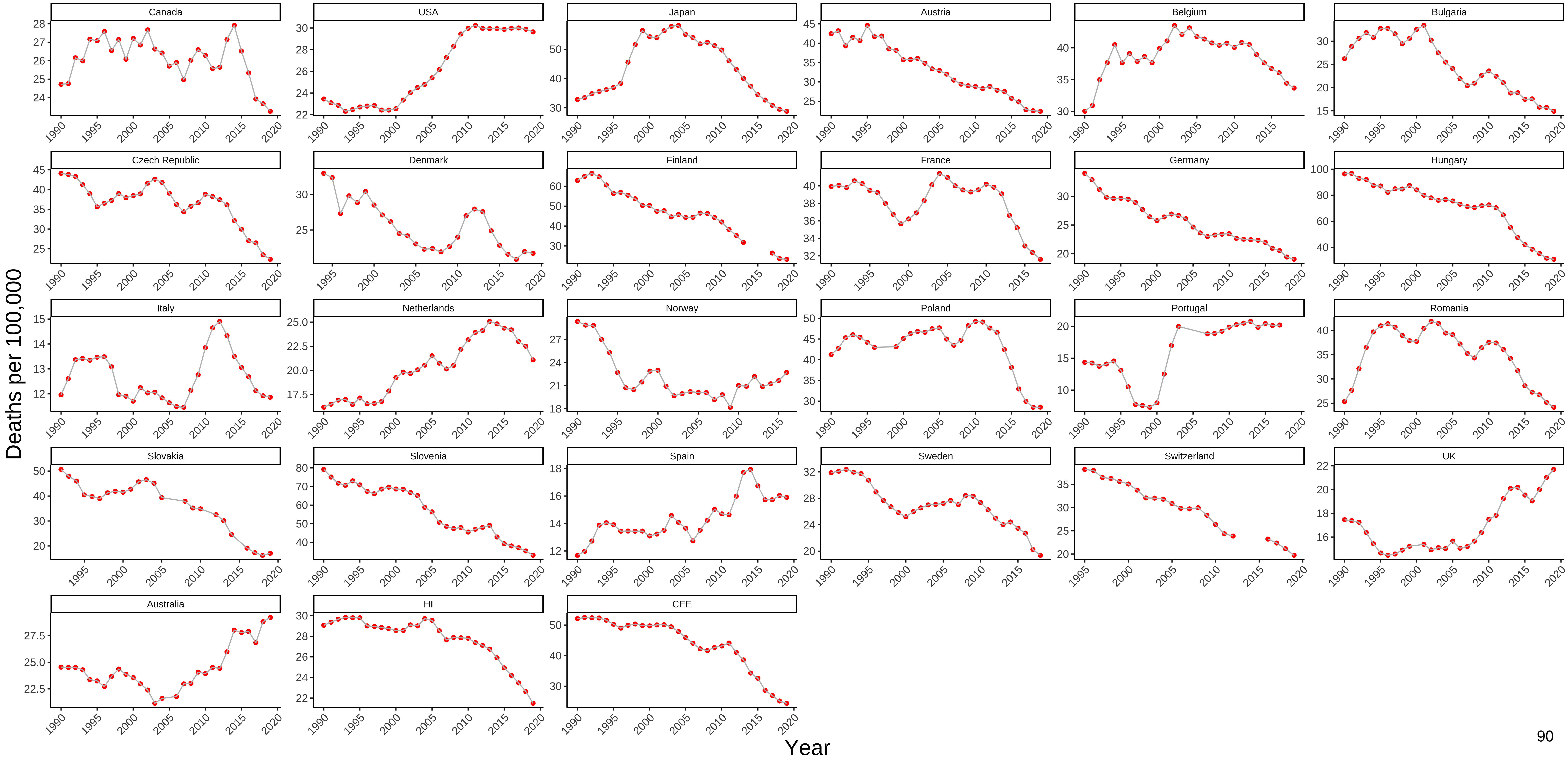

Figure S84. Three-Year Moving Average of Female Mortality from Suicide at Ages 45-54

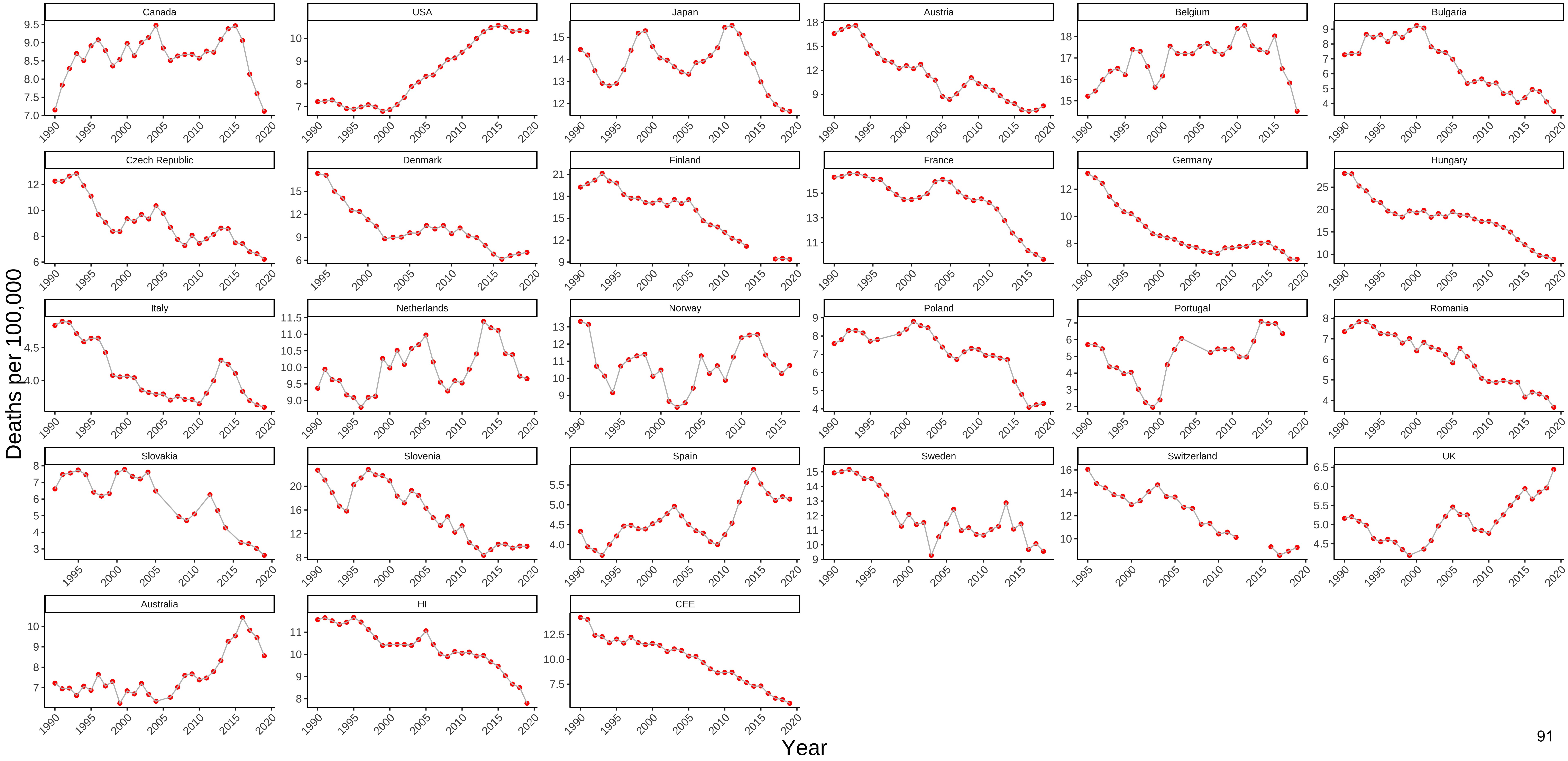

Figure S85. Three-Year Moving Average of Male Mortality from Homicide at Ages 45-54

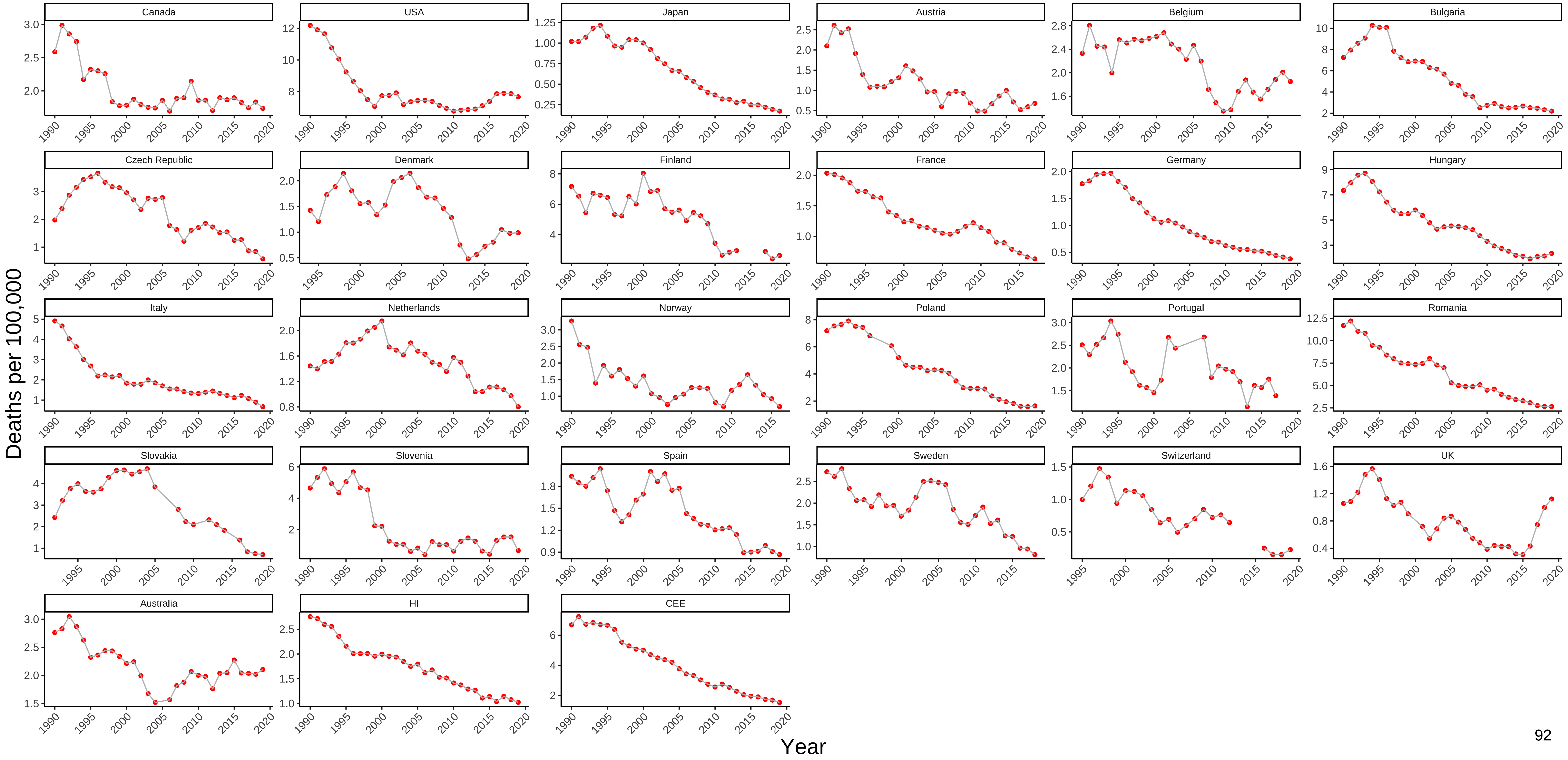

Figure S86. Three-Year Moving Average of Female Mortality from Homicide at Ages 45-54

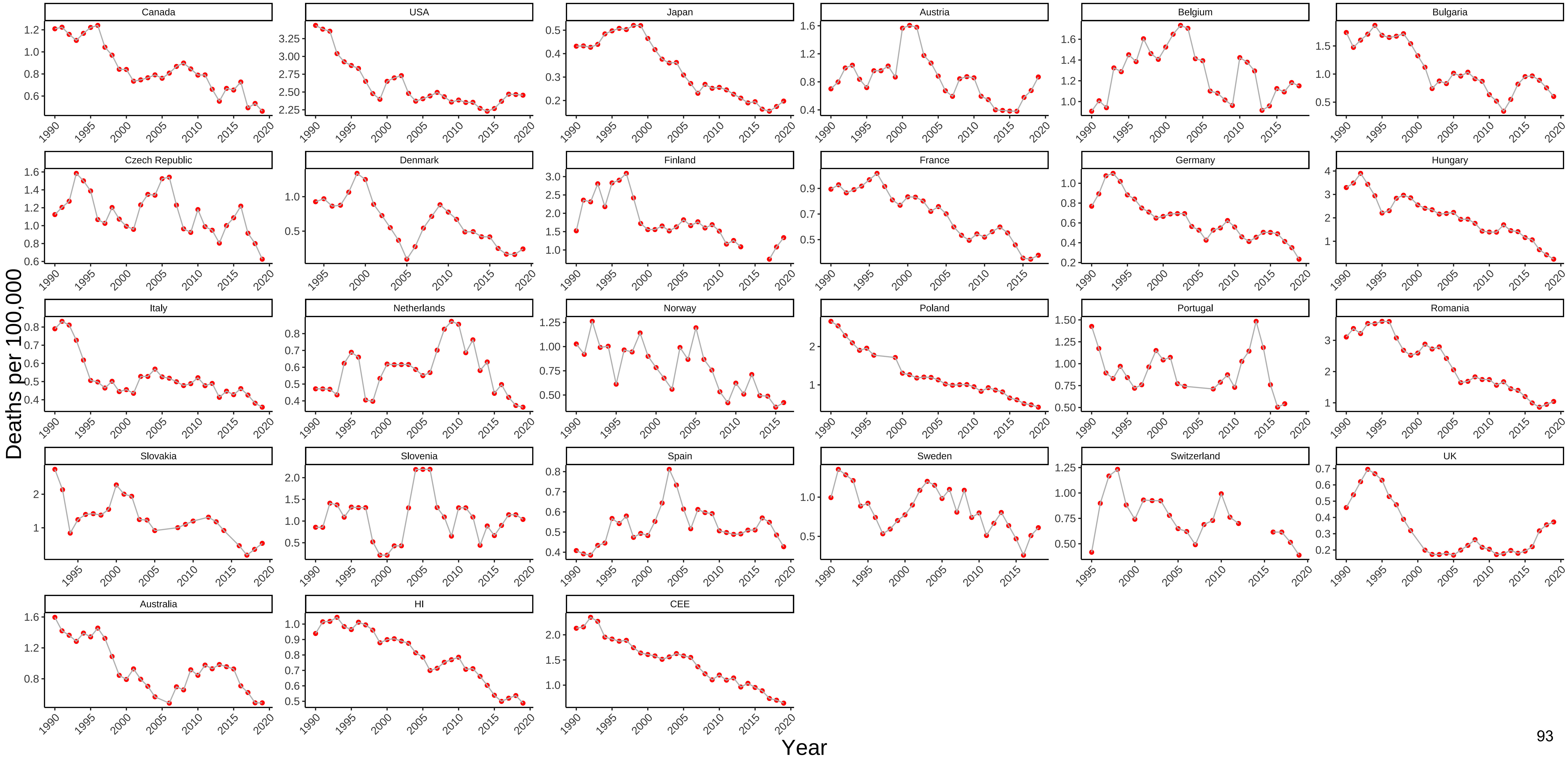

Figure S87. Three-Year Moving Average of Male Mortality from Transport Accidents at Ages 45-54

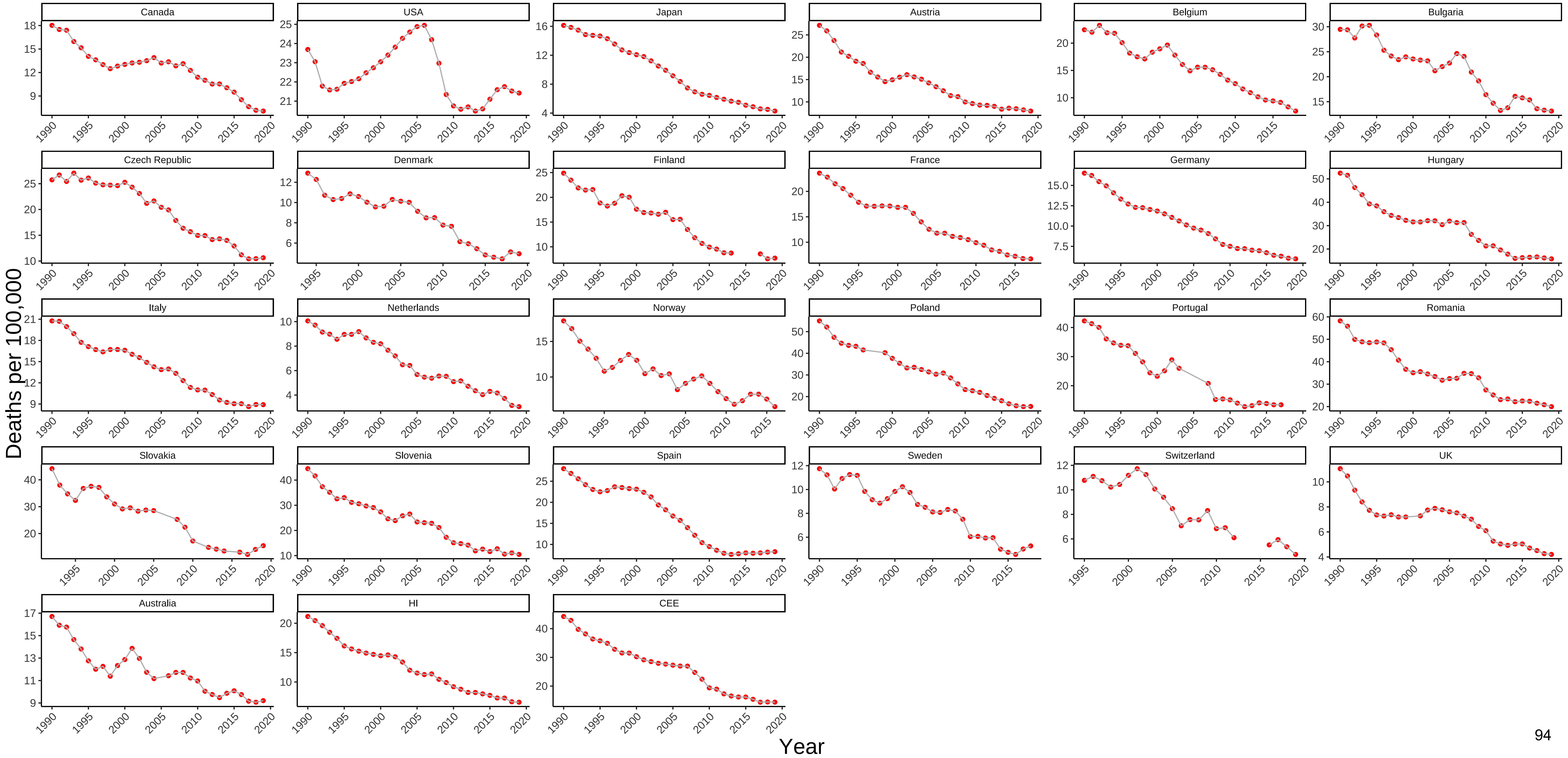

Figure S88. Three-Year Moving Average of Female Mortality from Transport Accidents at Ages 45-54

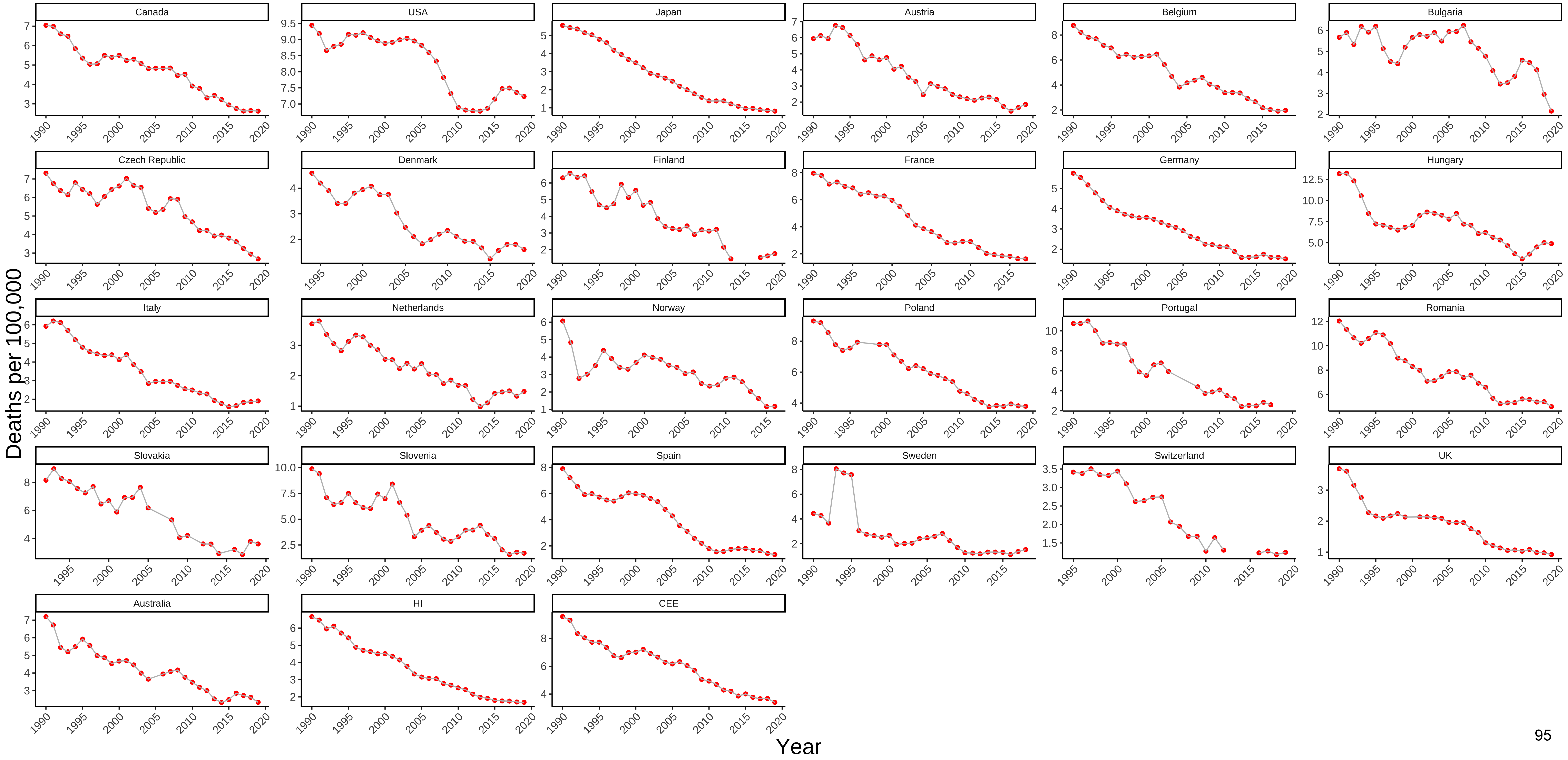

Figure S89. Three-Year Moving Average of Male Mortality from Other External Causes at Ages 45-54

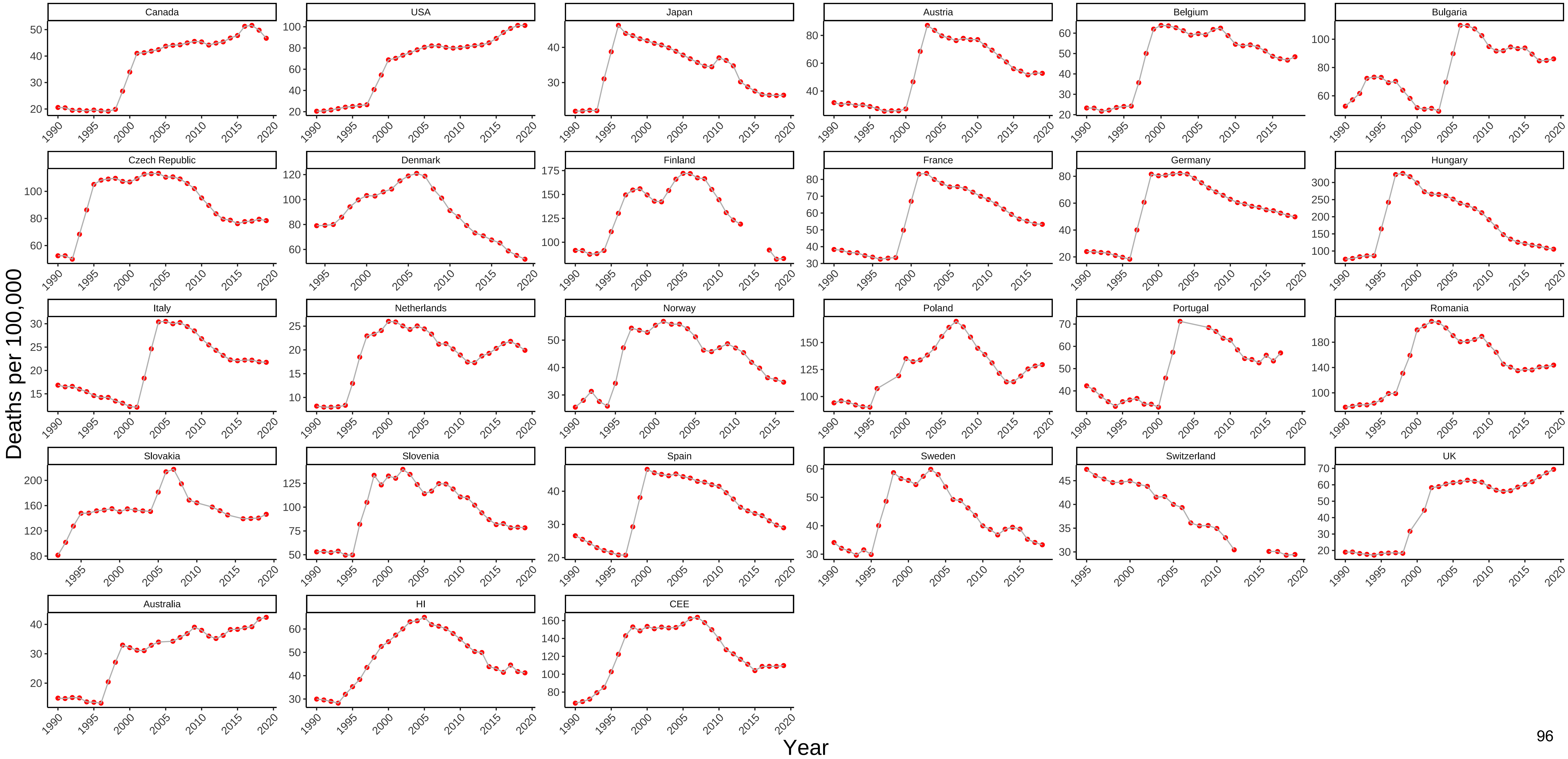

Figure S90. Three-Year Moving Average of Female Mortality from Other External Causes at Ages 45-54

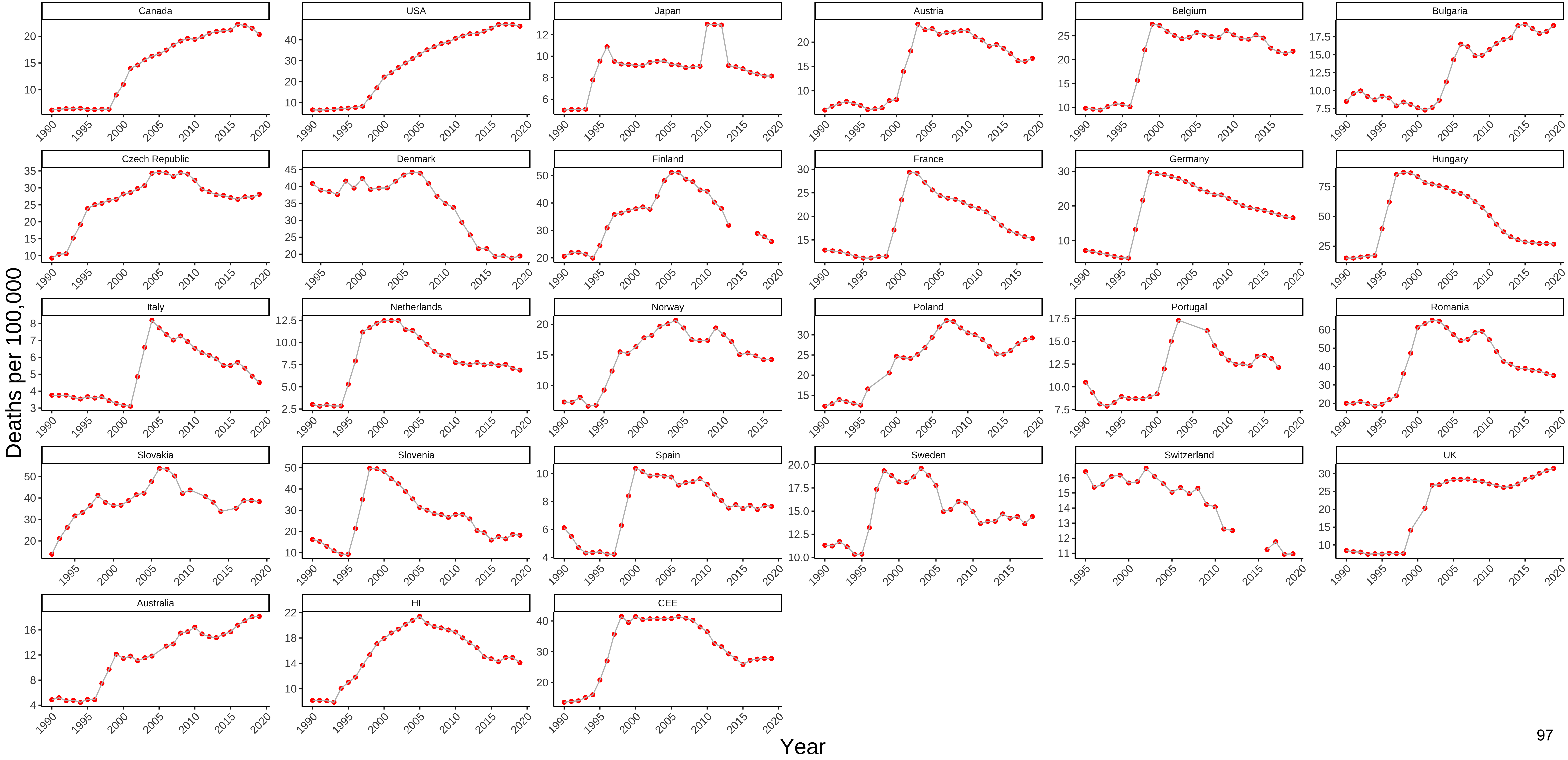

Figure S91. Three-Year Moving Average of Male Mortality from All Other Causes at Ages 45-54

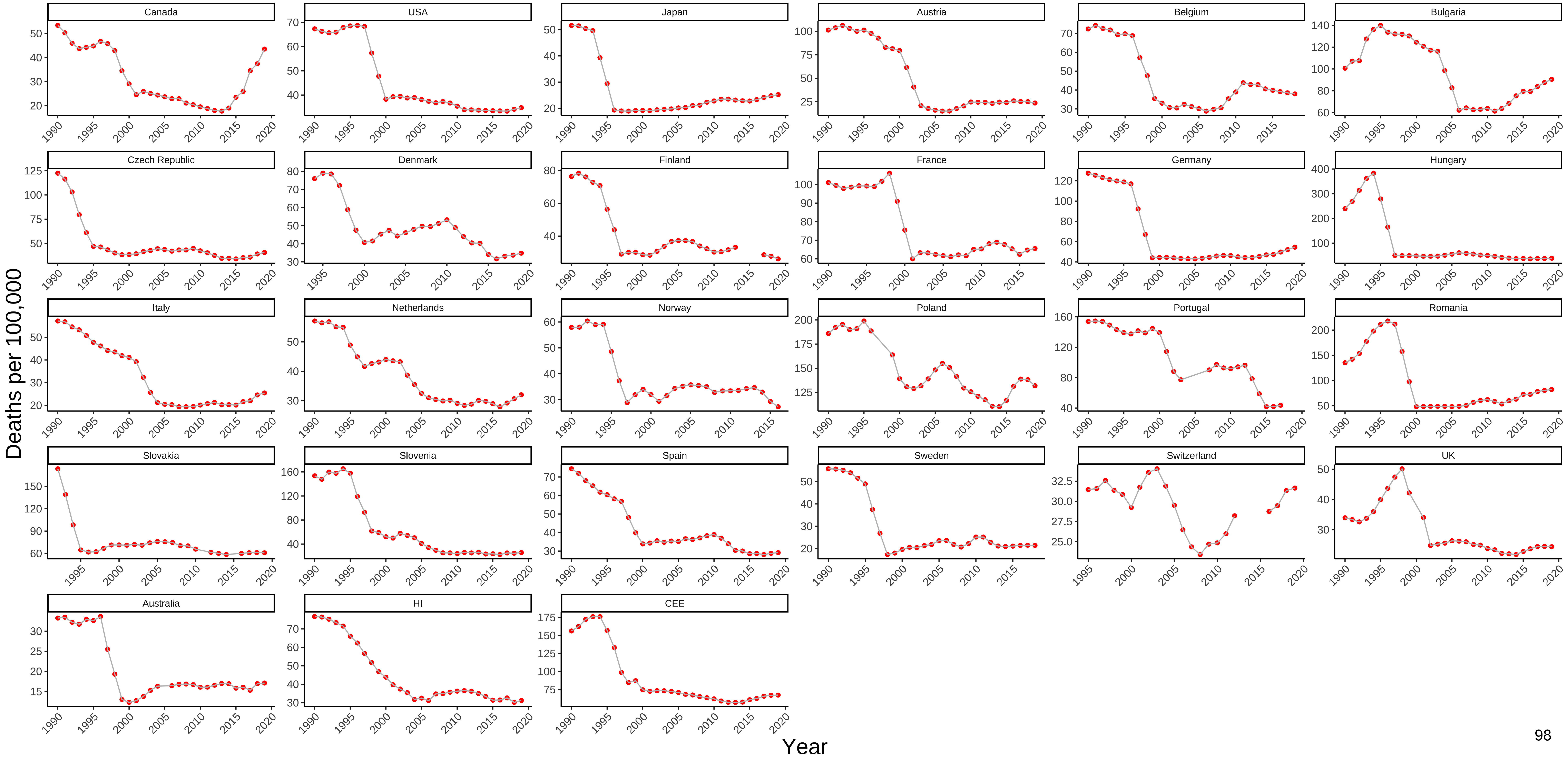

Figure S92. Three-Year Moving Average of Female Mortality from All Other Causes at Ages 45-54

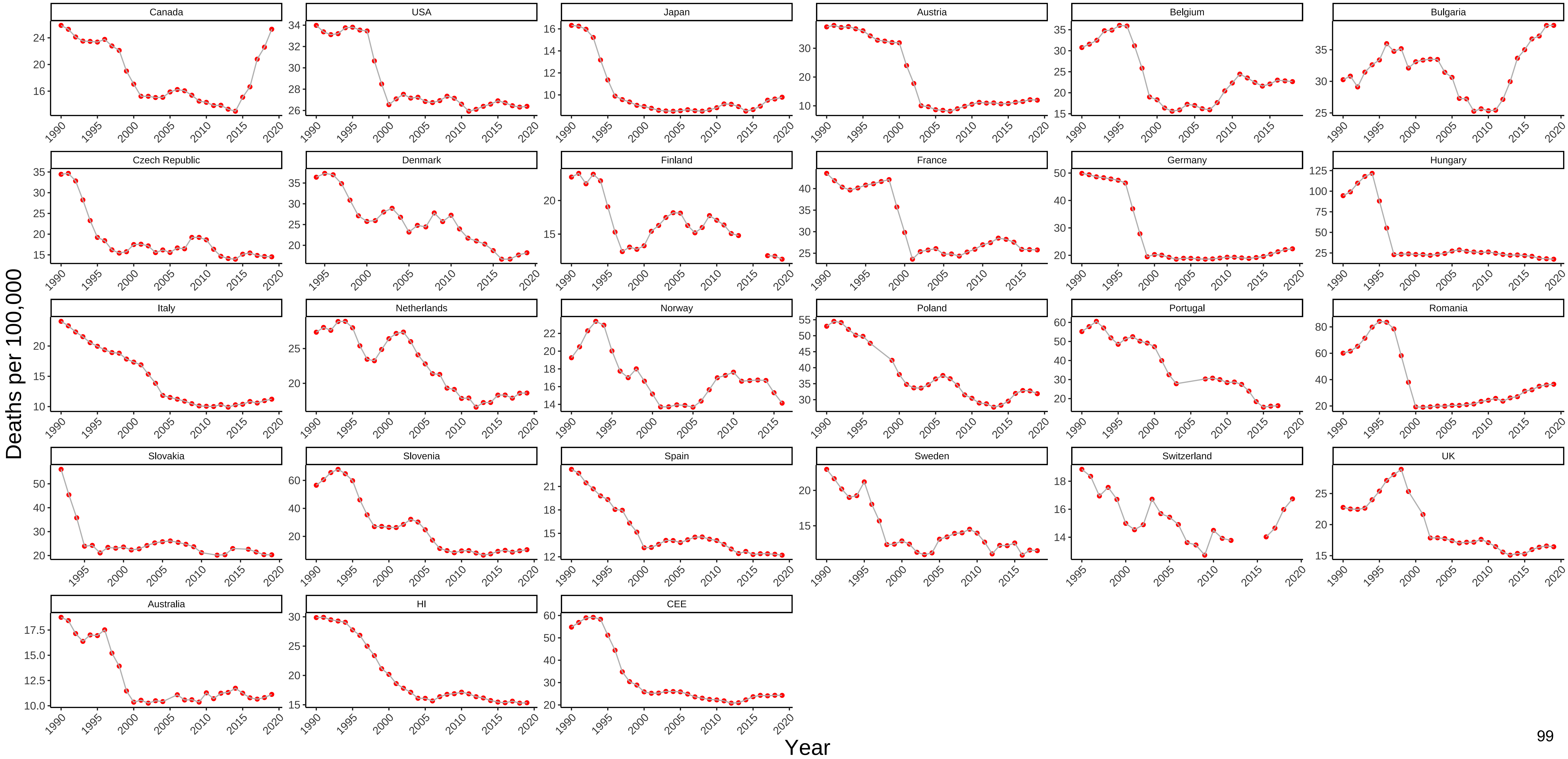

Figure S93. Three-Year Moving Average of Male Mortality from All Causes at Ages 45-54

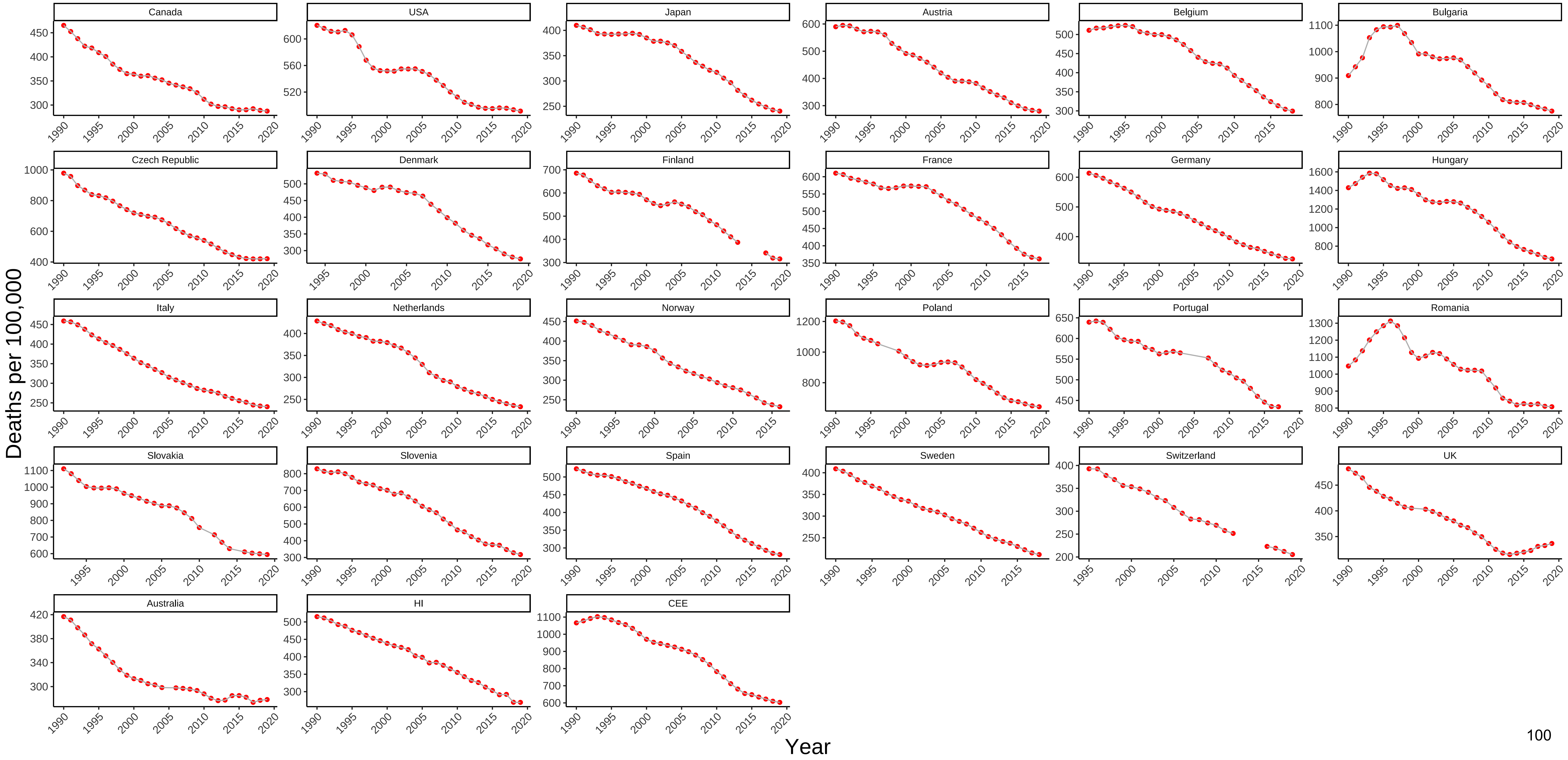

Figure S94. Three-Year Moving Average of Female Mortality from All Causes at Ages 45-54

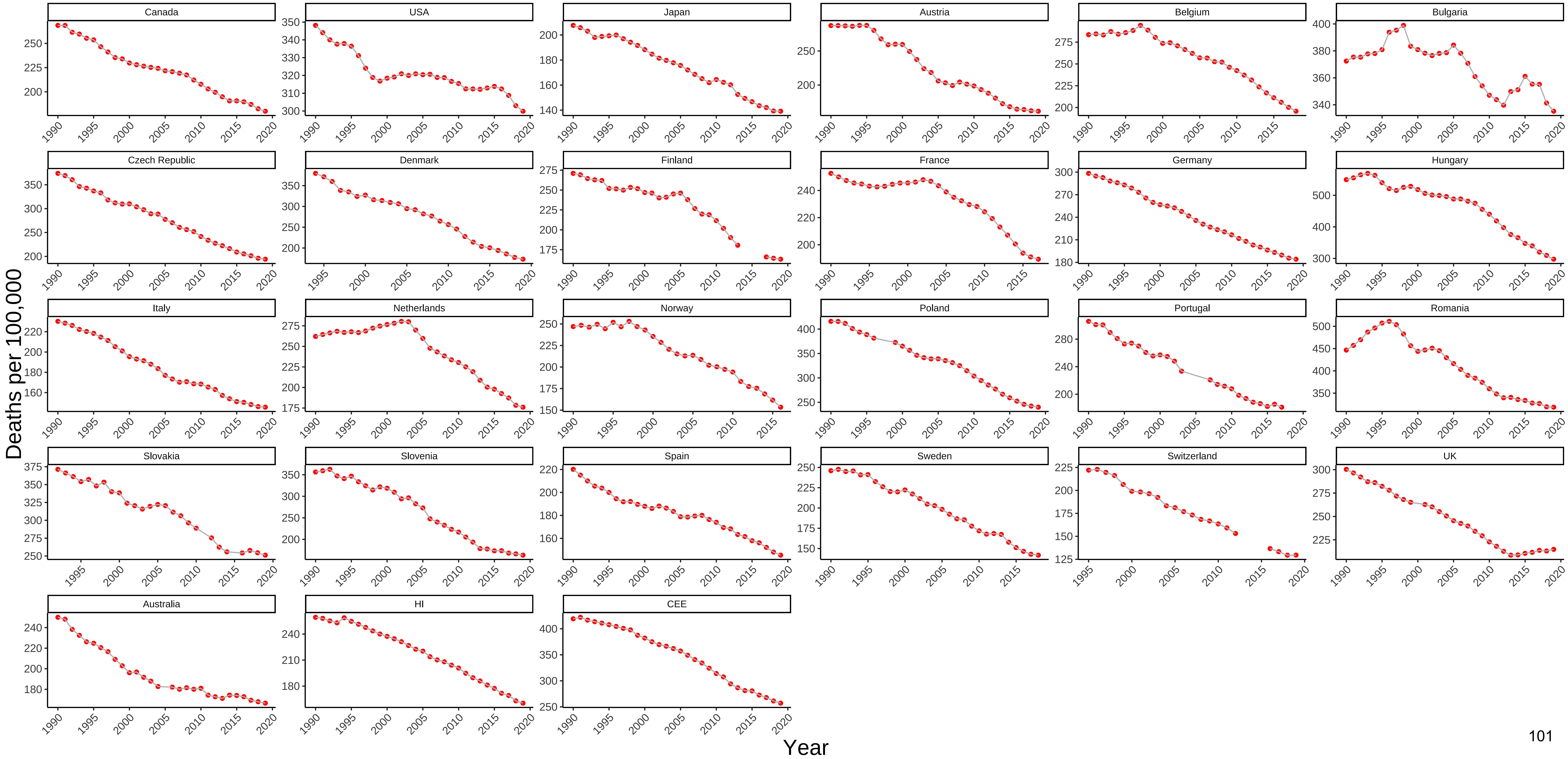

Figure S95. Three-Year Moving Average of Male Mortality from Infectious and Parasitic Diseases at Ages 55-64

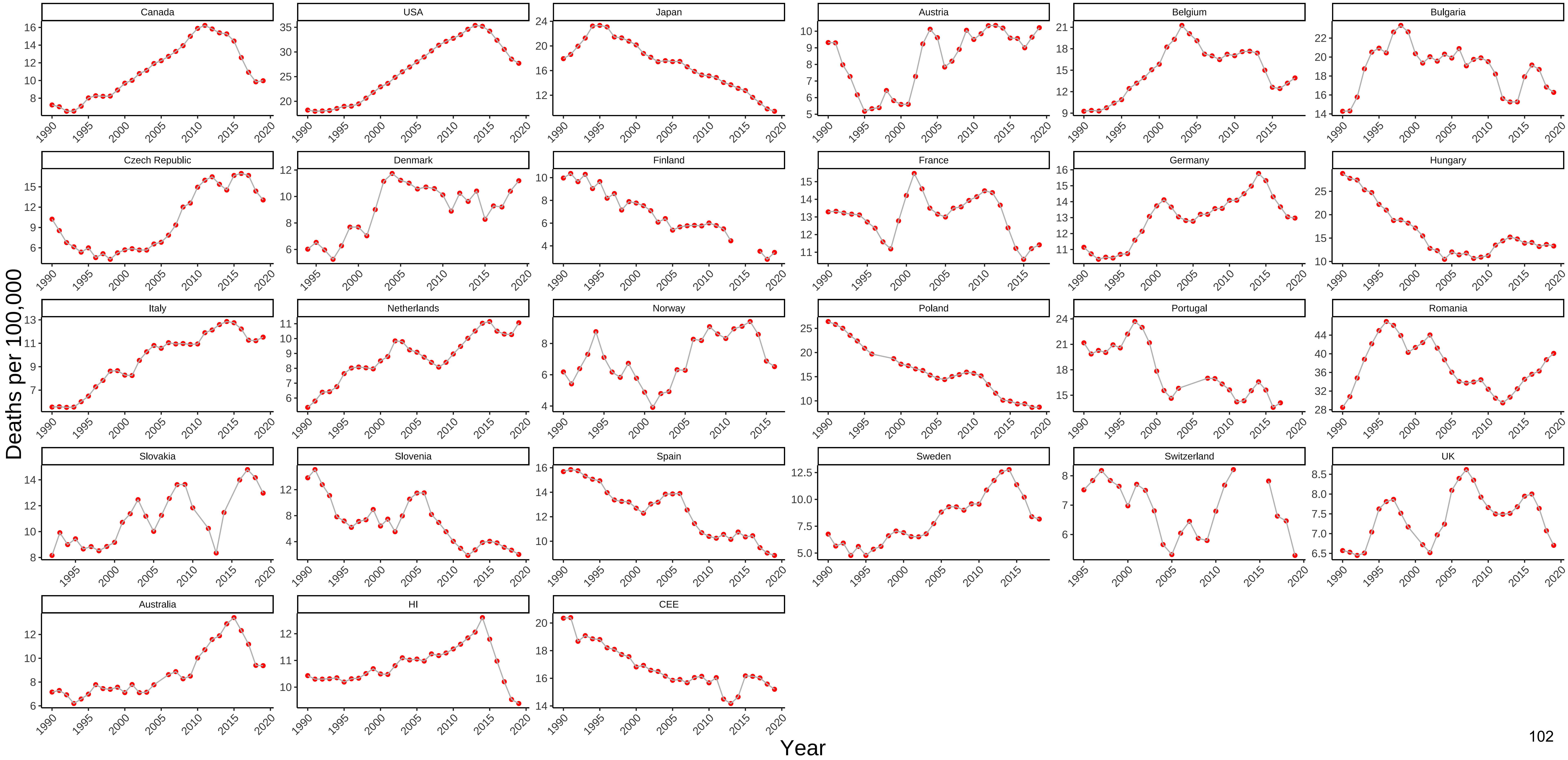

Figure S96. Three-Year Moving Average of Female Mortality from Infectious and Parasitic Diseases at Ages 55-64

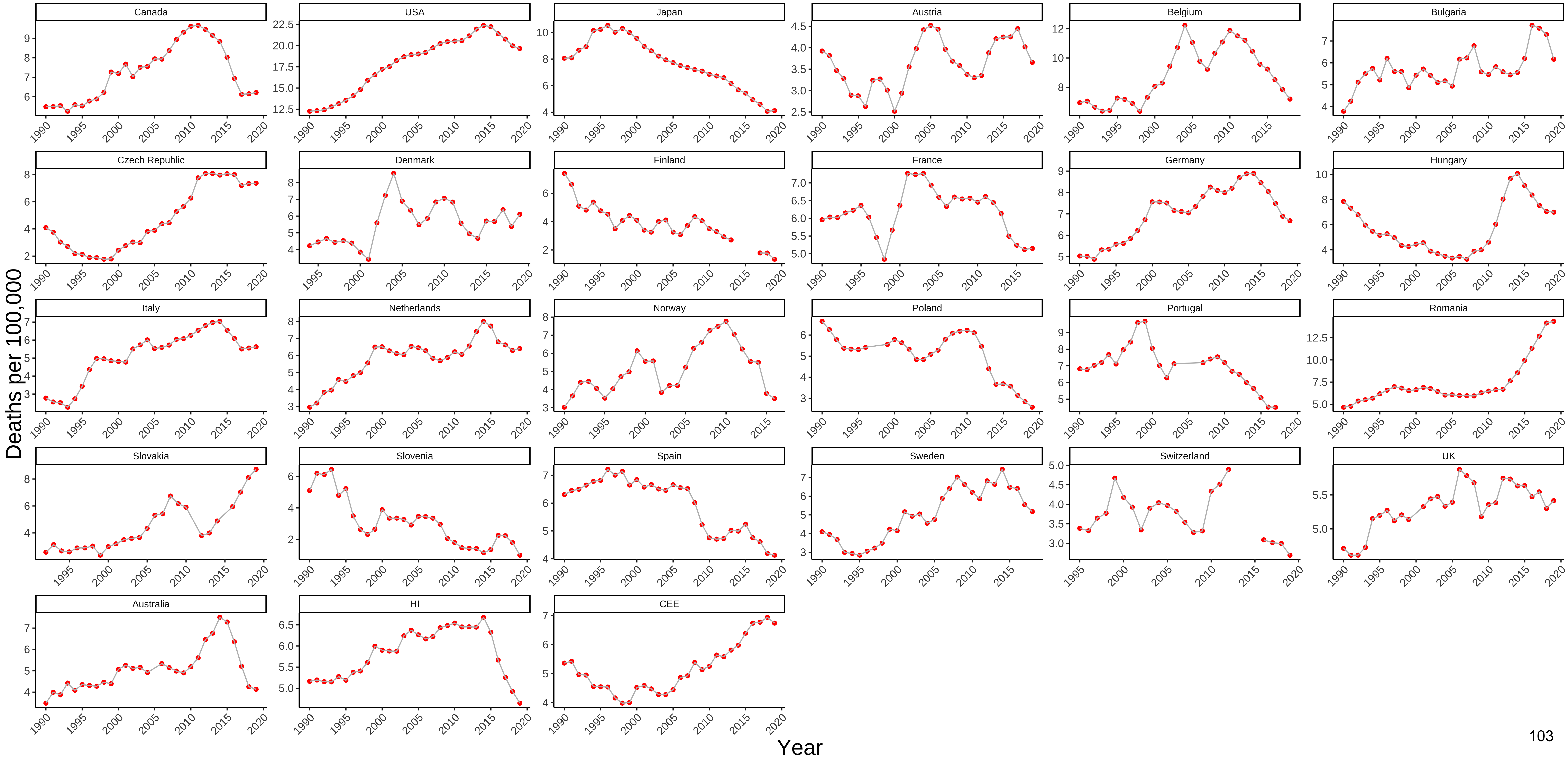

Figure S97. Three-Year Moving Average of Male Mortality from HIV/AIDS at Ages 55-64

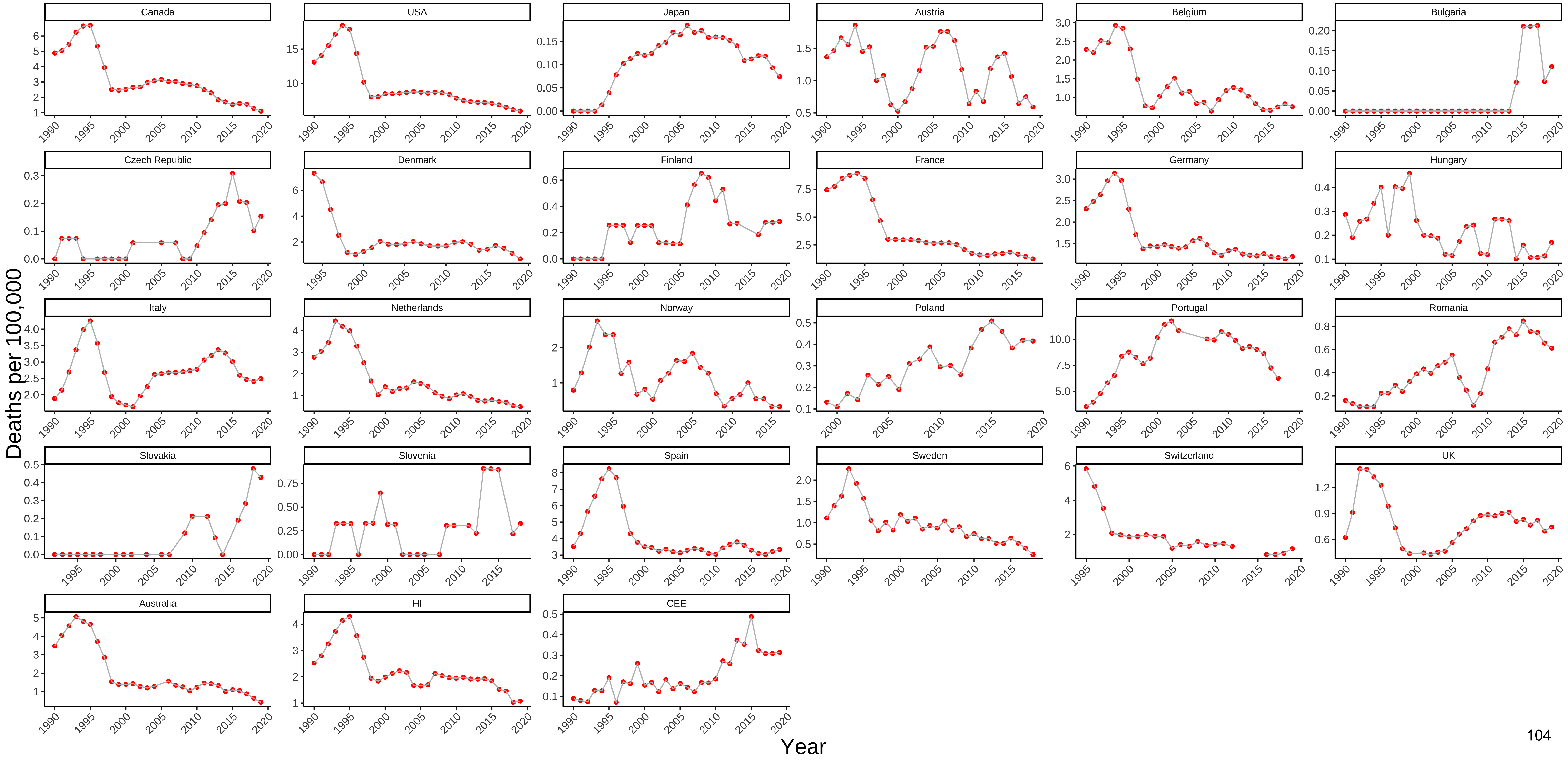

Figure S98. Three-Year Moving Average of Female Mortality from HIV/AIDS at Ages 55-64

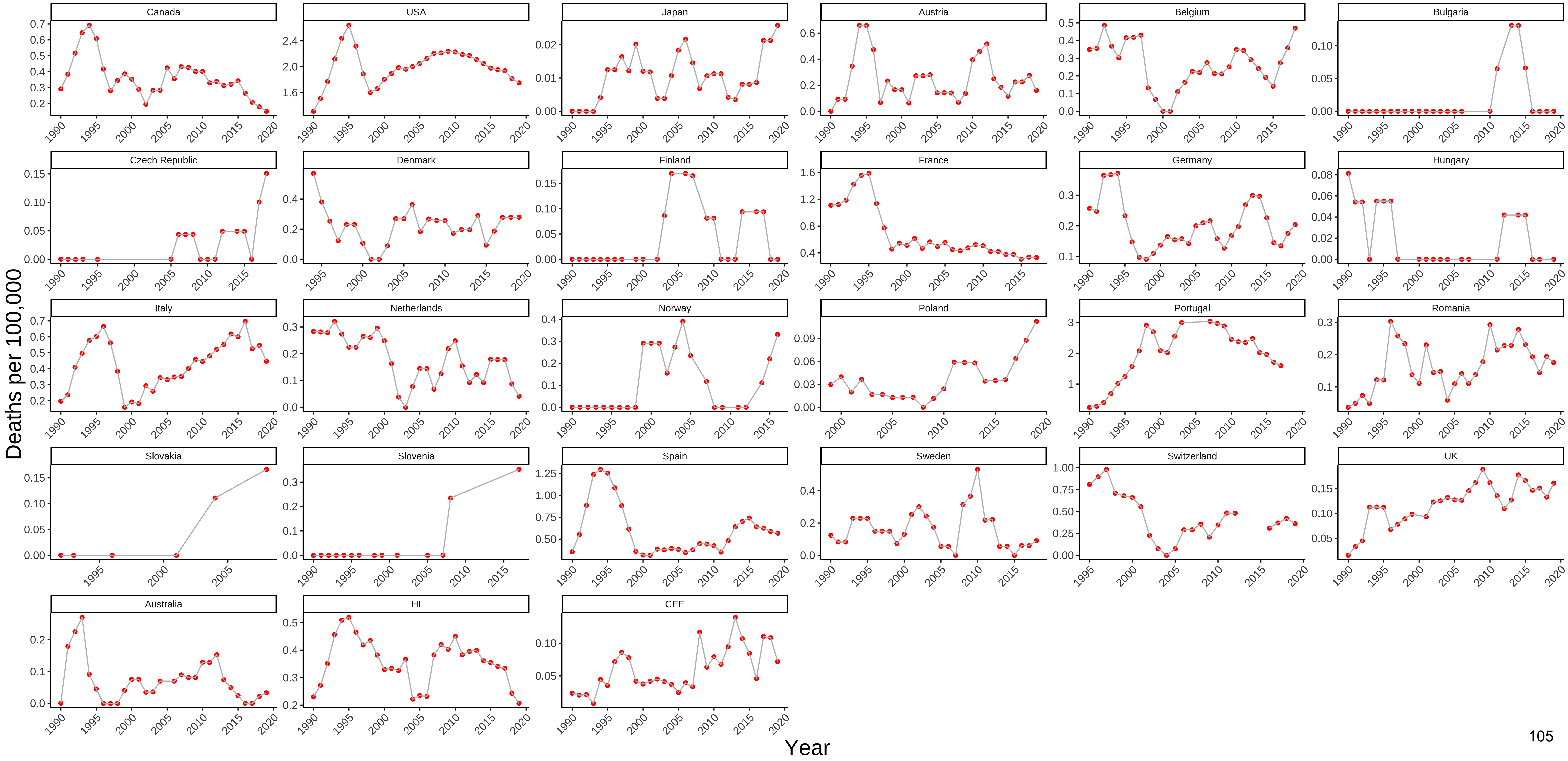

Figure S99. Three-Year Moving Average of Male Mortality from Respiratory Diseases at Ages 55-64

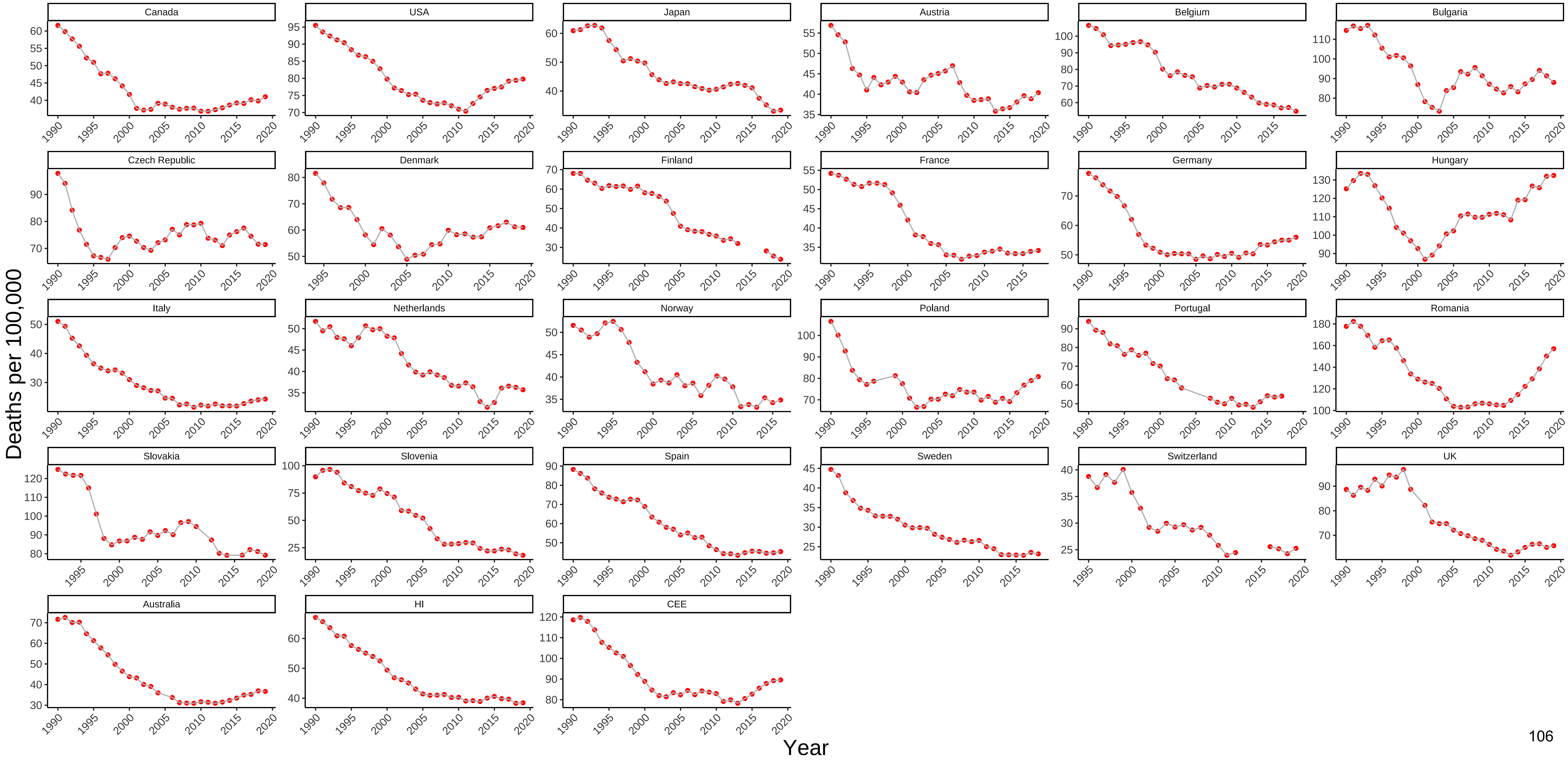

Figure S100. Three-Year Moving Average of Female Mortality from Respiratory Diseases at Ages 55-64

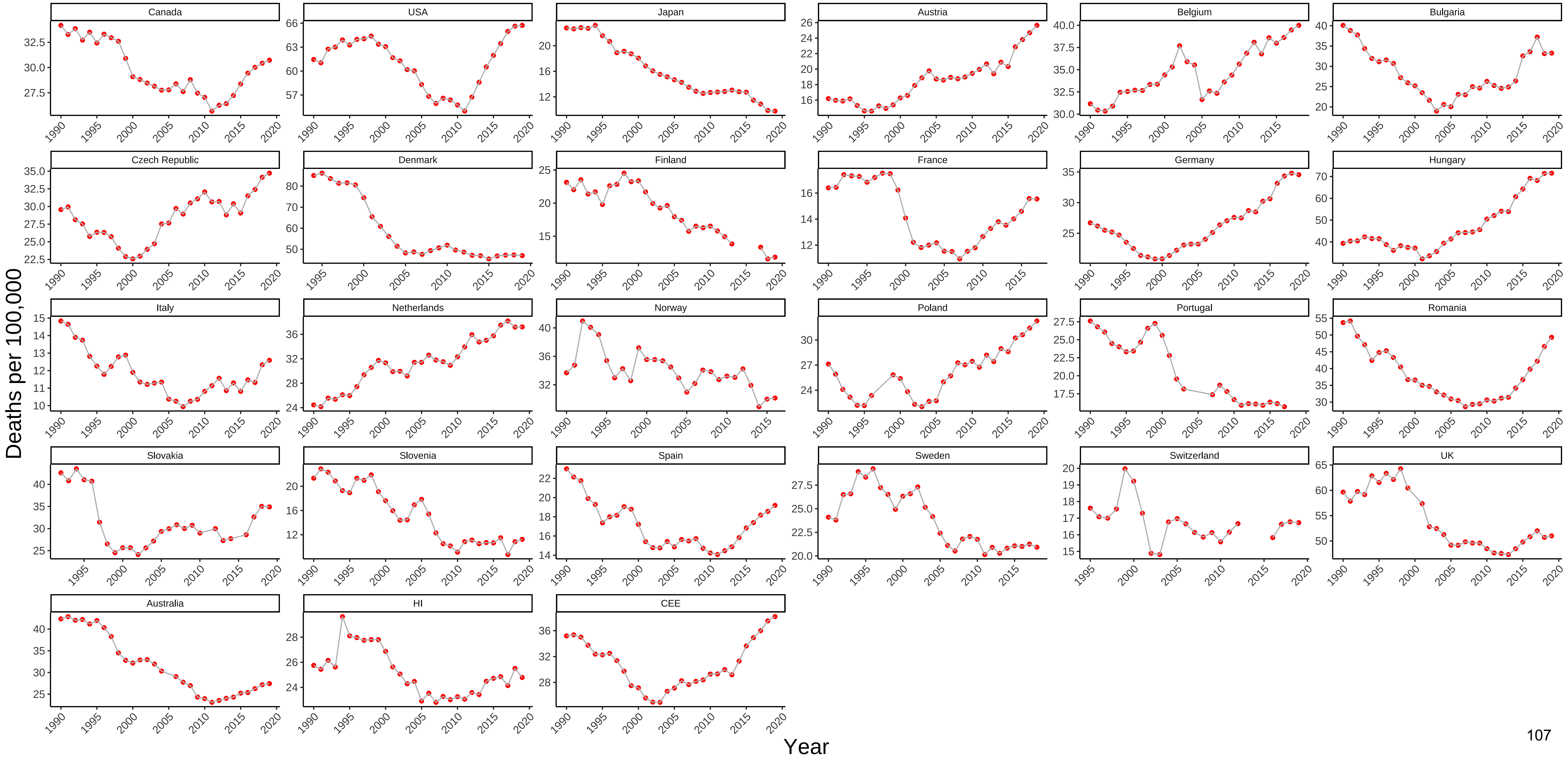

Figure S101. Three-Year Moving Average of Male Mortality from Trachea/Bronchus, Lung Cancers at Ages 55-64

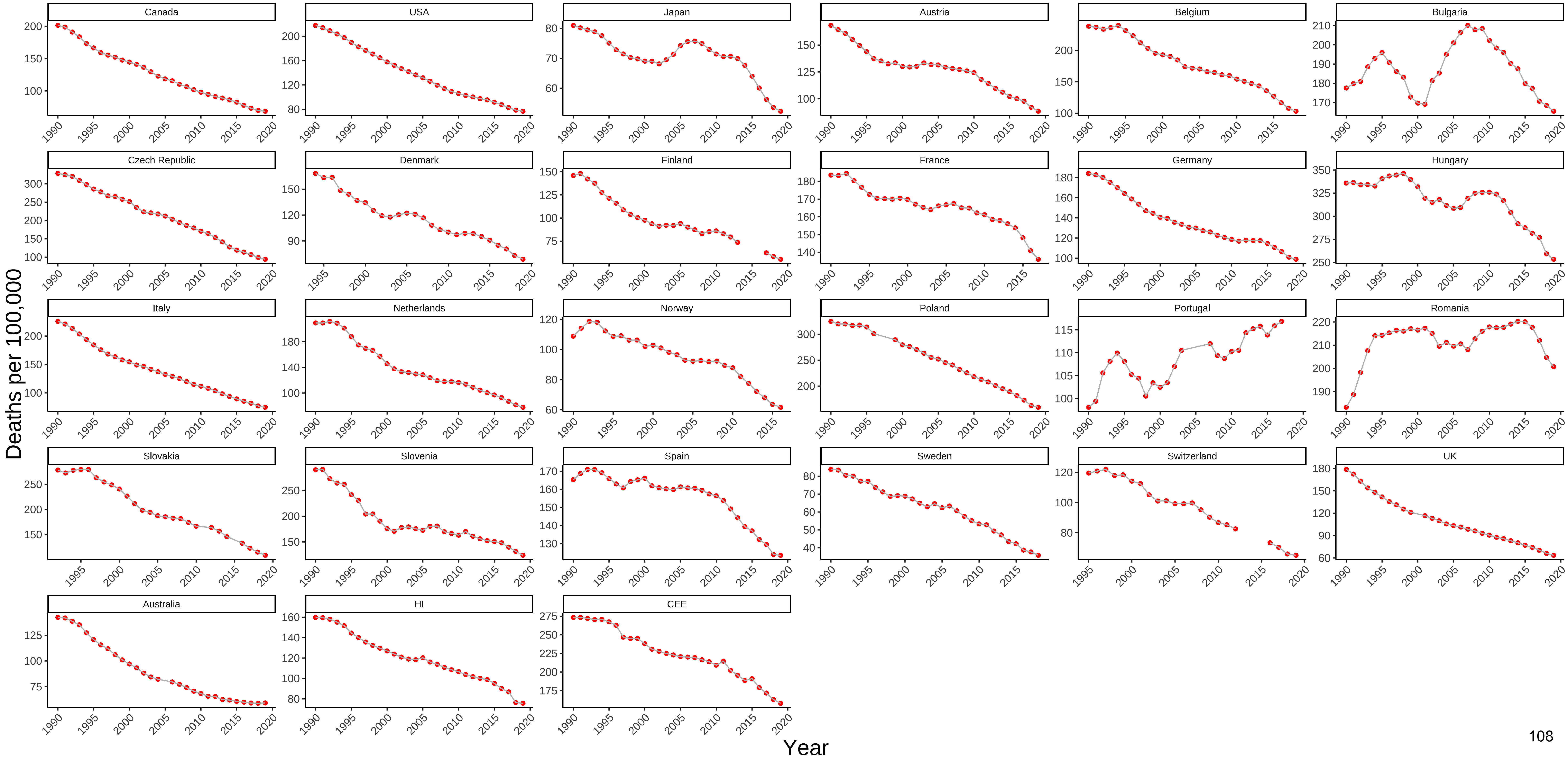

Figure S102. Three-Year Moving Average of Female Mortality from Trachea/Bronchus, Lung Cancers at Ages 55-64

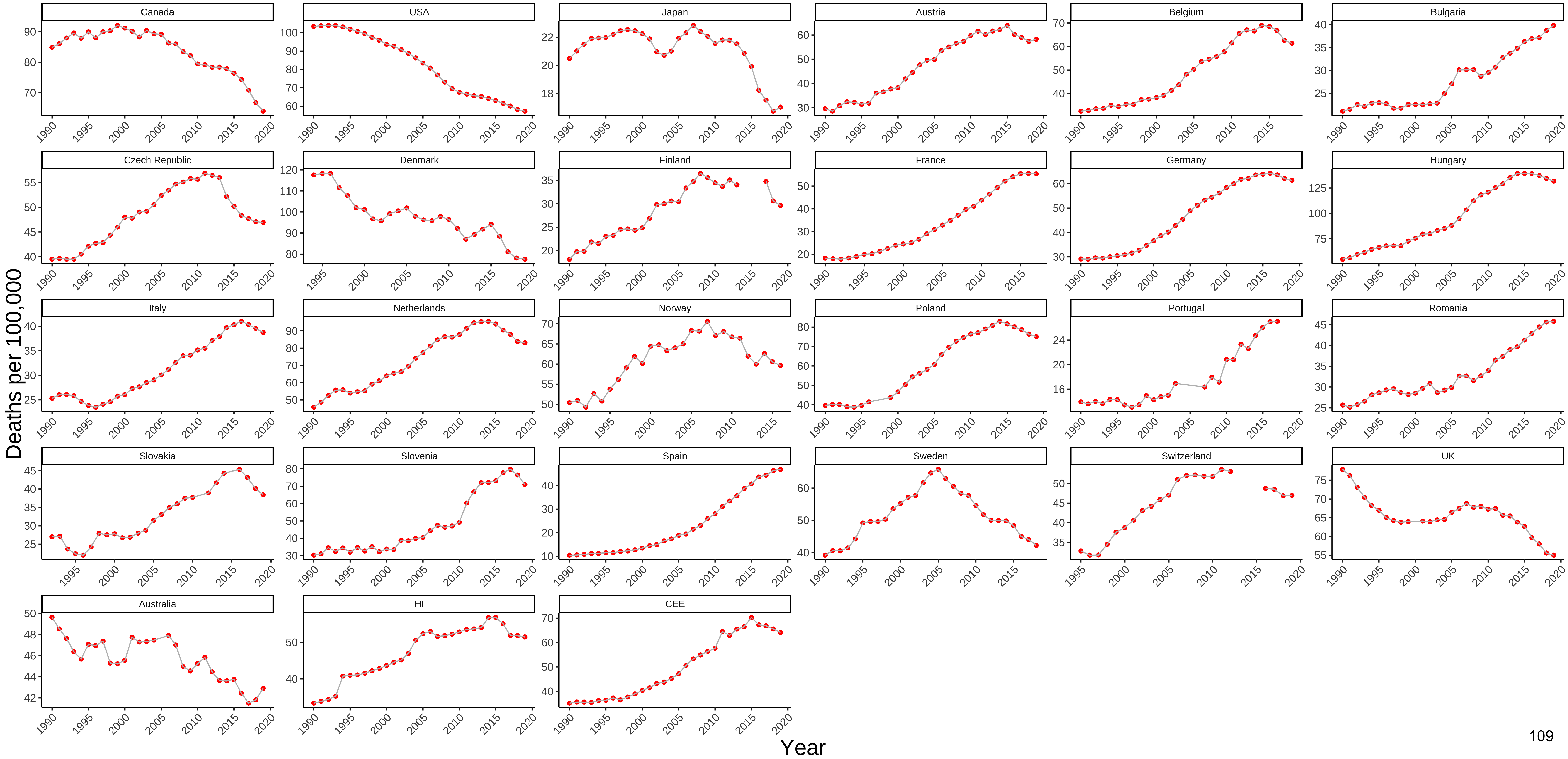

Figure S103. Three-Year Moving Average of Male Mortality from All Other Cancers at Ages 55-64

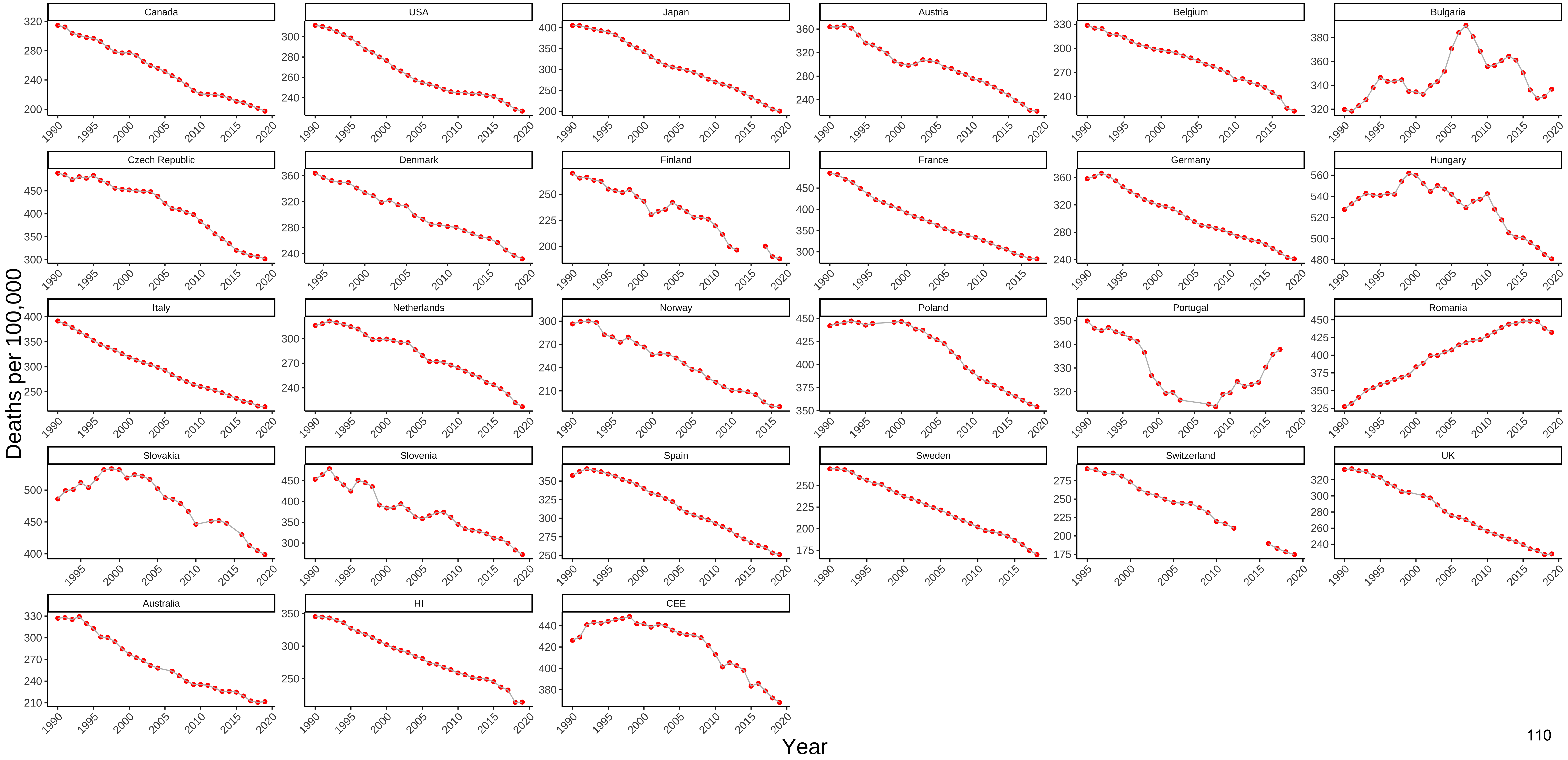

Figure S104. Three-Year Moving Average of Female Mortality from All Other Cancers at Ages 55-64

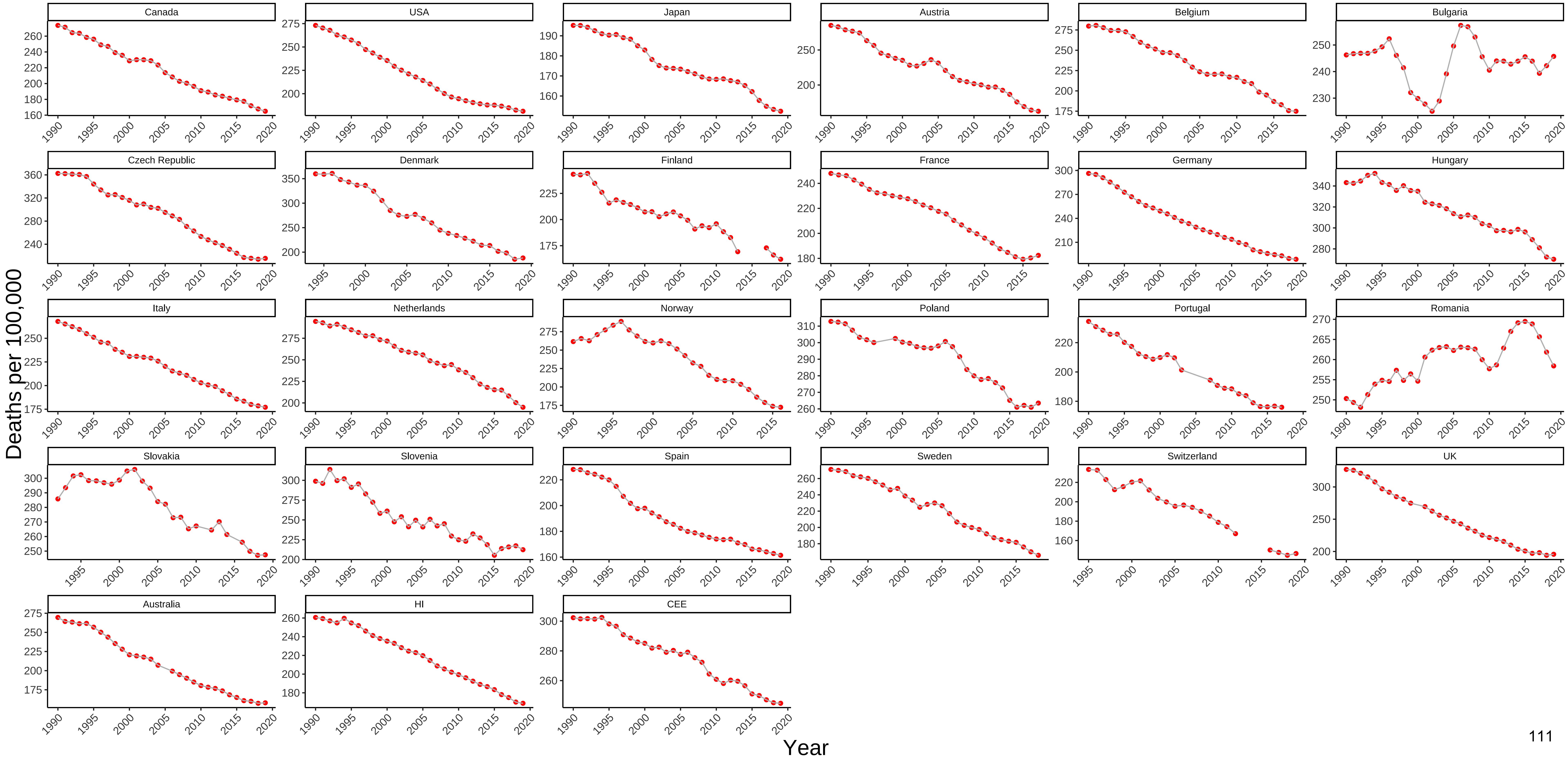

Figure S105. Three-Year Moving Average of Male Mortality from Nervous System Diseases at Ages 55-64

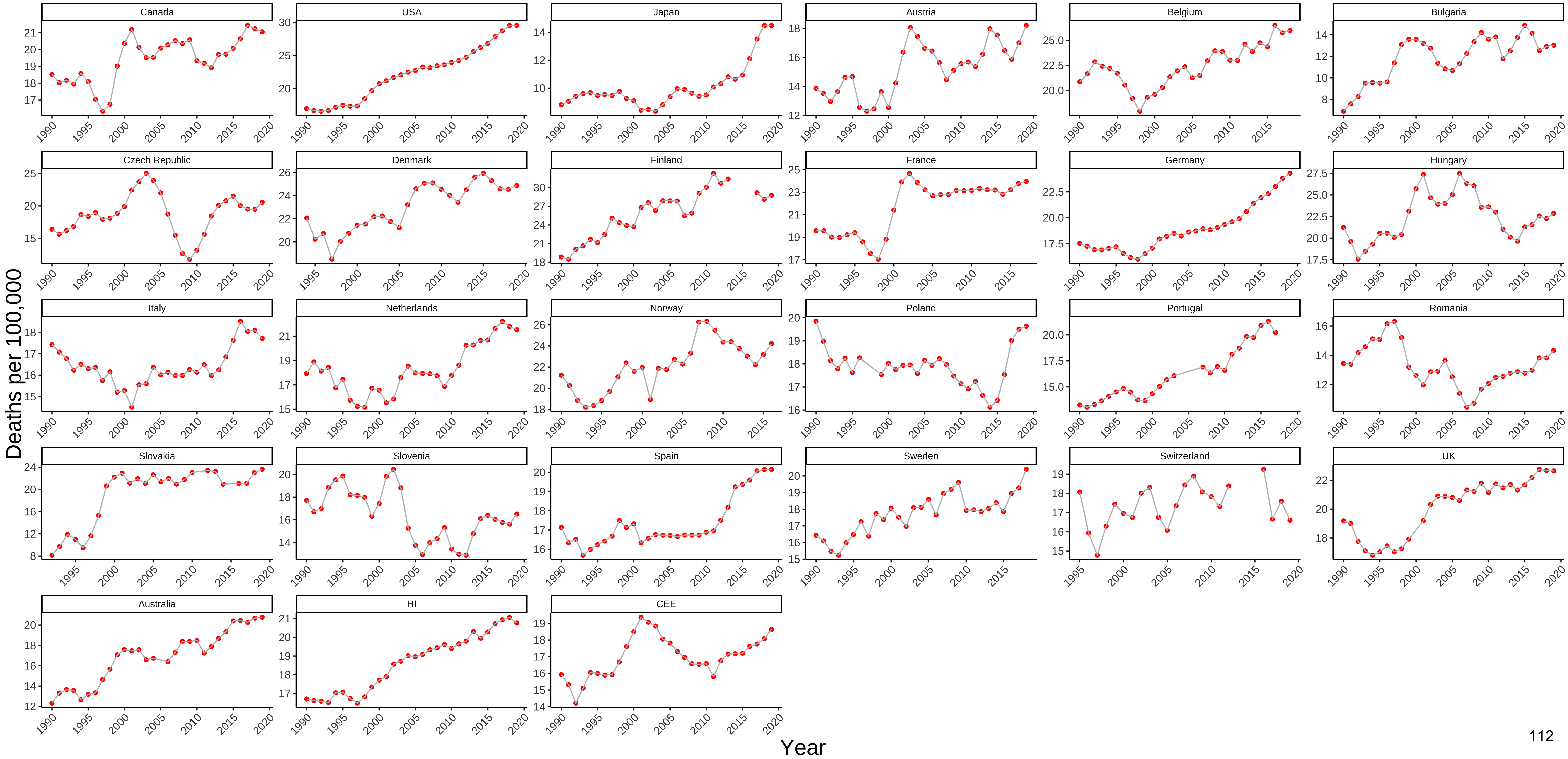

Figure S106. Three-Year Moving Average of Female Mortality from Nervous System Diseases at Ages 55-64

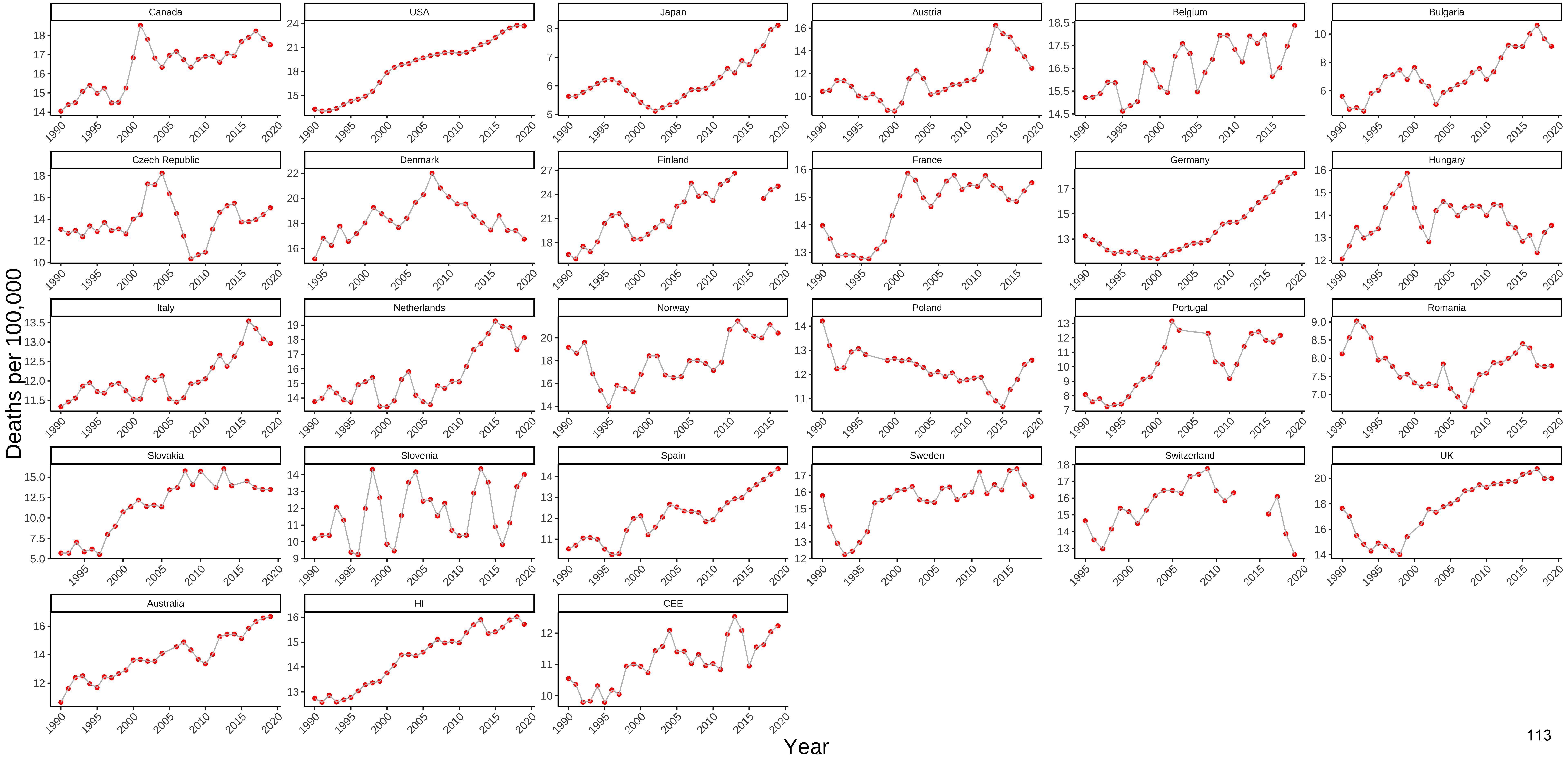

Figure S107. Three-Year Moving Average of Male Mortality from Metabolic Diseases at Ages 55-64

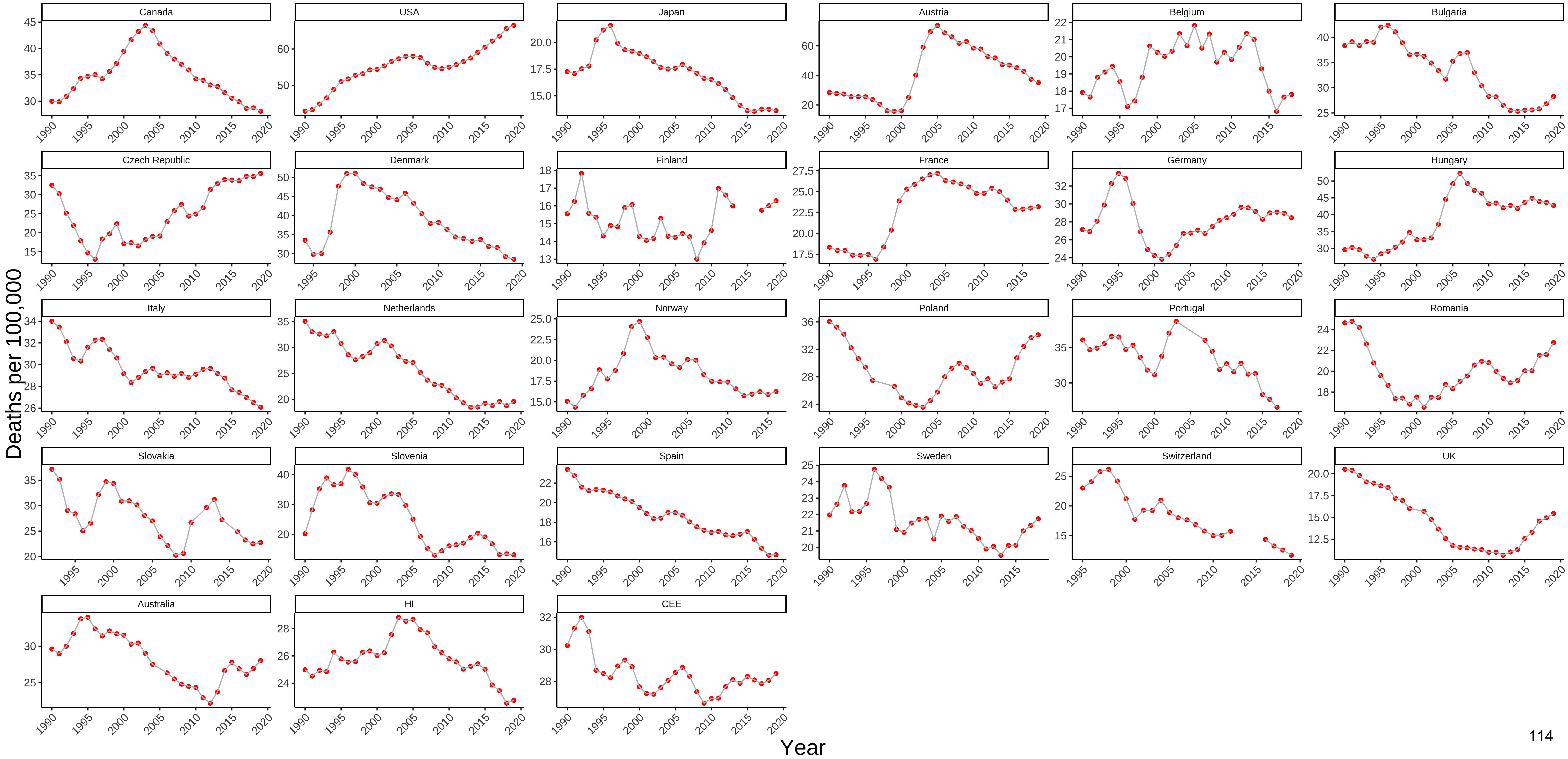

Figure S108. Three-Year Moving Average of Female Mortality from Metabolic Diseases at Ages 55-64

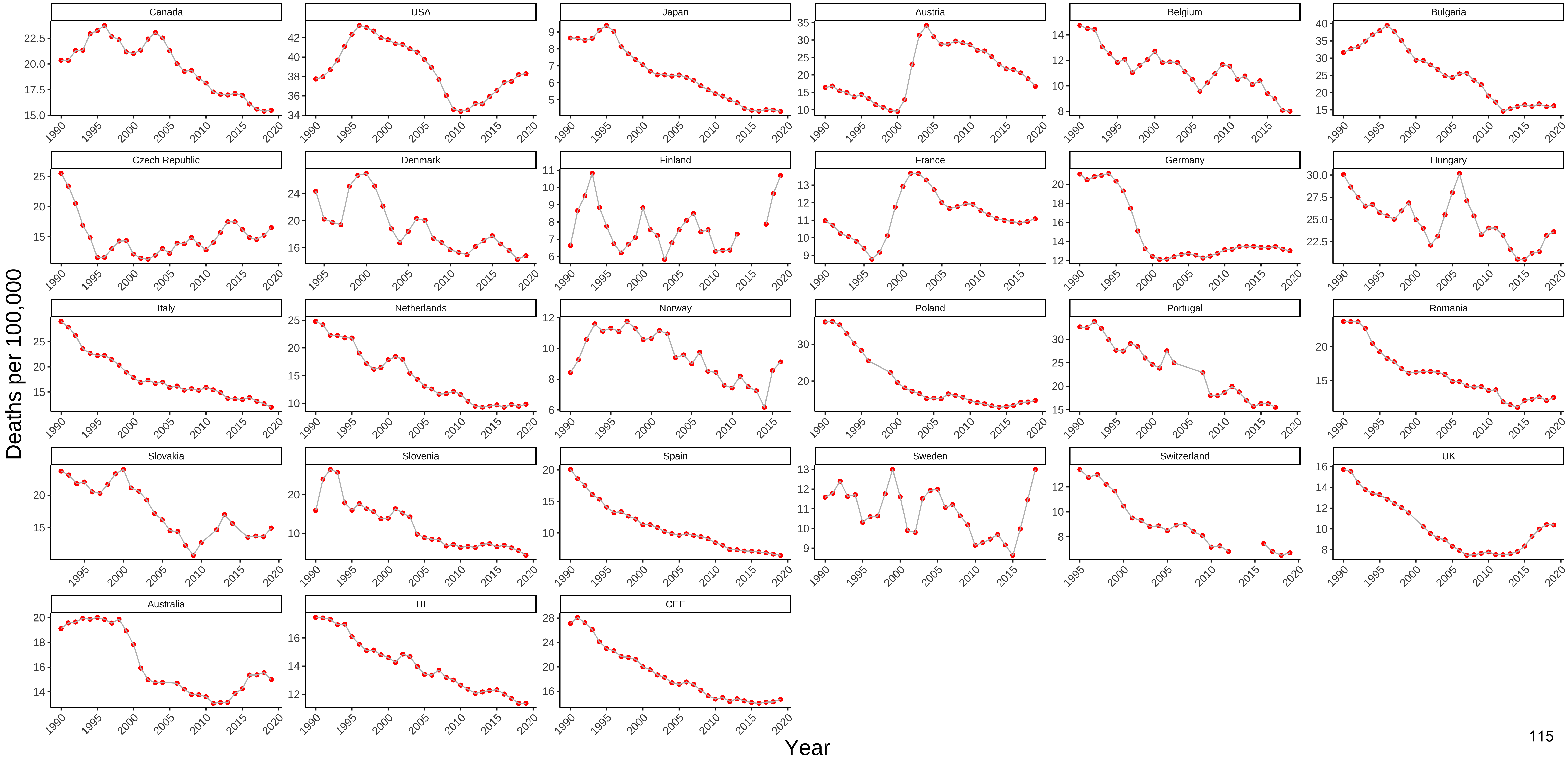

Figure S109. Three-Year Moving Average of Male Mortality from Cardiovascular Disease at Ages 55-64

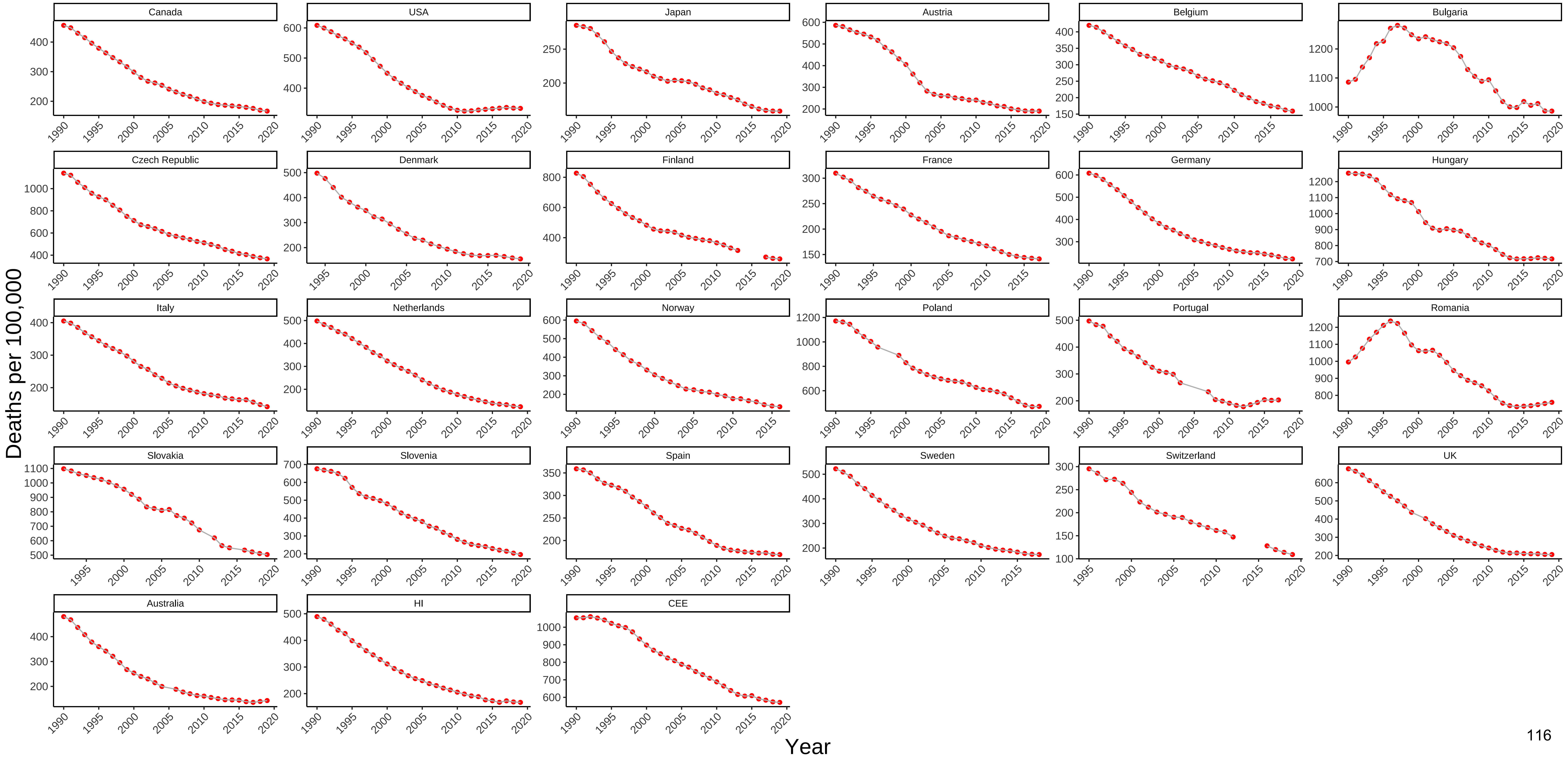

Figure S110. Three-Year Moving Average of Female Mortality from Cardiovascular Disease at Ages 55-64

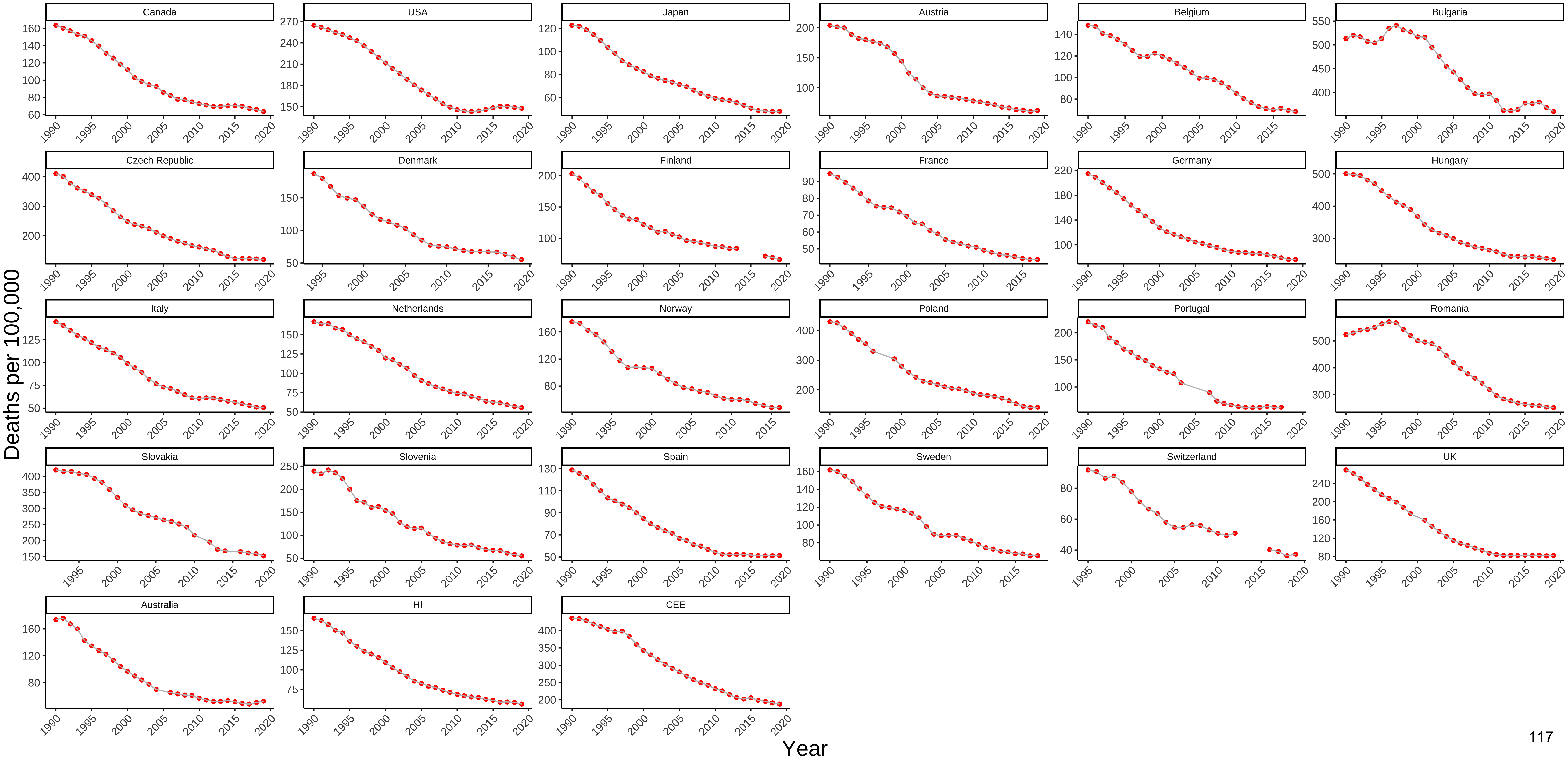

Figure S111. Three-Year Moving Average of Male Mortality from Suicide at Ages 55-64

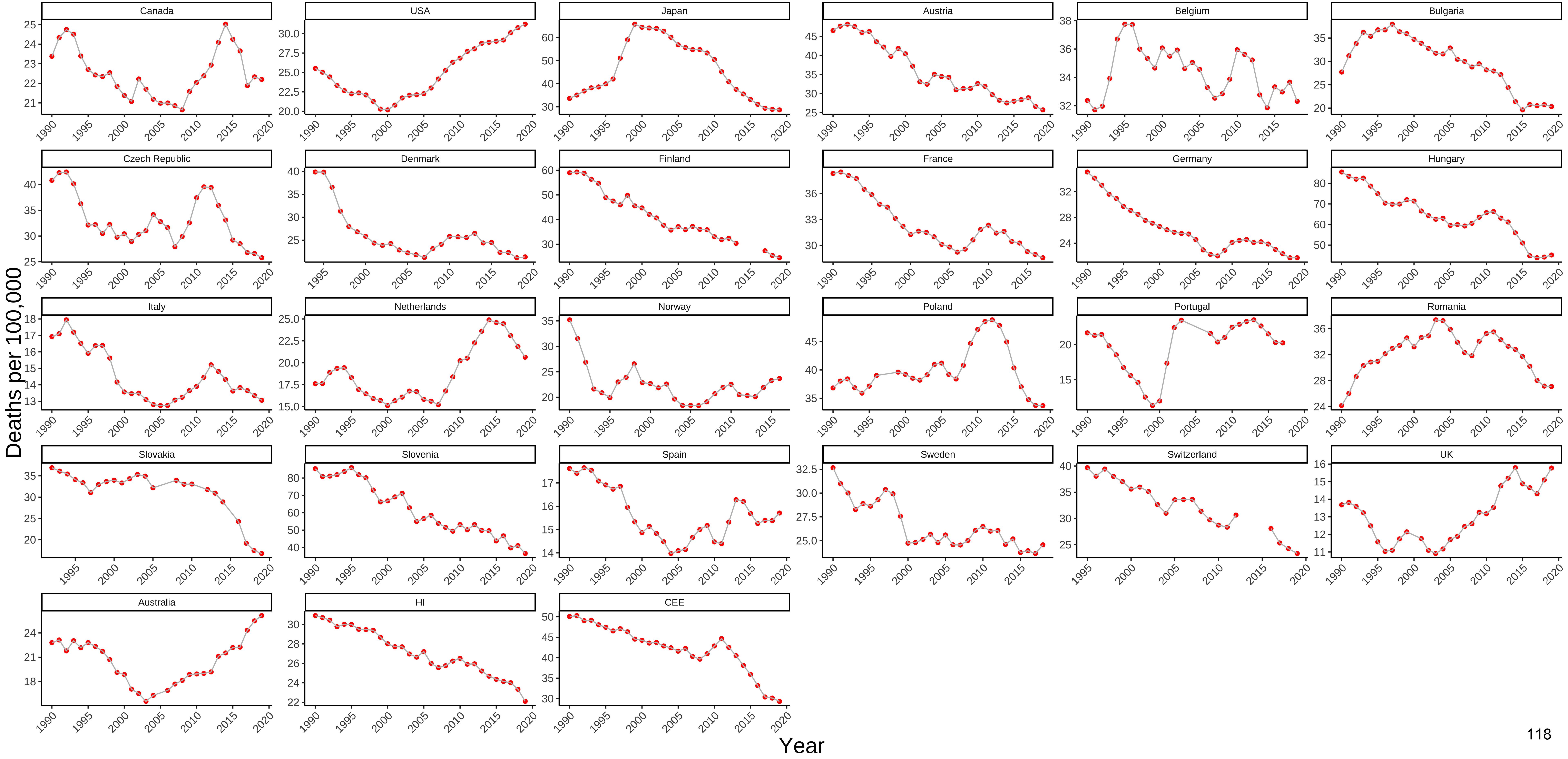

Figure S112. Three-Year Moving Average of Female Mortality from Suicide at Ages 55-64

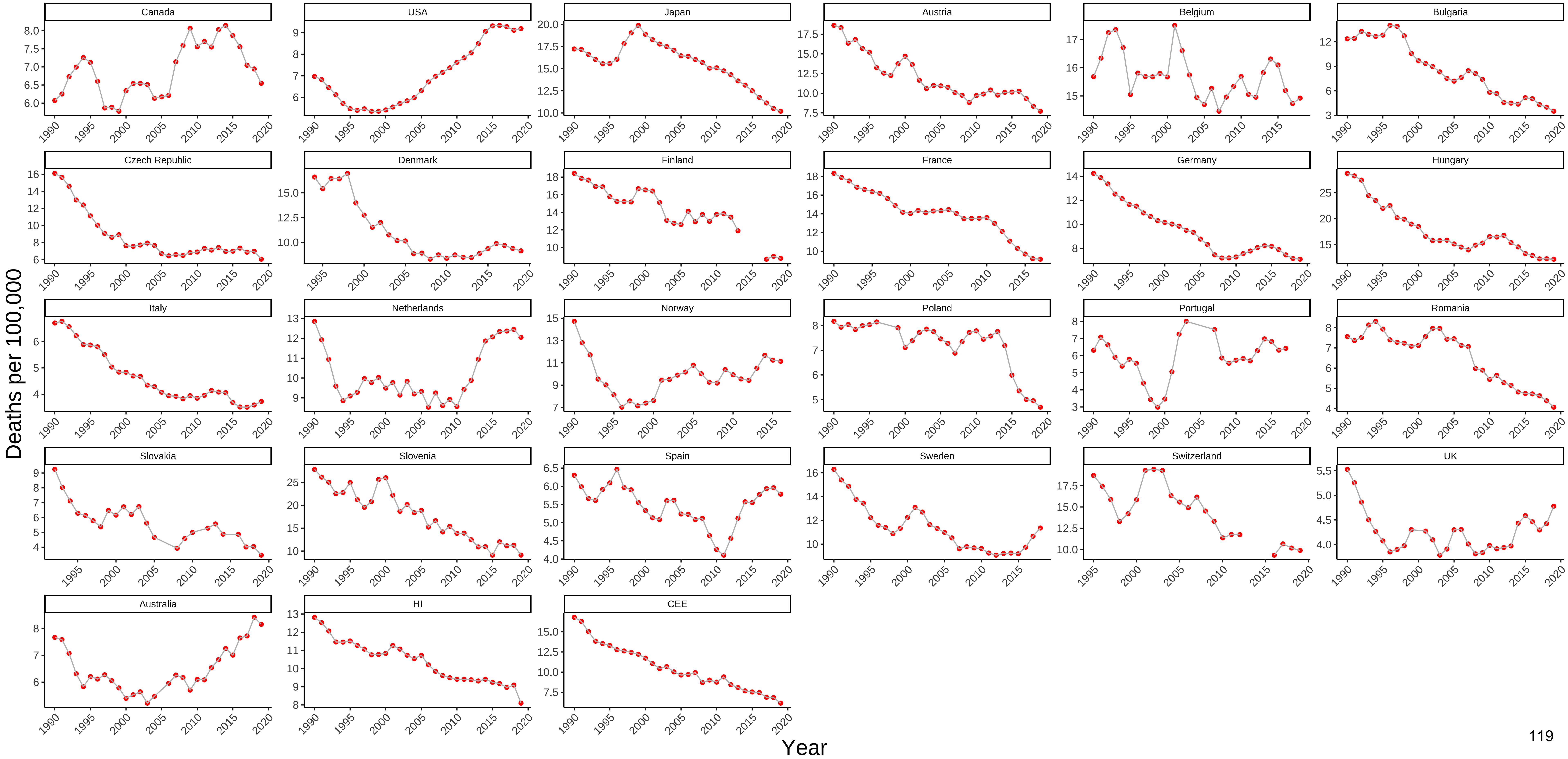

Figure S113. Three-Year Moving Average of Male Mortality from Homicide at Ages 55-64

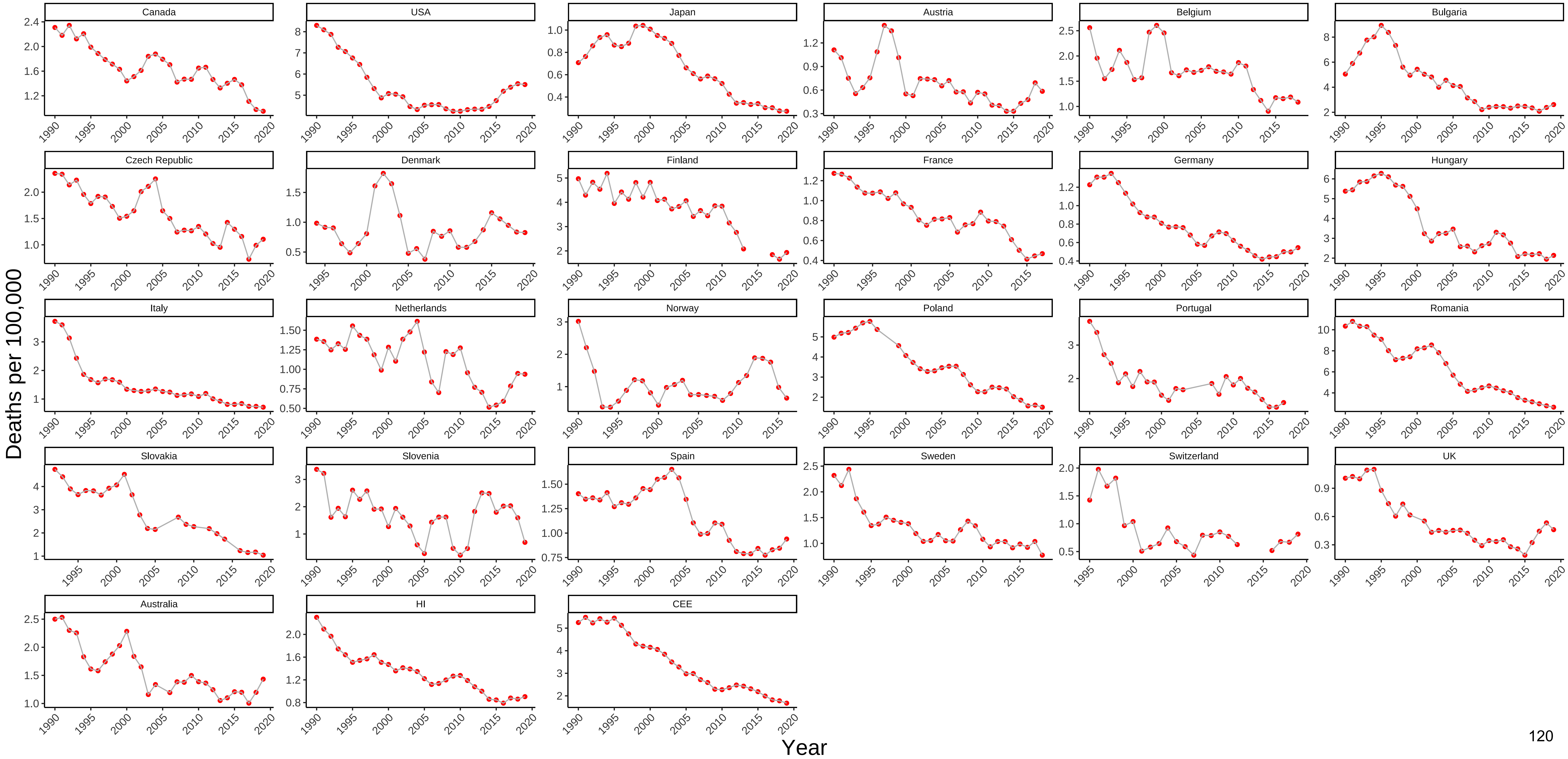

Figure S114. Three-Year Moving Average of Female Mortality from Homicide at Ages 55-64

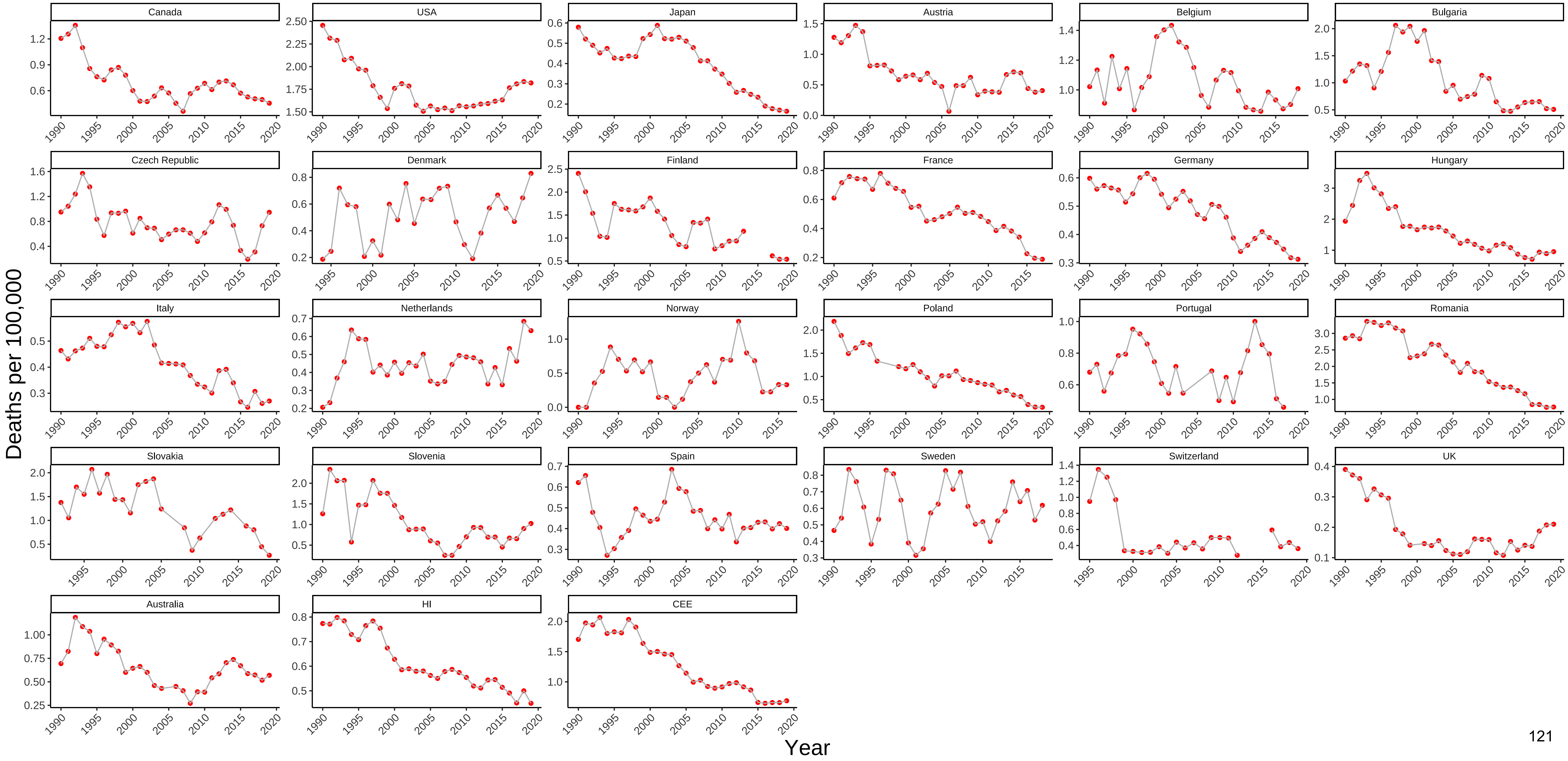

Figure S115. Three-Year Moving Average of Male Mortality from Transport Accidents at Ages 55-64

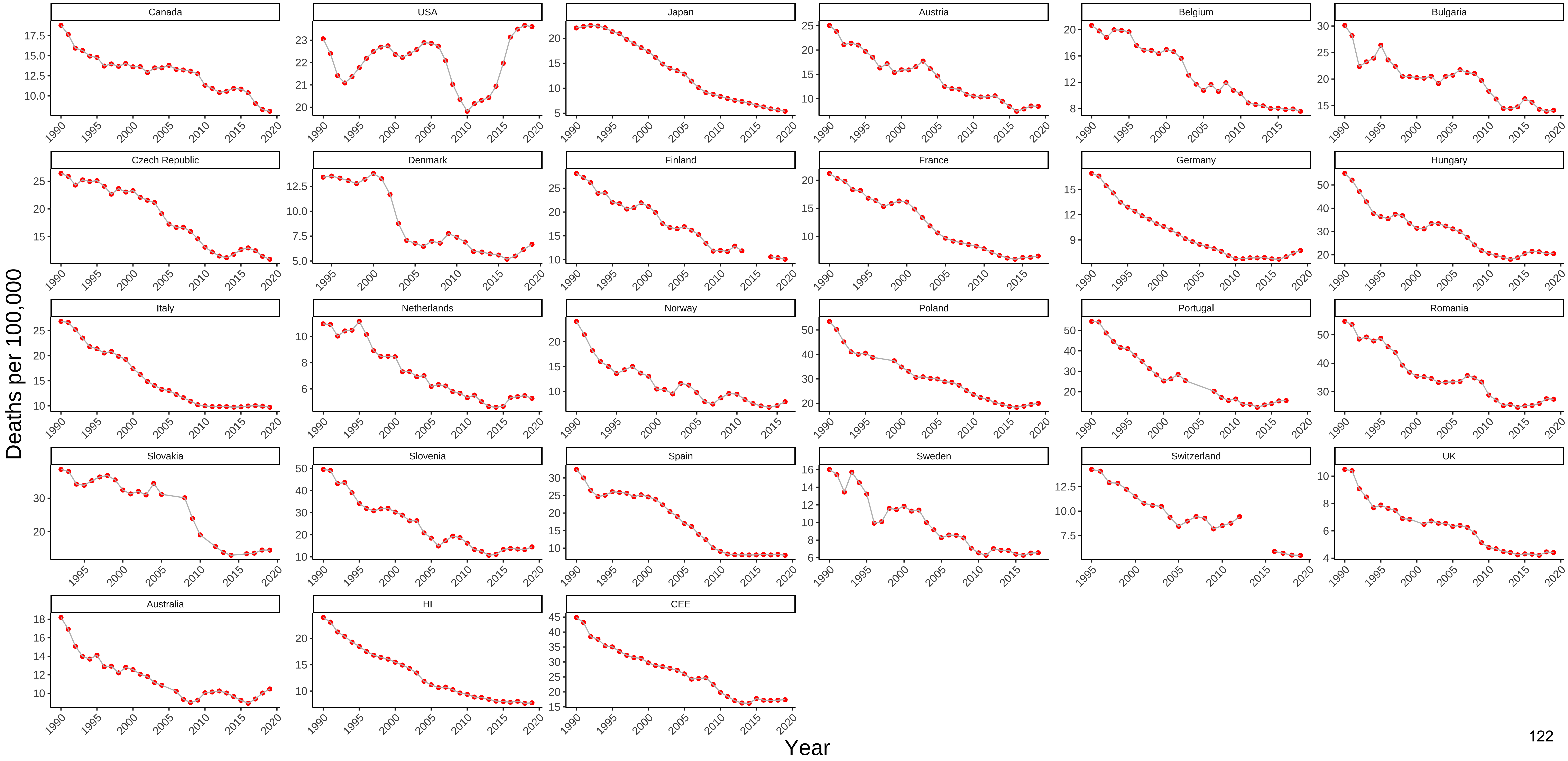

Figure S116. Three-Year Moving Average of Female Mortality from Transport Accidents at Ages 55-64

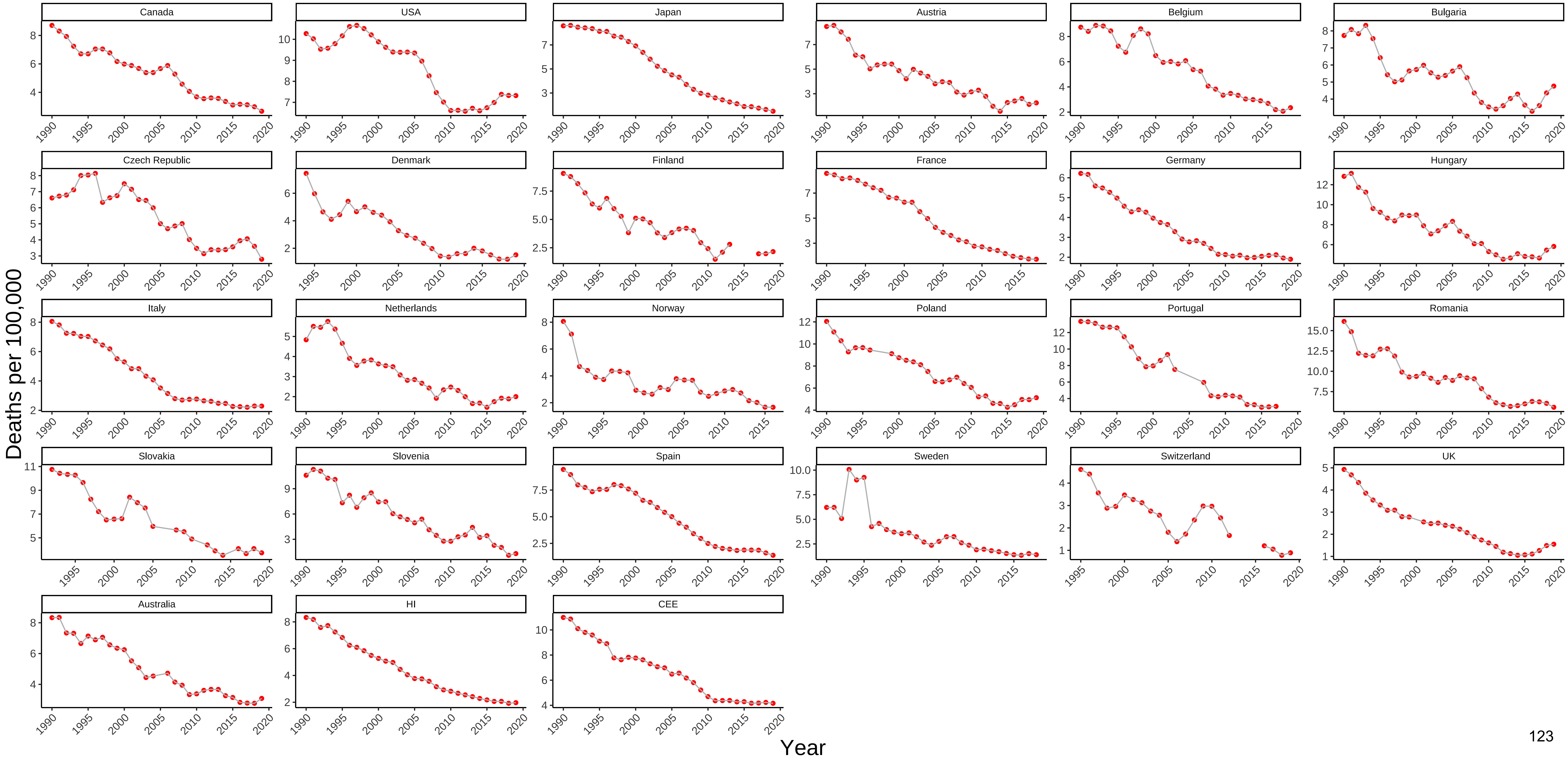

Figure S117. Three-Year Moving Average of Male Mortality from Other External Causes at Ages 55-64

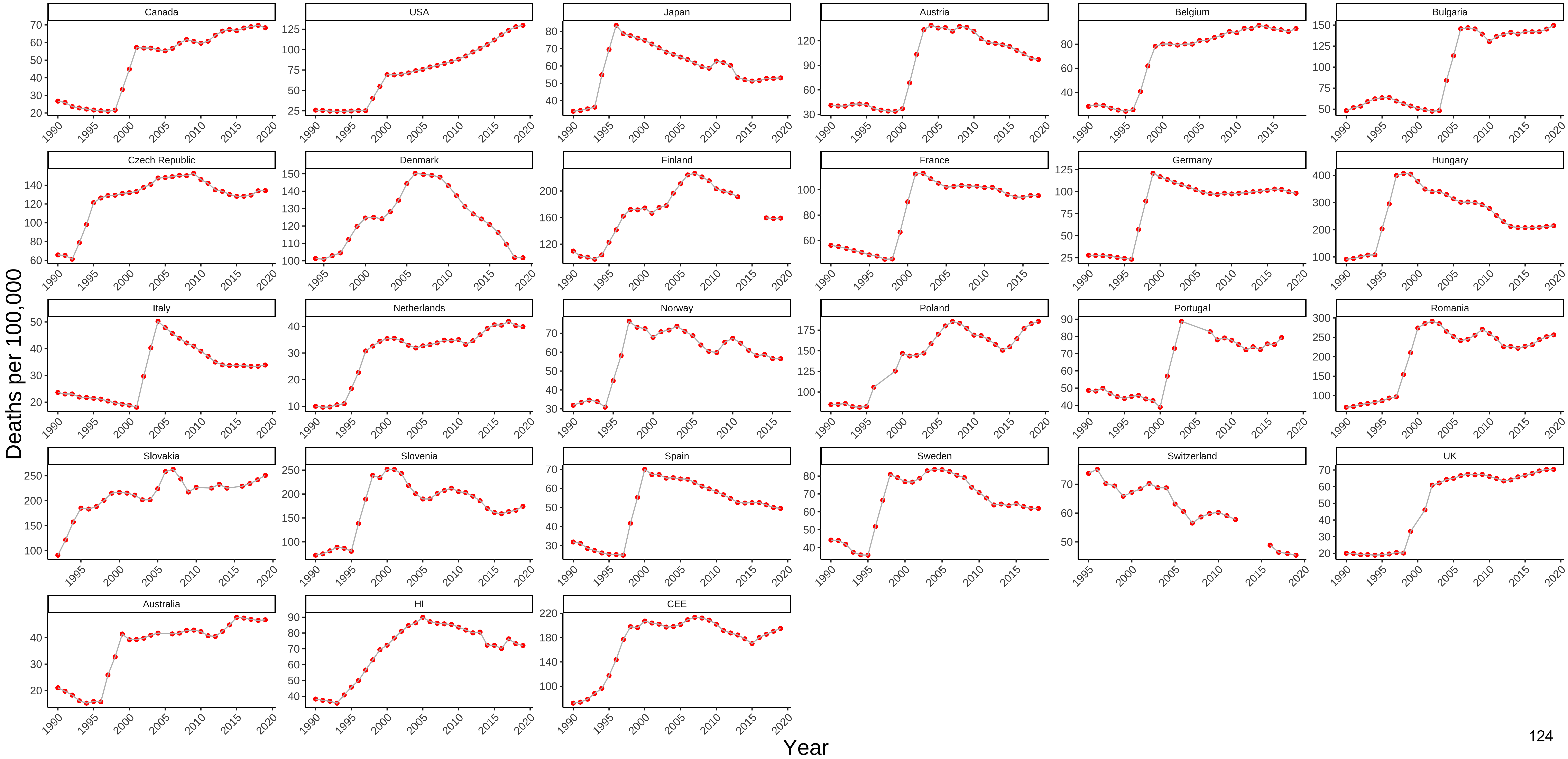

Figure S118. Three-Year Moving Average of Female Mortality from Other External Causes at Ages 55-64

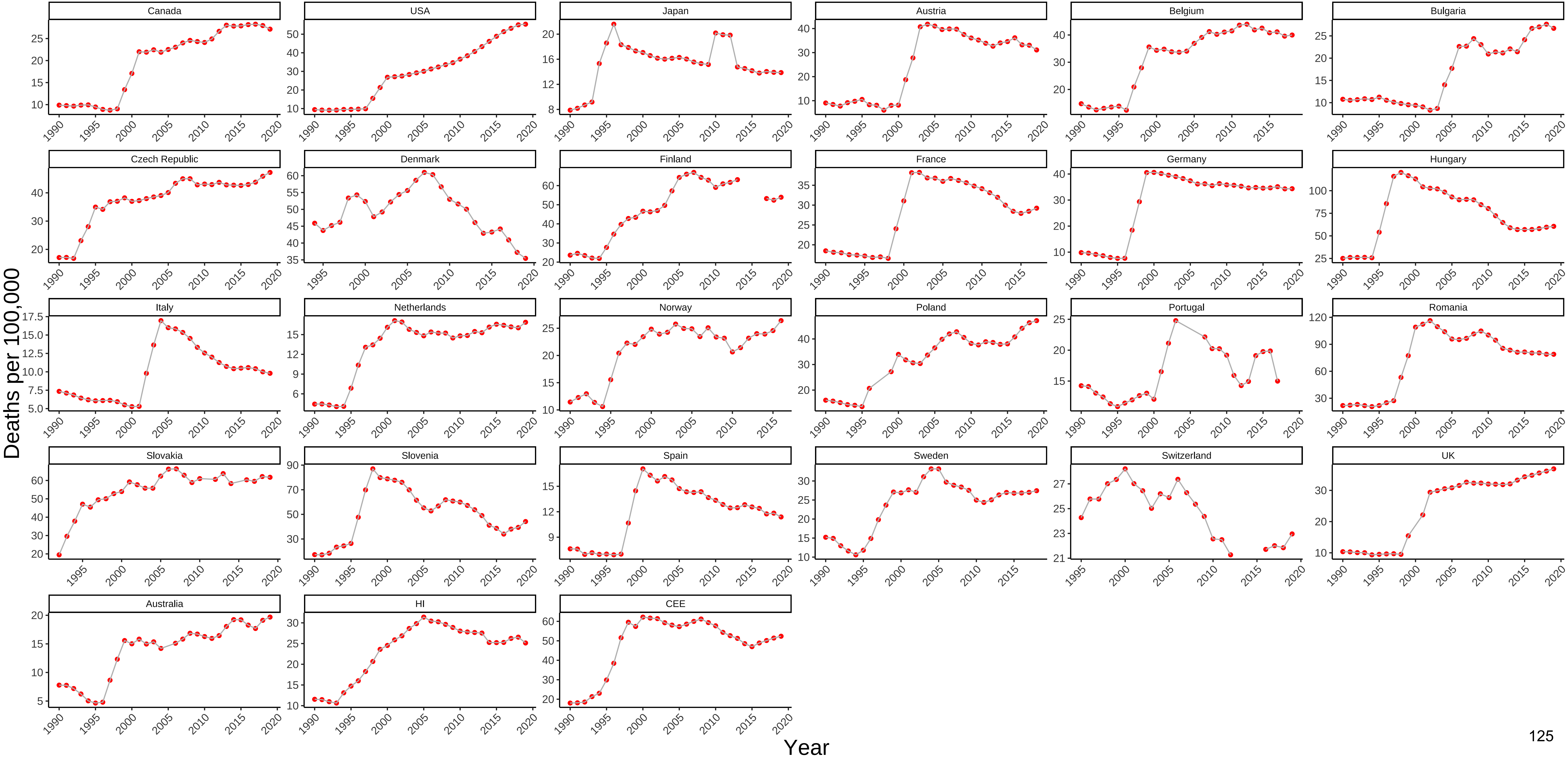

Figure S119. Three-Year Moving Average of Male Mortality from All Other Causes at Ages 55-64

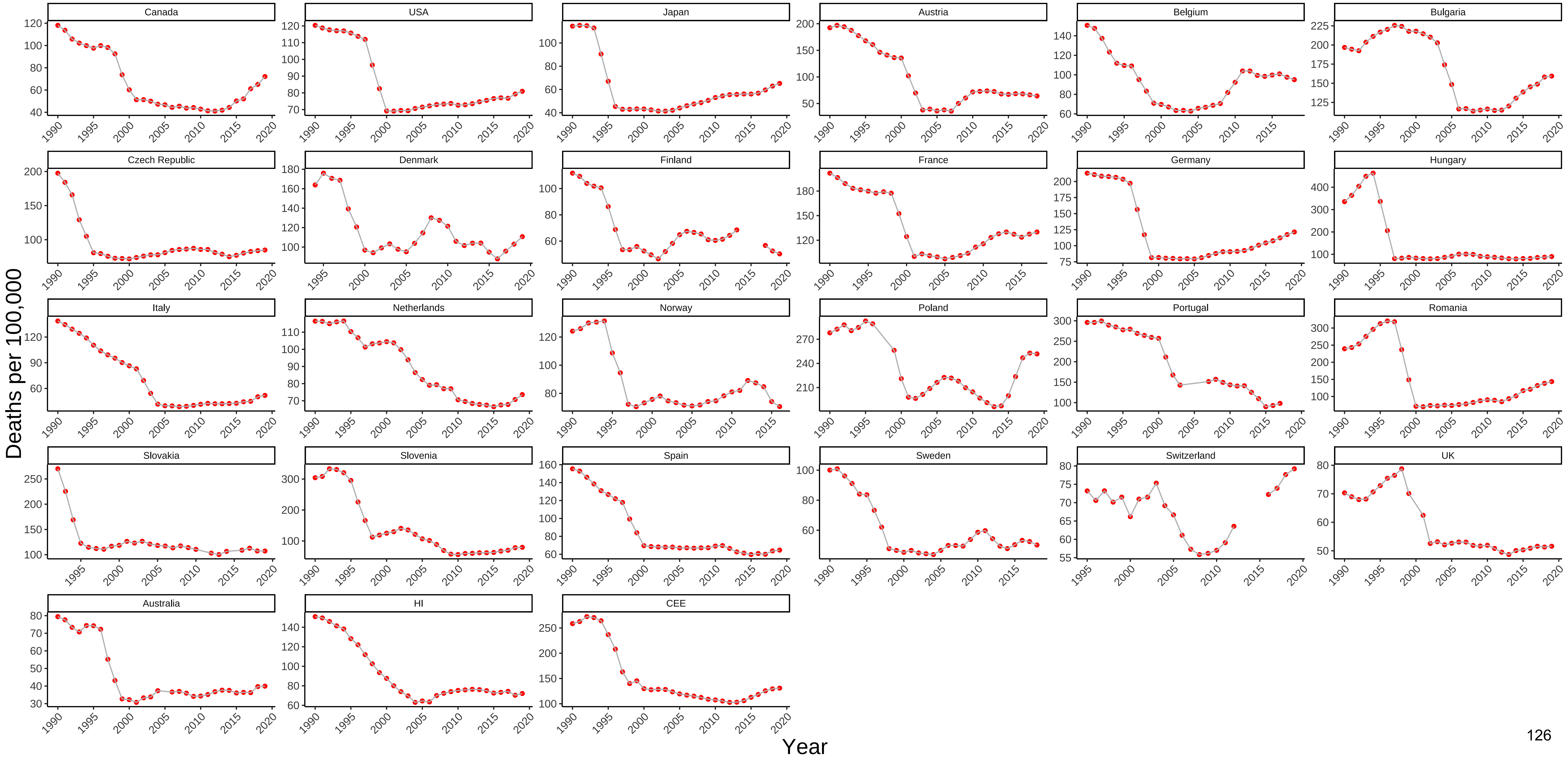

Figure S120. Three-Year Moving Average of Female Mortality from All Other Causes at Ages 55-64

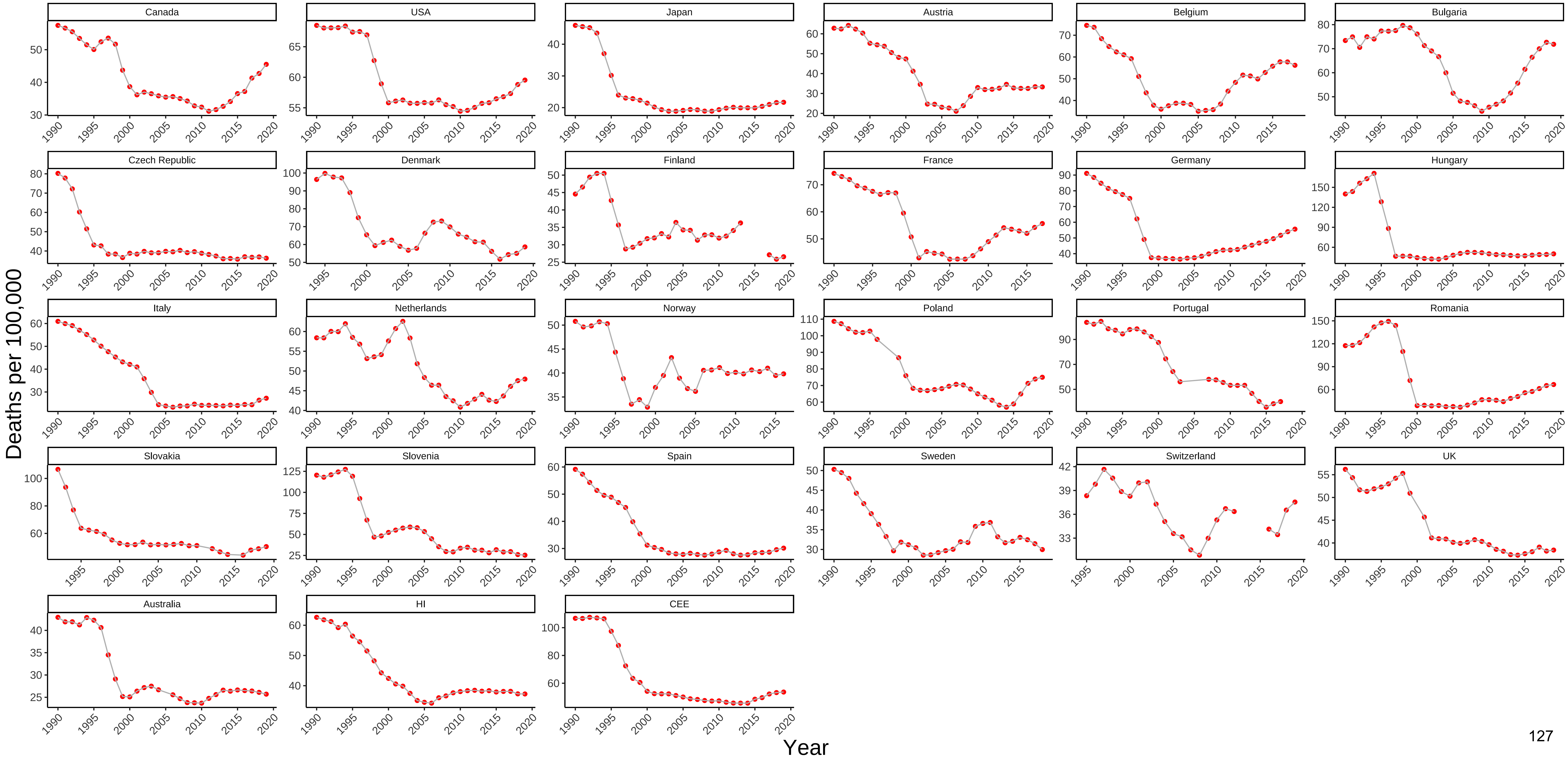

Figure S121. Three-Year Moving Average of Male Mortality from All Causes at Ages 55-64

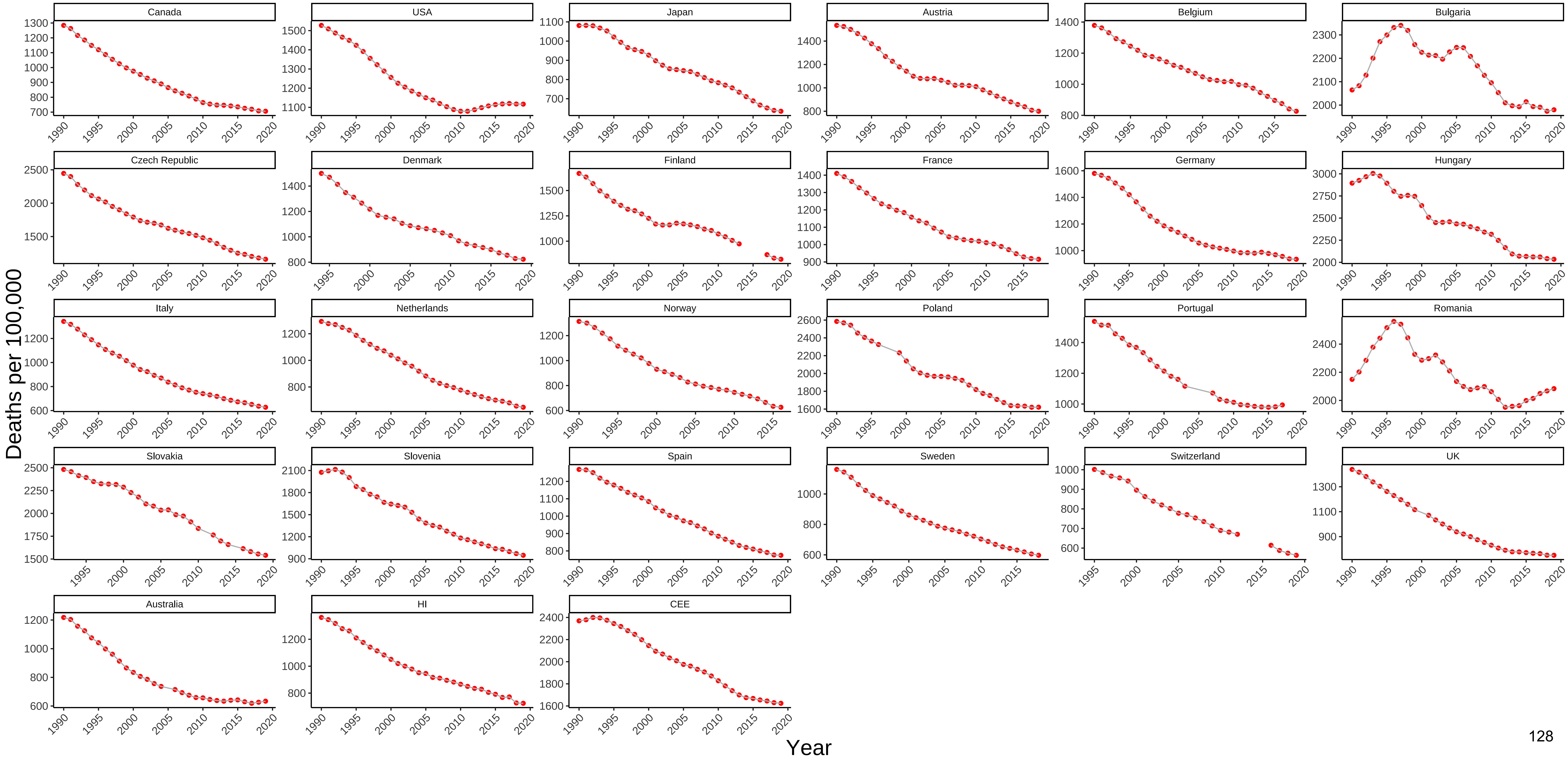

Figure S122. Three-Year Moving Average of Female Mortality from All Causes at Ages 55-64

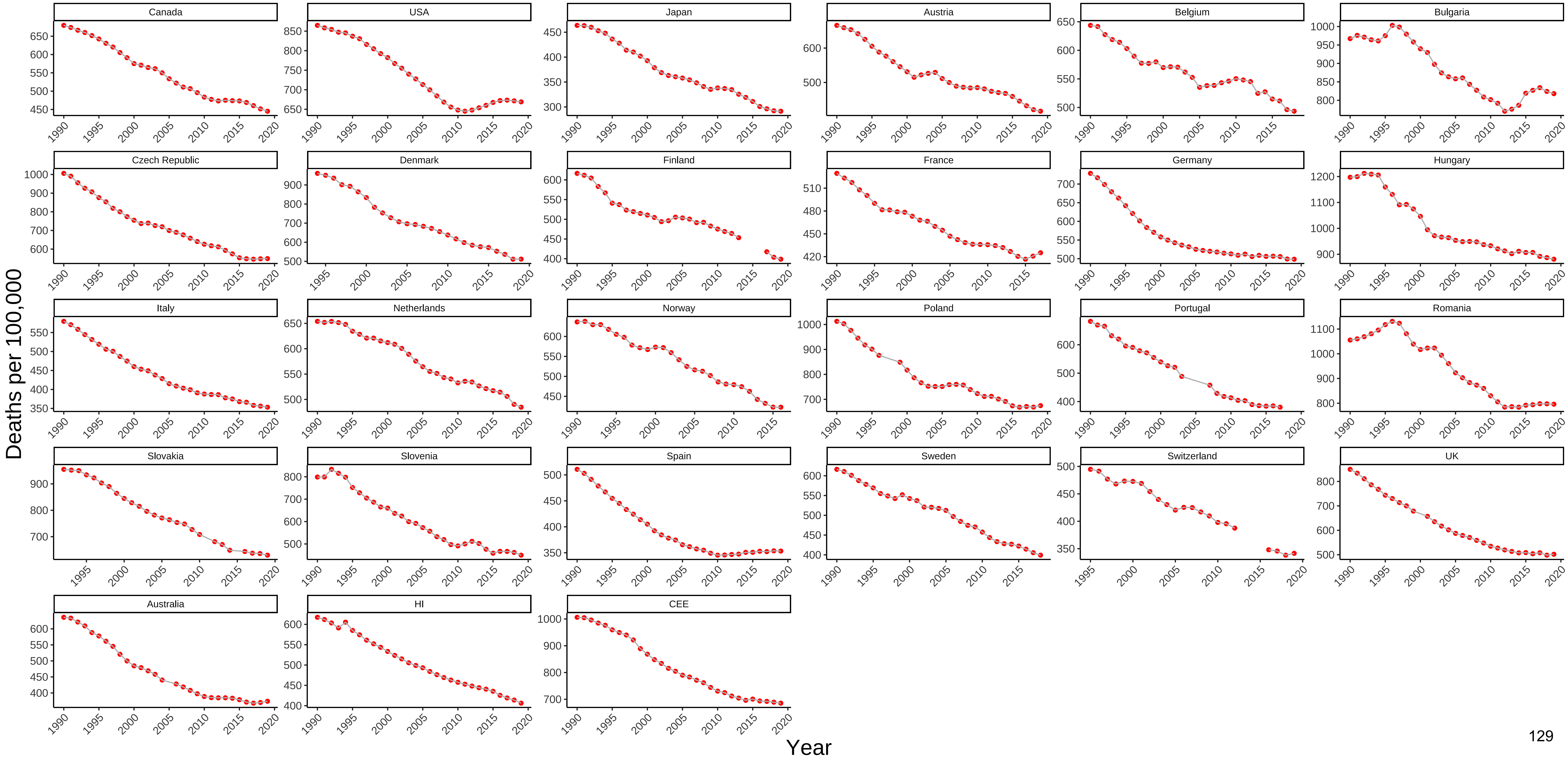

Figure S123. Three-Year Moving Average of Male Mortality from Drug-Related Causes at Ages 25-44

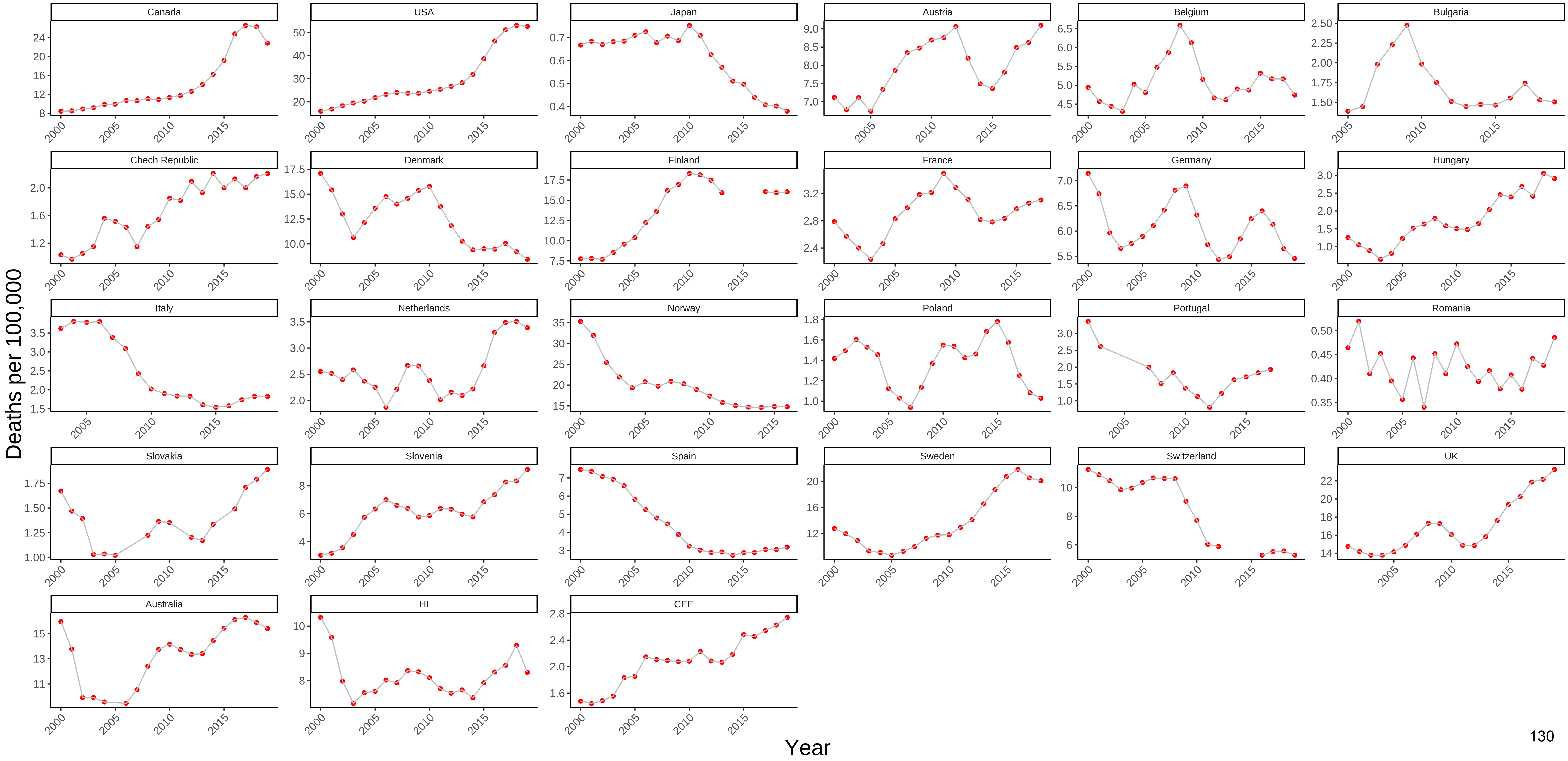

Figure S124. Three-Year Moving Average of Female Mortality from Drug-Related Causes at Ages 25-44

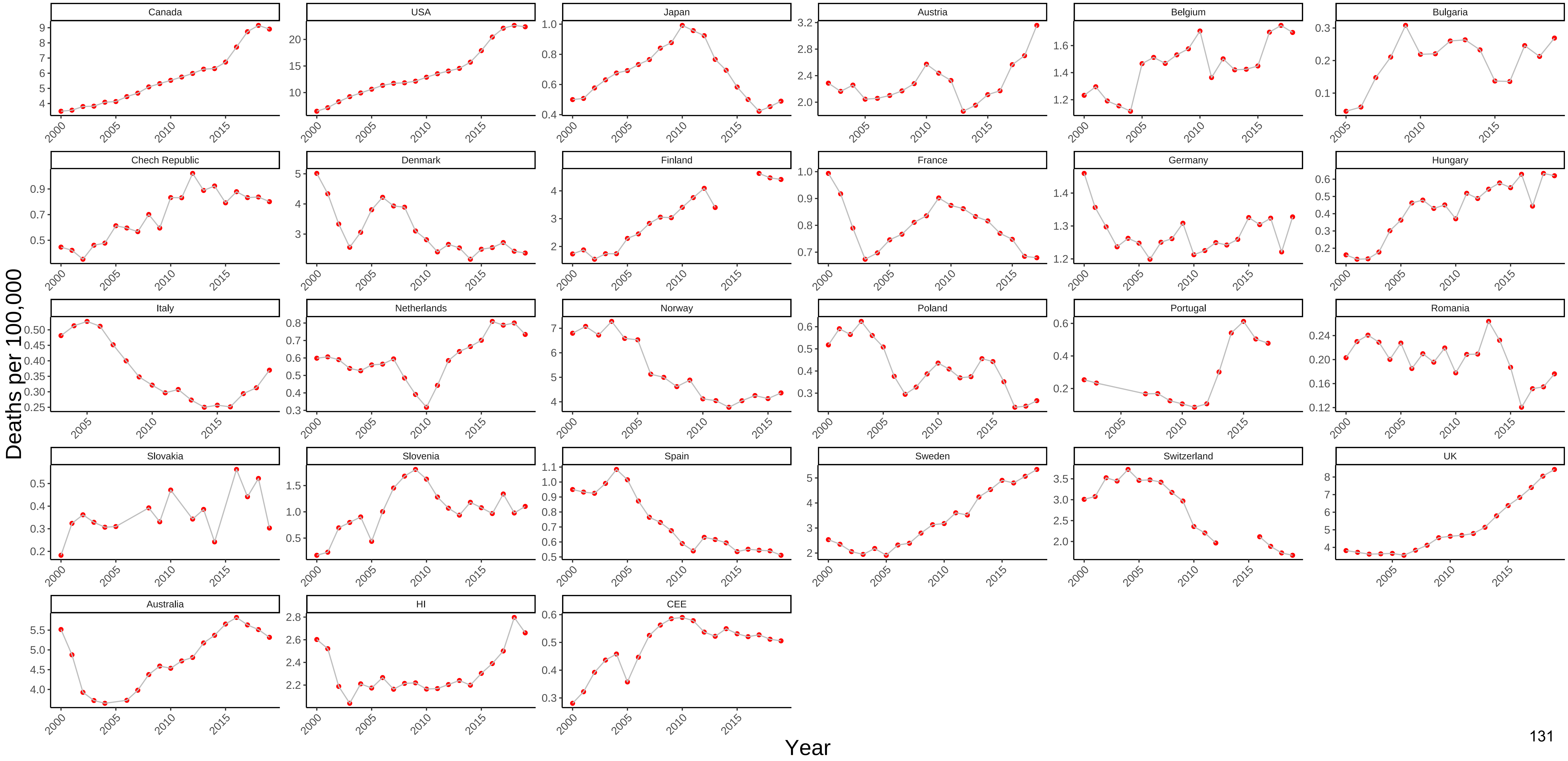

Figure S125. Three-Year Moving Average of Male Mortality from Alcohol-Related Causes at Ages 25-44

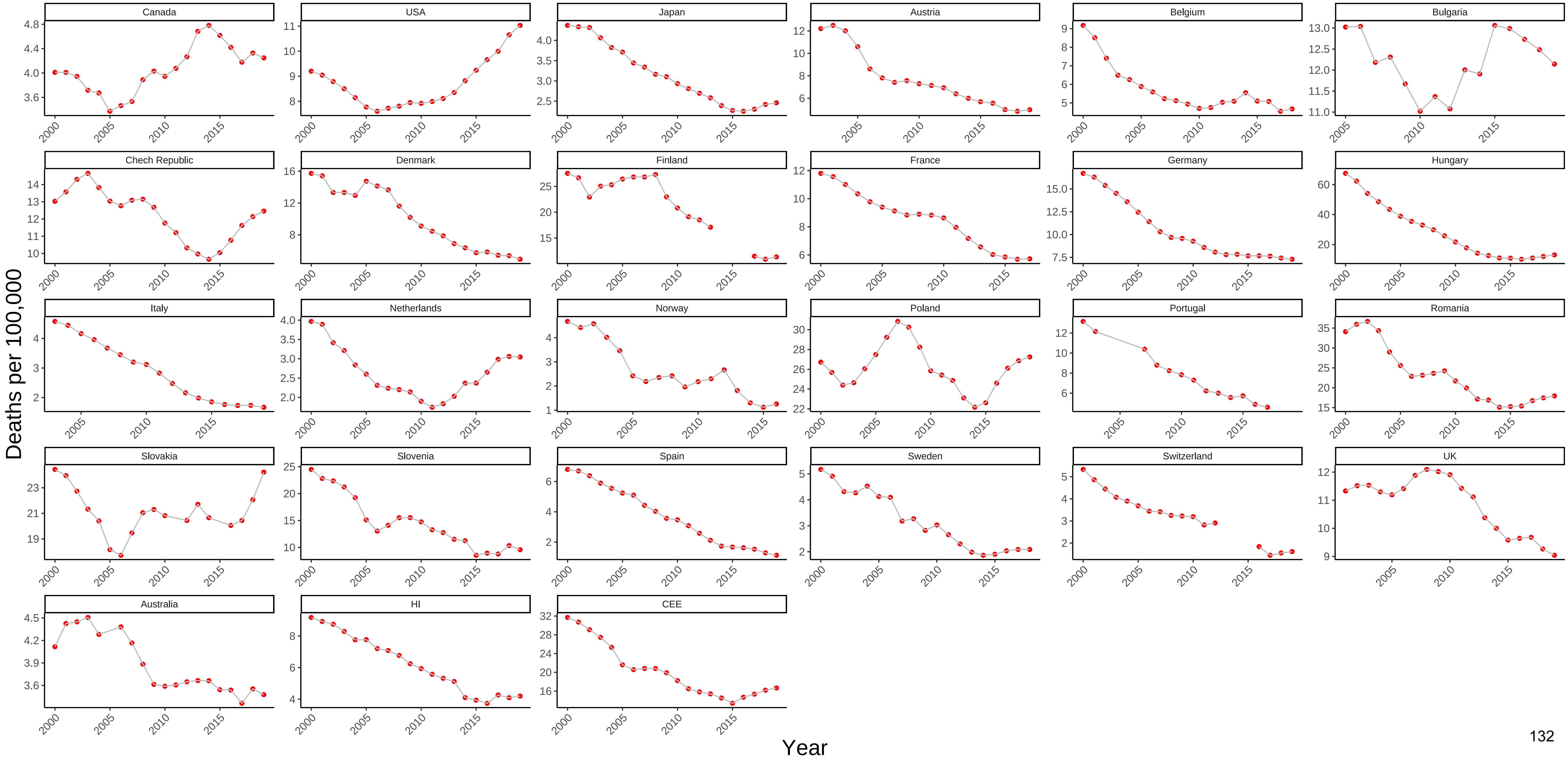

Figure S126. Three-Year Moving Average of Female Mortality from Alcohol-Related Causes at Ages 25-44

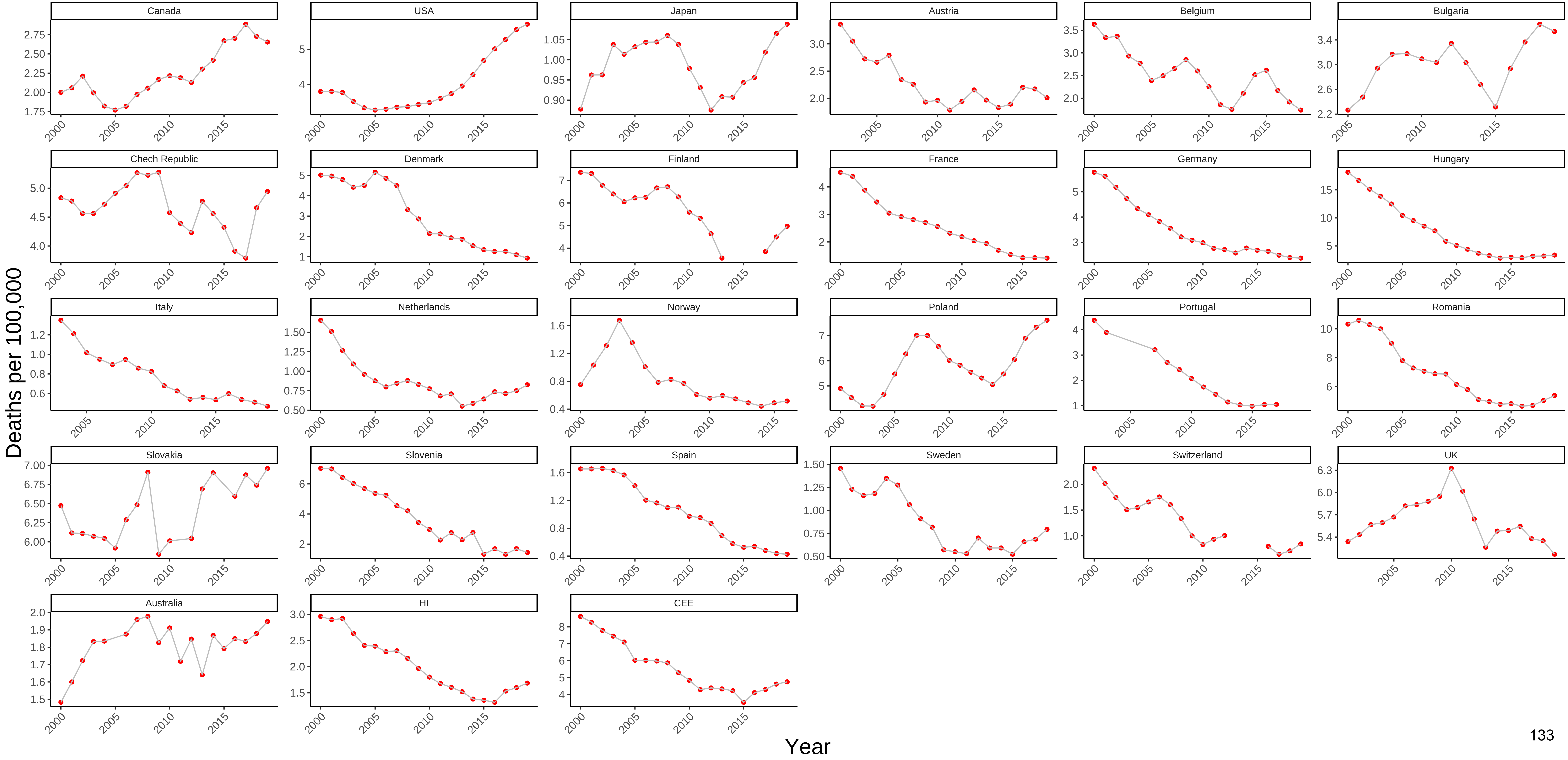

Figure S127. Three-Year Moving Average of Male Mortality from Drug-Related Causes at Ages 45-54

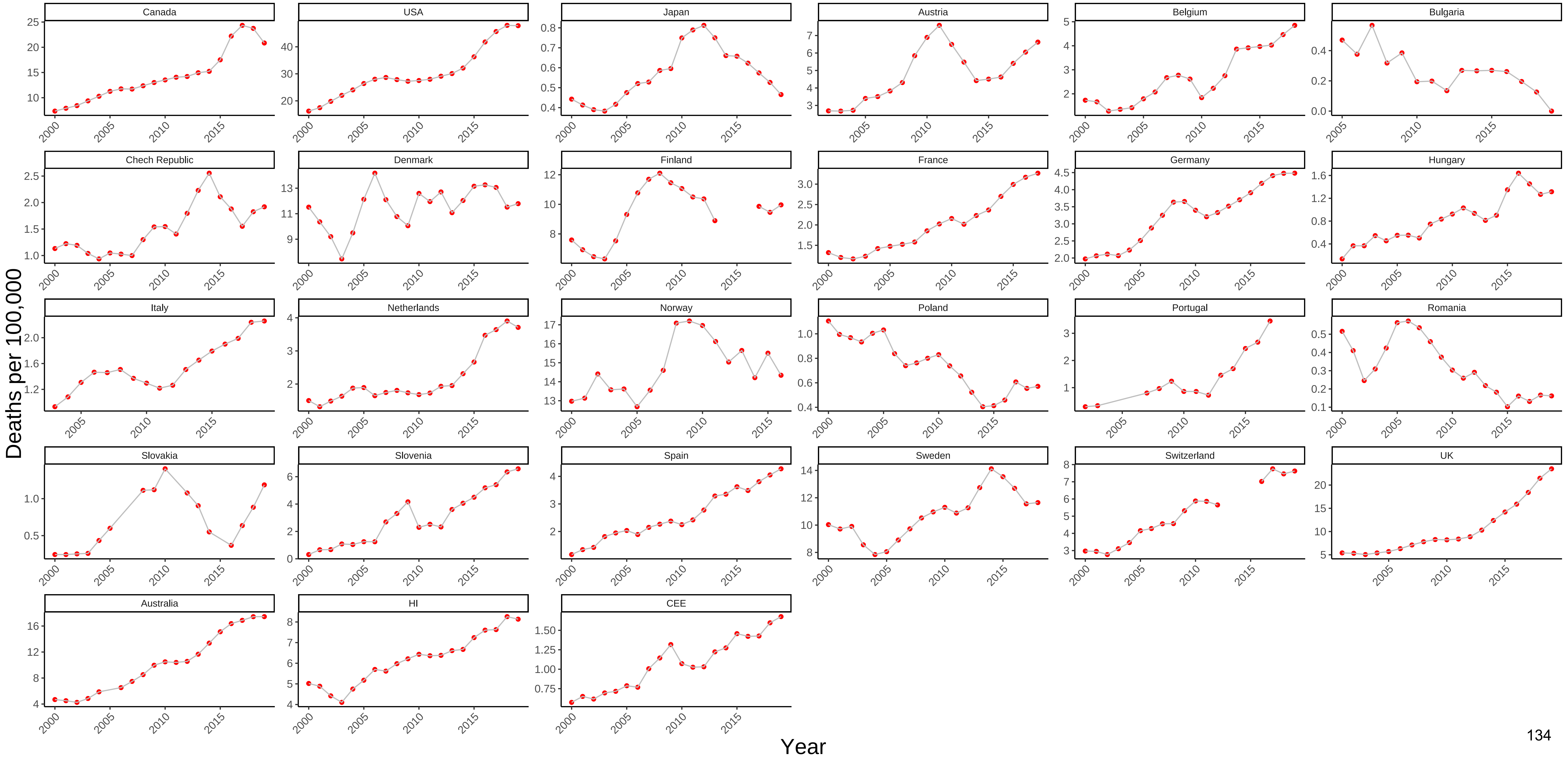

Figure S128. Three-Year Moving Average of Female Mortality from Drug-Related Causes at Ages 45-54

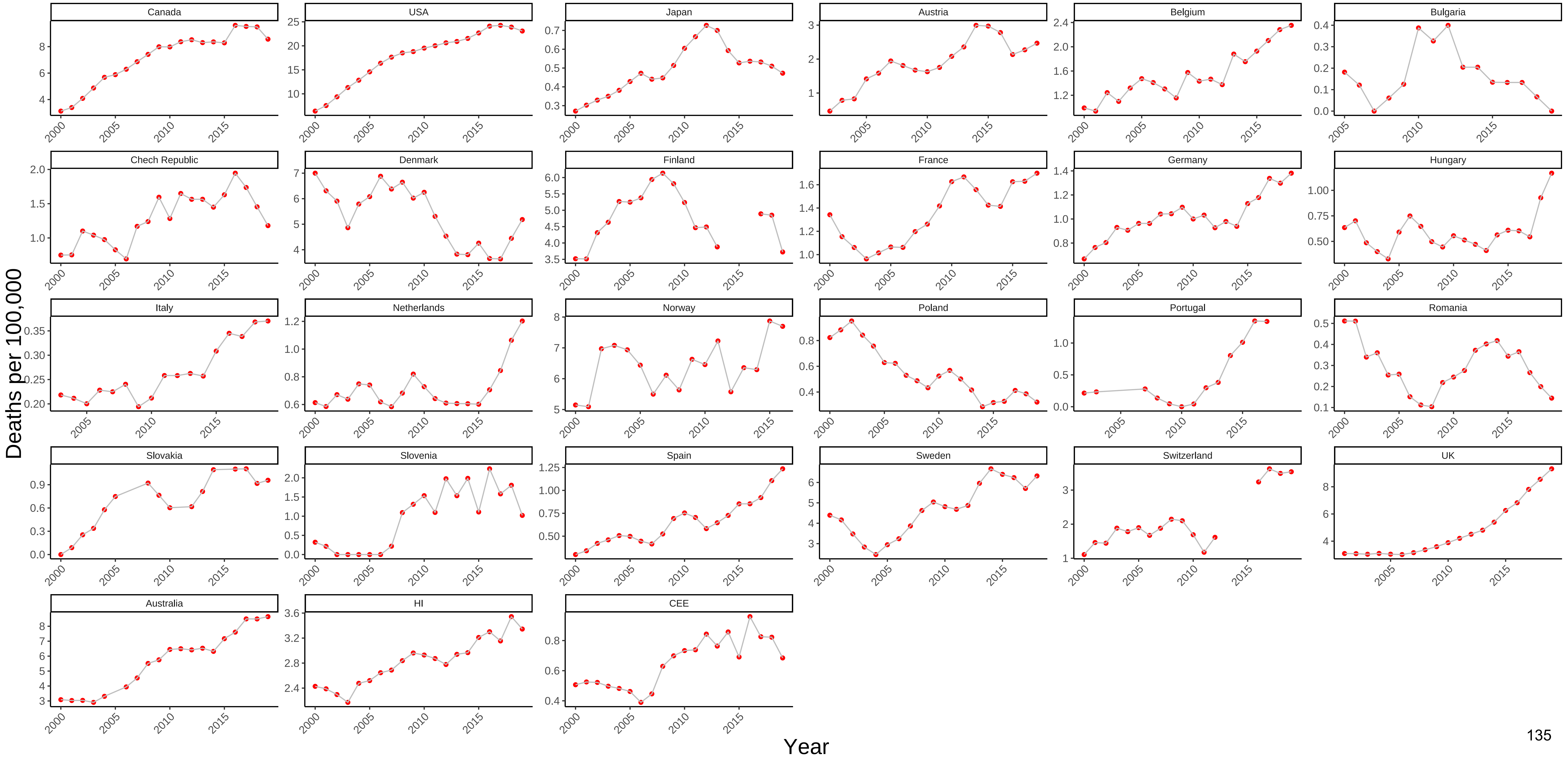

Figure S129. Three-Year Moving Average of Male Mortality from Alcohol-Related Causes at Ages 45-54

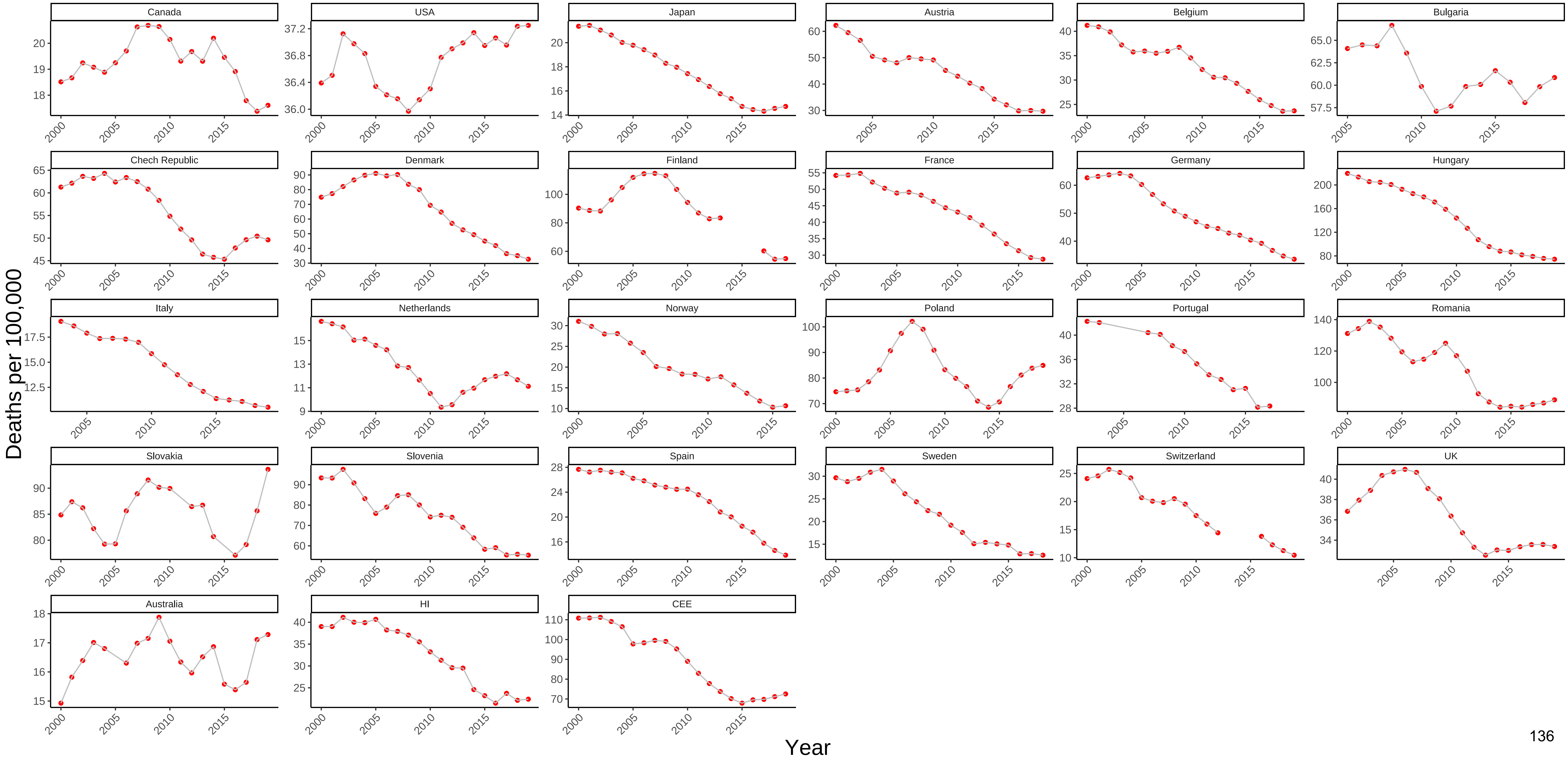

Figure S130. Three-Year Moving Average of Female Mortality from Alcohol-Related Causes at Ages 45-54

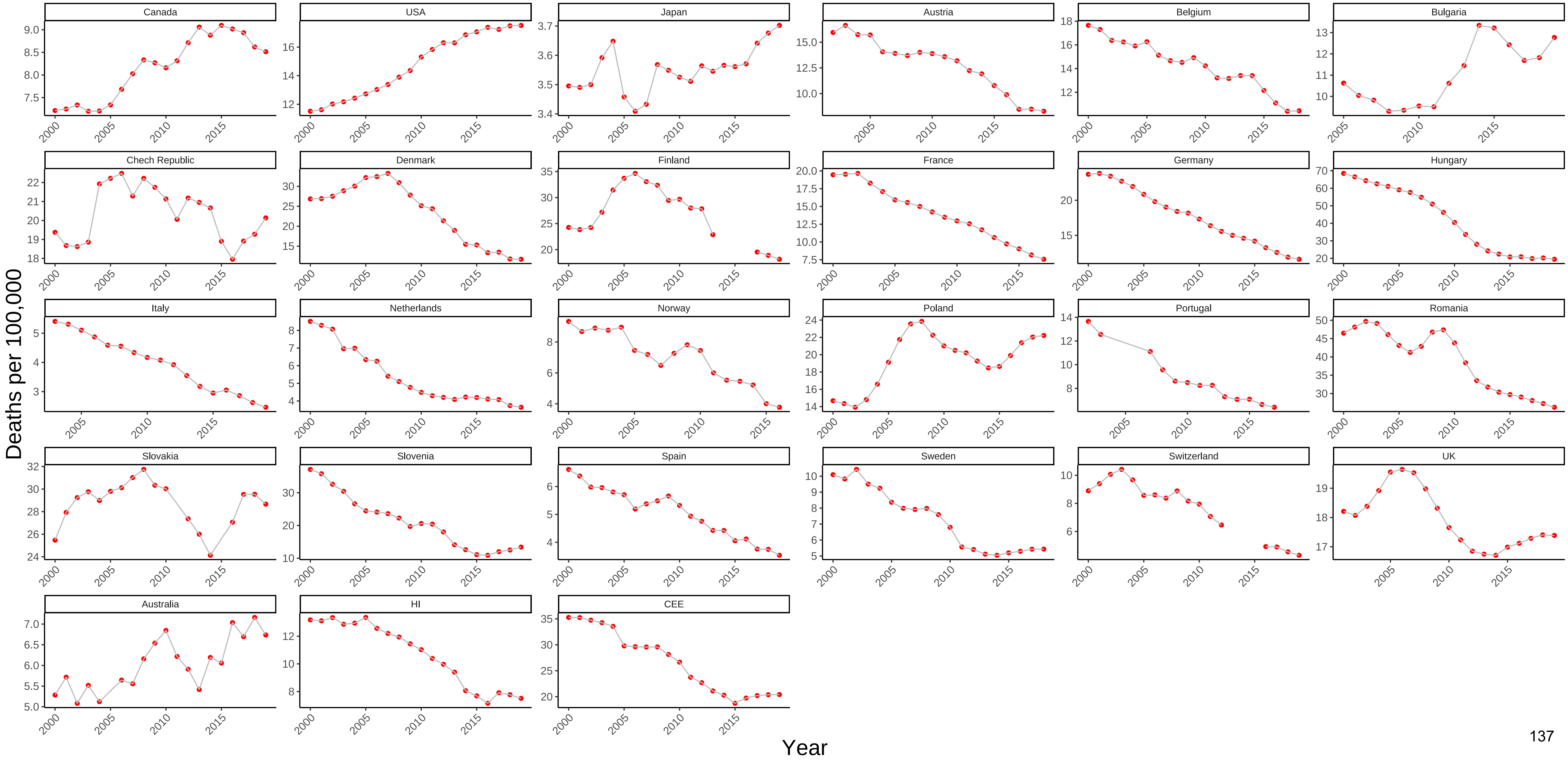

Figure S131. Three-Year Moving Average of Male Mortality from Drug-Related Causes at Ages 55-64

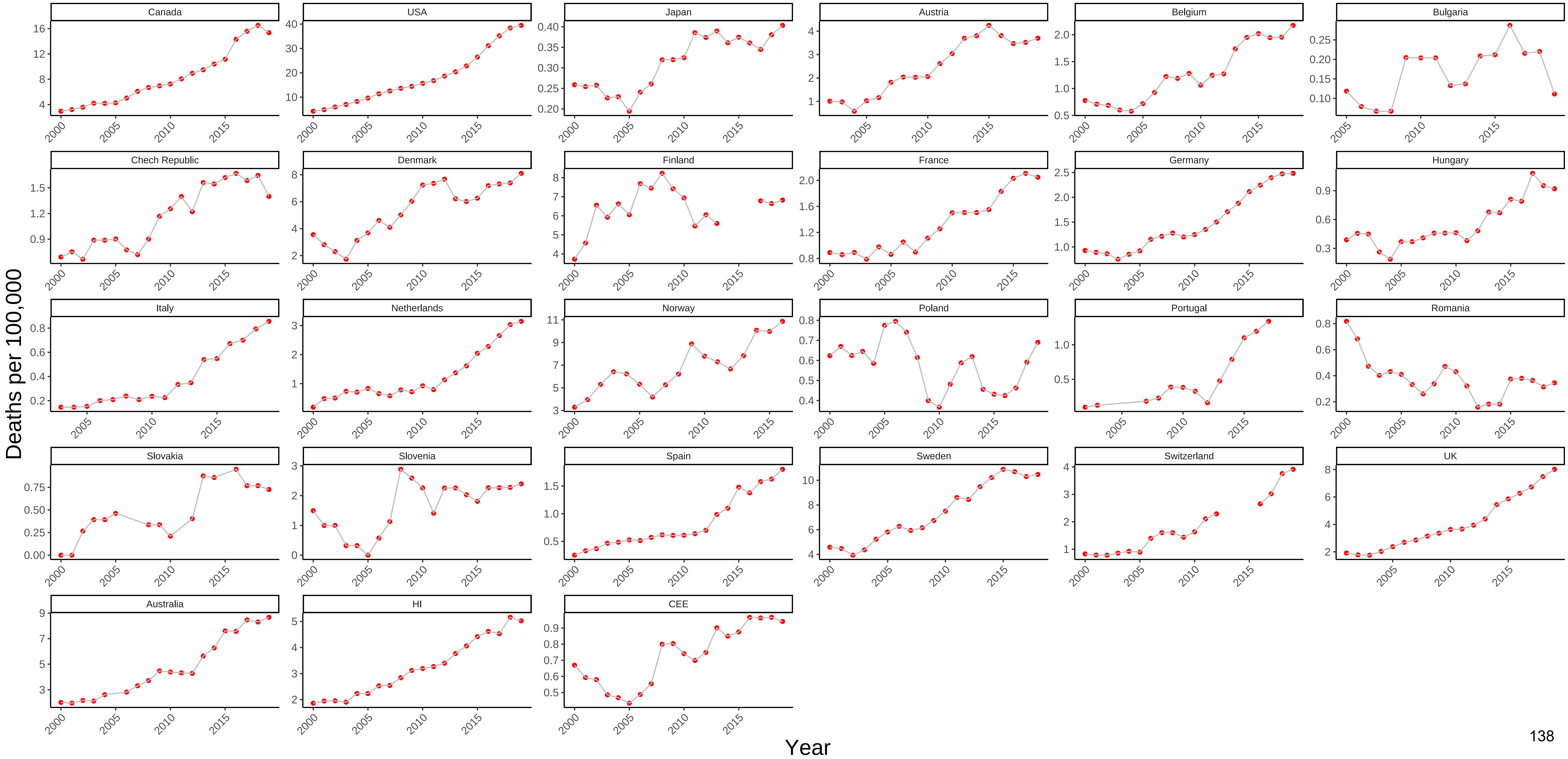

Figure S132. Three-Year Moving Average of Female Mortality from Drug-Related Causes at Ages 55-64

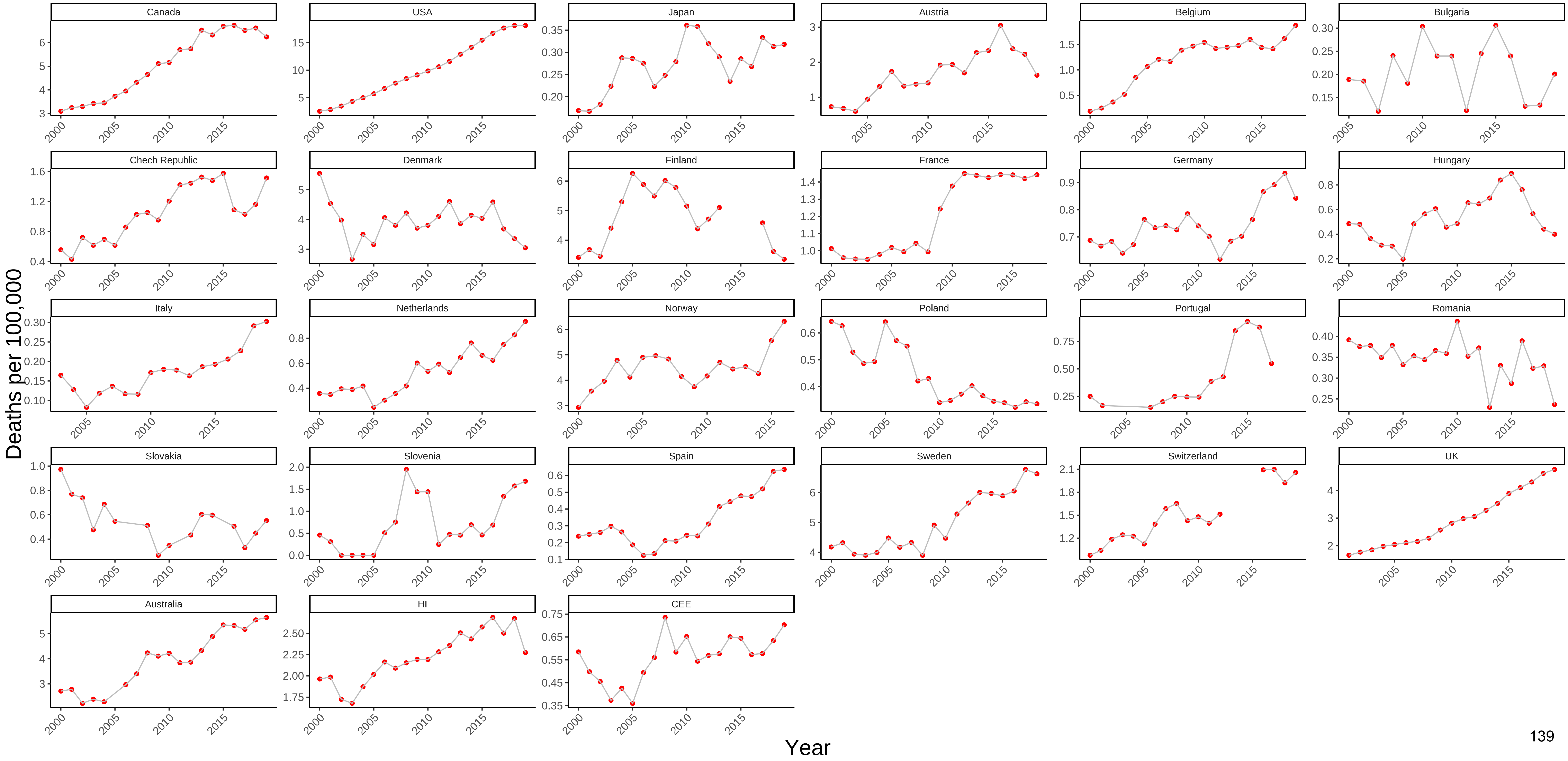

Figure S133. Three-Year Moving Average of Male Mortality from Alcohol-Related Causes at Ages 55-64

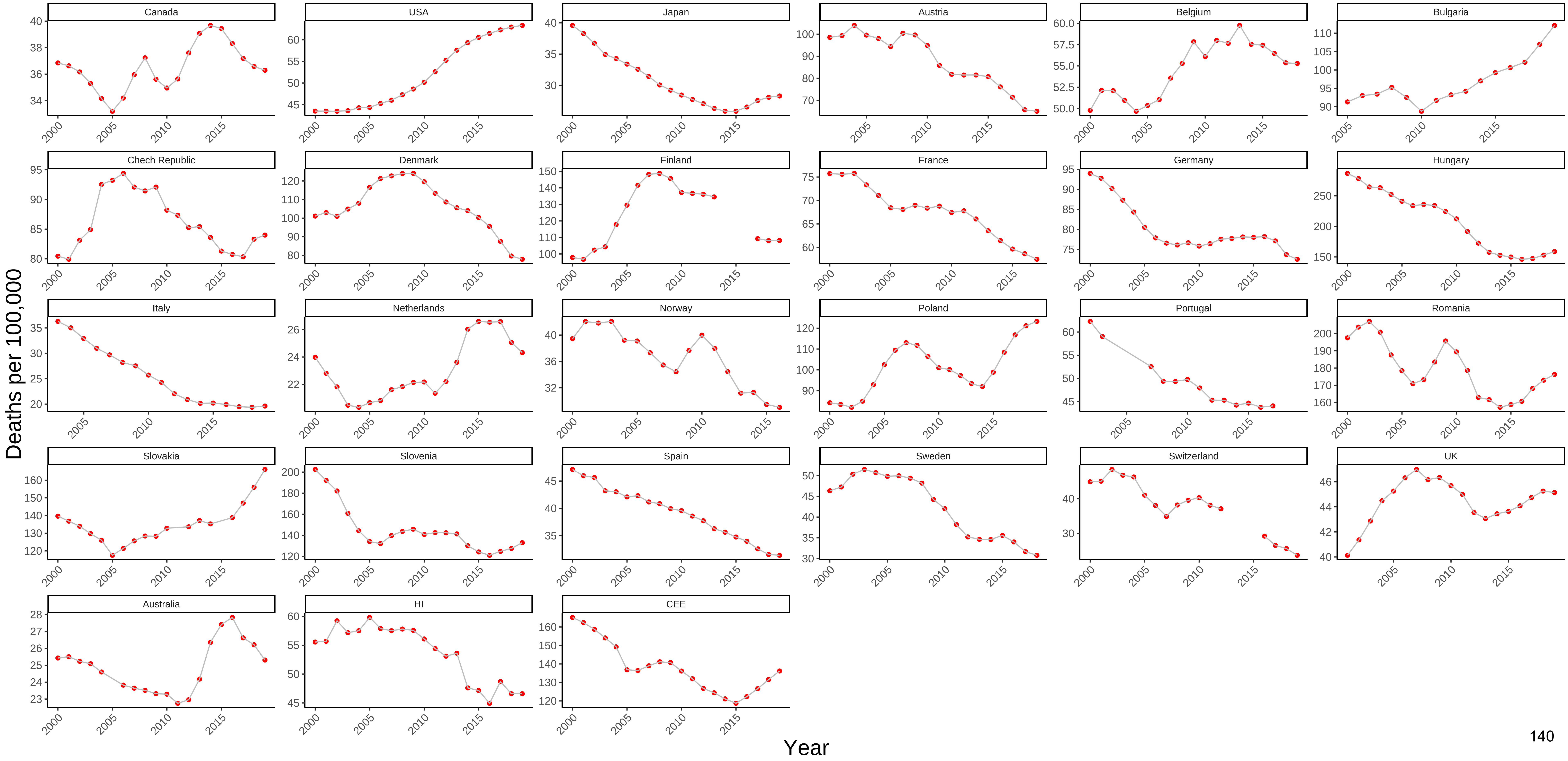

Figure S134. Three-Year Moving Average of Female Mortality from Alcohol-Related Causes at Ages 55-64

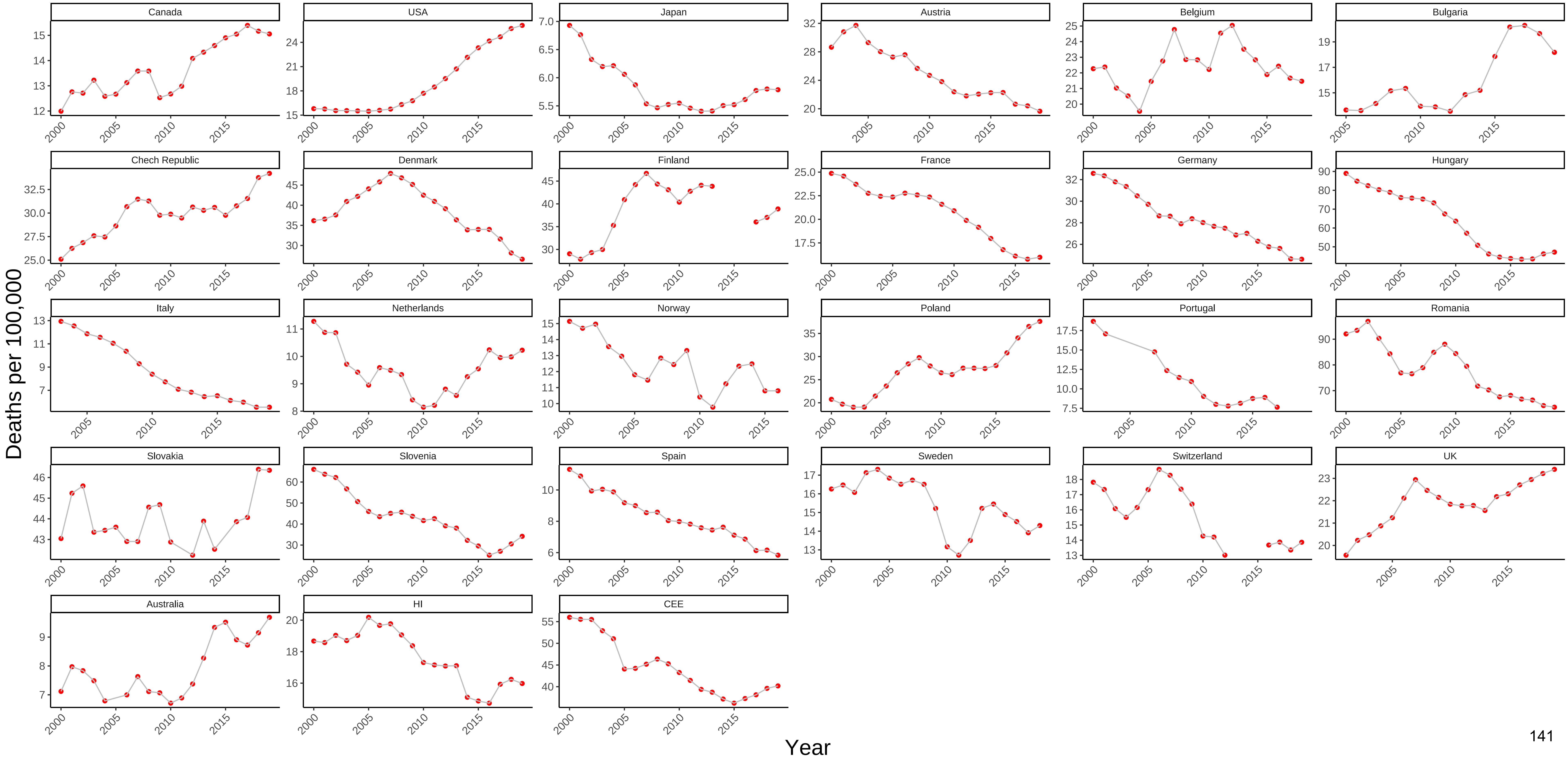

Supplement: dyae024_Supplementary_Data [file dyae024_supplementary_data.pdf]
